# Safety and immunogenicity of seven COVID-19 vaccines as a third dose (booster) following two doses of ChAdOx1 nCov-19 or BNT162b2 in the UK (COV-BOOST): a blinded, multicentre, randomised, controlled, phase 2 trial

**DOI:** 10.1016/S0140-6736(21)02717-3

**Published:** 2021-12-18

**Authors:** Alasdair P S Munro, Leila Janani, Victoria Cornelius, Parvinder K Aley, Gavin Babbage, David Baxter, Marcin Bula, Katrina Cathie, Krishna Chatterjee, Kate Dodd, Yvanne Enever, Karishma Gokani, Anna L Goodman, Christopher A Green, Linda Harndahl, John Haughney, Alexander Hicks, Agatha A van der Klaauw, Jonathan Kwok, Teresa Lambe, Vincenzo Libri, Martin J Llewelyn, Alastair C McGregor, Angela M Minassian, Patrick Moore, Mehmood Mughal, Yama F Mujadidi, Jennifer Murira, Orod Osanlou, Rostam Osanlou, Daniel R Owens, Mihaela Pacurar, Adrian Palfreeman, Daniel Pan, Tommy Rampling, Karen Regan, Stephen Saich, Jo Salkeld, Dinesh Saralaya, Sunil Sharma, Ray Sheridan, Ann Sturdy, Emma C Thomson, Shirley Todd, Chris Twelves, Robert C Read, Sue Charlton, Bassam Hallis, Mary Ramsay, Nick Andrews, Jonathan S Nguyen-Van-Tam, Matthew D Snape, Xinxue Liu, Saul N Faust, COV-BOOST study group, Andrew Riordan, Andrew Ustianowski, Chris A Rogers, Stephen Hughes, Laura Longshaw, Jane Stockport, Rachel Hughes, Lynne Grundy, Lona Tudor Jones, Arpan Guha, Emma Snashall, Tom Eadsforth, Sally Reeder, Kim Storton, Malathi Munusamy, Bridget Tandy, Akamino Egbo, Stephen Cox, Nabeela Nazir Ahmed, Anil Shenoy, Rachel Bousfield, Donna Wixted, Helen Gutteridge, Becky Mansfield, Christopher Herbert, Kyra Holliday, James Calderwood, Dominique Barker, Jacqueline Brandon, Hayley Tulloch, Suzie Colquhoun, Helen Thorp, Helen Radford, Julie Evans, Helena Baker, Jeanette Thorpe, Sally Batham, Jessica Hailstone, Rachael Phillips, Dileep Kumar, Fran Westwell, Fiona Makia, NinaSimone Hopkins, Lara Barcella, Mushiya Mpelembue, Maja dabagh, Matilda lang, Farida khan, Olumide Adebambo, Sunder Chita, Tumena Corrah, Ashley Whittington, Laurence John, Siobhan Roche, Lynda Wagstaff, Adam Farrier, Karen Bisnauthsing, Teona Serafimova, Elisa Nanino, Enya Cooney, Jaimie Wilson-Goldsmith, Hanna Nguyen, Andrea Mazzella, Beth Jackson, Suahil Aslam, Tanveer Bawa, Samantha Broadhead, Sadaf Farooqi, Jo Piper, Rowena Weighell, Lorinda Pickup, Djamila Shamtally, Jason Domingo, Evgenia Kourampa, Colin Hale, Jennifer Gibney, Michael Stackpoole, Zalina Rashid-Gardner, Rebecca Lyon, Chloe McDonnell, Christine Cole, Anna Stewart, Gillian McMillan, Mary Savage, Helen Beckett, Chantelle Moorbey, Amisha Desai, Claire Brown, Kush Naker, Ehsaan Qureshi, Charlotte Trinham, Charlotte Sabine, Sophie Moore, Steve Hurdover, Edwin Justice, David Smith, Emma Plested, Carla Ferreira Da Silva, Rachel White, Hannah Robinson, Liliana Cifuentes, Gertraud Morshead, Rachael Drake-Brockman, Patrick Kinch, Mwila Kasanyinga, Elizabeth A. Clutterbuck, Sagida Bibi, Arabella SV Stuart, Robert H Shaw, Michael Singh, Trishna Champaneri, Margaret Irwin, Mohammed Khan, Alicja Kownacka, Martha Nabunjo, Carool Osuji, John Hladkiwskyj, Dominic Galvin, Gita Patel, Johanna Mouland, Beverley Longhurst, Maria Moon, Beth Giddins, Carlota Pereira Dias Alves, Leah Richmond, Christine Minnis, Sonia Baryschpolec, Scott Elliott, Lauren Fox, Victoria Graham, Natalie Baker, Kerry Godwin, Karren Buttigieg, Chanice Knight, Phillip Brown, Paminder Lall, Imam Shaik, Emily Chiplin, Emily Brunt, Stephanie Leung, Lauren Allen, Steve Thomas, Sara Fraser, Bea Choi, Jade Gouriet, Andrew Freedman, Jonathan Perkins, Andrew Gowland, Jonathan Macdonald, John Paul Seenan, Igor Starinskij, Andrew Seaton, Erica Peters, Stephen Singh, Ben Gardside, Avril Bonnaud, Ceri Davies, Elizabeth Gordon, Samantha Keenan, Jane Hall, Suzanne Wilkins, Suzanne Tasker, Rob James, Ingrid Seath, Kelly Littlewood, Joseph Newman, Iryna Boubriak, Debbie Suggitt, Helen Haydock, Sara Bennett, Wiesia Woodyatt, Kerry Hughes, Judith Bell, Tricia Coughlan, Donald van Welsenes, Mohammed Kamal, Chris Cooper, Simon Tunstall, Nicholas Ronan, Rebecca Cutts, Tracey Dare, Yee Ting Nicole Yim, Sarah Whittley, Marivic Ricamara, Shama Hamal, Kirsty Adams, Holly Baker, Kimberley Driver, Nicola Turner, Todd Rawlins, Subarna Roy, Marta Merida-Morillas, Yukari Sakagami, Antonette Andrews, Lillian Goncalves cordeiro, Matthew Stokes, Wythehi Ambihapathy, Joanne Spencer, Nina Parungao, Lisa Berry, James Cullinane, Laura Presland, Amy Ross-Russell, Sarah Warren, Jonathan Baker, Abigail Oliver, Amanda Buadi, Kim Lee, Louise Haskell, Rossana Romani, Ian Bentley, Tim Whitbred, Simon Fowler, John Gavin, Alan Magee, Tara Watson, Kari Nightingale, Phedra Marius, Eloise Summerton, Emily Locke, Thomas Honey, Aidan Lingwood, Anastasia de la Haye, Ryan Stephen Elliott, Karen Underwood, Mikayala King, Sharon Davies-Dear, Emily Horsfall, Olivia Chalwin, Holly Burton, Christopher J Edwards, Benjamin Welham, Sarah Garrahy, Fran Hall, Eleni Ladikou, Dee Mullan, Daniel Hansen, Marion Campbell, Filipa Dos Santos, Haniah Habash-Bailey, Nicki Lakeman, Debbie Branney, Luke Vamplew, Alison Hogan, Jorden Frankham, Martin Wiselka, Dennyl Vail, Victoria Wenn, Valerie Renals, Kate Ellis, Jessica Lewis-Taylor, Javier Magan, Anna Hardy, Kim Appleby

**Affiliations:** a NIHR Southampton Clinical Research Facility and Biomedical Research Centre, University Hospital Southampton NHS Foundation Trust, Southampton, UK; b Faculty of Medicine and Institute for Life Sciences, University of Southampton, Southampton, UK; c Imperial Clinical Trials Unit, Imperial College London, London, UK; d Oxford Vaccine Group, Department of Paediatrics, University of Oxford, Oxford, UK; e Cancer Research UK Oxford Centre, University of Oxford, Oxford, UK; f Jenner Institute, Nuffield Department of Medicine, University of Oxford, Oxford, UK; g NIHR Oxford Biomedical Research Centre, Oxford, UK; h Stockport NHS Foundation Trust, Stockport, UK; i NIHR Liverpool and Broadgreen Clinical Research Facility, Liverpool, UK; j NIHR Cambridge Clinical Research Facility, Cambridge University Hospitals NHS Foundation Trust, Cambridge, UK; k PHARMExcel, Welwyn Garden City, Hertfordshire, UK; l NIHR/Wellcome Clinical Research Facility, University Hospitals Birmingham NHS Foundation Trust, Birmingham, UK; m Department of Infection, Guy's and St Thomas’ NHS Foundation Trust, London, UK; n MRC Clinical Trials Unit, University College London, London, UK; o Portsmouth Hospitals University NHS Trust, Portsmouth, UK; p Queen Elizabeth University Hospital, NHS Greater Glasgow & Clyde, Glasgow, UK; q Wellcome-MRC Institute of Metabolic Science, Department of Clinical Biochemistry, University of Cambridge, Cambridge, UK; r NIHR UCLH Clinical Research Facility and NIHR UCLH Biomedical Research Centre, University College London Hospitals NHS Foundation Trust, London, UK; s University Hospitals Sussex NHS Foundation Trust, Brighton, UK; t Department of Infectious Diseases and Tropical Medicine, London Northwest University Healthcare, London, UK; u The Adam Practice, Poole, UK; v NIHR Leeds Clinical Research Facility, Leeds Teaching Hospitals Trust and University of Leeds, Leeds, UK; w North Wales Clinical Research Facility, Betsi Cadwaladr University Health Board and Bangor University, Bangor, UK; x Department of Molecular and Clinical Pharmacology, University of Liverpool, Liverpool, UK; y University Hospitals of Leicester NHS Trust, University of Leicester, Leicester, UK; z Bradford Institute for Health Research and Bradford Teaching Hospitals NHS Foundation Trust, Bradford, UK; aa Royal Devon and Exeter Hospital NHS Foundation Trust, Exeter, UK; ab MRC University of Glasgow Centre for Virus Research, Glasgow, UK; ac UK Health Security Agency, Porton Down, UK; ad UK Health Security Agency, Colindale, London, UK; ae Division of Epidemiology and Public Health, University of Nottingham School of Medicine, Nottingham, UK

## Abstract

**Background:**

Few data exist on the comparative safety and immunogenicity of different COVID-19 vaccines given as a third (booster) dose. To generate data to optimise selection of booster vaccines, we investigated the reactogenicity and immunogenicity of seven different COVID-19 vaccines as a third dose after two doses of ChAdOx1 nCov-19 (Oxford–AstraZeneca; hereafter referred to as ChAd) or BNT162b2 (Pfizer–BioNtech, hearafter referred to as BNT).

**Methods:**

COV-BOOST is a multicentre, randomised, controlled, phase 2 trial of third dose booster vaccination against COVID-19. Participants were aged older than 30 years, and were at least 70 days post two doses of ChAd or at least 84 days post two doses of BNT primary COVID-19 immunisation course, with no history of laboratory-confirmed SARS-CoV-2 infection. 18 sites were split into three groups (A, B, and C). Within each site group (A, B, or C), participants were randomly assigned to an experimental vaccine or control. Group A received NVX-CoV2373 (Novavax; hereafter referred to as NVX), a half dose of NVX, ChAd, or quadrivalent meningococcal conjugate vaccine (MenACWY)control (1:1:1:1). Group B received BNT, VLA2001 (Valneva; hereafter referred to as VLA), a half dose of VLA, Ad26.COV2.S (Janssen; hereafter referred to as Ad26) or MenACWY (1:1:1:1:1). Group C received mRNA1273 (Moderna; hereafter referred to as m1273), CVnCov (CureVac; hereafter referred to as CVn), a half dose of BNT, or MenACWY (1:1:1:1). Participants and all investigatory staff were blinded to treatment allocation. Coprimary outcomes were safety and reactogenicity and immunogenicity of anti-spike IgG measured by ELISA. The primary analysis for immunogenicity was on a modified intention-to-treat basis; safety and reactogenicity were assessed in the intention-to-treat population. Secondary outcomes included assessment of viral neutralisation and cellular responses. This trial is registered with ISRCTN, number 73765130.

**Findings:**

Between June 1 and June 30, 2021, 3498 people were screened. 2878 participants met eligibility criteria and received COVID-19 vaccine or control. The median ages of ChAd/ChAd-primed participants were 53 years (IQR 44–61) in the younger age group and 76 years (73–78) in the older age group. In the BNT/BNT-primed participants, the median ages were 51 years (41–59) in the younger age group and 78 years (75–82) in the older age group. In the ChAd/ChAD-primed group, 676 (46·7%) participants were female and 1380 (95·4%) were White, and in the BNT/BNT-primed group 770 (53·6%) participants were female and 1321 (91·9%) were White. Three vaccines showed overall increased reactogenicity: m1273 after ChAd/ChAd or BNT/BNT; and ChAd and Ad26 after BNT/BNT. For ChAd/ChAd-primed individuals, spike IgG geometric mean ratios (GMRs) between study vaccines and controls ranged from 1·8 (99% CI 1·5–2·3) in the half VLA group to 32·3 (24·8–42·0) in the m1273 group. GMRs for wild-type cellular responses compared with controls ranged from 1·1 (95% CI 0·7–1·6) for ChAd to 3·6 (2·4–5·5) for m1273. For BNT/BNT-primed individuals, spike IgG GMRs ranged from 1·3 (99% CI 1·0–1·5) in the half VLA group to 11·5 (9·4–14·1) in the m1273 group. GMRs for wild-type cellular responses compared with controls ranged from 1·0 (95% CI 0·7–1·6) for half VLA to 4·7 (3·1–7·1) for m1273. The results were similar between those aged 30–69 years and those aged 70 years and older. Fatigue and pain were the most common solicited local and systemic adverse events, experienced more in people aged 30–69 years than those aged 70 years or older. Serious adverse events were uncommon, similar in active vaccine and control groups. In total, there were 24 serious adverse events: five in the control group (two in control group A, three in control group B, and zero in control group C), two in Ad26, five in VLA, one in VLA-half, one in BNT, two in BNT-half, two in ChAd, one in CVn, two in NVX, two in NVX-half, and one in m1273.

**Interpretation:**

All study vaccines boosted antibody and neutralising responses after ChAd/ChAd initial course and all except one after BNT/BNT, with no safety concerns. Substantial differences in humoral and cellular responses, and vaccine availability will influence policy choices for booster vaccination.

**Funding:**

UK Vaccine Taskforce and National Institute for Health Research.

## Introduction

Although most studies suggest well preserved protection against severe COVID-19 disease and death from deployed vaccines, even with the delta (B.1.617.2) strain predominant,^1^ observational data suggest there is a progressive reduction in protection against any infection or symptomatic infection.^2^, ^3^, ^4^, ^5^

As protection against SARS-CoV-2 infection has waned after a two-dose schedule of COVID-19 vaccines, policy makers have begun to consider the implications for periodic or seasonal third dose, also known as a booster, vaccination against COVID-19 to protect the most vulnerable patients, and mitigate health-care and economic impacts. Decisions regarding when and which populations to whom boosters should be administered are made based on real-world data and cohort studies.^1^, ^3^, ^6^, ^7^ We set out to generate data to optimise selection of booster vaccines after two doses of ChAdOx1 nCov-19 (Oxford–AstraZeneca; hereafter referred to as ChAd) or BNT162b2 (Pfizer–BioNtech, hearafter referred to as BNT). There are theoretical reasons why providing a mixed schedule might provide greater protection. The UK COMCOV trial demonstrated that a heterologous prime-boost schedule can be more immunogenic than a homologous schedule,^8^, ^9^ albeit with increased reactogenicity in some combinations.^10^ We also investigated the effect of reduced (ie, fractional) dosing for third doses of three vaccines on reactogenicity and immunogencity. If viral killing can be maintained despite using a reduced dose compared with that previously tested in phase 3 trials,^11^, ^12^, ^13^ it might be possible to reduce reactogenicity for third dose recipients and increase global reach of finite vaccine supply.

The importance of both cellular and humoral immunity in vaccine effectiveness is evident from real-world studies that have demonstrated little difference in initial protection from infection^14^ or from severe disease and death^1^, ^3^ after ChAd/ChAd or BNT/BNT, despite BNT generating levels of neutralising antibodies many times higher than ChAd.^8^ The importance of antibody-mediated immunity in protection against SARS-CoV-2 infection has been demonstrated in non-human primates and humans from both infection and immunisation, although correlates of protection against asymptomatic infection are not yet clear.^15^, ^16^, ^17^, ^18^ T cells are important in controlling disease severity, although correlates of protection against symptomatic disease have not yet been demonstrated.^19^ T-cell activity appears minimally affected by spike antigen mutations^20^ and responses remain against variants of concern, even though neutralising antibody levels are reduced.^21^ Although neutralising antibody titres after mRNA vaccination are higher compared with adenovirus vector vaccines, there are much smaller differences in T-cell responses.^8^ T-cell responses have also been shown to be higher in heterologous schedules compared with homologous—eg, mRNA second dose after adenoviral vector prime.^8^

**Research in context**

**Evidence before this study**

We searched PubMed for randomised controlled trials in non-immunocompromised adults published between database inception and Nov 3, 2021, using the terms “(COVID) AND (vaccin*) AND (booster OR third dose)” with no language restrictions. We identified three published clinical trials of ChAdOx1 nCov-19 (Oxford–AstraZeneca; hereafter referred to as ChAd), BNT162b2 (Pfizer–BioNtech; hearafter referred to as BNT), and mRNA1273 (Moderna) homologous third dose boosters. For all vaccines, neutralising antibody titres were significantly boosted by a third dose of vaccine, including against the delta variant for mRNA1273 and BNT. Reactogenicity was reported as similar to after dose two of BNT, and lower than after dose two of ChAd. T-cell responses were significantly boosted by a third dose of ChAd, and not reported for mRNA1273 or BNT. We also identified two preprints, including a small trial of homologous and heterologous third dose boosters for BNT, mRNA1273, and Ad26.COV2.S (Janssen; hereafter referred to as Ad26), which demonstrated similar reactogenicity to the original immunisation series, and improved neutralising antibody titres for heterologous boosting. A small Chinese trial of Convidecia (CanSino Biologicals) or CoronaVac (Sinovac Biotech) following a primary immunisation series with CoronaVac demonstrated increased reactogenicity with a heterologous third dose, with accompanying increased neutralising antibody titers. Cellular immunity was not reported for either study.

**Added value of this study**

This was, to our knowledge, the first randomised trial of third dose booster vaccines given 10–12 weeks after an initial course of ChAd/ChAd or BNT/BNT COVID-19 immunisation. This trial has demonstrated the potential of all vaccines tested (ChAd, BNT, mRNA1273, NVX-CoV2373 [Novavax; hereafter referred to as NVX], Ad26, CVnCov [CureVac; hereafter referred to as CVn], and VLA2001 [Valneva; hereafter referred to as VLA]) to boost immunity after an initial course of ChAd/ChAd and of six vaccines (ChAd, BNT, mRNA1273, NVX, Ad26, and CVn) after an initial course of BNT/BNT. All vaccines showed acceptable side-effect profiles, although some schedules were more reactogenic than others.

**Implications of all the available evidence**

Policy makers and national immunisation advisory committees should establish criteria for choosing which booster vaccines to use in their populations. This decision should be based on immunological considerations, known side-effect profiles, in-country availability, and ultimately a decision on what level of boost is sufficient in the context of national strategic disease control objectives. Data from our study and others suggest that current mRNA doses might be higher than required to provide adequate boost to immunity after a third dose. Interpretation should avoid focus on the headline spike IgG levels or antibody boost ratios because the relationship between antibody levels at day 28 and long-term protection and immunological memory is unknown.

In this The Evaluating COVID-19 Vaccination Boosters (COV-BOOST) trial, we investigated the reactogenicity and immunogenicity of seven different COVID-19 vaccines, with three at full and half dose, as a third dose after ChAd/ChAd or BNT/BNT.

## Methods

### Trial design

COV-BOOST is a multicentre, randomised, phase 2 trial of third dose booster vaccination against COVID-19, with a subgroup to investigate detailed immunology. The study was conducted at 18 UK sites, in a mixture of community and secondary care settings. To reduce the risk of vaccine administration error and delays if there were problems in the supply of one or more vaccines, the ten experimental vaccine and control groups (seven vaccines with three also at half dose and controls) were split into three groups with six sites per group.

The trial was reviewed and approved by the South-Central Berkshire Research Ethics Committee, University Hospital Southampton, and the Medicines and Healthcare Products Regulatory Agency (EudraCT 2021–002175–19, IRAS 299180, REC reference 21/SC/0171). The study protocol is provided in appendix 2 (pp 2–103).

### Participants

Participants were adults aged 30 years or older, in good physical health (mild to moderate well controlled comorbidities were permitted), who had received two doses of either BNT or ChAd (first dose in December, 2020, January, 2021, or February, 2021), and were at least 84 days post second dose by the time of enrolment. Due to timelines of UK vaccine deployment, some sites were permitted to enrol participants who were at least 70 days post second dose of ChAd. The timing of the trial meant that enrolment included people aged 75 years or older, health and social care workers, and people residing in care homes. Enrolment was managed so that approximately half of participants had received two doses of ChAd and half two doses of BNT, and approximately half of people were aged 70 years or older. Full exclusion and inclusion criteria listed in appendix 2 (pp 33–35). Participants provided written informed consent.

### Randomisation and masking

An unblinded statistician created the computer-generated randomisation list. Randomisation schedules were prepared separately for participants primed with ChAd/ChAd and BNT/BNT and stratified by study site, age (<70 years and ≥70 years) and subgroup (general and immunology). Permuted random blocks were used, and participants were randomly assigned to the study groups with equal probability within groups A–C. Group A received NVX-CoV2373 (Novavax; hereafter referred to as NVX), a half dose of NVX, ChAd, or control quadrivalent meningococcal conjugate vaccine (MenACWY; 1:1:1:1). Group B received BNT, VLA2001 (Valneva; hereafter referred to as VLA), a half dose of VLA, Ad26.COV2.S (Janssen; hereafter referred to as Ad26) or MenACWY (1:1:1:1:1). Group C received mRNA1273 (Moderna**;** hereafter referred to as m1273), CVnCov (CureVac; hereafter referred to as CVn), a half dose of BNT, or MenACWY (1:1:1:1). Each cohort (A, B, and C) had their own control group and recruited two separate populations, those receiving ChAd/ChAd and those receiving BNT/BNT. Randomisation was done in the electronic data capture system REDCap, version 10.6.13.

Participants, laboratory staff, and the clinical study team not delivering the vaccines were blind to treatment allocation, including those undertaking adverse event assessments. Participant blinding to vaccines was maintained by concealing randomisation pages, preparing vaccines out of sight, and applying masking tape to vaccine syringes to conceal dose, volume, and appearance. The analysing statisticians remained blind until the statistical analysis plan was signed off.

### Procedures

Participants who met the inclusion and exclusion criteria via the online screening or the telephone screening (or both) were invited to a baseline visit (day 0). Participants who passed the final eligibility assessment were randomly assigned to a study group.

Seven COVID-19 vaccines, and three with a half dose, were used (table 1); all vaccines were administered via intramuscular injection into the upper arm.

**Table 1:** Vaccines used in COV-BOOST trial

	**Mechanism of action**	**Administration**
ChAdOx-nCov19 (ChAd; AZD1222, AstraZeneca)	Replication-deficient chimpanzee adenovirus vectored vaccine, expressing the SARS-CoV-2 spike surface glycoprotein	5 × 10^10^ viral particles per 0·5 mL via intramuscular injection
NVX-CoV2373 (NVX; Novavax)	Nanoparticle vaccine containing purified spike glycoprotein	5 μg with Matrix-M1 50 μg adjuvant in 0·5 mL via intramuscular injection
NVX-CoV2373 (NVX half; Novavax)	Nanoparticle vaccine containing purified spike glycoprotein	2·5 μg with Matrix-M1 25 μg adjuvant in 0·25 mL via intramuscular injection
BNT162b2 (BNT; Pfizer–BioNTech)	mRNA vaccine encoding SARS-CoV-2 spike glycoprotein	30 μg in 0·3 mL via intramuscular injection
BNT162b2 (BNT half; Pfizer–BioNTech)	mRNA vaccine encoding SARS-CoV-2 spike glycoprotein	15 μg in 0·15 mL via intramuscular injection
VLA2001 (VLA; Valneva)	Whole, inactivated SARS-CoV-2 virus	33 antigen units with 1 mg CpG adjuvant in 0·5 mL via intramuscular injection
VLA2001 (VLA half; Valneva)	Whole, inactivated SARS-CoV-2 virus	16·5 antigen units with 0·5 mg CpG adjuvant in 0·25 mL via intramuscular injection
Ad26.COV2.S (Ad26; Janssen)	Replication-deficient adenovirus vector vaccine constructed to encode a full-length spike protein	5 × 10^10^ viral particles per mL in 0·5 mL via intramuscular injection
mRNA1273 (m1273; Moderna)	mRNA vaccine encoding SARS-CoV-2 spike glycoprotein	100 μg administered via 0·5 mL via intramuscular injection
CVnCoV (CVn; Curevac), withdrawn from further clinical development October, 2021^22^	mRNA vaccine encoding SARS-CoV-2 spike glycoprotein	12 μg administered via 0·6 mL via intramuscular injection
MenACWY (Pfizer) control	Quadrivalent meningococcal conjugate vaccine	0·5 mL via intramuscular injection

Participants attended screening and vaccination at day 0. Blood was taken for immunogenicity analyses at days 28, 84, and 365. A separate immunology subgroup comprised of 25 individuals from each treatment group (n=650 participants) attended additional visits to have blood taken at day 7 (to detect evidence of previous immunological priming via rapid spike IgG responses) and day 14 (to detect the peak T-cell response). The cellular immunology samples were collected from nine sites based on logistical reasons (proximity to external laboratory). The immunology subgroup was allocated at six of these sites, including three sites recruiting immunology subgroup only and the other three sites enrolling participants up to the required number.

Vaccines were administered by appropriately trained trial staff at trial sites. Participants were observed for at least 15 min after vaccination. During the baseline visit, participants were given an oral thermometer, tape measure, and diary card (electronic or paper) to record solicited adverse events on day 7, unsolicited adverse events on day 28, and medically attended adverse events on day 84. The study sites’ physicians reviewed the diary card regularly to record adverse events, adverse events of special interest, and serious adverse events. The timepoints for subsequent visits for immunogenicity blood sampling are shown in the protocol (appendix 2 p 81). During the study visits, adverse events, adverse events of special interest, and serious adverse events that had not been recorded in the diary card were also collected.

Participants who tested positive for SARS-CoV-2 in the community were invited for an additional visit for clinical assessment, collection of blood samples, and throat swab, and completion of a COVID-19 symptom diary.

Detailed methods for SARS-CoV-2 anti-spike IgG concentrations by ELISA (reported as ELISA laboratory units [ELU]/mL) and SARS-CoV-2 pseudotype virus neutralisation (PNA) assays (Nexelis, Laval, QC, Canada) and for T-cell assays (Oxford Immunotec Abingdon, UK) are provided in appendix 1 (p 41).

### Outcomes

The coprimary outcomes were safety and reactogenicity, and immunogenicity. Safety and reactogenicity were determined by the occurrence of solicited, unsolicited adverse events, adverse events of special interest, or serious adverse events following vaccination, as recorded in participant electronic diaries or ascertained at follow-up visits. Immunogenicity outcome was anti-spike protein IgG at day 28 follow-up.

Secondary endpoints included other immunogenicity assays such as neutralising antibody titres against wild-type (ie, the original strain identified in Wuhan), and pseudovirus neutralisation, and T-cell response (measured by ELISpot) against wild-type and SARS-CoV-2 virus variants of concern: alpha (B.1.1.7), beta (B.1.351), and delta. A complete list of secondary endpoints is provided in appendix 2 (pp 17–20).

### Statistical analysis

We powered on the immunogenicity outcome and designed to have 90% power to compare the geometric mean concentration (GMC) of anti-spike IgG between each COVID-19 vaccine group with the MenACWY group within each of the three groups (A and C [three comparisons], and B [four comparisons]) and populations (ChAd/ChAd and BNT/BNT). Since we made, at most, four comparisons within a cohort, using a Bonferroni correction would need to adjust for a significance level of 0·05/4 = 0·0125. To account for multiple testing within each cohort a conservative two-sided significance level of 0·01 was used. We required 83 participants per group to detect an established minimum clinically important difference of 1·75-times difference in GMC assuming a log10-SD of 0·4. The minimum clinical important difference was chosen based on the discussions with UK policy makers and regulatory agency. We inflated the required sample size by 25% to take account of participants who would be seropositive at baseline or lost to follow-up, recruiting n=111 per group. A subset of n=25 per group were included in the immunology substudy, as the purpose of this substudy was descriptive we did not undertake a power calculation.

All analyses were stratified by prime series of vaccination (ChAd/ChAd and BNT/BNT). The primary analysis for immunogenicity outcomes was on a modified intention-to-treat (mITT) basis to include all participants who were seronegative at baseline (defined by the Roche Elecsys anti-Sars-CoV-2 assay) with no confirmed SARS-CoV-2 infection within 14 days post third dose, and with endpoint data available. The primary immunogenicity outcome of anti-spike IgG at day 28 in each group was reported as GMC and 95% CI. The geometric mean ratio (GMR) and 99% CIs (to account for multiple comparisons) of anti-spike IgG between each experimental group and the corresponding control group was also reported. The GMR was calculated as the antilogarithm of the difference between the mean of the log10 transformed anti-spike IgG in the experimental group and that in the control group. The original analysis plan included use of a linear mixed effect model with site as a random effect, but due to model convergence, a linear regression with site as a fixed effect was used. The model computed the difference of log10 transformed anti-spike IgG after adjusting for baseline immunogenicity and randomisation design variables (ie, study site and age group), duration between first and second vaccine, and the duration between second to third dose vaccine. Residual analysis was done to examine model assumptions.

The analysis population for reactogenicity and safety included all randomly assigned participants who received a study vaccine, including both seronegative and seropositive populations at baseline. The primary outcome of reactogenicity examined solicited adverse events (local and systemic) within the first 7 days. The proportion with at least one severe episode (grade 3 and grade 4) are presented for each of the groups (A, B, and C) and priming vaccine by vaccine group. An additional view of reactogenicity outcomes displays severity of the event by vaccine group and stratified by priming vaccine and age group. Unsolicited adverse events reported within 28 days post third dose were coded according to the Medical Dictionary for Regulatory Activities and tabulated at System Organ Class level across vaccine groups. Adverse events of special interest and serious adverse events were reported until the data lock date of Aug 19, 2021, by line listing.

Secondary immunogenicity outcomes were analysed with the same approach as for the primary immunogenicity outcome. We repeated the analyses for primary and secondary outcomes in the group aged 30–69 years and the group aged 70 years and older separately, as subgroup analysis. For descriptive statistics, secondary outcomes, and subgroup analysis, we reported GMRs with 95% CIs.

### Role of the funding source

The funders of the study had no role in study design, data collection, data analysis, data interpretation, or writing of the report.

## Results

**Figure 1:**
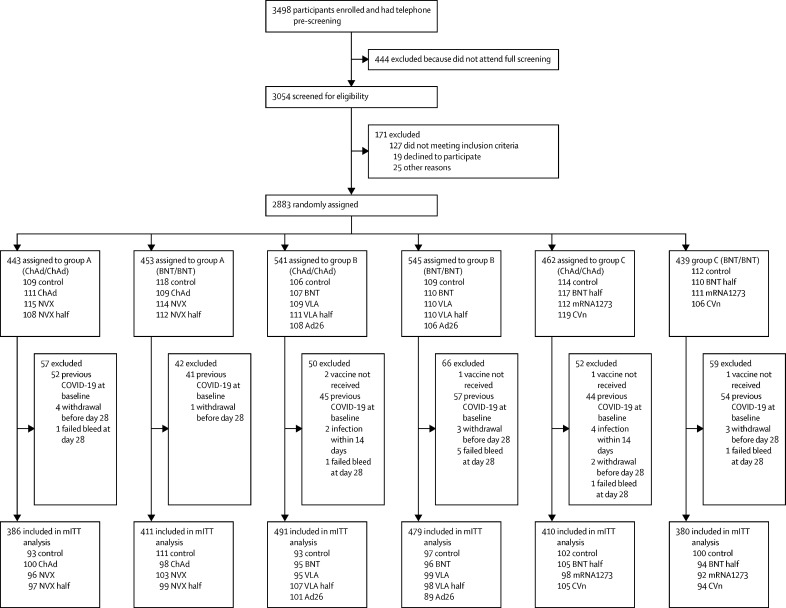
Trial profile Control=quadrivalent meningococcal conjugate vaccine. ChAd=ChAdOx1 nCoV-19 vaccine, Oxford–AstraZeneca. NVX=NVX-CoV2373 vaccine, Novavax. NVX half=half dose of NVX-CoV2373 vaccine. BNT=BNT162b2 vaccine, Pfizer–BioNTech. VLA=VLA2001 vaccine, Valneva. VLA half=half dose of VLA2001 vaccine. Ad26=Ad26.COV2.S vaccine, Janssen. BNT half=half dose of BNT162b2 vaccine. m1273=mRNA1273 vaccine, Moderna. CVn=CVnCoV vaccine, Curevac. mITT=modified intention to treat.

Between June 1 and June 30, 2021, 3498 people were screened, of whom 2883 were recruited (figure 1). Five participants withdrew before vaccination, leading to 2878 participants receiving a third dose vaccine.

The median age of ChAd/ChAd-primed participants was 53 years (IQR 44–61) in the younger age group and 76 years (73–78) in the older age group (Table 2, Table 3, Table 4). In the BNT/BNT-primed participants, the median age was 51 years (41–59) in the younger age group and 78 years (75–82) in the older age group. In the ChAd/ChAD-primed group 676 (46·7%) participants were female and 1380 (95·4%) were White, and in the BNT/BNT-primed group, 770 (53·6%) participants were female and 1321 (91·9%) were White. In the younger age group, 409 (57·6%) were female, 665 (93·7%) were White in the ChAD/ChAD-primed group, and 518 (64·0%) were female, and 709 (87·6%) were White in the BNT/BNT-primed group. In the older age group, 267 (36·3%) were female and 715 (97·1%) were White in the ChAD/ChAD-primed group, and 252 (40·1%) were female and 612 (97·5%) were White in the BNT/BNT-primed group. Because BNT was rolled out at a 3-week interdose interval at the beginning of the UK vaccination campaign, mainly in older people and health-care workers, and before ChAd was deployed, we observed expected differences in baseline characteristics between the ChAd/ChAd-primed group and the BNT/BNT-primed group. Within the ChAd/ChAd-primed or BNT/BNT-primed populations, the baseline characteristics were well balanced between the randomly assigned groups for each group of A–C.

**Table 2:** Baseline characteristics by third dose vaccine allocation and priming vaccine schedule, group A

		**Prime with ChAd/ChAd**				**Prime with BNT/BNT**			
		Control (n=109)	ChAd (n=111)	NVX (n=115)	NVX half (n=108)	Control (n=118)	ChAd (n=109)	NVX (n=114)	NVX half (n=112)
Age, years									
	Mean (SD)	64·0 (14·0)	63·7 (14·1)	63·5 (13·7)	61·8 (15·1)	63·1 (16·9)	61·9 (16·6)	62·1 (16·4)	62·9 (16·0)
	Median (IQR)	68·1 (55·1–75·9)	67·8 (52·2–75·7)	65·3 (52·6–74·1)	65·8 (49·9–75·6)	62·4 (49·4–78·5)	61·9 (46·5–76·3)	62·7 (48·0–75·5)	62·2 (49·9–77·3)
Intervals between first and second doses, days		68·0 (59·0–76·0)	69·0 (61·0–76·0)	68·0 (60·0–76·0)	70·0 (62·8–77·0)	41·0 (21·0–68·8)	34·0 (21·0–65·0)	42·0 (23·2–65·5)	56·0 (28·0–70·0)
Intervals between second and third doses, days		78·0 (72·0–86·0)	78·0 (73·0–84·5)	76·0 (72·0–84·5)	77·0 (71·0–85·0)	104·5 (95·2–146·0)	110·0 (92·0–148·0)	104·5 (93·0–146·8)	100·0 (91·8–134·8)
Age groups, years									
	<70	57 (52·3%)	59 (53·2%)	63 (54·8%)	59 (54·6%)	66 (55·9%)	64 (58·7%)	65 (57·0%)	67 (59·8%)
	≥70	52 (47·7%)	52 (46·8%)	52 (45·2%)	49 (45·4%)	52 (44·1%)	45 (41·3%)	49 (43·0%)	45 (40·2%)
Gender									
	Female	54 (49·5%)	54 (48·6%)	61 (53·0%)	58 (53·7%)	69 (58·5%)	57 (52·3%)	65 (57·0%)	67 (59·8%)
	Male	55 (50·5%)	57 (51·4%)	54 (47·0%)	50 (46·3%)	49 (41·5%)	52 (47·7%)	49 (43·0%)	45 (40·2%)
Occupation									
	Health worker	31 (28·4%)	31 (27·9%)	40 (34·8%)	40 (37·0%)	57 (48·3%)	53 (48·6%)	59 (51·8%)	53 (47·3%)
	Other	78 (71·6%)	80 (72·1%)	75 (65·2%)	68 (63·0%)	61 (51·7%)	56 (51·4%)	55 (48·2%)	59 (52·7%)
Ethnicity									
	White	107 (98·2%)	105 (94·6%)	107 (93·0%)	103 (95·4%)	106 (89·8%)	102 (93·6%)	109 (95·6%)	107 (95·5%)
	Black	0	0	1 (0·9%)	1 (0·9%)	1 (0·8%)	0	0	0
	Asian	2 (1·8%)	4 (3·6%)	3 (2·6%)	2 (1·9%)	10 (8·5%)	6 (5·5%)	5 (4·4%)	2 (1·8%)
	Mixed	0	2 (1·8%)	0	0	1 (0·8%)	1 (0·9%)	0	0
	Other	0	0	3 (2·6%)	2 (1·9%)	0	0	0	3 (2·7%)
	Not given	0	0	1 (0·9%)	0	0	0	0	0
Comorbidities									
	Cardiovascular	36 (33·0%)	36 (32·4%)	41 (35·7%)	35 (32·4%)	37 (31·4%)	31 (28·4%)	35 (30·7%)	35 (31·2%)
	Respiratory	19 (17·4%)	13 (11·7%)	15 (13·0%)	24 (22·2%)	14 (11·9%)	13 (11·9%)	15 (13·2%)	18 (16·1%)
	Diabetes	8 (7·3%)	12 (10·8%)	14 (12·2%)	11 (10·2%)	11 (9·3%)	7 (6·4%)	7 (6·1%)	12 (10·7%)

Data are median (IQR) or n (%), unless otherwise stated. There were three participants missing on occupation and five participants missing on ethnicity, which were not included in this table. ChAd=ChAdOx1 nCoV-19 vaccine, Oxford–AstraZeneca. BNT=BNT162b2 vaccine, Pfizer–BioNTech. Control=quadrivalent meningococcal conjugate vaccine. NVX=NVX-CoV2373 vaccine, Novavax. NVX half=half dose of NVX-CoV2373 vaccine.

**Table 3:** Baseline characteristics by third dose vaccine allocation and priming vaccine schedule, group B

		**Prime with ChAd/ChAd**					**Prime with BNT/BNT**				
		Control (n=106)	BNT (n=107)	VLA (n=109)	VLA half (n=111)	Ad26 (n=108)	Control (n=109)	BNT (n=110)	VLA (n=110)	VLA half (n=110)	Ad26 (n=106)
Age, years											
	Mean (SD)	66·0 (14·3)	65·1 (15·3)	64·4 (15·3)	64·0 (14·9)	65·0 (14·9)	62·9 (16·9)	62·6 (17·1)	60·9 (18·1)	62·4 (16·7)	62·0 (17·4)
	Median (IQR)	72·6 (57·6–77·2)	71·4 (53·8–77·0)	71·8 (51·2–76·5)	71·0 (51·2–75·9)	71·9 (51·0–76·4)	63·5 (50·4–78·3)	64·2 (49·8–77·4)	61·2 (46·2–77·7)	62·0 (51·8–76·2)	61·6 (49·2–78·3)
Intervals between first and second doses, days		68·5 (63·0–77·0)	73·0 (66·0–77·0)	70·0 (63·0–77·0)	72·0 (64·0–77·0)	74·5 (68·0–77·0)	64·0 (24·0–74·0)	65·0 (28·0–74·0)	64·5 (27·2–73·0)	63·5 (27·2–74·0)	62·0 (25·2–74·0)
Intervals between second and third doses, days		78·0 (75·0–84·0)	77·0 (73·0–84·8)	79·0 (73·0–85·0)	77·0 (73·0–84·0)	77·0 (72·0–83·0)	101·0 (89·0–147·0)	100·0 (91·0–135·0)	100·5 (91·0–146·8)	101·5 (90·2–141·5)	106·0 (91·0–143·8)
Age groups, years											
	<70	48 (45·3%)	50 (46·7%)	51 (46·8%)	51 (45·9%)	50 (46·3%)	62 (56·9%)	60 (54·5%)	63 (57·3%)	61 (55·5%)	59 (55·7%)
	≥70	58 (54·7%)	57 (53·3%)	58 (53·2%)	60 (54·1%)	58 (53·7%)	47 (43·1%)	50 (45·5%)	47 (42·7%)	49 (44·5%)	47 (44·3%)
Gender											
	Female	53 (50·0%)	50 (46·7%)	50 (45·9%)	54 (48·6%)	48 (44·4%)	52 (47·7%)	61 (55·5%)	59 (53·6%)	49 (44·5%)	60 (56·6%)
	Male	53 (50·0%)	57 (53·3%)	59 (54·1%)	57 (51·4%)	60 (55·6%)	57 (52·3%)	49 (44·5%)	51 (46·4%)	61 (55·5%)	46 (43·4%)
Occupation											
	Health worker	24 (22·6%)	28 (26·2%)	33 (30·3%)	32 (28·8%)	29 (26·9%)	54 (49·5%)	55 (50·0%)	51 (46·4%)	48 (43·6%)	55 (51·9%)
	Other	82 (77·4%)	79 (73·8%)	76 (69·7%)	78 (70·3%)	79 (73·1%)	55 (50·5%)	55 (50·0%)	59 (53·6%)	62 (56·4%)	51 (48·1%)
Ethnicity											
	White	103 (97·2%)	104 (97·2%)	100 (91·7%)	107 (96·4%)	100 (92·6%)	101 (92·7%)	105 (95·5%)	99 (90·0%)	102 (92·7%)	103 (97·2%)
	Black	0	0	2 (1·8%)	1 (0·9%)	0	0	1 (0·9%)	2 (1·8%)	0	0
	Asian	1 (0·9%)	3 (2·8%)	5 (4·6%)	2 (1·8%)	5 (4·6%)	4 (3·7%)	3 (2·7%)	7 (6·4%)	6 (5·5%)	2 (1·9%)
	Mixed	1 (0·9%)	0	0	0	0	2 (1·8%)	0	1 (0·9%)	2 (1·8%)	1 (0·9%)
	Other	1 (0·9%)	0	1 (0·9%)	0	2 (1·9%)	2 (1·8%)	1 (0·9%)	1 (0·9%)	0 (0·0%)	0
	Not given	0	0	1 (0·9%)	0	1 (0·9%)	0	0	0	0	0
Comorbidities											
	Cardiovascular	33 (31·4%)	39 (36·4%)	39 (35·8%)	30 (27·0%)	42 (38·9%)	25 (22·9%)	30 (27·3%)	29 (26·4%)	27 (24·5%)	30 (28·3%)
	Respiratory	18 (17·1%)	10 (9·3%)	15 (13·8%)	14 (12·6%)	20 (18·5%)	10 (9·2%)	12 (10·9%)	16 (14·5%)	13 (11·8%)	17 (16·0%)
	Diabetes	4 (3·8%)	7 (6·5%)	8 (7·3%)	6 (5·4%)	7 (6·5%)	5 (4·6%)	6 (5·5%)	5 (4·5%)	5 (4·5%)	4 (3·8%)

Data are median (IQR) or n (%), unless otherwise stated. There were three participants missing on occupation and five participants missing on ethnicity, which were not included in this table. ChAd=ChAdOx1 nCoV-19 vaccine, Oxford–AstraZeneca. Control=quadrivalent meningococcal conjugate vaccine. BNT=BNT162b2 vaccine, Pfizer–BioNTech. VLA=VLA2001 vaccine, Valneva. VLA half=half dose of VLA2001 vaccine. Ad26=Ad26.COV2.S vaccine, Janssen.

**Table 4:** Baseline characteristics by third dose vaccine allocation and priming vaccine schedule, group C

		**Prime with ChAd/ChAd**				**Prime with BNT/BNT**			
		Control (n=114)	BNT half (n=117)	m1273 (n=112)	CVn (n=119)	Control (n=112)	BNT half (n=110)	m1273 (n=111)	CVn (n=106)
Age, years									
	Mean (SD)	64·0 (13·2)	64·6 (13·6)	63·8 (14·1)	64·4 (13·5)	63·6 (16·3)	62·6 (17·3)	63·0 (15·3)	62·7 (16·4)
	Median (IQR)	70·3 (54·4–75·1)	71·0 (55·8–75·3)	70·2 (53·0–75·3)	70·3 (54·8–75·1)	66·8 (51·9–78·0)	64·4 (47·7–78·2)	65·0 (50·3–75·5)	63·4 (47·3–76·6)
Intervals between first and second doses, days		72·0 (65·0–77·0)	74·0 (66·0–77·0)	70·0 (63·0–77·0)	72·0 (65·0–77·0)	70·0 (45·0–76·0)	60·5 (22·2–74·8)	66·0 (23·0–76·0)	66·0 (23·0–73·8)
Intervals between second and third doses, days		77·5 (73·0–85·0)	78·0 (73·0–84·2)	79·0 (74·0–86·0)	78·0 (73·5–85·0)	93·5 (87·0–116·0)	107·5 (90·0–157·5)	101·5 (89·0–152·2)	98·0 (88·0–151·8)
Age groups, years									
	<70	54 (47·4%)	55 (47·0%)	55 (49·1%)	58 (48·7%)	60 (53·6%)	62 (56·4%)	62 (55·9%)	58 (54·7%)
	≥70	60 (52·6%)	62 (53·0%)	57 (50·9%)	61 (51·3%)	52 (46·4%)	48 (43·6%)	49 (44·1%)	48 (45·3%)
Gender									
	Female	47 (41·2%)	48 (41·0%)	45 (40·2%)	54 (45·4%)	60 (53·6%)	52 (47·3%)	63 (56·8%)	56 (52·8%)
	Male	67 (58·8%)	69 (59·0%)	67 (59·8%)	65 (54·6%)	52 (46·4%)	58 (52·7%)	48 (43·2%)	50 (47·2%)
Occupation									
	Health worker	29 (25·4%)	28 (23·9%)	26 (23·2%)	26 (21·8%)	48 (42·9%)	58 (52·7%)	59 (53·2%)	55 (51·9%)
	Other	85 (74·6%)	89 (76·1%)	85 (75·9%)	92 (77·3%)	64 (57·1%)	52 (47·3%)	52 (46·8%)	51 (48·1%)
Ethnicity									
	White	108 (94·7%)	115 (98·3%)	108 (96·4%)	113 (95·0%)	105 (92·9%)	93 (84·5%)	103 (92·8%)	87 (82·1%)
	Black	1 (0·9%)	0	0	0	0	2 (1·8%)	0	1 (0·9%)
	Asian	2 (1·8%)	1 (0·9%)	2 (1·8%)	5 (4·2%)	7 (6·2%)	9 (8·2%)	4 (3·6%)	14 (13·2%)
	Mixed	1 (0·9%)	0	1 (0·9%)	0	1 (0·9%)	3 (2·7%)	3 (2·7%)	2 (1·9%)
	Other	1 (0·9%)	0	0	0	0	2 (1·8%)	0	1 (0·9%)
	Not given	0	1 (0·9%)	0	0	0	1 (0·9%)	0	1 (0·9%)
Comorbidities									
	Cardiovascular	39 (34·2%)	35 (29·9%)	36 (32·1%)	36 (30·3%)	43 (38·4%)	35 (31·8%)	29 (26·1%)	35 (33·0%)
	Respiratory	14 (12·3%)	17 (14·5%)	12 (10·7%)	12 (10·1%)	12 (10·6%)	17 (15·5%)	17 (15·3%)	9 (8·5%)
	Diabetes	5 (4·4%)	4 (3·4%)	7 (6·2%)	11 (9·2%)	7 (6·2%)	11 (10·0%)	3 (2·7%)	8 (7·5%)

Data are median (IQR) or n (%), unless otherwise stated. There were three participants missing on occupation and five participants missing on ethnicity, which were not included in this table. ChAd=ChAdOx1 nCoV-19 vaccine, Oxford–AstraZeneca. Control=quadrivalent meningococcal conjugate vaccine. BNT=BNT162b2 vaccine, Pfizer–BioNTech. BNT half=half dose of BNT162b2 vaccine. m1273=mRNA1273 vaccine, Moderna. CVn=CVnCoV vaccine, Curevac.

The profiles of any grade local and systemic reactions within 7 days after all vaccines were similar, with fatigue and headache the most common systemic reactions, and pain the most frequent local reaction (appendix 1 pp 22–25). Overall, reactogenicity was greater in people aged 30–69 years compared with older participants regardless of the first vaccines received (appendix 1 pp 22–32). Participants primed with BNT/BNT reported more frequent local and systemic reactions after receiving m1273, CVn, ChAd, and Ad26 as a third dose, compared with other vaccines and control. Participants receiving mRNA vaccines or Ad26 after ChAd/ChAd also showed increased systemic and local adverse events. Among all mRNA vaccines, m1273 was the most reactogenic. Moderate or severe pain was reported to a similar degree in BNT and half BNT groups with some reduction in systemic adverse events, although these observations were in different groups. Ad26, m1273, and CVn recipients also reported feverishness frequently, which was not seen among BNT or half BNT participants.

**Figure 2:**
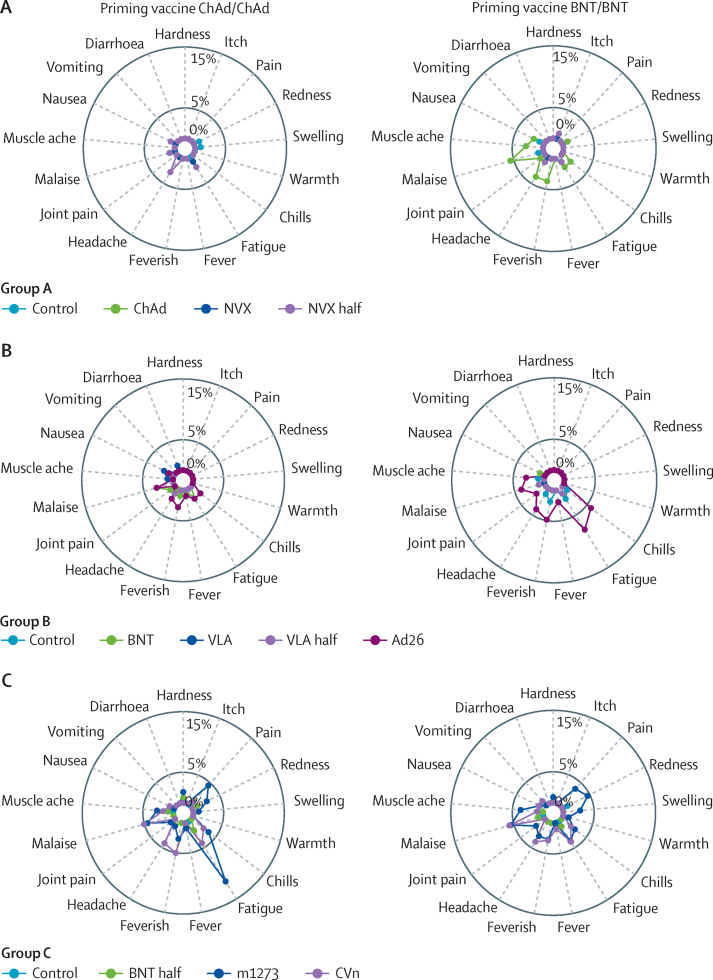
Radial graph for the occurrence of severe local and solicited adverse events in the first 7 days post vaccination in groups A, B, and C Control=quadrivalent meningococcal conjugate vaccine. ChAd=ChAdOx1 nCoV-19 vaccine, Oxford–AstraZeneca. NVX=NVX-CoV2373 vaccine, Novavax. NVX half=half dose of NVX-CoV2373 vaccine. BNT=BNT162b2 vaccine, Pfizer–BioNTech. VLA=VLA2001 vaccine, Valneva. VLA half=half dose of VLA2001 vaccine. Ad26=Ad26.COV2.S vaccine, Janssen. m1273=mRNA1273 vaccine, Moderna. CVn=CVnCoV vaccine, Curevac.

For ChAd/ChAd, the frequencies of severe local and systemic reactions were less than 5% for all vaccine groups except severe fatigue, which was reported in 13 (11·6%) of 112 m1273 recipients (figure 2). For the BNT/BNT group, malaise was reported in six (5·6%) of 108 ChAd recipients, six (5·5%) of 109 m1273 recipients, and six (5·8%) of 104 CVn recipients, whereas six (5·8%) of 103 participants boosted with Ad26 reported chills and eight (7·8%) of 103 reported fatigue. All the other severe reactions were reported in less than 5% of participants across all vaccine groups.

Between study enrolment and the data lock on Aug 19, 2021, there were 1306 adverse events reported from 912 participants (appendix 1 pp 2–10). 20 adverse events of special interest occurred after excluding SARS-CoV-2 infection in the 14 days immediately after third dose and serious adverse events, among which six were deemed as possibly related to the study vaccine (appendix 1 pp 11–12). 21 participants reported a PCR test result positive for SARS-CoV-2 with no hospitalisation (appendix 1 p 13). Description of the 24 serious adverse events including four suspected unexpected serious adverse reactions are listed in appendix 1 (pp 14–16).

**Figure 3:**
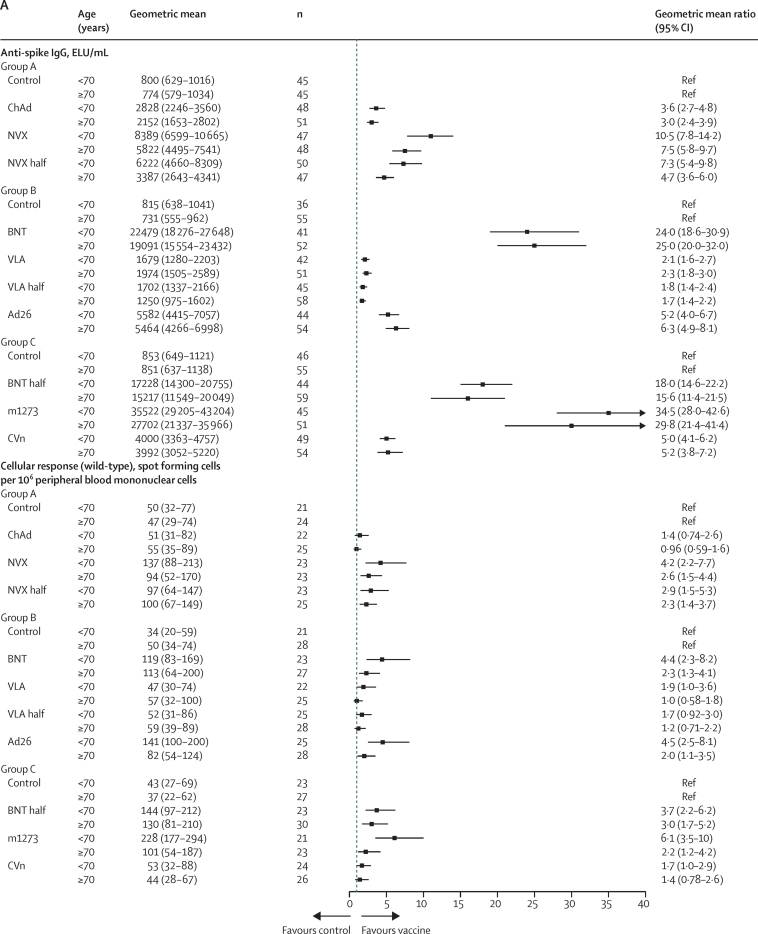

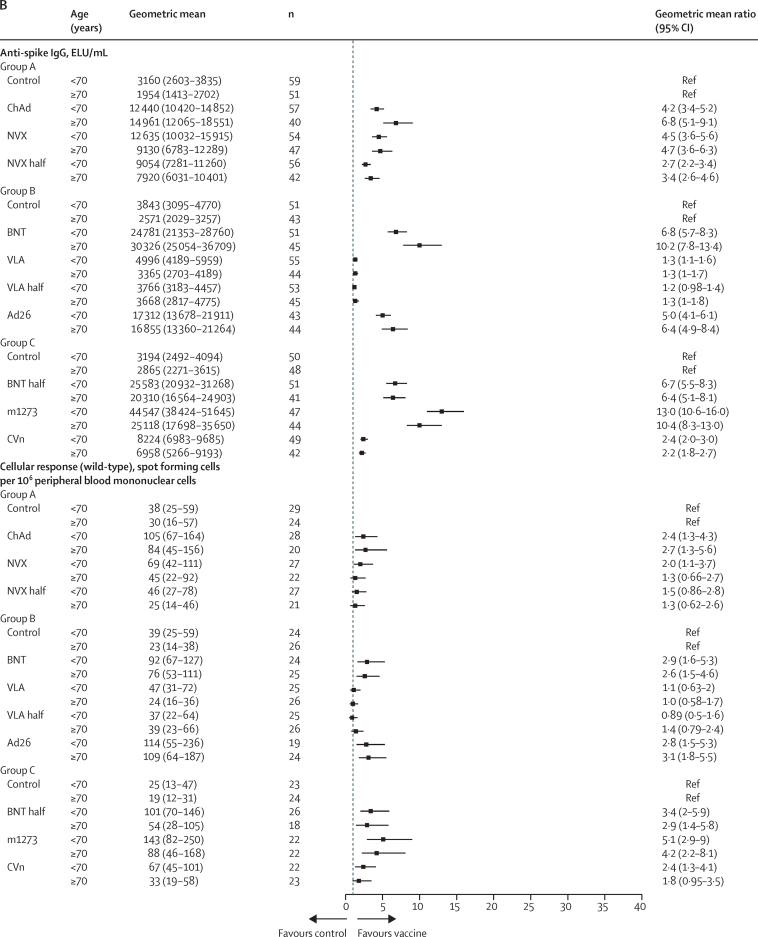
Subgroup immunogenicity analyses by age for anti-spike IgG and cellular response at 28 days post third dose between study vaccines and controls for the ChAd/ChAd-primed population (A) and BNT/BNT-primed population (B) ELU=ELISA laboratory units. Control=quadrivalent meningococcal conjugate vaccine. ChAd=ChAdOx1 nCoV-19 vaccine, Oxford–AstraZeneca. NVX=NVX-CoV2373 vaccine, Novavax. NVX half=half dose of NVX-CoV2373 vaccine. BNT=BNT162b2 vaccine, Pfizer–BioNTech. VLA=VLA2001 vaccine, Valneva. VLA half=half dose of VLA2001 vaccine. Ad26=Ad26.COV2.S vaccine, Janssen. BNT half=half dose of BNT162b2 vaccine. m1273=mRNA1273 vaccine, Moderna. CVn=CVnCoV vaccine, Curevac.

Among participants primed with ChAd/ChAd, all COVID-19 vaccines given as the third dose induced significantly higher anti-spike IgG at 28 days post boost, compared with their corresponding controls (Table 5, Table 6, Table 7; figure 3). The GMRs between study vaccines and controls ranged from 1·8 (99% CI 1·5–2·3) in the half VLA group to 32·3 (24·8–42·0) in the m1273 group. GMRs for pseudotype virus neutralising antibodies against wild-type were consistent with those of anti-spike IgG. All the study vaccines except ChAd, VLA, and half VLA, given as the third dose, significantly induced cellular responses by T-cell ELISpot in ChAd/ChAd-primed participants. The GMRs compared with control groups ranged from 1·1 (95% CI 0·7–1·6) in the ChAd group to 3·6 (2·4–5·5) in the m1273 group.

**Table 5:** Immune responses by third dose vaccine allocation and priming vaccine schedule at 28 days post boost dose among the COVID-19-naive modified intention-to-treat population, group A

	**Prime with ChAd/ChAd**				**Prime with BNT/BNT**			
	Control (n=93)	ChAd (n=100)	NVX (n=96)	NVX half (n=97)	Control (n=111)	ChAd (n=98)	NVX (n=103)	NVX half (n=99)
**SARS-CoV-2 anti-spike IgG, ELU/mL**								
GMC*	801 (664–967; n=91)	2457 (2058–2933; n=99)	6975 (5829–8347; n=95)	4634 (3794–5660; n=97)	2541 (2110–3060; n=111)	13 424 (11 702–15 399; n=97)	10 862 (9009–13 097; n=101)	8550 (7210–10 138; n=98)
GMR†	Ref	3·25 (2·52–4·20)	8·75 (6·77–11·31)	5·82 (4·50–7·51)	Ref	5·33 (4·23–6·73)	4·78 (3·80–6·02)	3·07 (2·43–3·88)
**Pseudotype virus neutralising antibody (wild-type), NT**_50_								
GMT*	84·9 (68·7–105·0; n=90)	193 (161–231; n=98)	727 (598–883; n=87)	470 (378–583; n=86)	157 (129–192; n=111)	950 (802–1126; n=98)	766 (624–939; n=94)	606 (495–743; n=89)
GMR†	Ref	2·47 (1·96–3·11)	8·86 (7·00–11·22)	5·89 (4·64–7·46)	Ref	6·01 (4·87–7·41)	5·39 (4·35–6·67)	3·50 (2·81–4·36)
**Pseudotype virus neutralising antibody (delta), NT**_50_								
GMT*	20·0 (15·6–25·7; n=91)	48·9 (39·7–60·2; n=99)	124 (99–156; n=84)	87·2 (68·5–111; n=83)	37·9 (30·5–47·1; n=111)	260 (217–313; n=98)	165 (131–209; n=89)	131 (106–163; n=88)
GMR†	Ref	2·58 (1·92–3·47)	6·25 (4·60–8·50)	4·40 (3·23–6·00)	Ref	6·84 (5·39–8·68)	4·94 (3·86–6·31)	3·27 (2·55–4·20)
**Live virus neutralising antibody, normalised NT**_80_								
GMT*	146 (111–191; n=32)	346 (263–454; n=31)	837 (536–1307; n=18)	713 (490–1038; n=20)	531 (377–748; n=38)	2614 (2075–3294; n=40)	1454 (1060–1995; n=24)	1792 (1261–2547; n=21)
GMR†	Ref	2·57 (1·86–3·56)	6·29 (4·22–9·37)	5·30 (3·59–7·80)	Ref	5·01 (3·59–7·01)	2·65 (1·77–3·98)	2·81 (1·85–4·26)
**Cellular response (wild-type), spot forming cells per 10**^6^**peripheral blood mononuclear cells**								
GM*	48·1 (35·0–66·3; n=45)	53·0 (37·9–74·2; n=47)	113·7 (78·7–164·2; n=46)	98·4 (73·9–131·1; n=48)	34·5 (23·8–50·0; n=53)	95·8 (66·6–137·7; n=48)	56·6 (37·2–86·2; n=49)	35·3 (23·7–52·7; n=48)
GMR‡	Ref	1·08 (0·74–1·57)	3·23 (2·20–4·76)	2·43 (1·66–3·56)	Ref	2·55 (1·64–3·96)	1·79 (1·15–2·77)	1·40 (0·89–2·18)
**Cellular response (delta), spot forming cells per 10**^6^**peripheral blood mononuclear cells**								
GM*	38·1 (27·0–54·0; n=45)	44·9 (30·6–65·7; n=47)	117·9 (85·5–162·7; n=46)	86·3 (64·8–114·9; n=48)	35·7 (25·1–50·9; n=53)	108·0 (78·7–148·2; n=48)	56·9 (37·9–85·4; n=49)	41·6 (28·7–60·4; n=48)
GMR‡	Ref	1·13 (0·76–1·68)	4·26 (2·84–6·39)	2·71 (1·81–4·05)	Ref	2·74 (1·85–4·05)	1·71 (1·16–2·53)	1·56 (1·05–2·33)
**Cellular response (beta), spot forming cells per 10**^6^**peripheral blood mononuclear cells**								
GM*	50·0 (36·1–69·0; n=45)	53·0 (38·0–73·8; n=47)	117·0 (82·8–165·4; n=46)	91·1 (68·7–120·9; n=48)	32·4 (22·4–46·9; n=53)	101·2 (69·9–146·4; n=48)	51·2 (34·7–75·4; n=49)	37·2 (25·7–53·9; n=48)
GMR‡	Ref	1·03 (0·71–1·51)	3·26 (2·21–4·81)	2·20 (1·50–3·23)	Ref	2·97 (1·95–4·51)	1·78 (1·18–2·71)	1·65 (1·08–2·52)

ChAd=ChAdOx1 nCoV-19 vaccine, Oxford–AstraZeneca. BNT=BNT162b2 vaccine, Pfizer–BioNTech. Control=quadrivalent meningococcal conjugate vaccine. NVX=NVX-CoV2373 vaccine, Novavax. NVX half=half dose of NVX-CoV2373 vaccine. ELU=ELISA laboratory units. GMC=geometric mean concentration. GMR=geometric mean ratio. GM=geometric mean. GMT=geometric mean titre. NT_50_=50% neutralising antibody titre. NT_80_=80% neutralising antibody titre.

* Data are GM (95% CI; number of samples available).

† GMRs of the study vaccines were calculated by comparing to their corresponding controls in group A, B, or C, after adjusting for age group, site, baseline anti-spike IgG, interval between first and second dose, and interval between second and third dose; for primary endpoint of anti-spike IgG, 99% CIs were presented to account for multiple comparisons; for the secondary endpoints, 95% CIs were presented.

‡ GMRs of the study vaccines were calculated by comparing to their corresponding controls in group A, B, or C, after adjusting for age group, site, baseline cellular response against wild-type, interval between first and second dose, and interval between second and third dose; 95% CIs were presented.

**Table 6:** Immune responses by third dose vaccine allocation and priming vaccine schedule at 28 days post boost dose among the COVID-19-naive modified intention-to-treat population, group B

	**Prime with ChAd/ChAd**					**Prime with BNT/BNT**				
	Control (n=93)	BNT (n=95)	VLA (n=95)	VLA half (n=107)	Ad26 (n=101)	Control (n=97)	BNT (n=96)	VLA (n=99)	VLA half (n=98)	Ad26 (n=89)
**SARS-CoV-2 anti-spike IgG, ELU/mL**										
GMC*	763 (630–924; n=91)	20 517 (17 718–23 757; n=93)	1835 (1514–2224; n=93)	1430 (1198–1707; n=103)	5517 (4647–6548; n=98)	3197 (2714–3767; n=94)	27 242 (24 148–30 731; n=96)	4204 (3640–4856; n=98)	3721 (3200–4326; n=98)	17 079 (14 488–20 133; n=87)
GMR†	Ref	24·48 (19·50–30·79)	2·20 (1·75–2·77)	1·81 (1·45–2·27)	5·84 (4·65–7·33)	Ref	8·11 (6·59–9·99)	1·31 (1·07–1·62)	1·25 (1·01–1·54)	5·63 (4·55–6·97)
**Pseudotype virus neutralising antibody (wild-type), NT**_50_										
GMT*	69·6 (57·2–84·6; n=91)	1621 (1314–1998; n=93)	202 (166–247; n=89)	147 (124–174; n=95)	563 (454–698; n=95)	205 (167–253; n=93)	1789 (1520–2107; n=95)	289 (244–342; n=91)	234 (200–272; n=87)	1441 (1188–1749; n=75)
GMR†	Ref	21·58 (16·93–27·51)	2·68 (2·10–3·43)	2·01 (1·57–2·55)	6·85 (5·37–8·73)	Ref	8·35 (6·88–10·14)	1·38 (1·14–1·68)	1·22 (1·00–1·49)	7·84 (6·37–9·64)
**Pseudotype virus neutralising antibody (delta), NT**_50_										
GMT*	20·4 (16·4–25·5; n=91)	315 (254–391; n=93)	35·2 (28·4–43·7; n=89)	31·1 (25·6–37·7; n=95)	125 (99–159; n=90)	56·5 (43·6–73·3; n=92)	392 (320–479; n=95)	67·1 (55·4–81·2; n=94)	54·7 (45·1–66·4; n=92)	418 (330–530; n=78)
GMR†	Ref	14·43 (10·97–18·98)	1·65 (1·25–2·17)	1·50 (1·14–1·96)	5·33 (4·04–7·03)	Ref	6·60 (5·10–8·53)	1·19 (0·92–1·54)	1·02 (0·79–1·32)	8·02 (6·12–10·50)
**Live virus neutralising antibody, normalised NT**_80_										
GMT*	174 (139–218; n=30)	4899 (3955–6069; n=38)	354 (215–584; n=21)	301 (212–427; n=25)	1053 (691–1605; n=23)	756 (568–1007; n=34)	4603 (3685–5749; n=36)	836 (580–1207; n=20)	555 (407–756; n=23)	3535 (2459–5080; n=19)
GMR†	Ref	25·61 (18·07–36·31)	2·04 (1·37–3·05)	1·81 (1·23–2·65)	5·97 (4·03–8·84)	Ref	5·79 (4·25–7·90)	1·42 (0·98–2·06)	0·93 (0·65–1·33)	5·36 (3·67–7·83)
**Cellular response (wild-type), spot forming cells per 10**^6^**peripheral blood mononuclear cells**										
GM*	42·6 (30·9–58·8; n=49)	115·5 (81·7–163·3; n=50)	52·2 (36·3–75·0; n=47)	55·5 (40·4–76·3; n=53)	106·0 (80·1–140·4; n=53)	29·4 (21·0–41·2; n=50)	83·8 (65·4–107·2; n=49)	33·5 (24·7–45·4; n=51)	38·1 (26·1–55·5; n=51)	111·0 (71·8–171·6; n=43)
GMR‡	Ref	3·15 (2·08–4·76)	1·39 (0·92–2·11)	1·40 (0·93–2·11)	2·74 (1·82–4·12)	Ref	2·65 (1·78–3·95)	1·04 (0·69–1·55)	1·12 (0·75–1·66)	2·93 (1·93–4·44)
**Cellular response (delta), spot forming cells per 10**^6^**peripheral blood mononuclear cells**										
GM*	42·2 (30·5–58·3; n=49)	123·2 (93·0–163·3; n=50)	52·8 (36·9–75·6; n=47)	54·7 (41·5–72·0; n=53)	102·1 (74·4–140·2; n=53)	28·2 (19·9–39·9; n=50)	82·1 (65·7–102·7; n=49)	29·6 (20·9–42·0; n=51)	39·2 (27·2–56·6; n=51)	121·5 (78·9–187·0; n=43)
GMR‡	Ref	3·23 (2·15–4·86)	1·40 (0·93–2·12)	1·39 (0·93–2·08)	2·67 (1·79–4·00)	Ref	2·71 (1·78–4·13)	0·96 (0·63–1·47)	1·22 (0·80–1·85)	3·29 (2·12–5·11)
**Cellular response (beta), spot forming cells per 10**^6^**peripheral blood mononuclear cells**										
GM*	47·6 (35·2–64·4; n=49)	120·5 (88·0–165·0; n=50)	52·6 (36·3–76·3; n=47)	56·8 (41·0–78·7; n=53)	99·9 (72·6–137·6; n=53)	27·6 (19·9–38·5; n=50)	85·2 (69·8–103·9; n=49)	31·1 (22·5–42·9; n=51)	40·3 (28·1–57·7; n=51)	118·6 (78·3–179·7; n=43)
GMR‡	Ref	2·88 (1·89–4·38)	1·25 (0·82–1·90)	1·28 (0·85–1·94)	2·30 (1·52–3·48)	Ref	2·86 (1·92–4·28)	1·05 (0·70–1·56)	1·27 (0·85–1·89)	3·36 (2·21–5·10)

ChAd=ChAdOx1 nCoV-19 vaccine, Oxford–AstraZeneca. Control=quadrivalent meningococcal conjugate vaccine. BNT=BNT162b2 vaccine, Pfizer–BioNTech. VLA=VLA2001 vaccine, Valneva. VLA half=half dose of VLA2001 vaccine. Ad26=Ad26.COV2.S vaccine, Janssen. ELU=ELISA laboratory units. GMC=geometric mean concentration. GMR=geometric mean ratio. GM=geometric mean. GMT=geometric mean titre. NT_50_=50% neutralising antibody titre. NT_80_=80% neutralising antibody titre.

* Data are GM (95% CI; number of samples available).

† GMRs of the study vaccines were calculated by comparing to their corresponding controls in group A, B, or C, after adjusting for age group, site, baseline anti-spike IgG, interval between first and second dose, and interval between second and third dose; for primary endpoint of anti-spike IgG, 99% CIs were presented to account for multiple comparisons; for the secondary endpoints, 95% CIs were presented.

‡ GMRs of the study vaccines were calculated by comparing to their corresponding controls in group A, B, or C, after adjusting for age group, site, baseline cellular response against wild-type, interval between first and second dose, and interval between second and third dose; 95% CIs were presented.

**Table 7:** Immune responses by third dose vaccine allocation and priming vaccine schedule at 28 days post boost dose among the COVID-19-naive modified intention-to-treat population, group C

	**Prime with ChAd/ChAd**				**Prime with BNT/BNT**			
	Control (n=102)	BNT half (n=105)	m1273 (n=98)	CVn (n=105)	Control (n=100)	BNT half (n=94)	m1273 (n=92)	CVn (n=94)
**SARS-CoV-2 anti-spike IgG, ELU/mL**								
GMC*	852 (697–1041; n=101)	16 045 (13 449–19 143; n=103)	31 111 (26 363–36 714; n=97)	3996 (3397–4700; n=103)	3029 (2556–3589; n=98)	23 082 (19 971–26 678; n=92)	33 768 (27 816–40 993; n=91)	7613 (6515–8897; n=91)
GMR†	Ref	16·80 (12·97–21·76)	32·30 (24·84–42·01)	5·05 (3·90–6·54)	Ref	6·78 (5·51–8·35)	11·49 (9·36–14·12)	2·30 (1·87–2·83)
**Pseudotype virus neutralising antibody (wild-type), NT**_50_								
GMT*	80·4 (65·6–98·5; n=101)	1344 (1131–1596; n=103)	2368 (2054–2730; n=97)	373 (310–448; n=99)	175 (144–212; n=98)	1339 (1123–1596; n=92)	2019 (1621–2513; n=91)	487 (411–577; n=91)
GMR†	Ref	15·14 (12·32–18·60)	26·98 (21·88–33·26)	5·06 (4·11–6·23)	Ref	6·91 (5·70–8·37)	12·04 (9·95–14·58)	2·57 (2·13–3·12)
**Pseudotype virus neutralising antibody (delta), NT**_50_								
GMT*	18·6 (14·7–23·5; n=101)	321·3 (262·4–393·5; n=103)	559·7 (441·3–709·9; n=96)	64·5 (54·2–76·7; n=93)	41·6 (33·7–51·4; n=98)	352·6 (286·7–433·6; n=91)	508·7 (408·6–633·4; n=91)	119·1 (96·1–147·5; n=89)
GMR†	Ref	15·71 (12·09–20·41)	27·17 (20·81–35·47)	3·76 (2·87–4·91)	Ref	7·39 (5·88–9·29)	12·58 (10·03–15·77)	2·59 (2·07–3·26)
**Live virus neutralising antibody, normalised NT**_80_								
GMT*	152 (106–218; n=38)	2501 (1978–3163; n=40)	5421 (4248–6918; n=24)	774 (485–1235; n=20)	469 (332–664; n=37)	3263 (2601–4093; n=37)	5354 (4195–6833; n=23)	1960 (1199–3205; n=18)
GMR†	Ref	12·93 (9·51–17·57)	28·26 (19·66–40·63)	5·00 (3·42–7·32)	Ref	5·18 (3·72–7·21)	9·32 (6·37–13·66)	3·26 (2·15–4·95)
**Cellular response (wild-type), spot forming cells per 10**^6^**peripheral blood mononuclear cells**								
GM*	39·5 (27·8–56·2; n=50)	135·9 (99·1–186·2; n=53)	148·9 (103·6–213·9; n=44)	47·8 (34·4–66·3; n=50)	22·0 (14·9–32·4; n=47)	78·4 (55·1–111·5; n=44)	112·0 (72·8–172·3; n=44)	46·7 (32·5–67; n=45)
GMR‡	Ref	3·31 (2·22–4·93)	3·59 (2·36–5·45)	1·51 (1·01–2·27)	Ref	3·01 (1·98–4·57)	4·66 (3·07–7·08)	2·10 (1·38–3·18)
**Cellular response (delta), spot forming cells per 10**^6^**peripheral blood mononuclear cells**								
GM*	35·2 (24·6–50·4; n=50)	139·1 (104·1–185·9; n=53)	152·1 (109·3–211·7; n=44)	45·5 (33·0–62·8; n=50)	25·9 (17·6–38·1; n=47)	93·0 (68·0–127·1; n=44)	118·3 (79·8–175·4; n=44)	52·2 (37·0–73·6; n=45)
GMR‡	Ref	3·91 (2·62–5·83)	4·25 (2·79–6·47)	1·67 (1·11–2·51)	Ref	3·04 (2·01–4·58)	4·14 (2·75–6·23)	1·90 (1·27–2·86)
**Cellular response (beta), spot forming cells per 10**^6^**peripheral blood mononuclear cells**								
GM*	41·1 (28·3–59·7; n=50)	127·5 (91·4–178·0; n=53)	128·9 (85·2–195·2; n=44)	38·3 (26·0–56·4; n=50)	25·8 (17·6–37·7; n=47)	80·9 (56·0–116·9; n=44)	102·4 (64·7–162·0; n=44)	42·2 (28·9–61·5; n=45)
GMR‡	Ref	2·94 (1·82–4·76)	3·10 (1·87–5·14)	1·17 (0·71–1·91)	Ref	2·62 (1·65–4·17)	3·57 (2·25–5·67)	1·61 (1·02–2·55)

ChAd=ChAdOx1 nCoV-19 vaccine, Oxford–AstraZeneca. Control=quadrivalent meningococcal conjugate vaccine. BNT=BNT162b2 vaccine, Pfizer–BioNTech. BNT half=half dose of BNT162b2 vaccine. m1273=mRNA1273 vaccine, Moderna. CVn=CVnCoV vaccine, Curevac. ELU=ELISA laboratory units. GMC=geometric mean concentration. GMR=geometric mean ratio. GM=geometric mean. GMT=geometric mean titre. NT_50_=50% neutralising antibody titre. NT_80_=80% neutralising antibody titre.

* Data are GM (95% CI; number of samples available).

† GMRs of the study vaccines were calculated by comparing to their corresponding controls in group A, B, or C, after adjusting for age group, site, baseline anti-spike IgG, interval between first and second dose, and interval between second and third dose; for primary endpoint of anti-spike IgG, 99% CIs were presented to account for multiple comparisons; for the secondary endpoints, 95% CIs were presented.

‡ GMRs of the study vaccines were calculated by comparing to their corresponding controls in group A, B, or C, after adjusting for age group, site, baseline cellular response against wild-type, interval between first and second dose, and interval between second and third dose; 95% CIs were presented.

In participants who received BNT/BNT as the initial schedule, significant GMRs were also observed in all study vaccine groups compared with controls for anti-spike IgG at 28 days post boost, ranging from 1·3 (99% CI 1·0–1·5) in the half VLA group to 11·5 (9·4–14·1) in the m1273 group (Table 5, Table 6, Table 7; figure 3). However, the upper limit of the 99% CI for VLA and half VLA did not reach the pre-established minimum clinically important difference of 1·75. Similarly, GMRs for pseudotype virus neutralising antibodies and anti-spike IgG antibodies were also consistent in the BNT/BNT participants. For the cellular responses, we observed a significant GMR for ChAd (2·6, 95% CI 1·6–4·0) following BNT/BNT, which was not seen for ChAd following ChAd/ChAd (1·1, 0·7–1·6). The T-cell-boosting effects of NVX and half NVX were lower in people who had received BNT/BNT compared with those who previously received ChAd/ChAd. The geometric mean of T-cell responses in the half NVX group was not significantly higher than control (1·4, 95% CI 0·89–2·2). All the other vaccines showed higher cellular responses compared with controls among participants who had previously received BNT/BNT.

The kinetics of binding antibodies are presented in Table 8, Table 9, Table 10 and appendix 1 (p 33), and the fold-rise of immunogencity between days 0 and 28 is presented in appendix 1 (pp 17–20). We observed a higher level of baseline anti-spike IgG concentrations in participants who had received BNT/BNT compared with participants who had received ChAd/ChAd. The GMCs at baseline were 3921 (95% CI 3721–4132; n=1266) in the participants primed with BNT/BNT and 1166 (1105–1230; n=1279) in the participants primed with ChAd/ChAd. An increase in anti-spike IgG concentrations at day 7 compared with baseline was observed in all study vaccine groups, except VLA and half VLA. BNT, half BNT, and m1273 concentrations did not rise further from day 7 to day 28 irrespective of the initial vaccines received (Table 8, Table 9, Table 10; appendix 1 p 33). Excepting people who received ChAd after ChAd/ChAd, we observed a further increase from day 7 to day 28 in people who received ChAd, NVX, half NVX, Ad26, and CVn following ChAd/ChAd and BNT/BNT. All vaccines that boosted cellular responses showed a peak at day 14 (Table 8, Table 9, Table 10; appendix 1 p 33).

**Table 8:** Kinetics of immune responses post third dose by study vaccine and priming vaccine schedule among the modified intention-to-treat population of immunology cohort, group A

	**Prime with ChAd/ChAd**				**Prime with BNT/BNT**			
	Control (n=18)	ChAd (n=16)	NVX (n=19)	NVX half (n=21)	Control (n=26)	ChAd (n=24)	NVX (n=24)	NVX half (n=21)
**SARS-CoV-2 anti-spike IgG, ELU/mL**								
Day 0	1237 (835–1833; n=18)	786 (593–1041; n=16)	1053 (610–1818; n=19)	1073 (702–1641; n=21)	3482 (2482–4886; n=26)	3196 (2142–4769; n=24)	3512 (2454–5026; n=24)	4469 (2836–7043; n=21)
Day 7	1177 (750–1849; n=14)	1242 (942–1637; n=15)	2935 (1932–4457; n=18)	3543 (2521–4979; n=20)	3124 (2216–4404; n=25)	8624 (6664–11 160; n=24)	5080 (3585–7199; n=23)	4881 (3207–7428; n=20)
Day 28	841 (538–1313; n=16)	1321 (995–1752; n=15)	4791 (3390–6769; n=18)	4959 (3413–7206; n=21)	2415 (1751–3330; n=26)	13 708 (10 368–18 125; n=24)	8754 (6262–12236; n=24)	10 171 (6892–15 010; n=21)
**Cellular response (wild-type), spot forming cells per 10**^6^**peripheral blood mononuclear cells**								
Day 0	52·8 (34·4–81·0; n=18)	61·7 (37·2–102·4; n=16)	25·3 (13·9–46·0; n=19)	37·1 (24·2–56·8; n=20)	37·9 (23·8–60·5; n=25)	57·9 (32·1–104·3; n=23)	56·9 (35·2–91·9; n=23)	25·8 (14·7–45·1; n=20)
Day 14	48·7 (24·1–98·3; n=14)	91·1 (48·7–170·6; n=13)	239·6 (156·0–368·0; n=18)	133·0 (76·8–230·4; n=20)	33·7 (20·2–56·1; n=25)	134·9 (75·9–239·9; n=24)	85·8 (52·4–140·7; n=23)	69·5 (40·9–118·1; n=20)
Day 28	56·1 (30·6–103·1; n=16)	72·4 (43·8–119·5; n=15)	104·5 (59·2–184·3; n=17)	171·5 (126·7–232·0; n=19)	33·2 (20·8–53·0; n=25)	116·4 (67·8–199·7; n=23)	75·0 (40·6–138·4; n=23)	39·8 (19·3–82·3; n=20)

Data are geometric mean (95% CI; number of samples available). ChAd=ChAdOx1 nCoV-19 vaccine, Oxford–AstraZeneca. BNT=BNT162b2 vaccine, Pfizer–BioNTech. Control=quadrivalent meningococcal conjugate vaccine. NVX=NVX-CoV2373 vaccine, Novavax. NVX half=half dose of NVX-CoV2373 vaccine. ELU=ELISA laboratory units.

**Table 9:** Kinetics of immune responses post third dose by study vaccine and priming vaccine schedule among the modified intention-to-treat population of immunology cohort, group B

	**Prime with ChAd/ChAd**					**Prime with BNT/BNT**				
	Control (n=23)	BNT (n=23)	VLA (n=23)	VLA half (n=27)	Ad26 (n=24)	Control (n=23)	BNT (n=23)	VLA (n=20)	VLA half (n=24)	Ad26 (n=19)
**SARS-CoV-2 anti-spike IgG, ELU/mL**										
Day 0	1276 (945–1723; n=23)	1443 (1051–1982; n=23)	1211 (744–1971; n=23)	1334 (899–1981; n=27)	1555 (1037–2331; n=24)	4483 (3153–6374; n=23)	5422 (3781–7776; n=23)	3352 (1814–6194; n=20)	3460 (2322–5156; n=24)	4181 (3037–5756; n=19)
Day 7	1080 (808–1443; n=23)	15 524 (10 938–22 033; n=23)	1299 (829–2036; n=23)	1292 (912–1830; n=27)	2735 (2023–3697; n=24)	3852 (2797–5303; n=23)	27 551 (21 016–36 118; n=23)	3829 (2620–5595; n=20)	3415 (2506–4653; n=24)	7726 (5592–10 675; n=19)
Day 28	867 (634–1186; n=23	21 824 (16 938–28 119; n=23)	1599 (988–2589; n=23)	1537 (1054–2243; n=27)	5673 (4078–7892; n=24)	3209 (2338–4404; n=23)	26 171 (21 245–32 239; n=23)	4428 (3264–6008; n=20)	3500 (2599–4714; n=24)	18 631 (11 767–29 499; n=19)
**Cellular response (wild-type), spot forming cells per 10**^6^**peripheral blood mononuclear cells**										
Day 0	56·6 (33·4–95·9; n=23)	44·2 (25·3–77·4; n=21)	31·7 (17·3–58·2; n=23)	35·9 (22·4–57·4; n=27)	36·7 (21·8–61·9; n=24)	36·6 (22·9–58·6; n=22)	32·9 (19·5–55·3; n=23)	21·6 (11·1–42·0; n=19)	31·5 (18·3–54·3; n=24)	42·1 (22·4–79·0; n=18)
Day 14	73·6 (44·1–123·0; n=22)	131·0 (81·7–210·1; n=23)	72·2 (45·9–113·7; n=23)	49·8 (31·1–79·7; n=25)	123·4 (70·2–217·0; n=22)	38·2 (25·8–56·7; n=22)	94·6 (59·6–150·2; n=22)	24·3 (13·8–42·8; n=19)	34·9 (21·0–58·0; n=24)	114·1 (66·7–195·4; n=19)
Day 28	50·0 (30·3–82·5; n=23)	129·4 (76·4–219·2; n=23)	54·2 (32·3–91·2; n=22)	64·4 (44·6–93·0; n=27)	102·7 (64·3–164·2; n=24)	35·7 (19·2–66·2; n=21)	88·6 (65·7–119·4; n=22)	34·5 (23·3–51·0; n=20)	39·1 (22·5–68·1; n=24)	153·2 (72·3–324·6; n=19)

Data are geometric mean (95% CI; number of samples available). ChAd=ChAdOx1 nCoV-19 vaccine, Oxford–AstraZeneca. BNT=BNT162b2 vaccine, Pfizer–BioNTech. Control=quadrivalent meningococcal conjugate vaccine. VLA=VLA2001 vaccine, Valneva. VLA half=half dose of VLA2001 vaccine. Ad26=Ad26.COV2.S vaccine, Janssen. ELU=ELISA laboratory units.

**Table 10:** Kinetics of immune responses post third dose by study vaccine and priming vaccine schedule among the modified intention-to-treat population of immunology cohort, group C

	**Prime with ChAd/ChAd**				**Prime with BNT/BNT**			
	Control (n=21)	BNT half (n=25)	m1273 (n=21)	CVn (n=21)	Control (n=21)	BNT half (n=21)	m1273 (n=18)	CVn (n=18)
**SARS-CoV-2 anti-spike IgG, ELU/mL**								
Day 0	712 (466–1086; n=20)	1485 (994–2218; n=25)	1265 (907–1766; n=21)	920 (570–1486; n=21)	2761 (1759–4334; n=21)	4060 (2505–6582; n=21)	3271 (1970–5432; n=18)	4175 (2914–5982; n=18)
Day 7	671 (439–1027; n=21)	13 078 (8708–19 641; n=25)	22 134 (15 902–30 809; n=21)	2466 (1609–3780; n=20)	2403 (1493–3869; n=21)	24 315 (17 943–32 950; n=21)	20 930 (11 594–37 786; n=18)	6756 (4881–9351; n=18)
Day 28	600 (376–957; n=21)	13 951 (8978–21 679; n=25)	23 771 (15 092–37 442; n=21)	4241 (2718–6618; n=21)	2094 (1306–3359; n=20)	27 498 (20 109–37 602; n=21)	30 654 (22 916–41 004; n=18)	8385 (5753–12 222; n=18)
**Cellular response (wild-type), spot forming cells per 10**^6^**peripheral blood mononuclear cells**								
Day 0	45·1 (27·3–74·8; n=21)	47·1 (30·1–73·6; n=25)	48·4 (29·3–80·0; n=19)	47·6 (27·7–81·7; n=20)	38·3 (24·0–61·2; n=21)	42·0 (25·1–70·2; n=20)	28·3 (16·6–48·5; n=18)	56·6 (35·8–89·5; n=17)
Day 14	30·5 (16·1–57·8; n=21)	154·7 (99·9–239·6; n=25)	140·7 (75·0–263·8; n=19)	40·1 (19·4–82·8; n=20)	23·1 (13·3–40·2; n=21)	96·3 (53·2–174·2; n=20)	117·4 (68·7–200·5; n=18)	74·2 (37·6–146·3; n=17)
Day 28	48·8 (30·4–78·3; n=21)	123·5 (79·4–192·3; n=25)	148·6 (92·8–237·9; n=20)	65·1 (36·3–116·7; n=21)	26·9 (15·6–46·5; n=20)	107·0 (73·4–156·1; n=20)	140·4 (85·3–231·1; n=17)	68·8 (36·2–131·0; n=18)

Data are geometric mean (95% CI; number of samples available). ChAd=ChAdOx1 nCoV-19 vaccine, Oxford–AstraZeneca. BNT=BNT162b2 vaccine, Pfizer–BioNTech. Control=quadrivalent meningococcal conjugate vaccine. BNT half=half dose of BNT162b2 vaccine. m1273=mRNA1273 vaccine, Moderna. CVn=CVnCoV vaccine, Curevac. ELU=ELISA laboratory units.

Pseudoneutralising antibodies (NT_50_) were reduced for delta variant, relative to wild-type, across all vaccines after ChAd/ChAd and BNT/BNT (table 5–7; appendix 1 pp 17–20). Although there was minor variation in the degree of drop in the GMT, no vaccine showed better cross-protective immunity than others (ie, similar GMRs were observed for delta and wild-type for all study vaccines, when comparing with control groups). T-cell responses against delta and beta were similar to wild-type (table 5–7; appendix 1 p 17).

The median intervals between second and third doses in the group younger than 70 years and the group aged 70 years and older were similar in both the ChAd/ChAd (<70 years 78 days *vs* ≥70 years 77 days) and BNT/BNT (<70 years 106 days *vs* ≥70 years 97 days). Higher anti-spike IgG pre-third dose was observed in the people younger than 70 years with 1262 (95% CI 1171–1359; n=593) versus 1089 (1010–1175; n=686) in the group aged 70 years and older for ChAd/ChAd, and 4500 (4211–4808, n=679) versus 3344 (3083–3627; n=587) in the older group for BNT/BNT. Similar levels of pre-third dose cellular responses between two age groups were found in the ChAd/ChAd population (<70 years GM 38, 95% CI 33–43 [n=300] *vs* ≥70 years 36, 31–41 [n=336]), but not the BNT/BNT population (<70 years 47, 42–53 [n=324] *vs* ≥70 years 25, 22–29 [n=304]). At day 28 post third dose, similar levels of boost effect on anti-spike IgG and cellular responses were seen between the two age groups for all the study vaccines (figure 3).

As expected, baseline seropositive participants had higher humoral and cellular response compared with seronegative participants in both ChAd/ChAd and BNT/BNT populations (appendix 1 p 21). For both populations, the difference of anti-spike IgG and cellular response between seropositive and seronegative participants became smaller after a third dose vaccination. Seropositive participants still had higher immunogenicity (appendix 1 pp 37–38) compared with seronegative participants.

## Discussion

All COVID-19 vaccines and doses tested showed acceptable reactogenicity (figure 2). In both age groups, four vaccines showed higher moderate or severe local and systemic side-effects in the first 7 days: ChAd after BNT/BNT in the group aged 30–69 years (consistent with ChAd after BNT in the COMCOV trial^8^); and m1273 and CVn in all ages for ChAd and ChAd prime and in the group aged younger than 70 years for BNT/BNT prime (consistent with m1273 as dose two in the COMCOV2 trial^23^); and Ad26 after ChAd/ChAd or BNT/BNT in people younger than 70 years (appendix 1 pp 22–25). These data are consistent with early data from other trials of homologous and heterologous third dose boosters.^24^, ^25^, ^26^, ^27^, ^28^

The analysis shows there was good correlation seen for all vaccines between the pseudoneutralising assay NT_50_ against the wild-type and delta variants at days 0 and 28 (day 28 shown in appendix 1 p 34). Vaccines that produce antibodies against wild-type appear to neutralise delta effectively in vitro to a consistent, but slightly lesser degree, confirming the current public health strategy of using wild-type vaccines to control the currently predominant delta epidemic. Future analysis will investigate the in vitro killing against alpha and beta variants.

Findings from this trial demonstrate that the immunogenicity of homologous or heterologous third dose boost with all tested vaccines was superior to control regardless of which vaccine had been received in the initial course, apart from VLA, which did not achieve predefined criteria for minimum clinically important difference following BNT/BNT (figure 3).

Regarding spike IgG third dose response, it is important to recognise that, as yet, there is no established or well defined correlate of long-term protection. To date, both ChAd/ChAd (79%) and BNT/BNT (90%) have maintained highly effective real-world protection against hospitalisation and death after 6 months^1^ despite much higher absolute spike IgG levels for BNT/BNT than ChAd/ChAd.^8^ The relative role of T-cell or memory immunity is unclear, but is probably of great importance. The impact of dose interval remains to be fully elucidated—eg, there is better reported immunogenicity when a second dose of Ad26 is given at 6 months after the first dose of Ad26 compared with 2 months,^29^ and improved antibody responses when the initial BNT doses are spaced by 12 weeks rather than 3 weeks, although cellular response might be lower.^30^ There is potentially an important clinical impact of these changes, because people given initial ChAd doses spaced 3–4 weeks appear to have lower protection against infection than people receiving ChAd/ChAd 16 weeks apart.^31^

All vaccines tested (ChAd, BNT, m1273, NVX, Ad26, CVn, and VAL) boosted immunity after ChAd/ChAd as measured by anti-spike IgG and neutralising assays, and six vaccines (ChAd, BNT, m1273, NVX, Ad26, CVn) boosted immunity after BNT/BNT (table 5–7; figure 3). All of the vaccines that boosted immunity did so in older and younger people; however, there were marked differences in response between specific booster vaccines, consistent with other data from non-randomised studies.^24^ Therefore, these data endow immunisation advisory committees and policy makers with additional immunological and reactogenicity information, which will allow flexibility to deploy heterologous or homologous third doses after initial ChAd or BNT vaccines. These decisions will also be based on clinical, logistical, and supply considerations, targeted to the populations at greatest need.^32^

Cellular responses show that the mRNA vaccines and Ad26 show increased responses after ChAd/ChAd and BNT/BNT (table 5–7); however, as demonstrated elsewhere, ChAd does not boost cellular responses after ChAd/ChAd;^8^, ^26^ NVX boosts cellular responses better after ChAd/ChAd than BNT/BNT;^23^ and VLA does not induce any significant cellular responses to spike protein at day 28 compared with control after ChAd/ChAd or BNT/BNT. Data obtained at 3 months and 1 year after third dose will provide further information on the impact of third doses on long-term protection and immunological memory.

Fractional doses can be indirectly compared for BNT and half BNT, although they were in different site groups; some reduction in systemic effects was demonstrated compared with controls, although there was no apparent reduction in pain by using a half dose. There was a minimal decrease on immunogenicity by anti-spike IgG and neutralising assays (table 5–7). Maximum spike IgG responses were seen at 7 days after the third dose of half BNT, also for full dose BNT and m1273 after ChAd/ChAd or BNT/BNT. For other vaccines including the low (12 mg) dose CVn mRNA vaccine, an increment was seen from day 7 IgG to day 28 IgG concentrations (table 8–10). Along with the finding of no reduction in pain and some reduction in systemic effects, this could suggest that currently approved mRNA vaccines are formulated at doses above the minimum needed for a third (booster) dose. If immunogenicity can be maintained in larger dose reduction studies this could significantly increase the numbers of doses available globally. Our data suggest that even half dose BNT produces a vigorous anti-spike IgG response at 7 days. No clinical trial could be powered to demonstrate the impact of dose reduction on rare side-effects that are seen during population deployment. However, it is biologically plausible that lower fractional dosing for third dose boost could reduce inflammation and possibly rates of myocarditis seen after BNT or m1273 deployment. Of note, m1273 is now approved at 50% dose (50 μg) when used as a homologous third dose.^33^

Although neutralising responses can be predicted from spike IgG concentrations, we found that cellular responses do not correlate well (appendix 1 pp 35–36).

This study has a number of limitations. Due to pandemic timelines and the need to generate data to inform policy in September, 2021, the interval from second to third dose (given in June, 2021) was shorter in some participants than between their first two doses of ChAd/ChAd or BNT/BNT. This could lead to underestimates of boosting GMRs that would be achieved at longer dose intervals as immune responses of all types wane over time. The short interval to dose 3 might also mean the impact on T cells and immunological memory could be lower than if longer dose intervals had been used.^34^ This possibility is being investigated in a trial amendment that has offered COVID-19 third dose to people who previously received control vaccine. The age range (only recruiting people >30 years) limits the generalisability to younger populations, which might be particularly relevant with respect to reactogenicity, which was generally inversely proportional to age. The study also recruited a mostly White population. Due to the group design, not all vaccines were able to be randomised together, limiting the ability to compare vaccines between site groups. In particular, BNT was not able to be studied in the same group as half BNT; however, baseline characteristics between all site groups were all broadly comparable. Further analysis will be done comparing vaccines in different groups, adjusting for the difference between groups. It was also not possible to test half m1273 (currently now recommended as third dose of this vaccine^33^) or other approved vaccines due to logistical reasons at the timepoint of the decision to include fractional doses. A single laboratory was used to conduct all cell preparation and T-cell assays using the same protocols and reagents to avoid interlab variability and issues from cell freezing. Because of this, direct comparison with data from other studies conducted in different laboratories should review trends rather than compare absolute numbers. Finally, external laboratory capacity issues meant that this analysis did not include pseudoneutralising assay results against the alpha and beta variants, and there are currently no formally validated viral neutralisation assays against variants of concern.

ChAd has now been deployed in more than 180 countries and BNT in more than 145 countries.^35^ This trial has demonstrated the potential of all vaccines tested (ChAd, BNT, m1273, NVX, Ad26, CVn, and VAL) to boost immunity following an initial course of ChAd/ChAd and of six vaccines (ChAd, BNT, m1273, NVX, Ad26, and CVn) following an initial course of BNT/BNT. All vaccines showed acceptable side-effect profiles, although some schedules were more reactogenic than others.

Policy makers and national immunisation advisory committees should establish criteria for choosing which booster vaccines to use in their populations. This decision should be based on immunological considerations, known side-effect profiles, in-country availability, and ultimately a decision on what level of boost is sufficient in the context of national strategic disease control objectives.

## Data sharing

Individual participant data will be made available when the study is complete, on reasonable requests made to the corresponding author; data can be shared through secure online platforms after proposals are approved. All the sequence datasets used in the T-cell analysis are available in the public GISAID database (https://www.gisaid.org).

**This online publication has been corrected. The corrected version first appeared at thelancet.com on December 16, 2021**

## Declaration of interests

KCa acts on behalf of University Hospital Southampton as an investigator on studies funded or sponsored by vaccine manufacturers including AstraZeneca, GlaxoSmithKline, Janssen, Medimmune, Merck, Pfizer, Sanofi, and Valneva. She receives no personal financial payment for this work. SNF acts on behalf of University Hospital Southampton National Health Service (NHS) Foundation Trust as an investigator or providing consultative advice, or both, on clinical trials and studies of COVID-19 and other vaccines funded or sponsored by vaccine manufacturers including Janssen, Pfizer, AstraZeneca, GlaxoSmithKline, Novavax, Seqirus, Sanofi, Medimmune, Merck, and Valneva. He receives no personal financial payment for this work. ALG is named as an inventor on a patent covering use of a particular promoter construct that is often used in ChAdOx1-vectored vaccines and is incorporated in the ChAdOx1 nCoV-19 vaccine. ALG might benefit from royalty income paid to the University of Oxford from sales of this vaccine by AstraZeneca and its sublicensees under the University's revenue sharing policy. JH has received payments for presentations for AstraZeneca, Boehringer Ingelheim, Chiesi, Ciple, and Teva. VL acts on behalf of University College London Hospitals NHS Foundation Trust as an investigator on clinical trials of COVID-19 vaccines funded or sponsored by vaccine manufacturers including Pfizer, AstraZeneca, and Valneva. He receives no personal financial payment for this work. PM acts on behalf of University Hospital Southampton NHS Foundation Trust and The Adam Practice as an investigator on studies funded or sponsored by vaccine manufacturers including AstraZeneca, GlaxoSmithKline, Novavax, Medicago, and Sanofi. He received no personal financial payment for this work. JSN-V-T is seconded to the Department of Health and Social Care, England. MR has provided post marketing surveillance reports on vaccines for Pfizer and GlaxoSmithKline for which a cost recover charge is made. MDS acts on behalf of the University of Oxford as an investigator on studies funded or sponsored by vaccine manufacturers including AstraZeneca, GlaxoSmithKline, Pfizer, Novavax, Janssen, Medimmune, and MCM vaccines. He received no personal financial payment for this work. All other authors declare no competing interests.
