
JOURNAL INFORMATION
==============================
NLM Title Abbreviation: Lancet
Journal ID: Lancet
NLM Journal ID: 2985213R

Lancet (London, England)

ISSN: 0140-6736
EISSN: 1474-547X

ARTICLE INFORMATION
==============================
PMCID: PMC8676693
PMID: 34922669
DOI: 10.1016/S0140-6736(21)02785-9
Article ID: S0140-6736(21)02785-9
Article version: 2
Subjects: Department of Error

Department of Error

Print publication date: 2021 Dec 18
Volume: 398
Issue: 10318
First page: 2246
Last page: 2246
Copyright: © 2021 The Author(s). Published by Elsevier Ltd.
Copyright year: 2021
Copyright holder: Elsevier Ltd
License: This is an open access article under the CC BY license (http://creativecommons.org/licenses/by/4.0/).
License URL: https://creativecommons.org/licenses/by/4.0/
Erratum for: Safety and immunogenicity of seven COVID-19 vaccines as a third dose (booster) following two doses of ChAdOx1 nCov-19 or BNT162b2 in the UK (COV-BOOST): a blinded, multicentre, randomised, controlled, phase 2 trial 398 10318 18 12 2021 2258 2276 Lancet (London, England) 10.1016/S0140-6736(21)02717-3 PMC8639161 34863358

==============================
Munro APS, Janani L, Cornelius V, et al. Safety and immunogenicity of seven COVID-19 vaccines as a third dose (booster) following two doses of ChAdOx1 nCov-19 or BNT162b2 in the UK (COV-BOOST): a blinded, multicentre, randomised, controlled, phase 2 trial. Lancet 2021; 398: 2258–76—In this Article, Prof Teresa Lambe should have been included in the author list, and her affiliations should have been Oxford Vaccine Group, Department of Paediatrics, University of Oxford, Oxford, UK and NIHR Oxford Biomedical Research Centre, Oxford, UK. Jonathan Kwok's degree should have been MB BChir. In table 9, the Day 0 SARS-CoV-2 anti-spike IgG should have read 1443 (1051–1982; n=23) for the ChAd/ChAd-primed group who received BNT as a third dose. The appendix has been corrected. These corrections have been made to the online version as of Dec 16, 2021, and the printed version is correct.

