# Supplementary material for: Safety and immunogenicity of seven COVID-19 vaccines as a third dose (booster) following two doses of ChAdOx1 nCov-19 or BNT162b2 in the UK (COV-BOOST): a blinded, multicentre, randomised, controlled, phase 2 trial
Source: Lancet. 2021 Dec 18;398(10318):2258–76. doi: 10.1016/S0140-6736(21)02717-3 (PMC8639161; doi:10.1016/S0140-6736(21)02717-3)
Supplement: Supplementary appendix [file mmc1.pdf]

THE LANCET

Supplementary appendix

This appendix formed part of the original submission and has been peer reviewed. We post it as supplied by the authors.

Supplement to: Munro APS, Janani L, Cornelius V, et al. Safety and immunogenicity of seven COVID-19 vaccines as a third dose (booster) following two doses of ChAdOx1 nCov-19 or BNT162b2 in the UK (COV-BOOST): a blinded, multicentre, randomised, controlled, phase 2 trial. *Lancet* 2021; published online Dec 2. https://doi.org/10.1016/S0140-6736(21)02717-3.

Safety and immunogenicity of seven COVID-19 vaccines as a third dose (booster) following two doses of ChAdOx1 nCov19 or BNT162b2 in the UK (COV-BOOST): a blinded, multicentre, randomised, controlled, phase 2 trial

Supplementary Appendix 1

Contents

Supplementary Tables 2

Supplementary Table 1: Summary of Adverse Events (Group A) 2
Supplementary Table 2: Summary of Adverse Events (Group B) 5
Supplementary Table 3: Summary of Adverse Events (Group C) 8
Supplementary Table 4: Adverse Events of Special Interest 11
Supplementary Table 5: COVID-19 cases 13
Supplementary Table 6: Serious Adverse Events 14
Supplementary Table 7: Baseline vs Day 28 immunogenicity 17
Supplementary Table 8: Baseline immunogenicity by Serostatus (anti-nucleocapsid IgG).. 21

Supplementary Figures 22

Supplementary Figure 1: Solicited systemic and local reactions (Bar chart) 22
Supplementary Figure 2: Severity of solicited reactions (Radial chart) 26
Supplementary Figure 3: Kinetics of immunogenicity 33
Supplementary Figure 4: Correlation between PNA for WT and Delta 35
Supplementary Figure 5: Correlation between cellular response for WT and Beta 37
Supplementary Figure 6: Correlation between cellular response for WT and Delta 39
Supplementary Figure 7: Immunogenicity by Serostatus (anti-nucleocapsid IgG). 41
Supplementary Figure 8: Correlation between anti-spike IgG and PNA 44
Supplementary Figure 9: Correlation between anti-spike IgG and cellular response 46

Supplementary Laboratory Information 48

Laboratory Methods 48
Laboratory Units Conversion 48

Human SARS-CoV-2 Pre-Spike IgG ELISA Conversion 48
Human SARS-CoV-2 Pseudoparticle Neutralisation Assay (PNA) 48

COV-BOOST Study Group 49

Supplementary Tables

Supplementary Table 1: **Summary of Adverse Events (Group A)**

Summary of adverse events by 3rd dose vaccine allocation and priming vaccine schedule in Group A

	**Prime with ChAd/ChAd**				**Prime with BNT/BNT**			
**N=Number of vaccinated participants**	**Control (N=109)**	**ChAd (N=111)**	**NVX (N=115)**	**NVX-half (N=108)**	**Control (N=118)**	**ChAd (N=109)**	**NVX (N=114)**	**NVX-half (N=112)**
**Number of unique participants with at least one adverse event**	33	23	37	37	30	38	44	41
**Number of adverse events**	51	29	53	57	33	54	58	58
**Severity**								
Grade 1	29 (56.9%)	16 (55.2%)	32 (60.4%)	34 (59.6%)	13 (39.4%)	36 (66.7%)	31 (53.4%)	28 (48.3%)
Grade 2	20 (39.2%)	11 (37.9%)	19 (35.8%)	20 (35.1%)	16 (48.5%)	11 (20.4%)	22 (37.9%)	26 (44.8%)
Grade 3	2 (3.9%)	1 (3.4%)	2 (3.8%)	3 (5.3%)	3 (9.1%)	7 (13.0%)	4 (6.9%)	2 (3.4%)
Grade 4	0 (0.0%)	1 (3.4%)	0 (0.0%)	0 (0.0%)	1 (3.0%)	0 (0.0%)	1 (1.7%)	2 (3.4%)
Not reported	0 (0.0%)	0 (0.0%)	0 (0.0%)	0 (0.0%)	0 (0.0%)	0 (0.0%)	0 (0.0%)	0 (0.0%)
**Causality**								
No relationship	17 (33.3%)	10 (34.5%)	21 (39.6%)	23 (40.4%)	17 (51.5%)	18 (33.3%)	20 (34.5%)	20 (34.5%)
Unlikely	17 (33.3%)	11 (37.9%)	21 (39.6%)	18 (31.6%)	12 (36.4%)	19 (35.2%)	12 (20.7%)	25 (43.1%)
Possible	11 (21.6%)	3 (10.3%)	10 (18.9%)	9 (15.8%)	3 (9.1%)	7 (13.0%)	20 (34.5%)	9 (15.5%)
Probable	6 (11.8%)	4 (13.8%)	1 (1.9%)	3 (5.3%)	1 (3.0%)	8 (14.8%)	5 (8.6%)	3 (5.2%)
Definite	0 (0.0%)	1 (3.4%)	0 (0.0%)	4 (7.0%)	0 (0.0%)	2 (3.7%)	1 (1.7%)	1 (1.7%)
Not reported	0 (0.0%)	0 (0.0%)	0 (0.0%)	0 (0.0%)	0 (0.0%)	0 (0.0%)	0 (0.0%)	0 (0.0%)
**System Organ Classes (SOC)**								

Blood and lymphatic system disorders	3 (5.9%)	1 (3.4%)	4 (7.5%)	1 (1.8%)	1 (3.0%)	5 (9.3%)	0 (0.0%)	2 (3.4%)
Cardiac disorders	1 (2.0%)	0 (0.0%)	2 (3.8%)	0 (0.0%)	2 (6.1%)	3 (5.6%)	2 (3.4%)	2 (3.4%)
Congenital, familial and genetic disorders	0 (0.0%)	0 (0.0%)	0 (0.0%)	0 (0.0%)	0 (0.0%)	0 (0.0%)	1 (1.7%)	0 (0.0%)
Ear and labyrinth disorders	3 (5.9%)	2 (6.9%)	1 (1.9%)	1 (1.8%)	0 (0.0%)	2 (3.7%)	1 (1.7%)	3 (5.2%)
Endocrine disorders	0 (0.0%)	0 (0.0%)	0 (0.0%)	0 (0.0%)	0 (0.0%)	0 (0.0%)	0 (0.0%)	0 (0.0%)
Eye disorders	0 (0.0%)	0 (0.0%)	0 (0.0%)	0 (0.0%)	0 (0.0%)	2 (3.7%)	0 (0.0%)	1 (1.7%)
Gastrointestinal disorders	4 (7.8%)	1 (3.4%)	2 (3.8%)	7 (12.3%)	2 (6.1%)	0 (0.0%)	3 (5.2%)	6 (10.3%)
General disorders and administration site conditions	6 (11.8%)	4 (13.8%)	10 (18.9%)	6 (10.5%)	2 (6.1%)	6 (11.1%)	7 (12.1%)	7 (12.1%)
Hepatobiliary disorders	0 (0.0%)	0 (0.0%)	0 (0.0%)	0 (0.0%)	1 (3.0%)	0 (0.0%)	0 (0.0%)	1 (1.7%)
Immune system disorders	0 (0.0%)	0 (0.0%)	0 (0.0%)	1 (1.8%)	1 (3.0%)	3 (5.6%)	0 (0.0%)	0 (0.0%)
Infections and infestations	6 (11.8%)	3 (10.3%)	4 (7.5%)	4 (7.0%)	3 (9.1%)	8 (14.8%)	4 (6.9%)	6 (10.3%)
Injury, poisoning and procedural complications	0 (0.0%)	1 (3.4%)	2 (3.8%)	2 (3.5%)	1 (3.0%)	0 (0.0%)	1 (1.7%)	2 (3.4%)
Investigations	3 (5.9%)	1 (3.4%)	5 (9.4%)	3 (5.3%)	3 (9.1%)	2 (3.7%)	1 (1.7%)	2 (3.4%)
Metabolism and nutrition disorders	1 (2.0%)	0 (0.0%)	2 (3.8%)	0 (0.0%)	2 (6.1%)	1 (1.9%)	4 (6.9%)	1 (1.7%)
Musculoskeletal and connective tissue disorders	6 (11.8%)	3 (10.3%)	9 (17.0%)	7 (12.3%)	2 (6.1%)	5 (9.3%)	8 (13.8%)	7 (12.1%)
Neoplasms benign, malignant and unspecified (incl cysts and polyps)	0 (0.0%)	0 (0.0%)	0 (0.0%)	1 (1.8%)	0 (0.0%)	1 (1.9%)	0 (0.0%)	1 (1.7%)
Nervous system disorders	8 (15.7%)	1 (3.4%)	3 (5.7%)	8 (14.0%)	6 (18.2%)	2 (3.7%)	5 (8.6%)	5 (8.6%)

Pregnancy, puerperium and perinatal conditions	0 (0.0%)	0 (0.0%)	0 (0.0%)	0 (0.0%)	0 (0.0%)	0 (0.0%)	0 (0.0%)	0 (0.0%)
Product issues	0 (0.0%)	0 (0.0%)	0 (0.0%)	0 (0.0%)	0 (0.0%)	0 (0.0%)	0 (0.0%)	0 (0.0%)
Psychiatric disorders	0 (0.0%)	1 (3.4%)	0 (0.0%)	1 (1.8%)	0 (0.0%)	3 (5.6%)	2 (3.4%)	0 (0.0%)
Renal and urinary disorders	1 (2.0%)	0 (0.0%)	1 (1.9%)	1 (1.8%)	2 (6.1%)	1 (1.9%)	1 (1.7%)	1 (1.7%)
Reproductive system and breast disorders	1 (2.0%)	2 (6.9%)	0 (0.0%)	1 (1.8%)	0 (0.0%)	3 (5.6%)	1 (1.7%)	0 (0.0%)
Respiratory, thoracic and mediastinal disorders	3 (5.9%)	6 (20.7%)	2 (3.8%)	6 (10.5%)	3 (9.1%)	4 (7.4%)	9 (15.5%)	2 (3.4%)
Skin and subcutaneous tissue disorders	1 (2.0%)	2 (6.9%)	3 (5.7%)	5 (8.8%)	0 (0.0%)	2 (3.7%)	5 (8.6%)	6 (10.3%)
Social circumstances	0 (0.0%)	0 (0.0%)	0 (0.0%)	0 (0.0%)	0 (0.0%)	0 (0.0%)	0 (0.0%)	0 (0.0%)
Surgical and medical procedures	1 (2.0%)	0 (0.0%)	2 (3.8%)	1 (1.8%)	0 (0.0%)	0 (0.0%)	0 (0.0%)	1 (1.7%)
Vascular disorders	3 (5.9%)	1 (3.4%)	1 (1.9%)	1 (1.8%)	2 (6.1%)	1 (1.9%)	3 (5.2%)	2 (3.4%)
**Menstrual disorder**								
Yes	0 (0.0%)	1 (3.4%)	0 (0.0%)	1 (1.8%)	0 (0.0%)	2 (3.7%)	0 (0.0%)	0 (0.0%)

* Data are N (%) unless otherwise indicated. Control =quadrivalent meningococcal conjugate vaccine; ChAd=ChAdOx1 nCoV-19 vaccine, Oxford–AstraZeneca; BNT=BNT162b2 vaccine (Pfizer–BioNTech); NVX=NVX-CoV2373 vaccine, Novavax; NVX half=half dose of NVX-CoV2373 vaccine.

Supplementary Table 2: Summary of Adverse Events (Group B)

Summary of adverse events by 3rd dose vaccine allocation and priming vaccine schedule in Group B

	**Prime with ChAd/ChAd**					**Prime with BNT/BNT**				
**N=Number of vaccinated participants**	**Control (N=105)**	**BNT (N=106)**	**VLA (N=109)**	**VLA-half (N=111)**	**Ad26 (N=108)**	**Control (N=109)**	**BNT (N=109)**	**VLA (N=110)**	**VLA-half (N=110)**	**Ad26 (N=106)**
**Number of unique participants with at least one adverse event**	26	46	46	39	44	33	32	36	35	35
**Number of adverse events**	37	57	61	51	68	41	53	48	48	48
**Severity**										
Grade 1	18 (48.6%)	25 (43.9%)	32 (52.5%)	37 (72.5%)	35 (51.5%)	28 (68.3%)	33 (62.3%)	36 (75.0%)	28 (58.3%)	31 (64.6%)
Grade 2	15 (40.5%)	25 (43.9%)	20 (32.8%)	12 (23.5%)	24 (35.3%)	12 (29.3%)	17 (32.1%)	9 (18.8%)	11 (22.9%)	13 (27.1%)
Grade 3	3 (8.1%)	7 (12.3%)	6 (9.8%)	2 (3.9%)	8 (11.8%)	1 (2.4%)	2 (3.8%)	3 (6.2%)	8 (16.7%)	4 (8.3%)
Grade 4	1 (2.7%)	0 (0.0%)	3 (4.9%)	0 (0.0%)	1 (1.5%)	0 (0.0%)	1 (1.9%)	0 (0.0%)	1 (2.1%)	0 (0.0%)
Not reported	0 (0.0%)	0 (0.0%)	0 (0.0%)	0 (0.0%)	0 (0.0%)	0 (0.0%)	0 (0.0%)	0 (0.0%)	0 (0.0%)	0 (0.0%)
**Causality**										
No relationship	14 (37.8%)	26 (45.6%)	29 (47.5%)	23 (45.1%)	28 (41.2%)	19 (46.3%)	13 (24.5%)	18 (37.5%)	21 (43.8%)	17 (35.4%)
Unlikely	16 (43.2%)	12 (21.1%)	15 (24.6%)	13 (25.5%)	15 (22.1%)	10 (24.4%)	10 (18.9%)	17 (35.4%)	10 (20.8%)	16 (33.3%)
Possible	3 (8.1%)	12 (21.1%)	13 (21.3%)	6 (11.8%)	16 (23.5%)	5 (12.2%)	16 (30.2%)	10 (20.8%)	11 (22.9%)	4 (8.3%)
Probable	3 (8.1%)	7 (12.3%)	4 (6.6%)	6 (11.8%)	7 (10.3%)	6 (14.6%)	8 (15.1%)	2 (4.2%)	5 (10.4%)	9 (18.8%)
Definite	1 (2.7%)	0 (0.0%)	0 (0.0%)	3 (5.9%)	2 (2.9%)	1 (2.4%)	6 (11.3%)	1 (2.1%)	1 (2.1%)	2 (4.2%)
Not reported	0 (0.0%)	0 (0.0%)	0 (0.0%)	0 (0.0%)	0 (0.0%)	0 (0.0%)	0 (0.0%)	0 (0.0%)	0 (0.0%)	0 (0.0%)
**System Organ Classes (SOC)**										

Blood and lymphatic system disorders	0 (0.0%)	2 (3.5%)	4 (6.6%)	0 (0.0%)	0 (0.0%)	2 (4.9%)	5 (9.4%)	1 (2.1%)	1 (2.1%)	1 (2.1%)
Cardiac disorders	1 (2.7%)	4 (7.0%)	4 (6.6%)	0 (0.0%)	1 (1.5%)	1 (2.4%)	1 (1.9%)	1 (2.1%)	1 (2.1%)	1 (2.1%)
Congenital, familial and genetic disorders	0 (0.0%)	0 (0.0%)	1 (1.6%)	0 (0.0%)	0 (0.0%)	0 (0.0%)	0 (0.0%)	0 (0.0%)	0 (0.0%)	0 (0.0%)
Ear and labyrinth disorders	0 (0.0%)	0 (0.0%)	2 (3.3%)	1 (2.0%)	0 (0.0%)	0 (0.0%)	2 (3.8%)	3 (6.2%)	1 (2.1%)	0 (0.0%)
Endocrine disorders	0 (0.0%)	0 (0.0%)	0 (0.0%)	0 (0.0%)	0 (0.0%)	0 (0.0%)	0 (0.0%)	0 (0.0%)	1 (2.1%)	0 (0.0%)
Eye disorders	2 (5.4%)	0 (0.0%)	0 (0.0%)	0 (0.0%)	0 (0.0%)	1 (2.4%)	0 (0.0%)	0 (0.0%)	0 (0.0%)	0 (0.0%)
Gastrointestinal disorders	3 (8.1%)	5 (8.8%)	4 (6.6%)	6 (11.8%)	6 (8.8%)	5 (12.2%)	5 (9.4%)	3 (6.2%)	3 (6.2%)	3 (6.2%)
General disorders and administration site conditions	3 (8.1%)	4 (7.0%)	4 (6.6%)	7 (13.7%)	7 (10.3%)	4 (9.8%)	10 (18.9%)	6 (12.5%)	7 (14.6%)	4 (8.3%)
Hepatobiliary disorders	0 (0.0%)	0 (0.0%)	1 (1.6%)	0 (0.0%)	1 (1.5%)	0 (0.0%)	1 (1.9%)	0 (0.0%)	0 (0.0%)	0 (0.0%)
Immune system disorders	0 (0.0%)	1 (1.8%)	1 (1.6%)	0 (0.0%)	2 (2.9%)	0 (0.0%)	0 (0.0%)	1 (2.1%)	2 (4.2%)	2 (4.2%)
Infections and infestations	3 (8.1%)	6 (10.5%)	6 (9.8%)	7 (13.7%)	11(16.2%)	1 (2.4%)	4 (7.5%)	5 (10.4%)	3 (6.2%)	4 (8.3%)
Injury, poisoning and procedural complications	1 (2.7%)	0 (0.0%)	0 (0.0%)	4 (7.8%)	0 (0.0%)	2 (4.9%)	0 (0.0%)	2 (4.2%)	0 (0.0%)	1 (2.1%)
Investigations	5 (13.5%)	2 (3.5%)	6 (9.8%)	3 (5.9%)	6 (8.8%)	4 (9.8%)	0 (0.0%)	5 (10.4%)	1 (2.1%)	1 (2.1%)
Metabolism and nutrition disorders	2 (5.4%)	0 (0.0%)	2 (3.3%)	1 (2.0%)	1 (1.5%)	1 (2.4%)	2 (3.8%)	0 (0.0%)	2 (4.2%)	2 (4.2%)
Musculoskeletal and connective tissue disorders	6 (16.2%)	13 (22.8%)	7 (11.5%)	7 (13.7%)	8 (11.8%)	4 (9.8%)	7 (13.2%)	6 (12.5%)	8 (16.7%)	6 (12.5%)
Neoplasms benign, malignant and unspecified (incl cysts and polyps)	0 (0.0%)	2 (3.5%)	0 (0.0%)	0 (0.0%)	0 (0.0%)	1 (2.4%)	0 (0.0%)	0 (0.0%)	1 (2.1%)	0 (0.0%)
Nervous system disorders	6 (16.2%)	6 (10.5%)	4 (6.6%)	8 (15.7%)	7 (10.3%)	3 (7.3%)	5 (9.4%)	6 (12.5%)	6 (12.5%)	6 (12.5%)

Pregnancy, puerperium and perinatal conditions	0 (0.0%)	0 (0.0%)	0 (0.0%)	0 (0.0%)	0 (0.0%)	0 (0.0%)	0 (0.0%)	0 (0.0%)	0 (0.0%)	0 (0.0%)
Product issues	0 (0.0%)	0 (0.0%)	0 (0.0%)	0 (0.0%)	0 (0.0%)	0 (0.0%)	0 (0.0%)	0 (0.0%)	0 (0.0%)	0 (0.0%)
Psychiatric disorders	0 (0.0%)	1 (1.8%)	0 (0.0%)	1 (2.0%)	4 (5.9%)	0 (0.0%)	0 (0.0%)	0 (0.0%)	1 (2.1%)	2 (4.2%)
Renal and urinary disorders	0 (0.0%)	0 (0.0%)	2 (3.3%)	1 (2.0%)	3 (4.4%)	1 (2.4%)	1 (1.9%)	1 (2.1%)	0 (0.0%)	2 (4.2%)
Reproductive system and breast disorders	2 (5.4%)	1 (1.8%)	2 (3.3%)	1 (2.0%)	1 (1.5%)	0 (0.0%)	2 (3.8%)	2 (4.2%)	0 (0.0%)	1 (2.1%)
Respiratory, thoracic and mediastinal disorders	2 (5.4%)	1 (1.8%)	2 (3.3%)	2 (3.9%)	2 (2.9%)	7 (17.1%)	3 (5.7%)	2 (4.2%)	5 (10.4%)	5 (10.4%)
Skin and subcutaneous tissue disorders	1 (2.7%)	4 (7.0%)	5 (8.2%)	2 (3.9%)	2 (2.9%)	1 (2.4%)	4 (7.5%)	1 (2.1%)	3 (6.2%)	3 (6.2%)
Social circumstances	0 (0.0%)	0 (0.0%)	0 (0.0%)	0 (0.0%)	0 (0.0%)	0 (0.0%)	0 (0.0%)	0 (0.0%)	0 (0.0%)	0 (0.0%)
Surgical and medical procedures	0 (0.0%)	1 (1.8%)	0 (0.0%)	0 (0.0%)	1 (1.5%)	1 (2.4%)	0 (0.0%)	1 (2.1%)	0 (0.0%)	1 (2.1%)
Vascular disorders	0 (0.0%)	4 (7.0%)	4 (6.6%)	0 (0.0%)	5 (7.4%)	2 (4.9%)	1 (1.9%)	2 (4.2%)	2 (4.2%)	3 (6.2%)
**Menstrual disorder**										
Yes	0 (0.0%)	0 (0.0%)	1 (1.6%)	0 (0.0%)	1 (1.5%)	0 (0.0%)	2 (3.8%)	1 (2.1%)	0 (0.0%)	1 (2.1%)

* Data are N (%) unless otherwise indicated. Control =quadrivalent meningococcal conjugate vaccine; ChAd=ChAdOx1 nCoV-19 vaccine, Oxford–AstraZeneca; BNT=BNT162b2 vaccine (Pfizer–BioNTech); VLA=VLA2001 vaccine, Valneva; VLA half=half dose of VLA2001 vaccine; Ad26=Ad26.COV2.S vaccine, Janssen.

Supplementary Table 3: Summary of Adverse Events (Group C)

Summary of adverse events by 3rd dose vaccine allocation and priming vaccine schedule in Group C

	**Prime with ChAd/ChAd**				**Prime with BNT/BNT**			
**N=Number of vaccinated participants**	**Control (N=114)**	**BNT-half (N=116)**	**m1273 (N=112)**	**CVn (N=119)**	**Control (N=112)**	**BNT-half (N=110)**	**m1273 (N=110)**	**CVn (N=106)**
**Number of unique participants with at least one adverse event**	33	26	36	42	27	30	31	32
**Number of adverse events**	46	31	69	86	36	42	50	41
**Severity**								
Grade 1	30 (65.2%)	18 (58.1%)	49 (71.0%)	56 (65.1%)	19 (52.8%)	32 (76.2%)	37 (74.0%)	30 (73.2%)
Grade 2	12 (26.1%)	12 (38.7%)	17 (24.6%)	25 (29.1%)	15 (41.7%)	7 (16.7%)	10 (20.0%)	9 (22.0%)
Grade 3	4 (8.7%)	1 (3.2%)	2 (2.9%)	4 (4.7%)	2 (5.6%)	3 (7.1%)	3 (6.0%)	1 (2.4%)
Grade 4	0 (0.0%)	0 (0.0%)	1 (1.4%)	0 (0.0%)	0 (0.0%)	0 (0.0%)	0 (0.0%)	1 (2.4%)
Not reported	0 (0.0%)	0 (0.0%)	0 (0.0%)	1 (1.2%)	0 (0.0%)	0 (0.0%)	0 (0.0%)	0 (0.0%)
**Causality**								
No relationship	8 (17.4%)	5 (16.1%)	11 (15.9%)	11 (12.8%)	9 (25.0%)	7 (16.7%)	7 (14.0%)	8 (19.5%)
Unlikely	18 (39.1%)	10 (32.3%)	13 (18.8%)	20 (23.3%)	7 (19.4%)	12 (28.6%)	18 (36.0%)	15 (36.6%)
Possible	15 (32.6%)	12 (38.7%)	28 (40.6%)	46 (53.5%)	17 (47.2%)	10 (23.8%)	9 (18.0%)	14 (34.1%)
Probable	4 (8.7%)	4 (12.9%)	15 (21.7%)	9 (10.5%)	3 (8.3%)	12 (28.6%)	16 (32.0%)	3 (7.3%)
Definite	1 (2.2%)	0 (0.0%)	1 (1.4%)	0 (0.0%)	0 (0.0%)	1 (2.4%)	0 (0.0%)	1 (2.4%)
Not reported	0 (0.0%)	0 (0.0%)	1 (1.4%)	0 (0.0%)	0 (0.0%)	0 (0.0%)	0 (0.0%)	0 (0.0%)
**System Organ Classes (SOC)**								

Blood and lymphatic system disorders	0 (0.0%)	3 (9.7%)	0 (0.0%)	1 (1.2%)	1 (2.8%)	4 (9.5%)	4 (8.0%)	2 (4.9%)
Cardiac disorders	0 (0.0%)	0 (0.0%)	3 (4.3%)	1 (1.2%)	0 (0.0%)	0 (0.0%)	0 (0.0%)	1 (2.4%)
Congenital, familial and genetic disorders	0 (0.0%)	0 (0.0%)	0 (0.0%)	0 (0.0%)	0 (0.0%)	0 (0.0%)	0 (0.0%)	0 (0.0%)
Ear and labyrinth disorders	1 (2.2%)	0 (0.0%)	0 (0.0%)	0 (0.0%)	0 (0.0%)	0 (0.0%)	0 (0.0%)	1 (2.4%)
Endocrine disorders	0 (0.0%)	0 (0.0%)	0 (0.0%)	0 (0.0%)	0 (0.0%)	0 (0.0%)	0 (0.0%)	0 (0.0%)
Eye disorders	1 (2.2%)	0 (0.0%)	3 (4.3%)	2 (2.3%)	0 (0.0%)	0 (0.0%)	1 (2.0%)	0 (0.0%)
Gastrointestinal disorders	1 (2.2%)	3 (9.7%)	6 (8.7%)	11 (12.8%)	3 (8.3%)	2 (4.8%)	4 (8.0%)	1 (2.4%)
General disorders and administration site conditions	6 (13.0%)	5 (16.1%)	24 (34.8%)	26 (30.2%)	9 (25.0%)	8 (19.0%)	9 (18.0%)	8 (19.5%)
Hepatobiliary disorders	0 (0.0%)	0 (0.0%)	0 (0.0%)	0 (0.0%)	0 (0.0%)	0 (0.0%)	0 (0.0%)	0 (0.0%)
Immune system disorders	0 (0.0%)	0 (0.0%)	0 (0.0%)	0 (0.0%)	0 (0.0%)	3 (7.1%)	1 (2.0%)	1 (2.4%)
Infections and infestations	3 (6.5%)	3 (9.7%)	5 (7.2%)	5 (5.8%)	5 (13.9%)	1 (2.4%)	1 (2.0%)	4 (9.8%)
Injury, poisoning and procedural complications	0 (0.0%)	0 (0.0%)	0 (0.0%)	1 (1.2%)	1 (2.8%)	1 (2.4%)	1 (2.0%)	1 (2.4%)
Investigations	0 (0.0%)	0 (0.0%)	0 (0.0%)	1 (1.2%)	0 (0.0%)	1 (2.4%)	0 (0.0%)	2 (4.9%)
Metabolism and nutrition disorders	0 (0.0%)	0 (0.0%)	0 (0.0%)	2 (2.3%)	0 (0.0%)	1 (2.4%)	2 (4.0%)	2 (4.9%)
Musculoskeletal and connective tissue disorders	13 (28.3%)	7 (22.6%)	11 (15.9%)	8 (9.3%)	2 (5.6%)	12 (28.6%)	7 (14.0%)	6 (14.6%)
Neoplasms benign, malignant and unspecified (incl cysts and polyps)	0 (0.0%)	0 (0.0%)	0 (0.0%)	0 (0.0%)	0 (0.0%)	0 (0.0%)	0 (0.0%)	0 (0.0%)
Nervous system disorders	9 (19.6%)	5 (16.1%)	7 (10.1%)	8 (9.3%)	6 (16.7%)	2 (4.8%)	8 (16.0%)	4 (9.8%)

Pregnancy, puerperium and perinatal conditions	0 (0.0%)	0 (0.0%)	0 (0.0%)	0 (0.0%)	0 (0.0%)	0 (0.0%)	0 (0.0%)	0 (0.0%)
Product issues	0 (0.0%)	0 (0.0%)	0 (0.0%)	0 (0.0%)	0 (0.0%)	0 (0.0%)	0 (0.0%)	0 (0.0%)
Psychiatric disorders	2 (4.3%)	1 (3.2%)	3 (4.3%)	1 (1.2%)	1 (2.8%)	0 (0.0%)	2 (4.0%)	0 (0.0%)
Renal and urinary disorders	3 (6.5%)	0 (0.0%)	1 (1.4%)	2 (2.3%)	4 (11.1%)	3 (7.1%)	0 (0.0%)	0 (0.0%)
Reproductive system and breast disorders	0 (0.0%)	1 (3.2%)	1 (1.4%)	4 (4.7%)	1 (2.8%)	2 (4.8%)	1 (2.0%)	0 (0.0%)
Respiratory, thoracic and mediastinal disorders	3 (6.5%)	1 (3.2%)	2 (2.9%)	5 (5.8%)	2 (5.6%)	1 (2.4%)	4 (8.0%)	2 (4.9%)
Skin and subcutaneous tissue disorders	4 (8.7%)	1 (3.2%)	3 (4.3%)	3 (3.5%)	0 (0.0%)	0 (0.0%)	1 (2.0%)	2 (4.9%)
Social circumstances	0 (0.0%)	0 (0.0%)	0 (0.0%)	0 (0.0%)	0 (0.0%)	0 (0.0%)	0 (0.0%)	0 (0.0%)
Surgical and medical procedures	0 (0.0%)	0 (0.0%)	0 (0.0%)	0 (0.0%)	0 (0.0%)	0 (0.0%)	1 (2.0%)	1 (2.4%)
Vascular disorders	0 (0.0%)	1 (3.2%)	0 (0.0%)	5 (5.8%)	1 (2.8%)	1 (2.4%)	3 (6.0%)	3 (7.3%)
**Menstrual disorder**								
Yes	0 (0.0%)	0 (0.0%)	0 (0.0%)	3 (3.5%)	0 (0.0%)	0 (0.0%)	0 (0.0%)	0 (0.0%)

* Data are N (%) unless otherwise indicated. Control =quadrivalent meningococcal conjugate vaccine; ChAd=ChAdOx1 nCoV-19 vaccine, Oxford–AstraZeneca; BNT=BNT162b2 vaccine (Pfizer–BioNTech); BNT half=half dose of BNT162b2 vaccine; m1273=mRNA1273 vaccine, Moderna; CVn=CVnCoV vaccine, Curevac.

Supplementary Table 4: Adverse Events of Special Interest

Adverse events of special interest in all study arms (Excluding SAEs and COVID-19 cases in immediate 14 days after 3rd dose)

Study arm	Site	Days to onset from boost	MedDRA Preferred Term	MedDRA System Order Class	Severity	Causality assessment
Ad26	University Hospital Southampton NHS FT	19	Acute kidney injury	Renal and urinary disorders	Grade3	Unlikely
VLA	University Hospital Southampton NHS FT	35	Ventricular extrasystoles	Cardiac disorders	Grade1	Unlikely
VLA	Guys and St Thomas' NHS FT	28	Acute kidney injury	Renal and urinary disorders	Grade1	Possible
BNT	Guys and St Thomas' NHS FT	0	Thrombocytopenia	Blood and lymphatic system disorders	Grade3	No relationship
Ad26	Guys and St Thomas' NHS FT	5	Epistaxis	Vascular disorders	Grade3	Unlikely
Ad26	Guys and St Thomas' NHS FT	21	Paraesthesia	Nervous system disorders	Grade2	Possible
VLA	Leeds Teaching Hospitals NHS Trust	12	COVID-19	Infections and infestations	Grade2	No relationship
Control-group B	Leeds Teaching Hospitals NHS Trust	21	Acute kidney injury	Renal and urinary disorders	Grade2	No relationship
VLA-half	Leeds Teaching Hospitals NHS Trust	12	COVID-19	Infections and infestations	Grade2	No relationship
m1273	Royal Devon and Exeter NHS FT	23	Acute kidney injury	Renal and urinary disorders	Grade1	Unlikely
Control-group C	Royal Devon and Exeter NHS FT	29	Acute kidney injury	Renal and urinary disorders	Grade3	Unlikely

CVn	University Hospitals Birmingham NHS FT	4	COVID-19	Infections and infestations	Grade1	No relationship
BNT-half	University Hospitals Birmingham NHS FT	6	COVID-19	Infections and infestations	Grade1	No relationship
Control-group C	University Hospitals Birmingham NHS FT	2	COVID-19	Infections and infestations	Grade1	No relationship
Control-group A	University Hospitals of Leicester NHS FT	0	Pericarditis	Cardiac disorders	Grade1	Possible
Control-group C	London North West University Healthcare NHS Trust	29	Acute kidney injury	Renal and urinary disorders	Grade3	Unlikely
BNT-half	London North West University Healthcare NHS Trust	28	Acute kidney injury	Renal and urinary disorders	Grade3	Possible
Control-group C	London North West University Healthcare NHS Trust	28	Acute kidney injury	Renal and urinary disorders	Grade3	Unlikely
CVn	Brighton and Sussex University Hospitals NHS Trust	1	COVID-19	Infections and infestations	Grade2	No relationship
Control-group C	Brighton and Sussex University Hospitals NHS Trust	26	Acute kidney injury	Renal and urinary disorders	Grade2	Possible

Supplementary Table 5: COVID-19 cases

Adverse events of special interest - COVID-19 cases, by study arm

Sites	Days to onset post 3rd dose	Arm
Bradford Teaching Hospitals NHS FT	39	Control- group A
Public Health Wales	65	Control- group A
Bradford Teaching Hospitals NHS FT	51	ChAd
University Hospitals of Leicester NHS FT	28	NVX
University College London Hospitals NHS FT	57	NVX-half
Public Health Wales	30	NVX-half
University Hospital Southampton NHS FT	50	Control- group B
University of Oxford	66	Control- group B
University of Oxford	28	Control- group B
Guys and St Thomas' NHS FT	60	Control- group B
Guys and St Thomas' NHS FT	52	VLA
NHS Greater Glasgow and Clyde	66	VLA
Cambridge University Hospitals NHS FT	18	VLA
Cambridge University Hospitals NHS FT	34	VLA
University Hospital Southampton NHS FT	17	VLA-half
Guys and St Thomas' NHS FT	28	VLA-half
Leeds Teaching Hospitals NHS Trust	25	VLA-half
NHS Greater Glasgow and Clyde	37	VLA-half
Cambridge University Hospitals NHS FT	59	Ad26
University Hospitals Birmingham NHS FT	31	Control- group C
Royal Devon and Exeter NHS FT	33	Control- group C

Supplementary Table 6: Serious Adverse Events

Study arm	Site	Days to onset from boost	MedDRA Preferred Term	MedDRA System Order Class	Severity	Serious adverse event type	Causality assessment
Ad26	University Hospital Southampton NHS FT	12	Cholecystitis	Hepatobiliary disorders	Grade3	Hospitalisation	Unlikely
VLA	University Hospital Southampton NHS FT	28	Liver injury	Hepatobiliary disorders	Grade4	An important medical event	Possible
Ad26	University of Oxford	33	Cerebrovascular accident	Nervous system disorders	Grade4	An important medical event	Possible
Control-group B	University of Oxford	15	Transient global amnesia	Nervous system disorders	Grade4	Hospitalisation	Unlikely
Control-group B	Guys and St Thomas' NHS FT	55	Breast cancer	Reproductive system and breast disorders	Grade3	An important medical event	No relationship
VLA	Leeds Teaching Hospitals NHS Trust	19	Myocardial infarction	Cardiac disorders	Grade3	Hospitalisation	Possible
VLA-half	Leeds Teaching Hospitals NHS Trust	0	Oesophageal squamous cell carcinoma	Gastrointestinal disorders	Grade3	An important medical event	No relationship
Control-group B	Leeds Teaching Hospitals NHS Trust	20	Cerebrovascular accident	Nervous system disorders	Grade3	Hospitalisation	Unlikely

VLA	Leeds Teaching Hospitals NHS Trust	46	Breast cancer	Reproductive system and breast disorders	Grade3	An important medical event	No relationship
VLA	NHS Greater Glasgow and Clyde	15	Sinus node dysfunction	Cardiac disorders	Grade4	Hospitalisation	Unlikely
BNT	NHS Greater Glasgow and Clyde	16	Liver injury	Hepatobiliary disorders	Grade4	Hospitalisation	Unlikely
ChAd	Bradford Teaching Hospitals NHS FT	24	Urinary tract infection	Infections and infestations	Grade4	Hospitalisation	No relationship
NVX	Bradford Teaching Hospitals NHS FT	58	Urinary tract infection	Infections and infestations	Grade3	An important medical event	No relationship
CVn	Royal Devon and Exeter NHS FT	28	Hyponatraemia	Metabolism and nutrition disorders	Grade4	An important medical event	Unlikely
Control-group A	University College London Hospitals NHS FT	55	Abdominal pain	Gastrointestinal disorders	Grade2	Hospitalisation	No relationship
Control-group A	University College London Hospitals NHS FT	28	Hyperbilirubinaemia	Hepatobiliary disorders	Grade4	An important medical event	No relationship
VLA	Cambridge University Hospitals NHS FT	49	Pyelocaliectasis	Renal and urinary disorders	Grade3	Hospitalisation	No relationship
NVX-half	Dorset Research Hub	31	Pericarditis	Cardiac disorders	Grade4	An important medical event	No relationship

ChAd	Portsmouth Research Hub	4	Malignant Melanoma	Neoplasms benign, malignant and unspecified (incl cysts and polyps)	Grade3	An important medical event	No relationship
BNT-half	University Hospitals Birmingham NHS FT	37	Eye haemorrhage	Vascular disorders	Grade3	An important medical event	Possible
NVX	University Hospitals of Leicester NHS FT	13	Ovarian cancer	Reproductive system and breast disorders	Grade4	An important medical event	No relationship
NVX-half	University Hospitals of Leicester NHS FT	51	Gastrointestinal carcinoma	Neoplasms benign, malignant and unspecified (incl cysts and polyps)	Grade4	Hospitalisation	No relationship
M1273	Liverpool University Hospitals NHS Foundation Trust	0	Myocardial ischaemia	Cardiac disorders	Grade4	Hospitalisation	No relationship
BNT-half	Liverpool University Hospitals NHS Foundation Trust	36	Paraesthesia	Nervous system disorders	Grade2	Hospitalisation	Unlikely

* See protocol for causality assessment guidance

Supplementary Table 7: Baseline vs Day 28 immunogenicity

Summary of immunogenicity at baseline (pre-3rd dose) and fold-rise at 28 days post boost compared with baseline*

	**Prime with ChAd/ChAd**				**Prime with BNT/BNT**			
**Group A**	**Control (N=93)**	**ChAd (N=100)**	**NVX (N=96)**	**NVX-half (N=97)**	**Control (N=111)**	**ChAd (N=98)**	**NVX (N=103)**	**NVX-half (N=99)**
SARS-CoV-2 anti-spike IgG, ELU/ml								
D0	1024 (854,1227) (n=93)	956 (783,1169) (n=100)	1037 (863,1247) (n=96)	1047 (846,1296) (n=97)	3437 (2832,4172) (n=110)	3350 (2763,4061) (n=98)	3081 (2573,3689) (n=103)	4442 (3712,5314) (n=98)
GMFR	0.79 (0.75,0.83) (n=91)	2.58 (2.16,3.08) (n=99)	6.70 (5.59,8.05) (n=95	4.42 (3.70,5.29) (n=97)	0.74 (0.71,0.77) (n=110)	4.06 (3.33,4.94) (n=97)	3.57 (2.98,4.27) (n=101)	1.97 (1.66,2.34) (n=97)
Pseudotype virus neutr alising antibody (WT), NT_{50}								
D0	122 (94,158) (n=48)	95 (75,121) (n=50)	123 (90,167) (n=48)	123 (88,171) (n=50)	191 (144,253) (n=53)	181 (130,253) (n=49)	217 (159,296) (n=49)	273 (208,360) (n=49)
GMFR	0.74 (0.66,0.84) (n=46)	1.79 (1.48,2.17) (n=49)	5.16 (3.77,7.07) (n=44)	4.24 (3.17,5.67) (n=45)	0.91 (0.82,1.00) (n=53)	6.12 (4.25,8.80) (n=49)	3.67 (2.75,4.90) (n=45)	2.74 (1.95,3.84) (n=45)
Pseudotype virus neutr alising antibody (Delta), NT_{50}								
D0	23.6 (16.1,34.5) (n=48)	19.3 (14.1,26.6) (n=50)	26.8 (18.8,38.1) (n=48)	27.1 (19.7,37.4) (n=50)	40.7 (29.1,56.9) (n=53)	45.9 (31.7,66.3) (n=49)	50.6 (37.5,68.3) (n=50)	63.3 (44.2,90.8) (n=49)

GMFR	1.04 (0.80,1.35) (n=46)	1.93 (1.52,2.46) (n=49)	5.64 (3.90,8.16) (n=39)	4.70 (3.23,6.82) (n=37)	0.91 (0.77,1.07) (n=53)	5.68 (3.78,8.52) (n=49)	3.72 (2.62,5.29) (n=40)	2.92 (1.96,4.36) (n=43)
Cellular response (WT), SFC/10^6 PBMCs								
D0	41.4 (30.0,57.1) (n=47)	38.7 (28.1,53.3) (n=48)	24.6 (16.4,37.1) (n=47)	27.2 (19.8,37.3) (n=48)	38.0 (26.5,54.5) (n=53)	48.5 (33.5,70.0) (n=49)	35.2 (24.0,51.4) (n=49)	26.1 (18.1,37.5) (n=48)
GMFR	1.11 (0.84,1.47) (n=44)	1.24 (0.98,1.58) (n=46)	4.88 (3.25,7.35) (n=44)	3.47 (2.61,4.61) (n=46)	0.88 (0.59,1.31) (n=52)	2.11 (1.56,2.84) (n=47)	1.62 (1.14,2.28) (n=48)	1.36 (0.98,1.89) (n=47)

**Group B**	**Control (N=93)**	**BNT (N=95)**	**VLA (N=95)**	**VLA-half (N=107)**	**Ad26 (N=101)**	**Control (N=97)**	**BNT (N=96)**	**VLA (N=99)**	**VLA-half (N=98)**	**Ad26 (N=89)**
SARS-CoV-2 anti-spike IgG, ELU/ml										
D0	1162 (972,1389) (n=92)	1286 (1076,1537) (n=94)	1303 (1062,1598) (n=95)	1186 (982,1432) (n=107)	1495 (1203,1858) (n=101)	4283 (3608,5083) (n=97)	4768 (3966,5732) (n=96)	4349 (3569,5299) (n=99)	3902 (3275,4649) (n=98)	4018 (3297,4898) (n=89)
GMFR	0.67 (0.58,0.78) (n=90)	15.96 (13.75,18.53) (n=93)	1.45 (1.31,1.60) (n=93)	1.22 (1.10,1.35) (n=103)	3.63 (3.04,4.33) (n=98)	0.73 (0.68,0.78) (n=94)	5.71 (4.86,6.72) (n=96)	0.96 (0.84,1.11) (n=98)	0.95 (0.84,1.09) (n=98)	4.35 (3.57,5.30) (n=87)
Pseudotype virus neutr alising antibody (WT), NT_{50}										
D0	88 (71,110) (n=48)	101 (80,127) (n=49)	125 (98,160) (n=48)	118 (92,150) (n=54)	142 (107,190) (n=54)	238 (186,305) (n=53)	257 (195,340) (n=49)	283 (218,367) (n=48)	258 (211,314) (n=50)	283 (203,394) (n=44)
GMFR	0.81 (0.72,0.90) (n=48)	15.40 (12.40,19.13) (n=49)	1.80 (1.48,2.20) (n=48)	1.17 (1.03,1.33) (n=51)	4.33 (3.18,5.89) (n=51)	0.82 (0.74,0.92) (n=50)	6.54 (5.11,8.37) (n=49)	1.09 (0.91,1.31) (n=44)	0.92 (0.81,1.04) (n=45)	6.61 (4.54,9.61) (n=35)
Pseudotype virus neutr alising antibody (Delta), NT_{50}										

D0	20.4 (15.0,27.7) (n=49	28.4 (21.6,37.4) (n=51)	24.7 (17.9,34.1) (n=48)	22.5 (17.2,29.4) (n=54)	27.3 (19.7,37.8) (n=54)	77.6 (59.8,100.6) (n=53)	70.9 (52.7,95.4) (n=49)	70.8 (52.1,96.1) (n=47)	52.8 (38.9,71.7) (n=50)	60.3 (42,86.6) (n=45)
GMFR	0.98 (0.82,1.17) (n=49)	11.65 (8.56,15.84) (n=51)	1.64 (1.38,1.94) (n=44)	1.27 (0.98,1.64) (n=46)	5.21 (3.92,6.94) (n=49)	0.66 (0.53,0.82) (n=49)	4.89 (3.50,6.83) (n=49)	1.08 (0.89,1.32) (n=45)	1.04 (0.88,1.23) (n=48)	7.37 (5.08,10.7) (n=40)
Cellular response (WT), SFC/10^6 PBMCs										
D0	42.4 (30.1,59.7) (n=49)	37.2 (25.9,53.4) (n=48)	31.6 (21.4,46.7) (n=48)	35.9 (26.2,49.1) (n=54)	37.0 (27.7,49.4) (n=53)	27.8 (20.5,37.6) (n=52)	29.6 (20.9,41.8) (n=51)	27.7 (19.5,39.2) (n=49)	33.8 (24.5,46.7) (n=51)	39.0 (26.4,57.6) (n=42)
GMFR	1.00 (0.73,1.39) (n=49)	3.40 (2.31,4.99) (n=48)	1.62 (1.11,2.35) (n=47)	1.52 (1.06,2.17) (n=52)	2.86 (2.15,3.81) (n=53)	1.13 (0.83,1.55) (n=49)	2.77 (2.12,3.62) (n=49)	1.18 (0.88,1.58) (n=49)	1.12 (0.80,1.58) (n=51)	2.77 (1.85,4.15) (n=42)

**Group C**	**Control (N=102)**	**BNT-half (N=105)**	**M1273 (N=98)**	**CVn (N=105)**	**Control (N=100)**	**BNT-half (N=94)**	**m1273 (N=92)**	**CVn (N=94)**
SARS-CoV-2 anti-spike IgG, ELU/ml								
D0	1117 (932,1338) (n=100)	1348 (1104,1647) (n=105)	1353 (1128,1623) (n=97)	999 (844,1183) (n=104)	4100 (3437,4891) (n=100)	4099 (3334,5041) (n=92)	3442 (2775,4268) (n=92)	4192 (3529,4979) (n=93)
GMFR	0.77 (0.70,0.85) (n=99)	11.79 (9.70,14.33) (n=103)	23.37 (20.01,27.30) (n=96)	4.08 (3.45,4.82) (n=102)	0.74 (0.69,0.79) (n=98)	5.64 (4.63,6.88) (n=90)	9.86 (8.15,11.93) (n=91)	1.82 (1.59,2.08) (n=90)
Pseudotype virus neutr alising antibody (WT), NT_{50}								
D0	98 (71,136) (n=48)	123 (89,171) (n=52)	117 (81,169) (n=45)	118 (91,153) (n=48)	191 (146,250) (n=49)	238 (167,339) (n=47)	168 (118,237) (n=45)	297 (236,373) (n=47)
GMFR	0.76 (0.57,1.02) (n=47)	10.43 (8.08,13.46) (n=50)	22.28 (17.16,28.91) (n=44)	3.34 (2.44,4.59) (n=43)	0.82 (0.68,0.98) (n=47)	5.93 (4.32,8.14) (n=45)	9.27 (7.04,12.19) (n=45)	1.88 (1.55,2.28) (n=44)

Pseudotype virus neutralising antibody (Delta), NT_{50}								
D0	21.2 (15.3,29.2) (n=48)	33.0 (23.4,46.6) (n=52)	28.6 (21.1,38.7) (n=45)	21.9 (16.8,28.5) (n=48)	56.9 (43.5,74.4) (n=49)	63.7 (42.9,94.5) (n=47)	48.6 (34.2,69.0) (n=45)	79.1 (57.8,108.2) (n=47)
GMFR	0.71 (0.61,0.82) (n=47)	9.00 (7.02,11.55) (n=50)	19.84 (15.19,25.90) (n=43)	3.14 (2.31,4.27) (n=40)	0.63 (0.54,0.73) (n=47)	5.10 (3.66,7.11) (n=44)	8.08 (5.98,10.91) (n=45)	1.61 (1.28,2.04) (n=43)
Cellular response (WT), SFC/10^6 PBMCs								
D0	43.1 (29.9,62.3) (n=48)	48.4 (35,66.9) (n=54)	47.2 (33.4,66.7) (n=44)	31.0 (22.1,43.5) (n=48)	38.3 (27.4,53.6) (n=48)	43.3 (30.2,62.2) (n=45)	38.7 (26.5,56.5) (n=45)	37.7 (26.6,53.6) (n=45)
GMFR	0.89 (0.66,1.19) (n=48)	2.80 (2.17,3.62) (n=52)	3.05 (2.07,4.48) (n=42)	1.57 (1.10,2.25) (n=48)	0.58 (0.42,0.80) (n=46)	1.70 (1.20,2.39) (n=43)	2.90 (2.05,4.10) (n=44)	1.26 (0.93,1.70) (n=43)

* Data shown are geometric mean (95% CI); GMFR=geometric mean of fold rise; Control =quadrivalent meningococcal conjugate vaccine; ChAd=ChAdOx1 nCoV-19 vaccine, Oxford–AstraZeneca; BNT=BNT162b2 vaccine (Pfizer–BioNTech); NVX=NVX-CoV2373 vaccine, Novavax; NVX half=half dose of NVX-CoV2373 vaccine; VLA=VLA2001 vaccine, Valneva; VLA half=half dose of VLA2001 vaccine; Ad26=Ad26.COV2.S vaccine, Janssen; BNT half=half dose of BNT162b2 vaccine; m1273=mRNA1273 vaccine, Moderna; CVn=CVnCoV vaccine, Curevac.

Supplementary Table 8: Baseline immunogenicity by Serostatus (anti-nucleocapsid IgG)

Summary of immunogenicity at baseline (pre-3rd dose) between seropositive and seronegative participants*

	**Prime with ChAd/ChAd**		**Prime with BNT/BNT**	
	**Seropositive N=143**	**Seronegative N=1303**	**Seropositive N=152**	**Seronegative N=1285**
SARS-CoV-2 anti-spike IgG, ELU/ml	7519 (95%CI: 6188-9137) [n=140]	1160 (95%CI: 1100-1224) [n=1294]	16077 (95%CI: 13992-18473) [n=150]	3900 (95%CI: 3701-4110) [n=1279]
Pseudotype virus neutralising antibody (WT), NT_{50}	1088 (95%CI: 786-1504) [n=69]	113 (95%CI: 105-123) [n=652]	1367 (95%CI: 1038-1801) [n=76]	231 (95%CI: 213-250) [n=640]
Live virus neutralising antibody, normalised NT_{80}	1395 (95%CI: 900-2164) [n=32]	262 (95%CI: 239-288) [n=317]	3041 (95%CI: 2380-3885) [n=30]	690 (95%CI: 630-756) [n=342]
Cellular response (WT), SFC/10^6 PBMCs	148 (95%CI: 110-199) [n=71]	36 (95%CI: 33-40) [n=645]	164 (95%CI: 132-203) [n=77]	35 (95%CI: 31-38) [n=634]

* Data shown are geometric mean (95% CI).

Supplementary Figures

Supplementary Figure 1: Solicited systemic and local reactions (Bar chart)

Severity of solicited local and systemic reactions in days 0-7 following 3rd dose vaccination by study arm, priming vaccine schedule and age group, as self-reported in participant electronic diaries, A1) Group A local; A2) Group A systemic; B1) Group B local; B2) Group B systemic; C1) Group C local; C2) Group C systemic.

The severity presented is the participant's highest severity across 7 days post vaccination for each solicited AEs. The details of grading can be found in the protocol section 13.4 "Assessment of severity". Control A/B/C=quadrivalent meningococcal conjugate vaccine; ChAd=ChAdOx1 nCoV-19 vaccine, Oxford–AstraZeneca; NVX=NVX-CoV2373 vaccine, Novavax; NVX half=half dose of NVX-CoV2373 vaccine; BNT=BNT162b2 vaccine (Pfizer–BioNTech); VLA=VLA2001 vaccine, Valneva; VLA half=half dose of VLA2001 vaccine; Ad26=Ad26.COV2.S vaccine, Janssen; BNT half=half dose of BNT162b2 vaccine; MOD=mRNA1273 vaccine, Moderna; CVn=CVnCoV vaccine, Curevac.

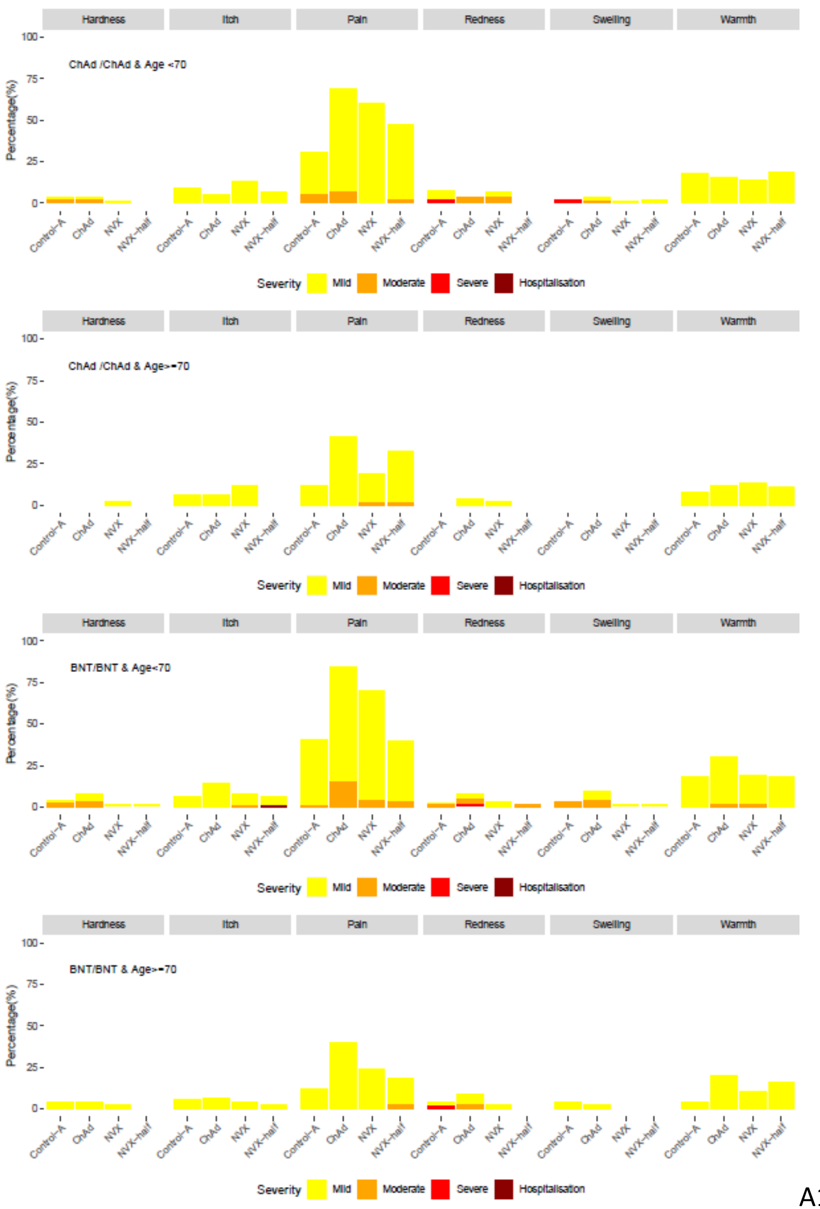
Hardness
Itch
Pain
Redness
Swelling
Warmth
ChAd /ChAd & Age <70
ChAd /ChAd & Age>=70
BNT/BNT & Age<70
BNT/BNT & Age>=70
Percentage(%)
Control-A
ChAd
NVX
NVX-half
Severity
Mild
Moderate
Severe
Hospitalisation

A1)

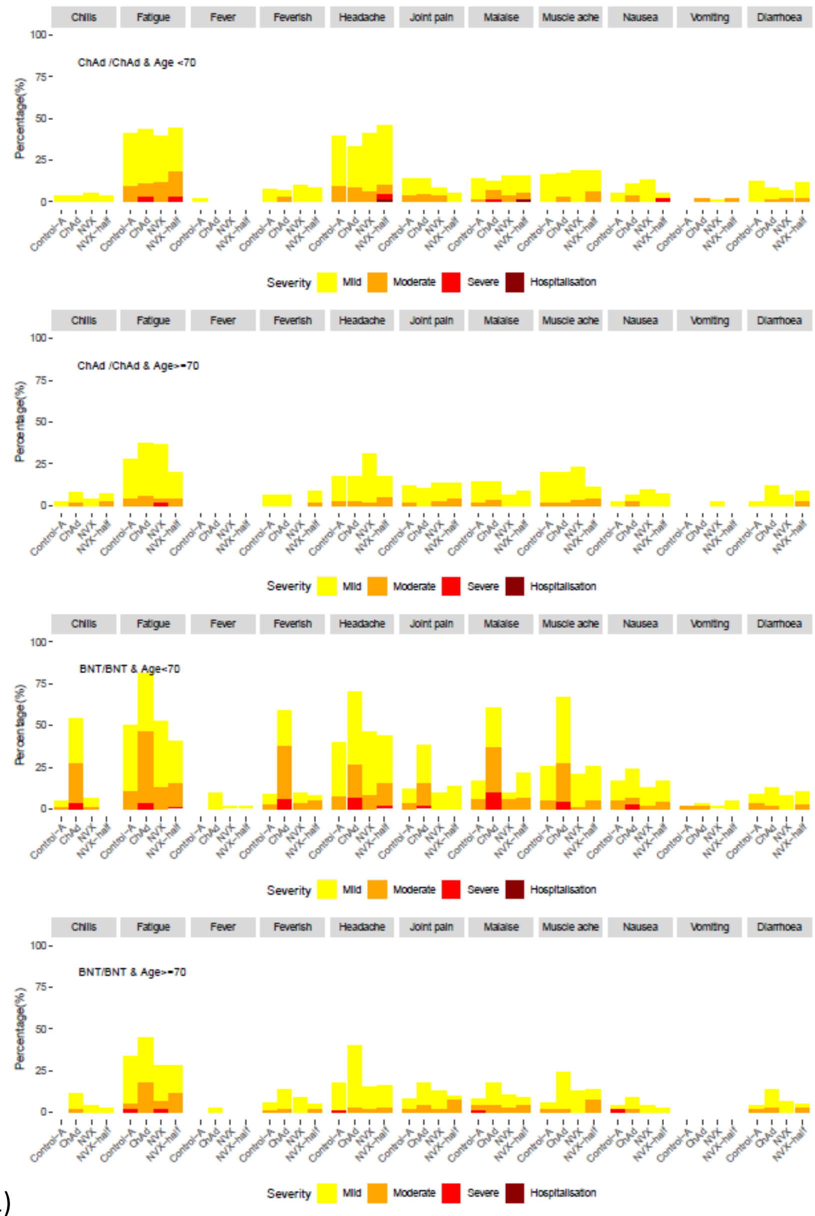
Chills
Fatigue
Fever
Feverish
Headache
Joint pain
Malaise
Muscle ache
Nausea
Vomiting
Diarrhoea
ChAd /ChAd & Age <70
ChAd /ChAd & Age>=70
BNT/BNT & Age<70
BNT/BNT & Age>=70
Percentage(%)
Control-A
ChAd
NVX
NVX-half
Severity
Mild
Moderate
Severe
Hospitalisation

A2)

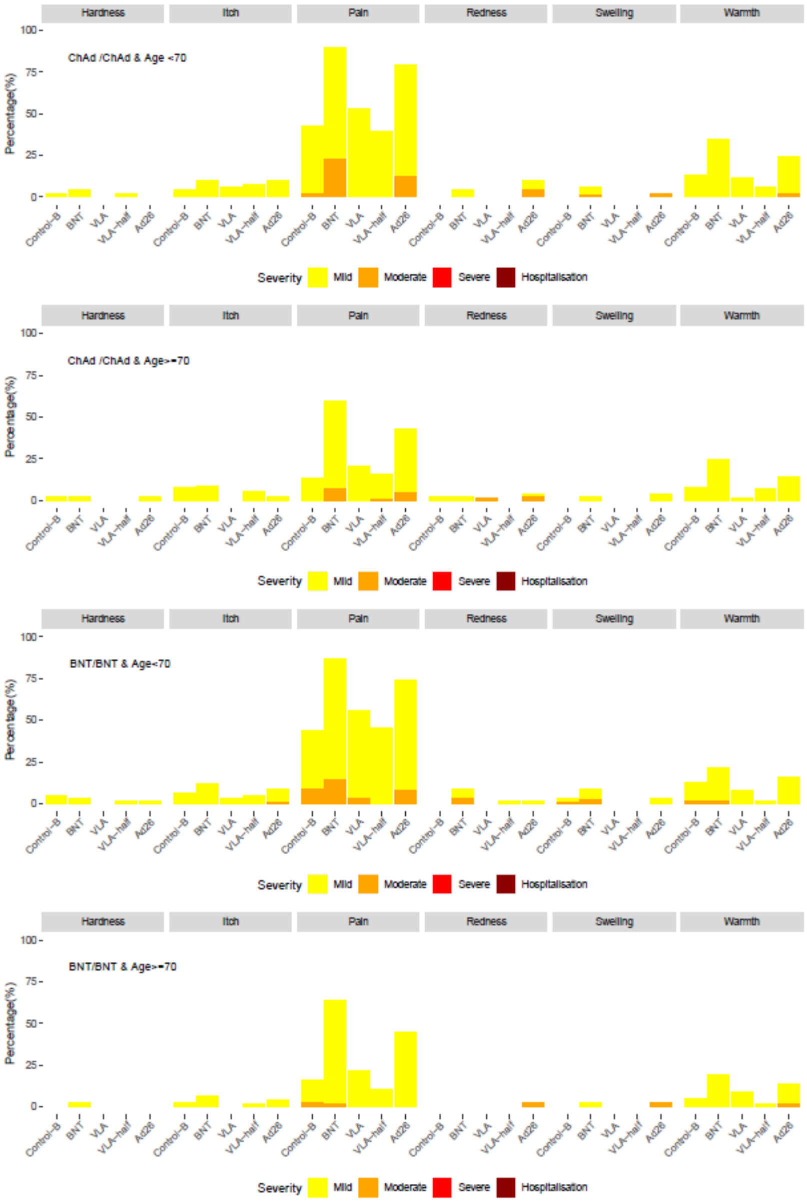

Hardness
Itch
Pain
Redness
Swelling
Warmth
ChAd /ChAd & Age <70
ChAd /ChAd & Age>=70
BNT/BNT & Age<70
BNT/BNT & Age>=70
Percentage(%)
100
75
50
25
0
Control-B
BNT
VLA
VLA-half
Ad26
Severity
Mild
Moderate
Severe
Hospitalisation

B1)

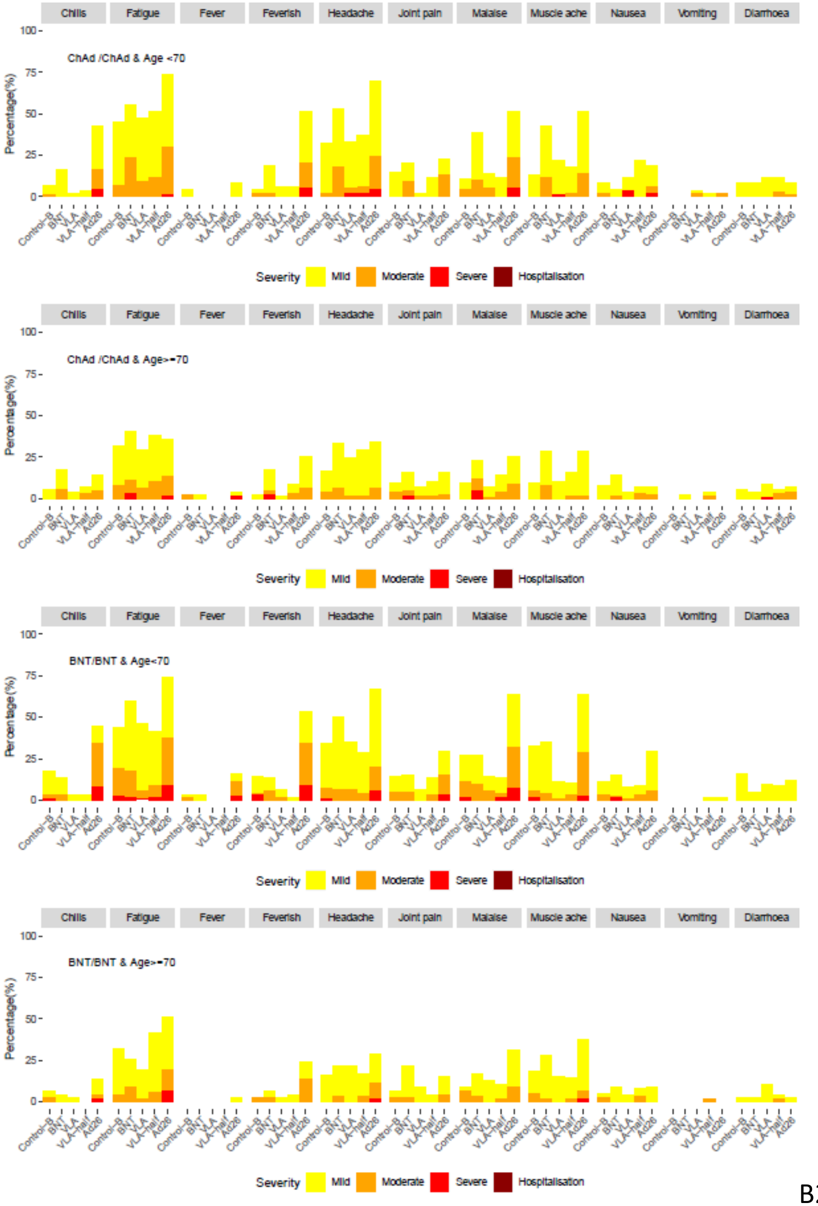

Chills
Fatigue
Fever
Feverish
Headache
Joint pain
Malaise
Muscle ache
Nausea
Vomiting
Diarrhoea
ChAd /ChAd & Age <70
ChAd /ChAd & Age>=70
BNT/BNT & Age<70
BNT/BNT & Age>=70
Percentage(%)
100
75
50
25
0
Control-B
BNT
VLA
VLA-half
Ad26
Severity
Mild
Moderate
Severe
Hospitalisation

B2)

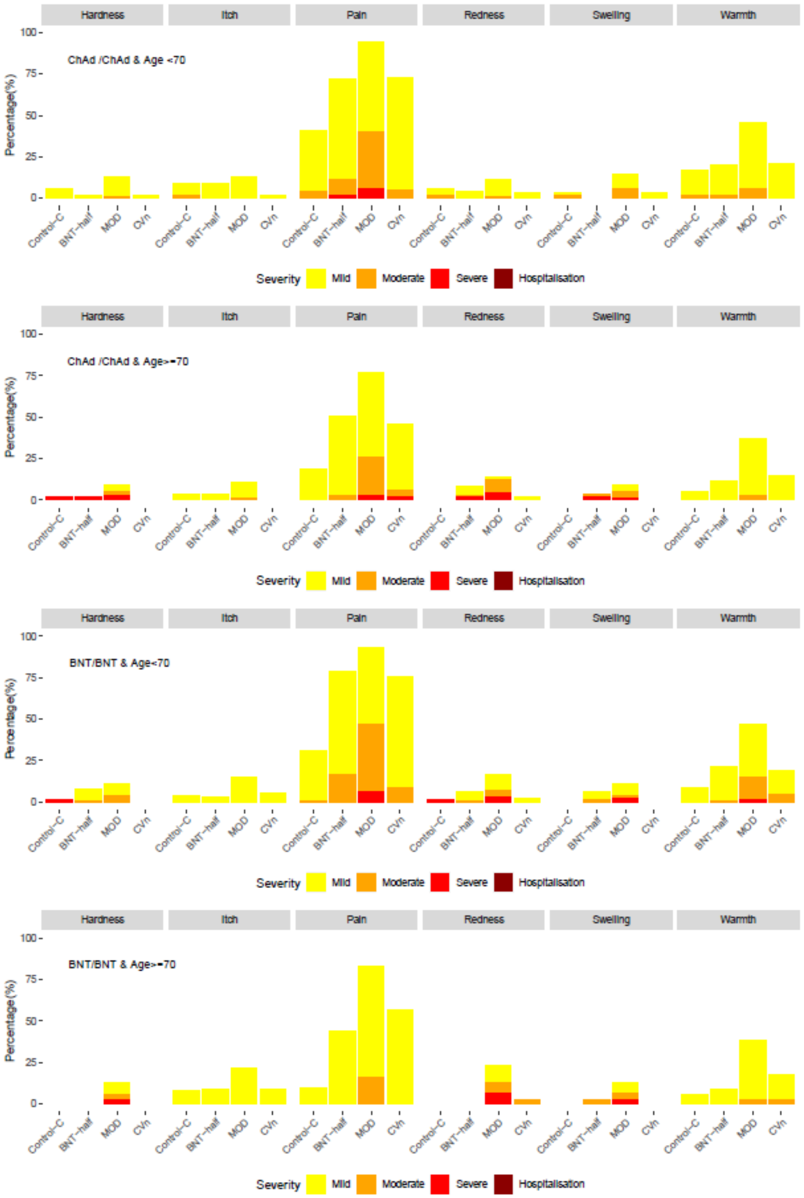
Hardness
Itch
Pain
Redness
Swelling
Warmth
ChAd /ChAd & Age <70
ChAd /ChAd & Age>=70
BNT/BNT & Age<70
BNT/BNT & Age>=70
Percentage(%)
100
75
50
25
0
Control-C
BNT-half
MOD
CVn
Severity
Mild
Moderate
Severe
Hospitalisation

C1)

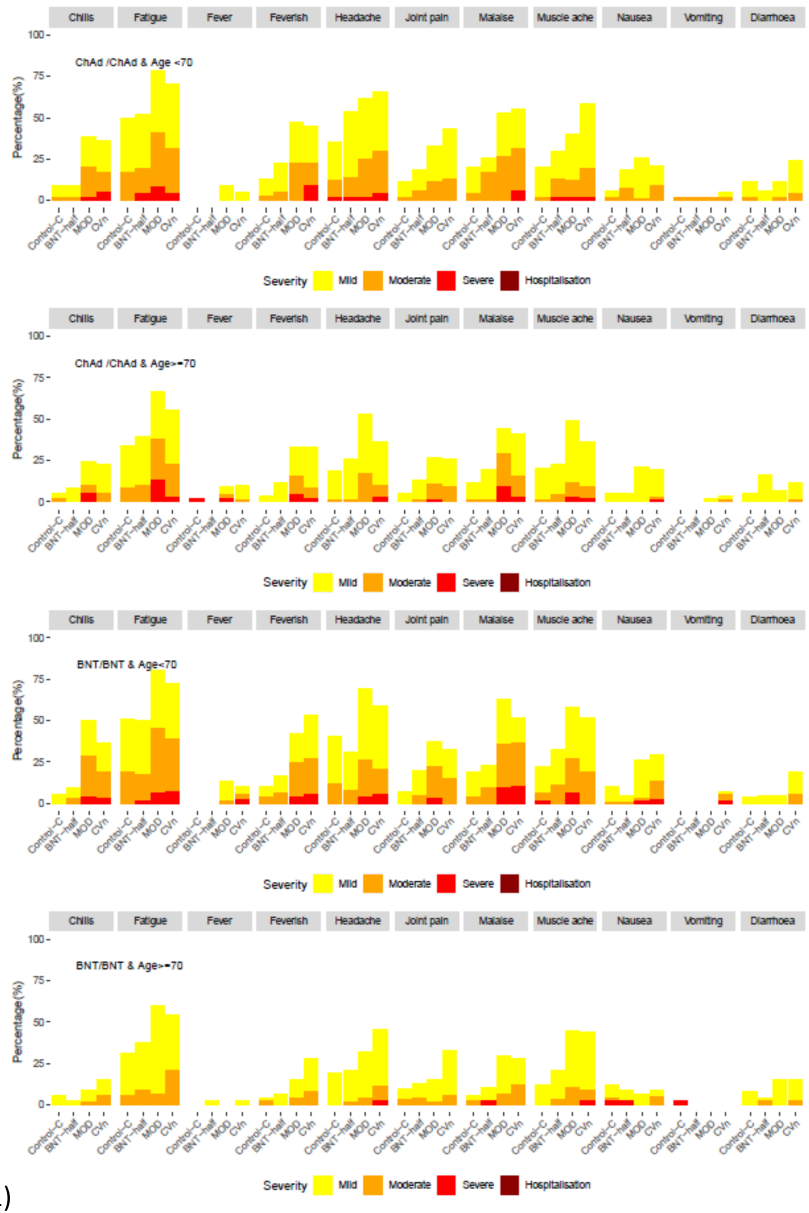
Chills
Fatigue
Fever
Feverish
Headache
Joint pain
Malaise
Muscle ache
Nausea
Vomiting
Diarrhoea
ChAd /ChAd & Age <70
ChAd /ChAd & Age>=70
BNT/BNT & Age<70
BNT/BNT & Age>=70
Percentage(%)
100
75
50
25
0
Control-C
BNT-half
MOD
CVn
Severity
Mild
Moderate
Severe
Hospitalisation

C2)

Supplementary Figure 2: Severity of solicited reactions (Radial chart)

Severity of solicited local and systemic reactions in days 0-7 following 3rd dose vaccination by study arm as self-reported in participant electronic diaries, A1) Group A severe; A2) Group A moderate; B1) Group B severe; B2) Group B moderate; C1) Group C severe; C2) Group C moderate

Data presented on each spike axis is the proportion of severe (A1, B1, C1) and moderate (A2, B2, C2) local and solicited AEs among the participants with self-reported diary data. For each AE, we used the maximum severity recorded across 7 days post vaccination. Area defined by each vaccine provides a visual representation of reactogenicity burden, where greater area indicates greater burden. The details of grading can be found in the protocol section 13.4 “Assessment of severity”. Control A/B/C=quadrivalent meningococcal conjugate vaccine; ChAd=ChAdOx1 nCoV-19 vaccine, Oxford–AstraZeneca; NVX=NVX-CoV2373 vaccine, Novavax; NVX half=half dose of NVX-CoV2373 vaccine; BNT=BNT162b2 vaccine (Pfizer–BioNTech); VLA=VLA2001 vaccine, Valneva; VLA half=half dose of VLA2001 vaccine; Ad26=Ad26.COV2.S vaccine, Janssen; BNT half=half dose of BNT162b2 vaccine; MOD=mRNA1273 vaccine, Moderna; CVn=CVnCoV vaccine, Curevac.

Supplementary Figure 2.A1 Group A severe

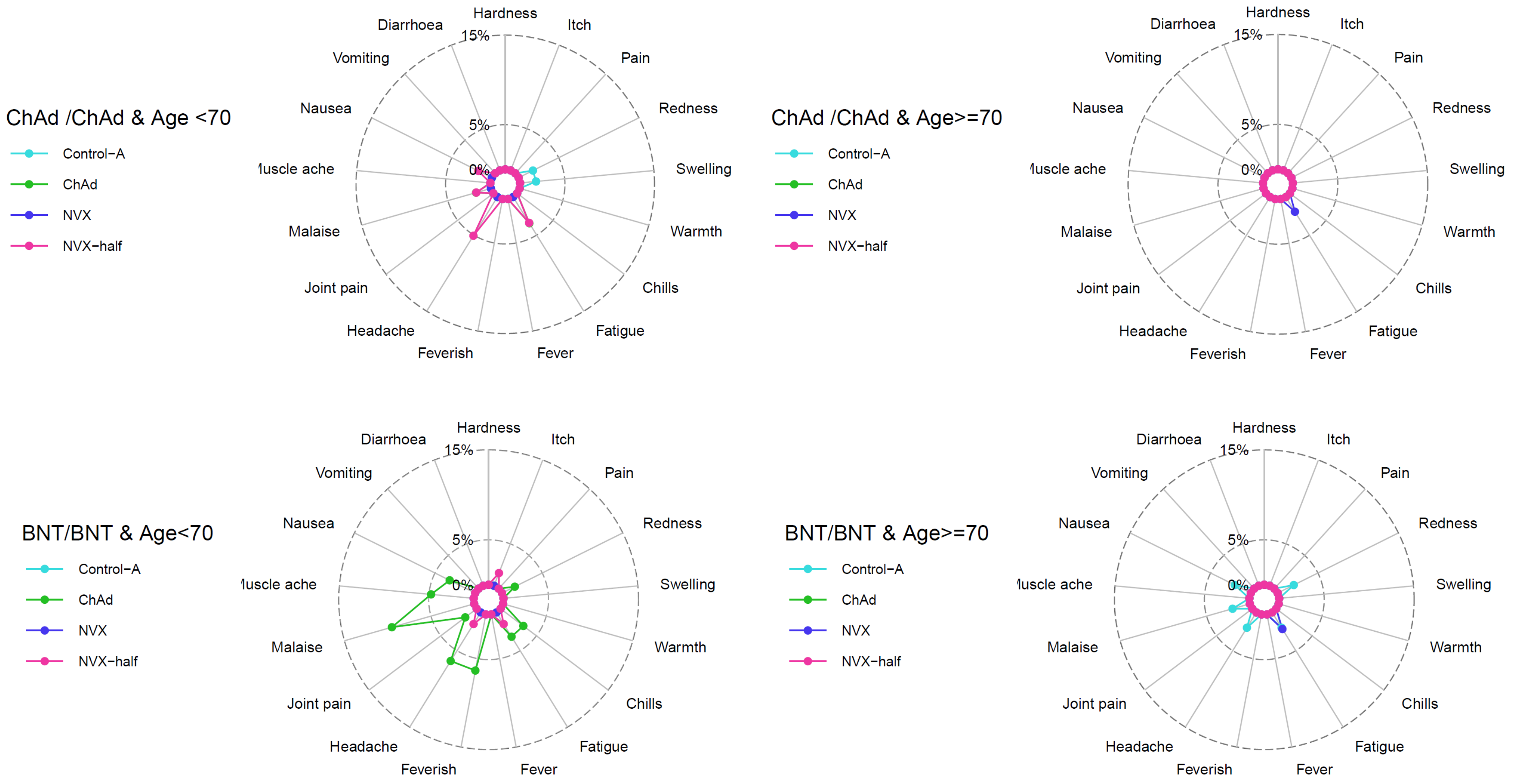

Supplementary Figure 2.A2 Group A moderate and severe

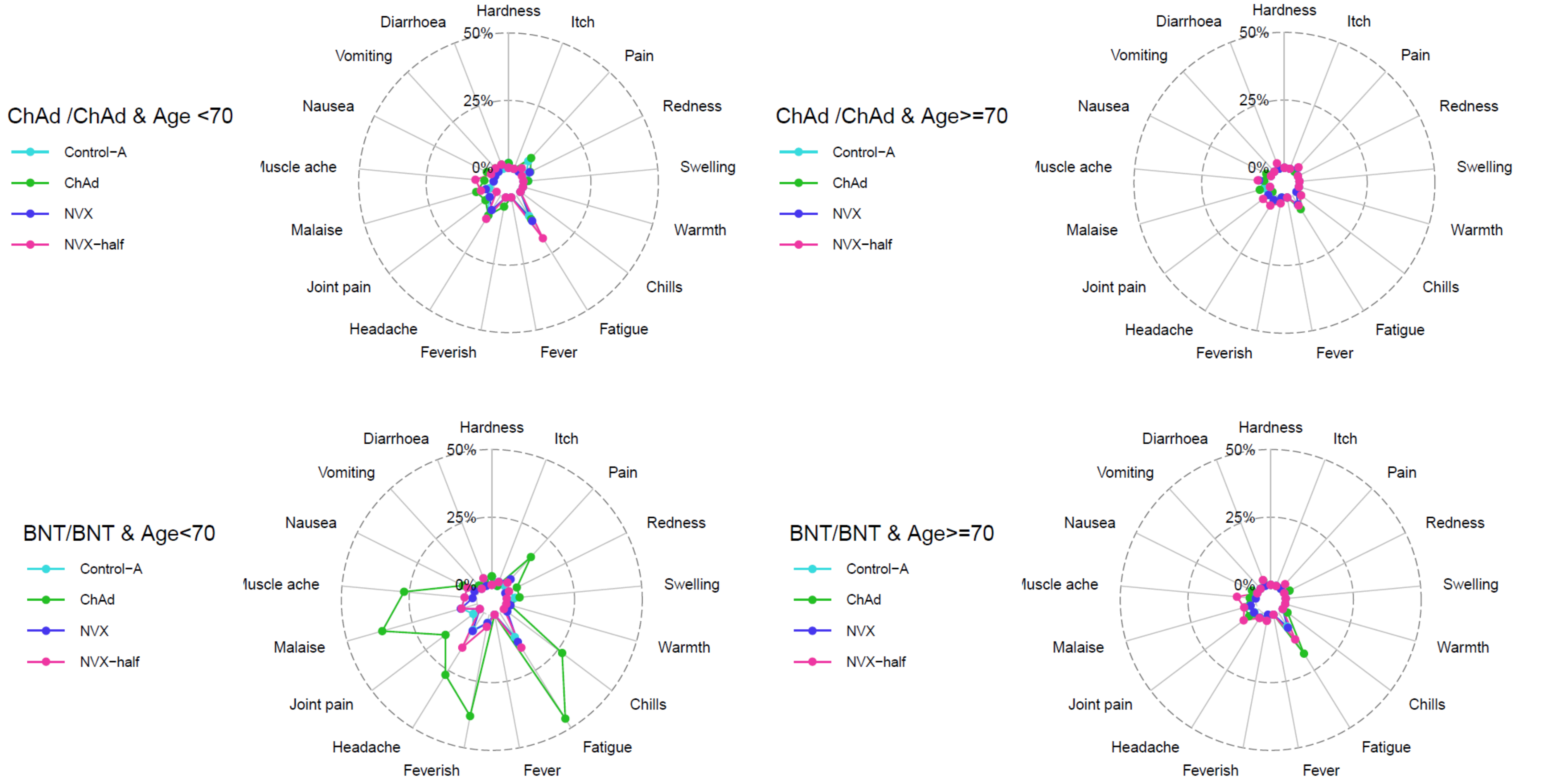

Supplementary Figure 2.B1 Group B severe

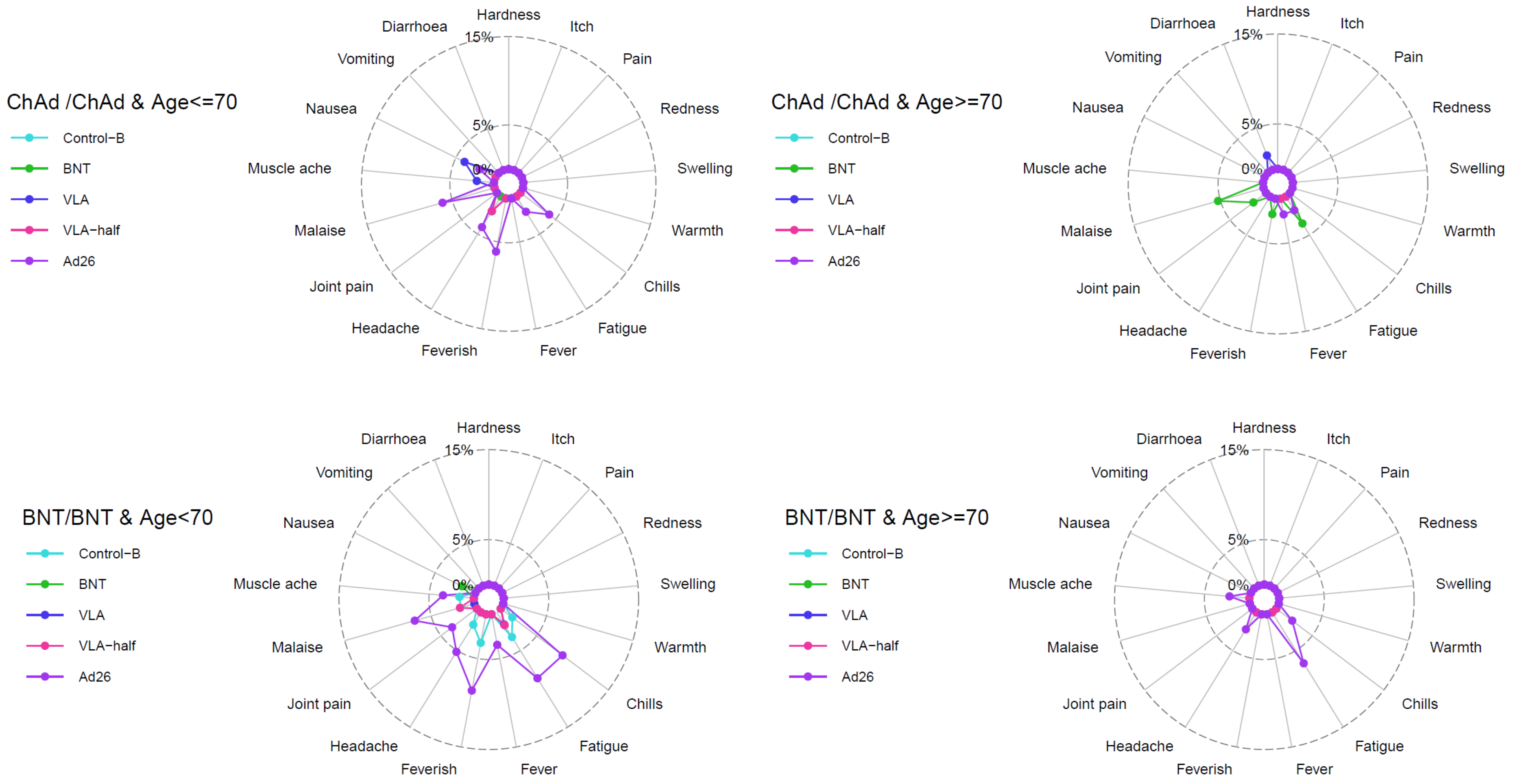

Supplementary Figure 2.B2 Group B moderate and severe

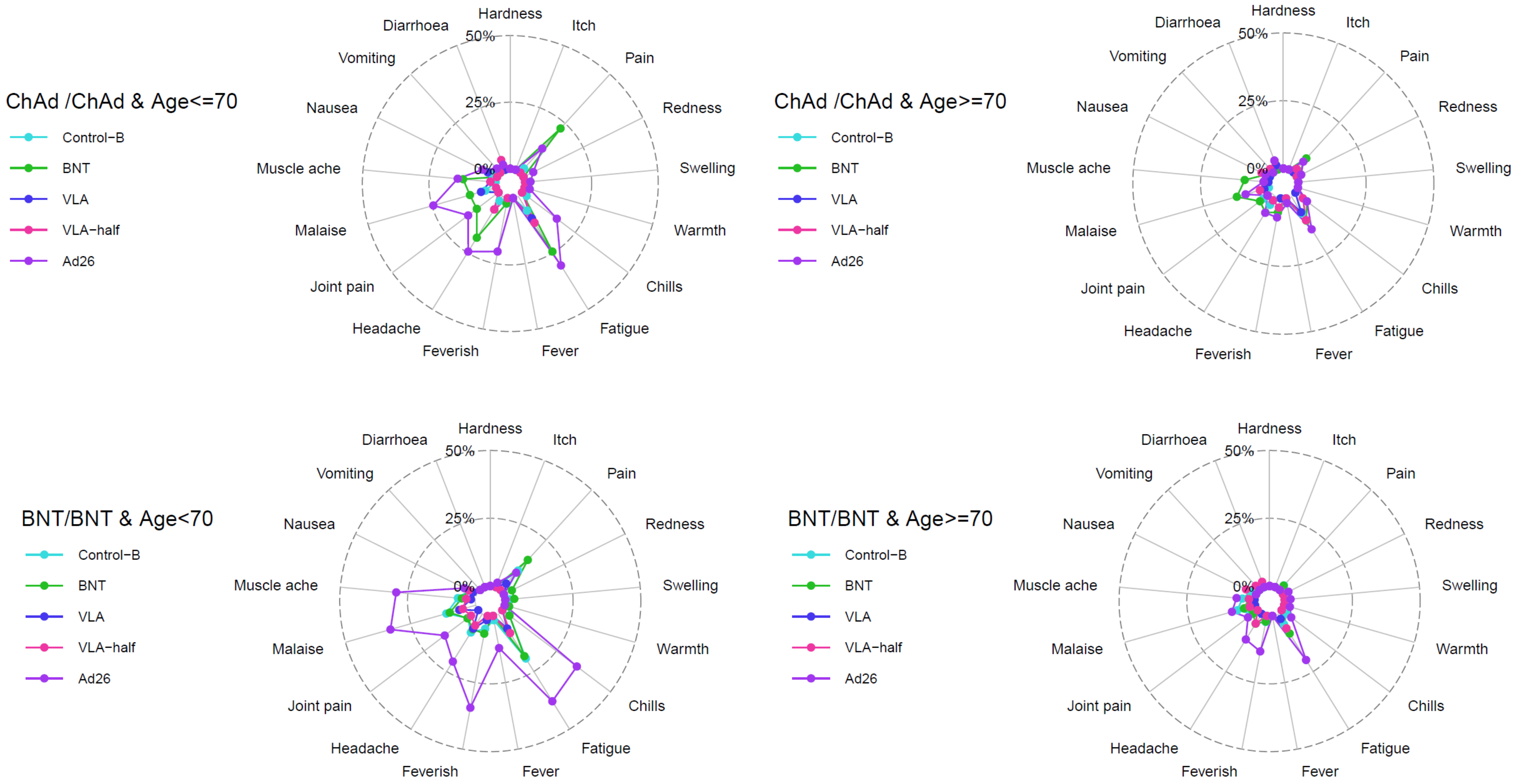

Supplementary Figure 2.C1 Group C severe

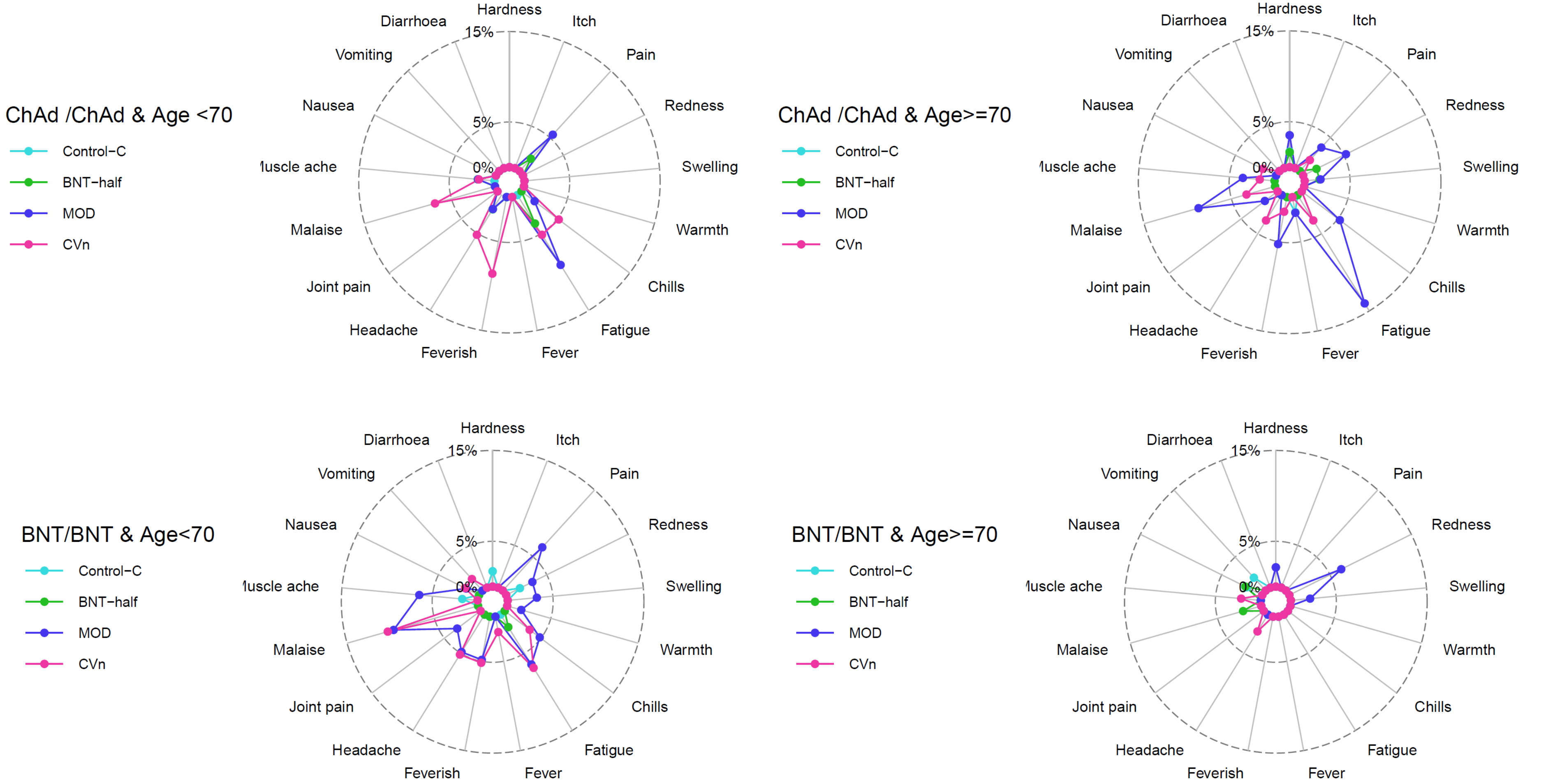

Supplementary Figure 2.C2 Group C moderate and severe

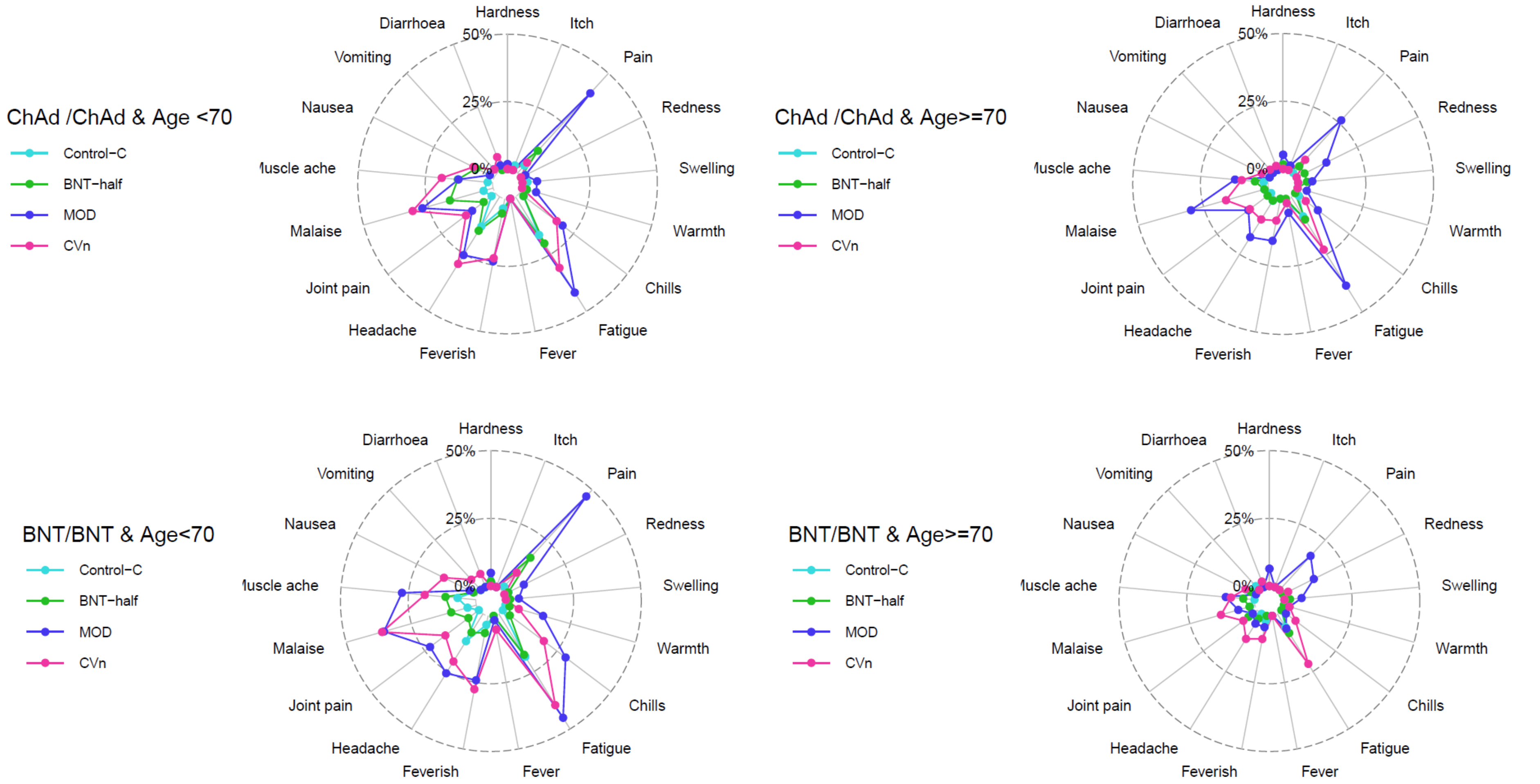

Supplementary Figure 3: Kinetics of immunogenicity

A) Anti-spike IgG; B) T cell response, by prime vaccine schedules among the immunology cohort
Control A/B/C=quadrivalent meningococcal conjugate vaccine; ChAd=ChAdOx1 nCoV-19 vaccine, Oxford–AstraZeneca; NVX=NVX-CoV2373 vaccine, Novavax; NVX half=half dose of NVX-CoV2373 vaccine; BNT=BNT162b2 vaccine (Pfizer–BioNTech); VLA=VLA2001 vaccine, Valneva; VLA half=half dose of VLA2001 vaccine; Ad26=Ad26.COV2.S vaccine, Janssen; BNT half=half dose of BNT162b2 vaccine; MOD=mRNA1273 vaccine, Moderna; CVn=CVnCoV vaccine, Curevac.

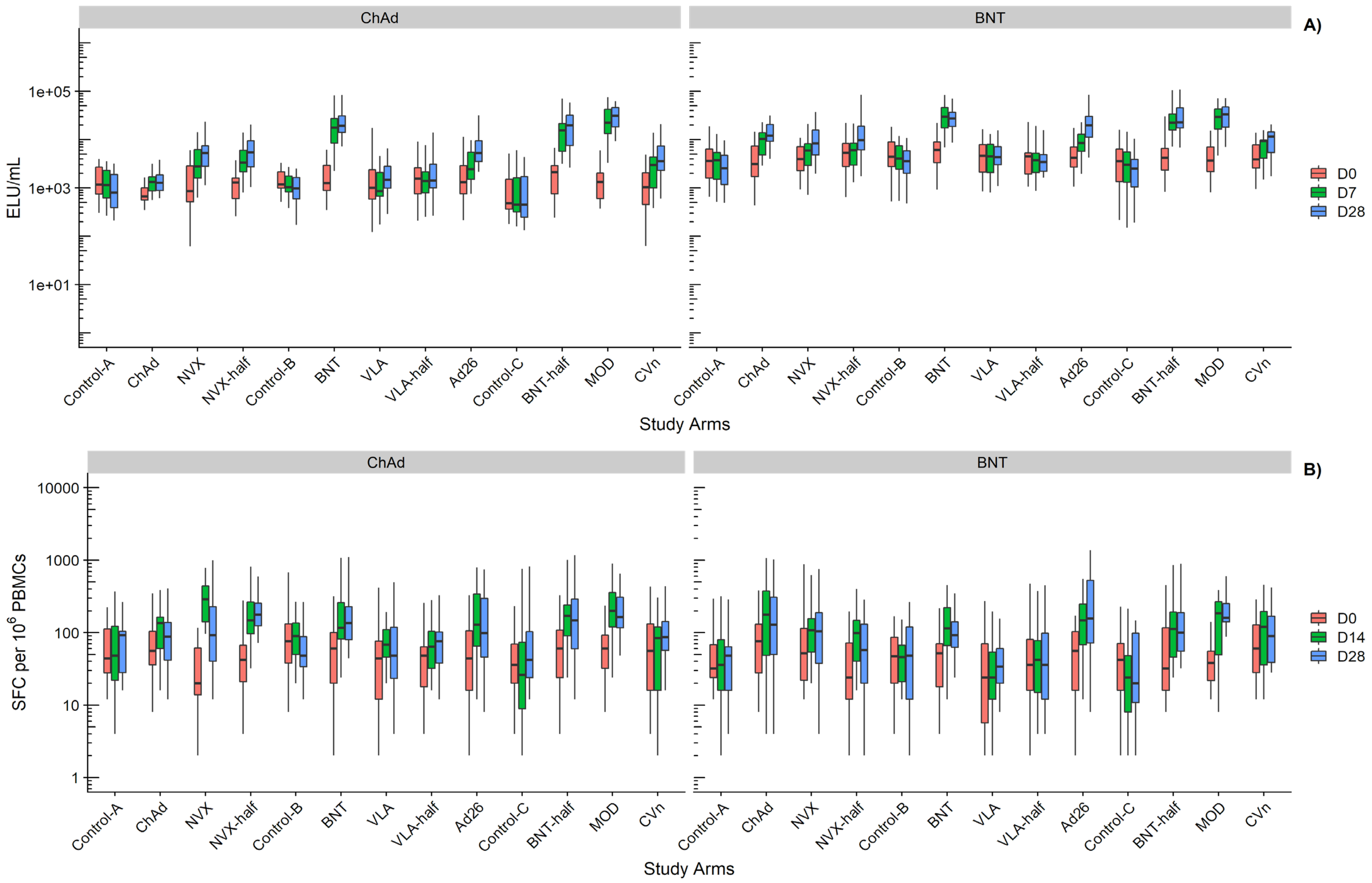
A)
ChAd
BNT
ELU/mL
1e+05
1e+03
1e+01
Control-A
ChAd
NVX
NVX-half
Control-B
BNT
VLA
VLA-half
Ad26
Control-C
BNT-half
MOD
CVn
Study Arms
D0
D7
D28
B)
SFC per 10^6 PBMCs
10000
1000
100
10
1
D0
D14
D28

Supplementary Figure 4: Correlation between PNA for WT and Delta

Correlation between PNA against WT and PNA against Delta strain at 28 days post 3rd dose in participants with A) ChAd/ChAd prime in Group A; B) BNT/BNT prime in Group A; C) ChAd/ChAd prime in Group B; D) BNT/BNT prime in Group B; E) ChAd/ChAd prime in Group C; F) BNT/BNT prime in Group C.

The dotted diagonal line shows the situation when the immunogenicity to a variant of concern (VOC) is the same as that to the WT (the vaccines were designed for). Ellipses show the 95% CIs for different vaccine schedules, assuming multivariate normal distributions. NT50=50% neutralising antibody titre. Control A/B/C=quadrivalent meningococcal conjugate vaccine; ChAd=ChAdOx1 nCoV-19 vaccine, Oxford–AstraZeneca; NVX=NVX-CoV2373 vaccine, Novavax; NVX half=half dose of NVX-CoV2373 vaccine; BNT=BNT162b2 vaccine (Pfizer–BioNTech); VLA=VLA2001 vaccine, Valneva; VLA half=half dose of VLA2001 vaccine; Ad26=Ad26.COV2.S vaccine, Janssen; BNT half=half dose of BNT162b2 vaccine; MOD=mRNA1273 vaccine, Moderna; CVn=CVnCoV vaccine, Curevac.

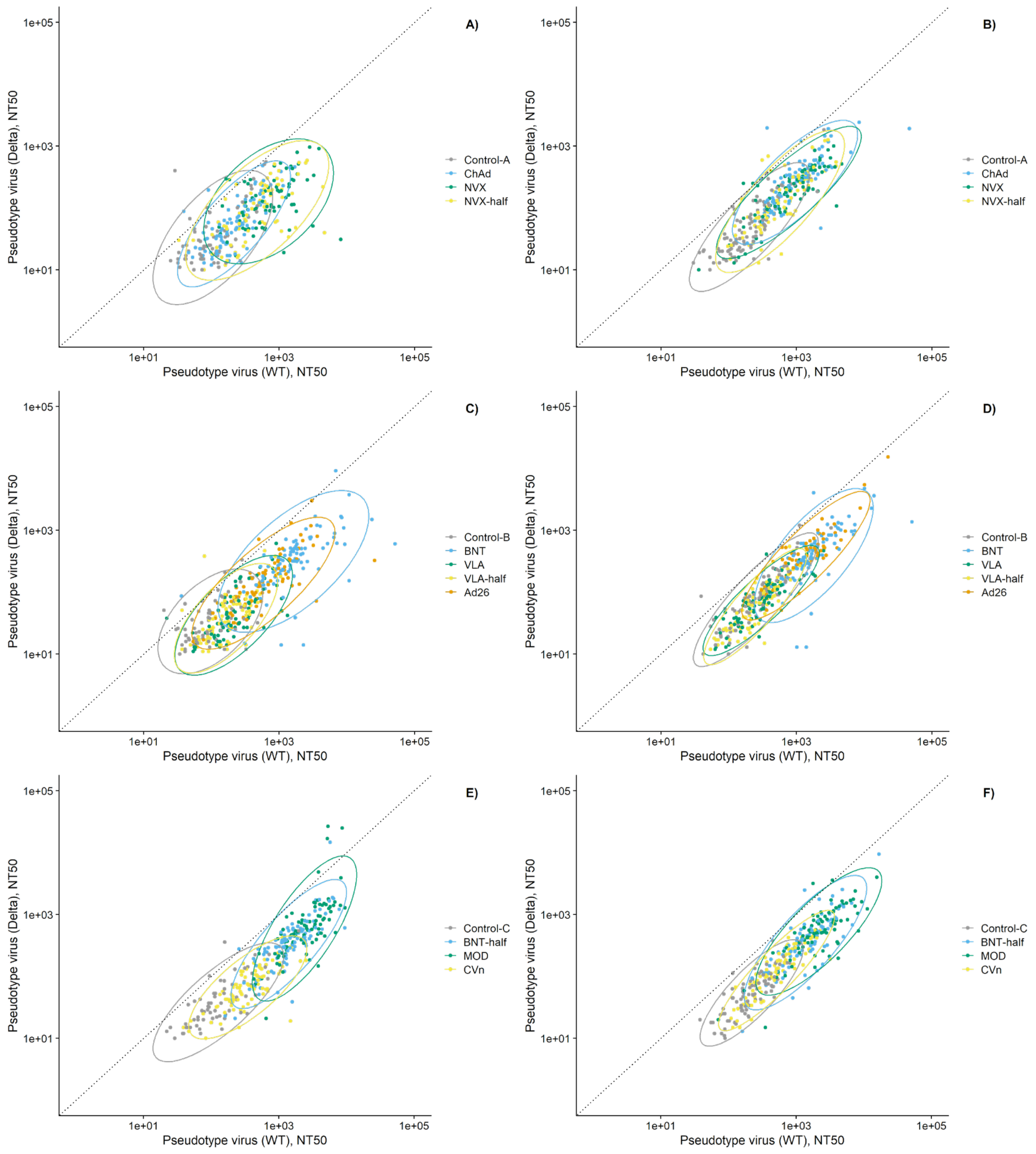
A)
Pseudotype virus (Delta), NT50
1e+05
1e+03
1e+01
Pseudotype virus (WT), NT50
1e+01
1e+03
1e+05
Control-A
ChAd
NVX
NVX-half
B)
Pseudotype virus (Delta), NT50
1e+05
1e+03
1e+01
Pseudotype virus (WT), NT50
1e+01
1e+03
1e+05
Control-A
ChAd
NVX
NVX-half
C)
Pseudotype virus (Delta), NT50
1e+05
1e+03
1e+01
Pseudotype virus (WT), NT50
1e+01
1e+03
1e+05
Control-B
BNT
VLA
VLA-half
Ad26
D)
Pseudotype virus (Delta), NT50
1e+05
1e+03
1e+01
Pseudotype virus (WT), NT50
1e+01
1e+03
1e+05
Control-B
BNT
VLA
VLA-half
Ad26
E)
Pseudotype virus (Delta), NT50
1e+05
1e+03
1e+01
Pseudotype virus (WT), NT50
1e+01
1e+03
1e+05
Control-C
BNT-half
MOD
CVn
F)
Pseudotype virus (Delta), NT50
1e+05
1e+03
1e+01
Pseudotype virus (WT), NT50
1e+01
1e+03
1e+05
Control-C
BNT-half
MOD
CVn

Supplementary Figure 5: Correlation between cellular response for WT and Beta

Correlation between cellular response against WT and cellular response against Beta strain at 28 days post 3rd dose in participants with A) ChAd/ChAd prime in Group A; B) BNT/BNT prime in Group A; C) ChAd/ChAd prime in Group B; D) BNT/BNT prime in Group B; E) ChAd/ChAd prime in Group C; F) BNT/BNT prime in Group C.

The dotted diagonal line shows the situation when the immunogenicity to a variant of concern (VOC) is the same as that to the WT (the vaccines were designed for). Ellipses show the 95% CIs for different vaccine schedules, assuming multivariate normal distributions. PBMC=peripheral blood mononuclear cell. SFC=spot-forming units. Control A/B/C=quadrivalent meningococcal conjugate vaccine; ChAd=ChAdOx1 nCoV-19 vaccine, Oxford–AstraZeneca; NVX=NVX-CoV2373 vaccine, Novavax; NVX half=half dose of NVX-CoV2373 vaccine; BNT=BNT162b2 vaccine (Pfizer–BioNTech); VLA=VLA2001 vaccine, Valneva; VLA half=half dose of VLA2001 vaccine; Ad26=Ad26.COV2.S vaccine, Janssen; BNT half=half dose of BNT162b2 vaccine; MOD=mRNA1273 vaccine, Moderna; CVn=CVnCoV vaccine, Curevac.

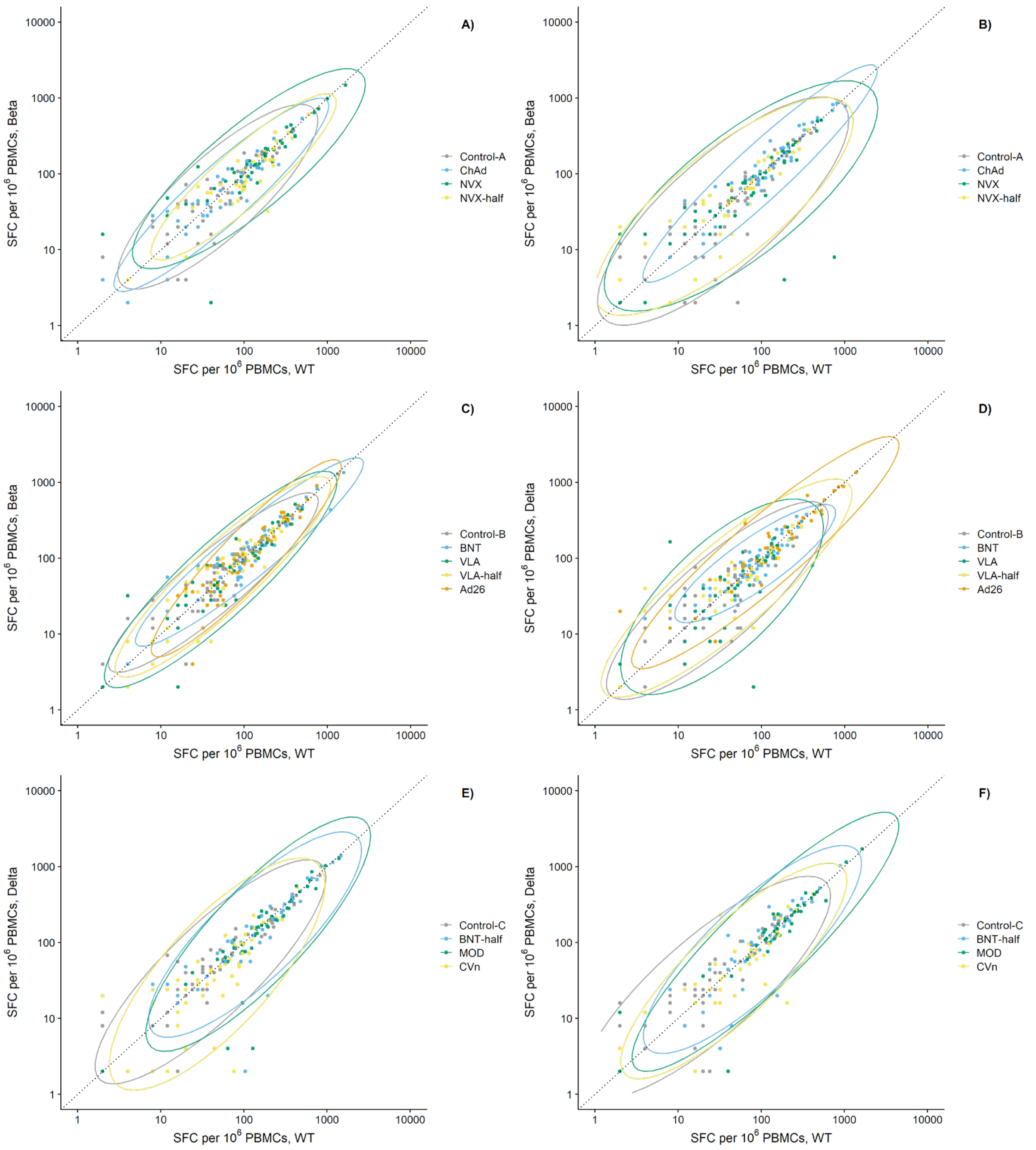
A)
10000
1000
100
10
1
SFC per 10^6 PBMCs, Beta
SFC per 10^6 PBMCs, WT
Control-A
ChAd
NVX
NVX-half
B)
SFC per 10^6 PBMCs, Beta
SFC per 10^6 PBMCs, WT
Control-A
ChAd
NVX
NVX-half
C)
SFC per 10^6 PBMCs, Beta
SFC per 10^6 PBMCs, WT
Control-B
BNT
VLA
VLA-half
Ad26
D)
SFC per 10^6 PBMCs, Delta
SFC per 10^6 PBMCs, WT
Control-B
BNT
VLA
VLA-half
Ad26
E)
SFC per 10^6 PBMCs, Delta
SFC per 10^6 PBMCs, WT
Control-C
BNT-half
MOD
CVn
F)
SFC per 10^6 PBMCs, Delta
SFC per 10^6 PBMCs, WT
Control-C
BNT-half
MOD
CVn

Supplementary Figure 6: Correlation between cellular response for WT and Delta

Correlation between cellular response against WT and cellular response against Delta strain at 28 days post 3rd dose in participants with A) ChAd/ChAd prime in Group A; B) BNT/BNT prime in Group A; C) ChAd/ChAd prime in Group B; D) BNT/BNT prime in Group B; E) ChAd/ChAd prime in Group C; F) BNT/BNT prime in Group C.

The dotted diagonal line shows the situation when the immunogenicity to a variant of concern (VOC) is the same as that to the WT (the vaccines were designed for). Ellipses show the 95% CIs for different vaccine schedules, assuming multivariate normal distributions. PBMC=peripheral blood mononuclear cell. SFC=spot-forming units. Control A/B/C=quadrivalent meningococcal conjugate vaccine; ChAd=ChAdOx1 nCoV-19 vaccine, Oxford–AstraZeneca; NVX=NVX-CoV2373 vaccine, Novavax; NVX half=half dose of NVX-CoV2373 vaccine; BNT=BNT162b2 vaccine (Pfizer–BioNTech); VLA=VLA2001 vaccine, Valneva; VLA half=half dose of VLA2001 vaccine; Ad26=Ad26.COV2.S vaccine, Janssen; BNT half=half dose of BNT162b2 vaccine; MOD=mRNA1273 vaccine, Moderna; CVn=CVnCoV vaccine, Curevac.

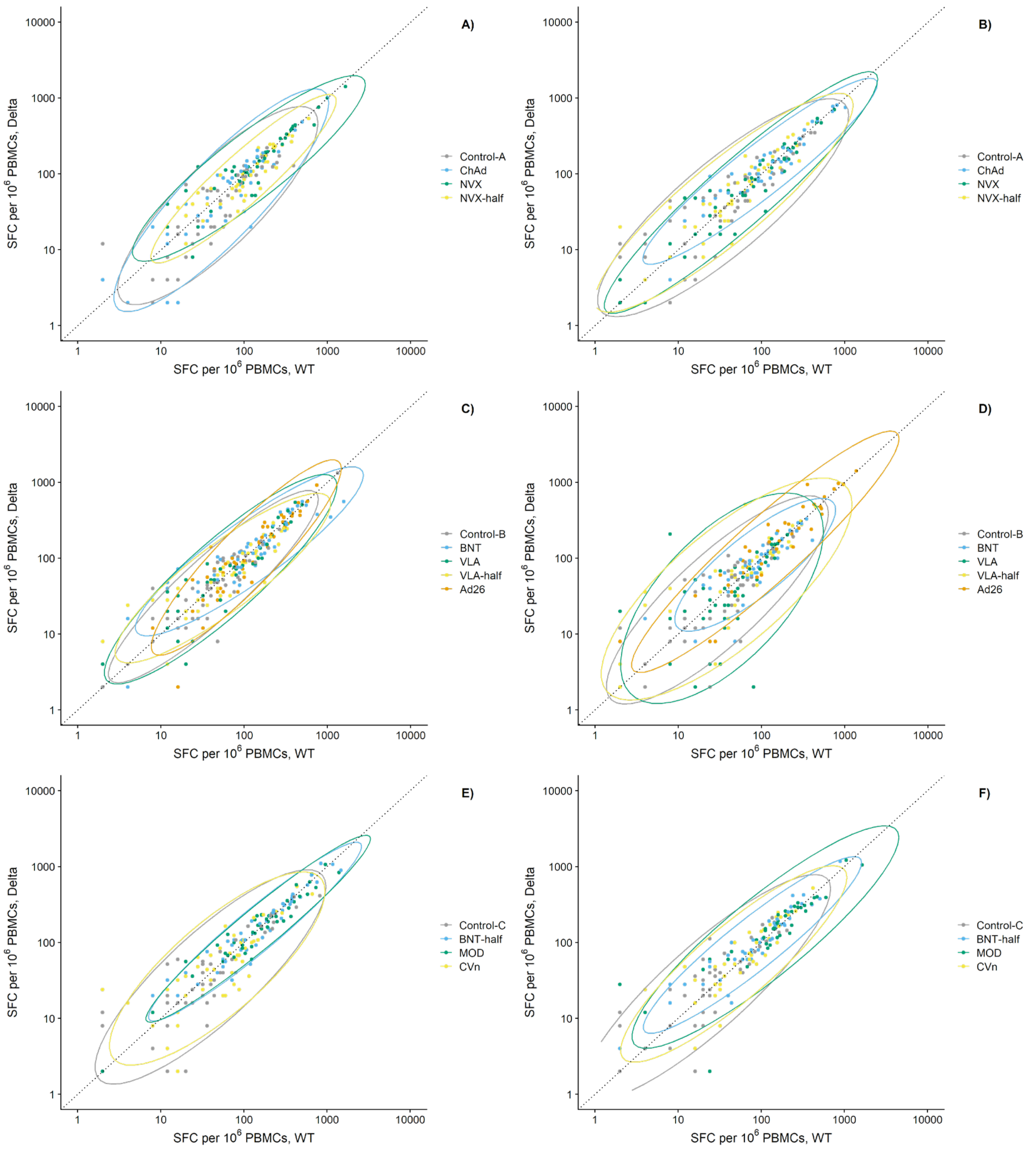
A)
B)
C)
D)
E)
F)
SFC per 10^6 PBMCs, Delta
SFC per 10^6 PBMCs, WT
1
10
100
1000
10000
Control-A
ChAd
NVX
NVX-half
Control-B
BNT
VLA
VLA-half
Ad26
Control-C
BNT-half
MOD
CVn

Supplementary Figure 7: Immunogenicity by Serostatus (anti-nucleocapsid IgG).

A) Anti-spike IgG and B) T cell response pre-3rd dose; C) Anti-spike IgG and D) T cell response at 28 days post 3rd dose vaccination by prime vaccine schedules between pre-vaccination serostatus (measured by anti-nucleocapsid IgG).

Control A/B/C=quadrivalent meningococcal conjugate vaccine; ChAd=ChAdOx1 nCoV-19 vaccine, Oxford–AstraZeneca; NVX=NVX-CoV2373 vaccine, Novavax; NVX half=half dose of NVX-CoV2373 vaccine; BNT=BNT162b2 vaccine (Pfizer–BioNTech); VLA=VLA2001 vaccine, Valneva; VLA half=half dose of VLA2001 vaccine; Ad26=Ad26.COV2.S vaccine, Janssen; BNT half=half dose of BNT162b2 vaccine; MOD=mRNA1273 vaccine, Moderna; CVn=CVnCoV vaccine, Curevac.

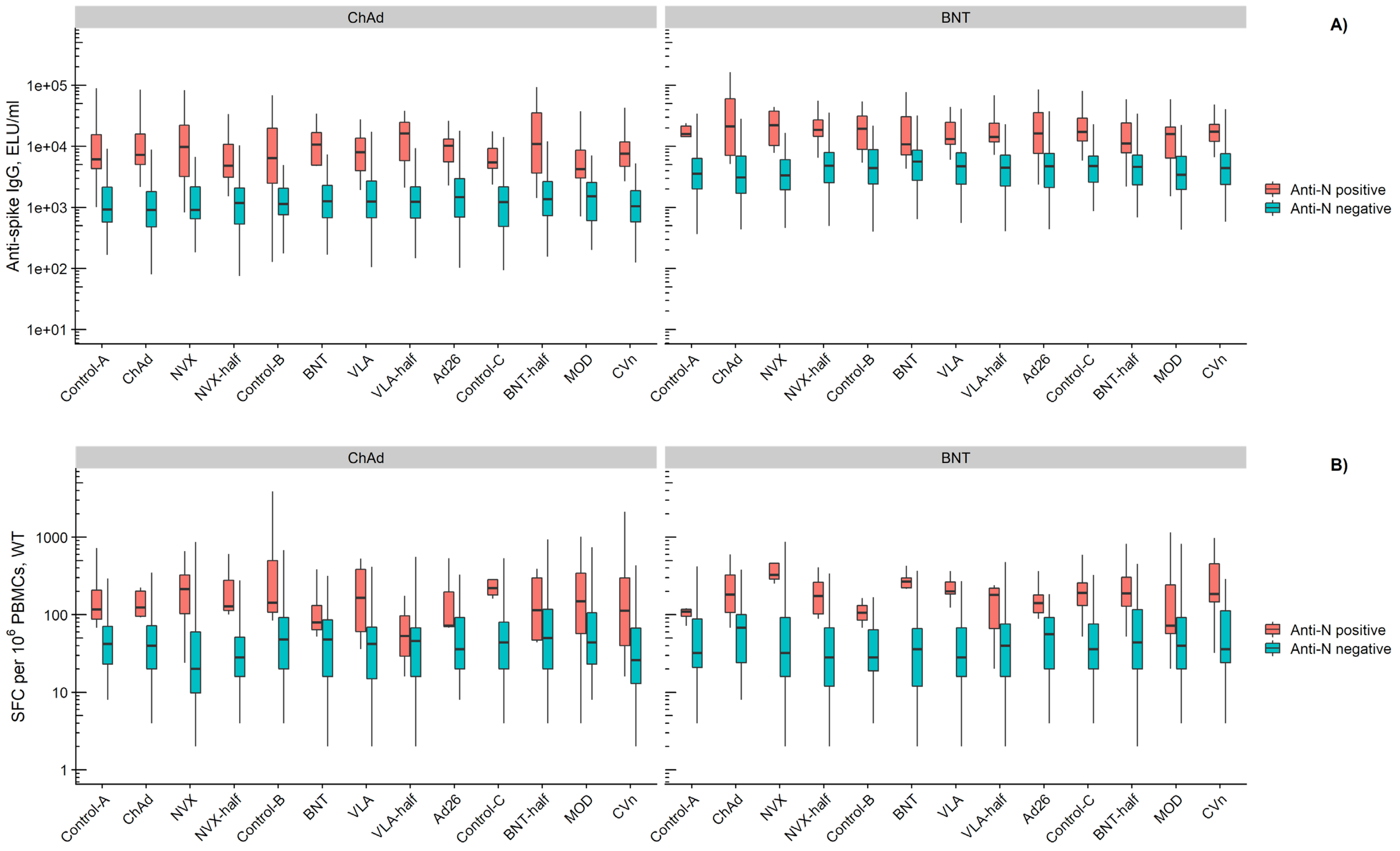
A)
B)
ChAd
BNT
Anti-spike IgG, ELU/ml
SFC per 10^6 PBMCs, WT
1e+05
1e+04
1e+03
1e+02
1e+01
1000
100
10
1
Control-A
ChAd
NVX
NVX-half
Control-B
BNT
VLA
VLA-half
Ad26
Control-C
BNT-half
MOD
CVn
Anti-N positive
Anti-N negative

C)

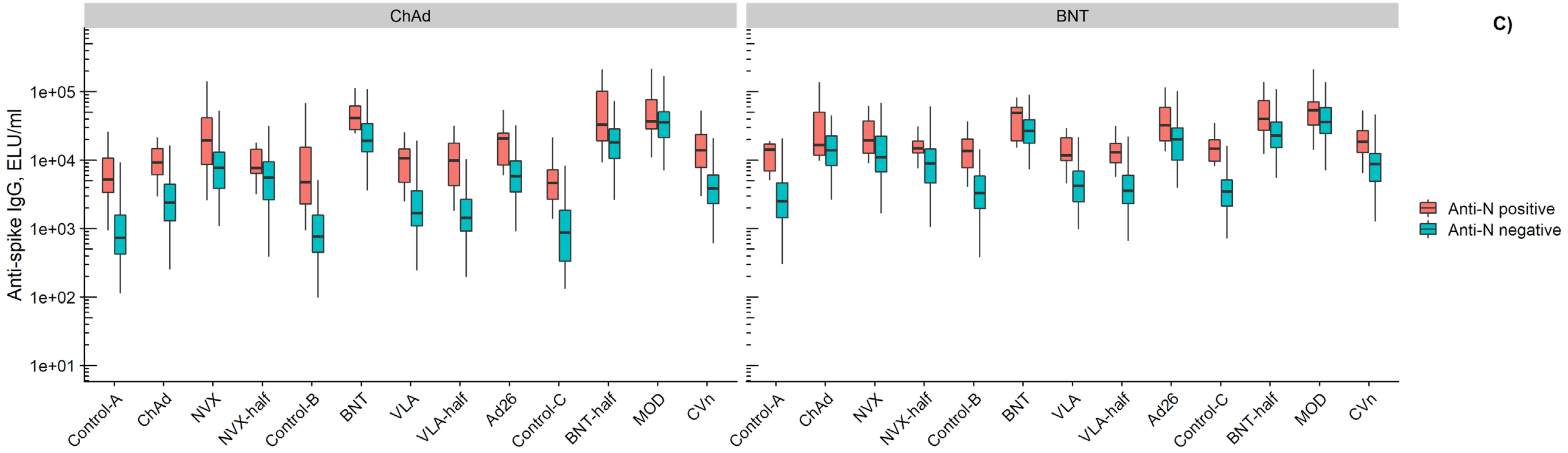
ChAd
BNT
Anti-spike IgG, ELU/ml
1e+05
1e+04
1e+03
1e+02
1e+01
Control-A
ChAd
NVX
NVX-half
Control-B
BNT
VLA
VLA-half
Ad26
Control-C
BNT-half
MOD
CVn
Anti-N positive
Anti-N negative

D)

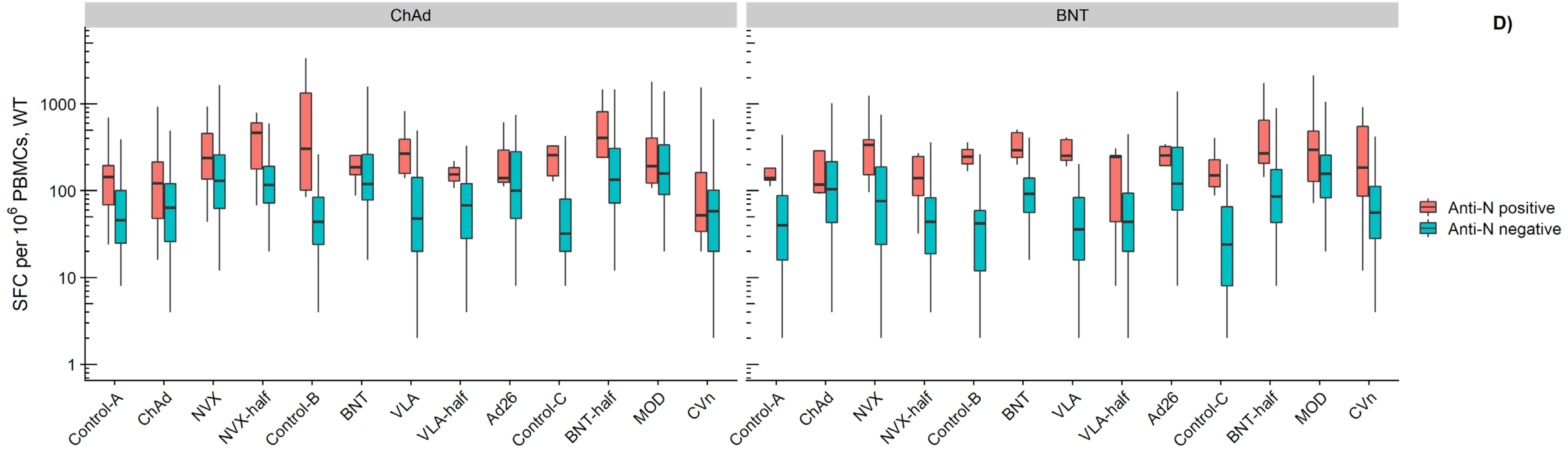
ChAd
BNT
SFC per 10^6 PBMCs, WT
1000
100
10
1
Control-A
ChAd
NVX
NVX-half
Control-B
BNT
VLA
VLA-half
Ad26
Control-C
BNT-half
MOD
CVn
Anti-N positive
Anti-N negative

Supplementary Figure 8: Correlation between anti-spike IgG and PNA

Correlation between anti-spike IgG and PNA against WT at 28 days post 3rd dose in participants with A) ChAd/ChAd prime in Group A; B) BNT/BNT prime in Group A; C) ChAd/ChAd prime in Group B; D) BNT/BNT prime in Group B; E) ChAd/ChAd prime in Group C; F) BNT/BNT prime in Group C.

Ellipses show the 95% CIs for different vaccine schedules, assuming multivariate normal distributions. ELU=ELISA laboratory units. NT50=50% neutralising antibody titre. Control A/B/C=quadrivalent meningococcal conjugate vaccine; ChAd=ChAdOx1 nCoV-19 vaccine, Oxford–AstraZeneca; NVX=NVX-CoV2373 vaccine, Novavax; NVX half=half dose of NVX-CoV2373 vaccine; BNT=BNT162b2 vaccine (Pfizer–BioNTech); VLA=VLA2001 vaccine, Valneva; VLA half=half dose of VLA2001 vaccine; Ad26=Ad26.COV2.S vaccine, Janssen; BNT half=half dose of BNT162b2 vaccine; MOD=mRNA1273 vaccine, Moderna; CVn=CVnCoV vaccine, Curevac.

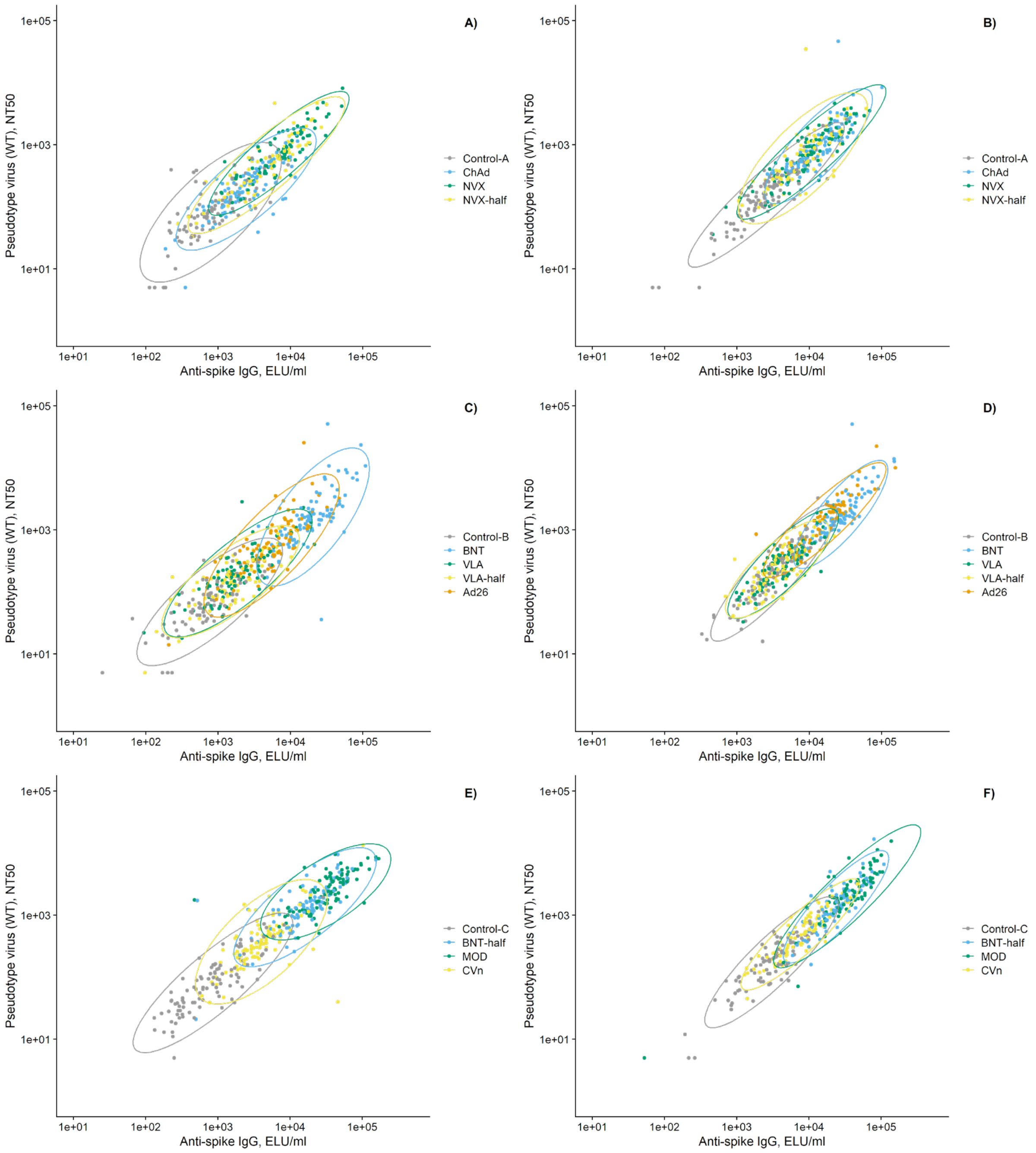
A)
B)
C)
D)
E)
F)
Pseudotype virus (WT), NT50
Anti-spike IgG, ELU/ml
1e+01
1e+02
1e+03
1e+04
1e+05
Control-A
ChAd
NVX
NVX-half
Control-B
BNT
VLA
VLA-half
Ad26
Control-C
BNT-half
MOD
CVn

Supplementary Figure 9: Correlation between anti-spike IgG and cellular response

Correlation between anti-spike IgG and cellular response against WT at 28 days post 3rd dose in participants with A) ChAd/ChAd prime in Group A; B) BNT/BNT prime in Group A; C) ChAd/ChAd prime in Group B; D) BNT/BNT prime in Group B; E) ChAd/ChAd prime in Group C; F) BNT/BNT prime in Group C.

Ellipses show the 95% CIs for different vaccine schedules, assuming multivariate normal distributions. ELU=ELISA laboratory units. PBMC=peripheral blood mononuclear cell. SFC=spot-forming units. Control A/B/C=quadrivalent meningococcal conjugate vaccine; ChAd=ChAdOx1 nCoV-19 vaccine, Oxford–AstraZeneca; NVX=NVX-CoV2373 vaccine, Novavax; NVX half=half dose of NVX-CoV2373 vaccine; BNT=BNT162b2 vaccine (Pfizer–BioNTech); VLA=VLA2001 vaccine, Valneva; VLA half=half dose of VLA2001 vaccine; Ad26=Ad26.COV2.S vaccine, Janssen; BNT half=half dose of BNT162b2 vaccine; MOD=mRNA1273 vaccine, Moderna; CVn=CVnCoV vaccine, Curevac.

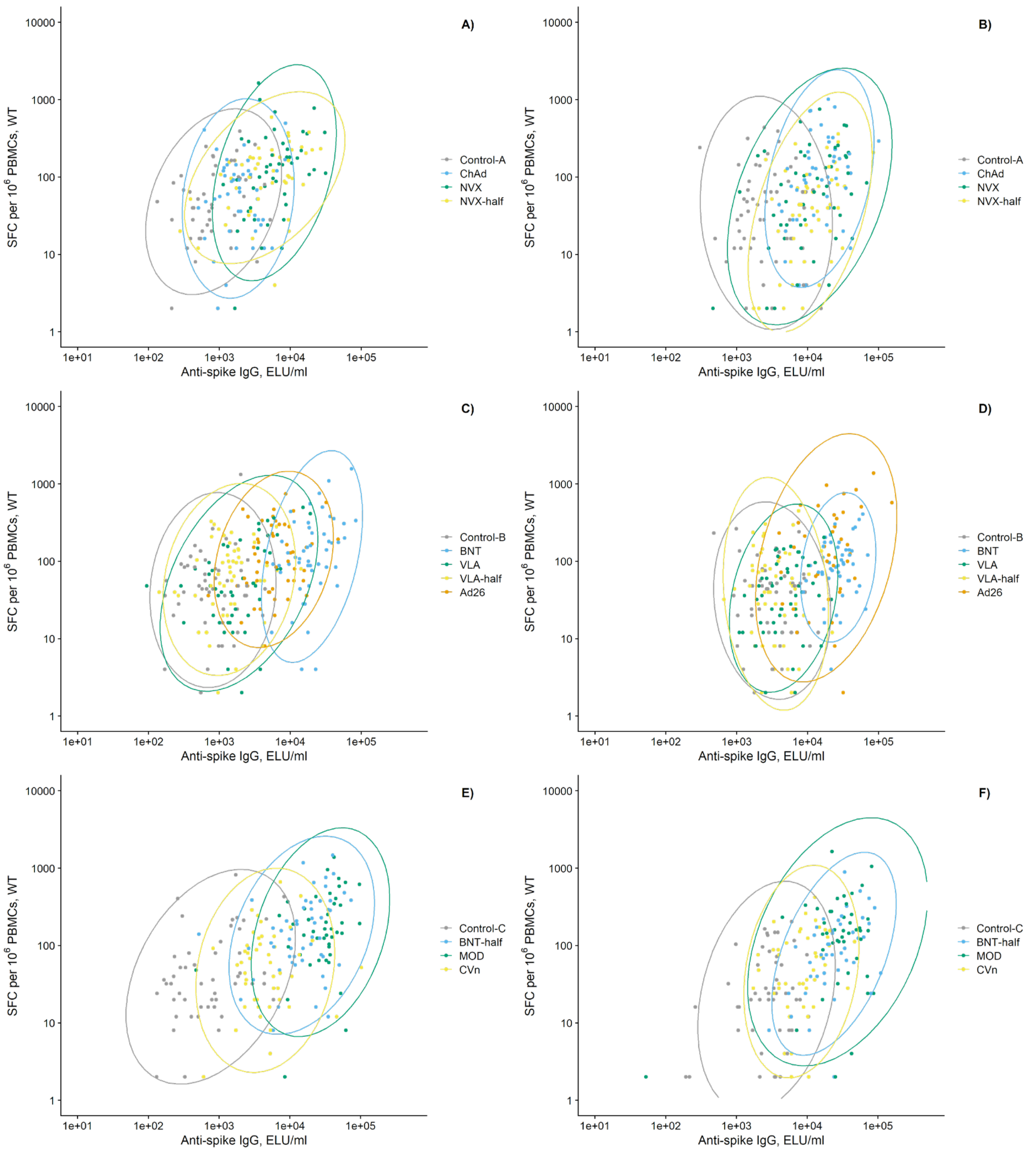

A)
B)
C)
D)
E)
F)
SFC per 10^6 PBMCs, WT
Anti-spike IgG, ELU/ml
Control-A
ChAd
NVX
NVX-half
Control-B
BNT
VLA
VLA-half
Ad26
Control-C
BNT-half
MOD
CVn

Supplementary Laboratory Information

Laboratory Methods

Serum was analysed at Nexelis (Laval, Canada) for SARS-CoV-2 anti-spike IgG concentrations by ELISA (reported as ELISA laboratory units [ELU]/mL), and for SARS-CoV-2 pseudotype virus neutralisation (PNA) assay, using a vesicular stomatitis virus backbone adapted to exhibit the SARS-CoV-2 spike protein, reported as 50% neutralising antibody titres (NT_{50}). PNA was performed on 50% of participants (same subset as cellular immunology samples) at day 0 and all participants at day 28. Serum from day 0 was analysed at Porton Down, Public Health England (PHE) by electrochemiluminescence immunoassay (Cobas platform, Roche Diagnostics) for SARS-CoV-2 anti-nucleocapsid IgG (reported as negative if below a cutoff index of 1.0). Live SARS-CoV-2 virus neutralisation (VNA, lineage Victoria/01/2020) was determined by microneutralisation assay (MNA) at Porton Down, reported as normalised $NT_{80.}$ Samples for VNA were pragmatically selected based on sample availability and laboratory capacity.

T-cell assays were performed at Oxford Immunotec (Abingdon, UK). IFN-γ secreting T cells specific to whole spike protein epitopes designed based on the Wuhan-Hu-1 sequence (YP_009724390.1), IFN-γ secreting T cells specific to whole spike protein epitopes (Panel 20) designed based on the SARS-CoV-2 Beta (B.1.351) variant of concern (GISAID whole genome accession number EPI_ISL_1052554), and IFN-γ secreting T cells specific to all genome-wide lineage defining mutations (Panel 17) and to whole spike protein epitopes (Panel 21) designed based on the SARS-CoV-2 Delta (B.1.617.2) variant of concern (GISAID whole genome accession number EPI_ISL_2022526), were detected by modified T-SPOT-Discovery test within 32h of venepuncture, using the addition of T-Cell Xtend reagent to extend peripheral blood mononuclear cell (PBMC) survival. T-cell frequencies were reported as spot forming cells (SFC) per 250,000 PBMCs with a lower limit of detection of one in 250,000 PBMCs, and these results were multiplied by four to express frequencies per million PBMCs.

Laboratory Units Conversion

Human SARS-CoV-2 Pre-Spike IgG ELISA Conversion

The results generated for the Human SARS-CoV-2 Pre-Spike IgG ELISA are reported with concentration units in “ELU/mL”. When required a correlation factor of 1/7.9815 will be applied to convert the reported results from ELU/mL to BAU/mL. For example, a sample with reported anti-PreSpike IgG antibody concentration of 7981.5 ELU/mL will have a concentration equivalent to 1000 BAU/mL.

The following formula may be used for converting concentration units from ELU/mL to BAU/mL:
Result (BAU/mL) = Result (ELU/mL) / 7.9815

Human SARS-CoV-2 Pseudoparticle Neutralisation Assay (PNA)

The results generated for the Human SARS-COV-2 PNA are reported with titer units “NT50”. When required, a correlation factor of 1/1.872 will be applied to convert the reported results from NT50 titer to IU/mL. For example, a sample with reported NT50 titer of 1872 will have a concentration equivalent to 1000IU/mL.

The following formula may be used for converting NT50 titer to IU/mL:

Result (IU/mL) = Result (NT50 titer) / 1.872

COV-BOOST Study Group

Name	Affiliation
Andrew Riordan	Data Monitoring Safety Committee, Alder Hey Children's Hospital
Andrew Ustianowski	Data Monitoring Safety Committee, North Manchester General Hospital
Chris A Rogers	Data Safety Monitoring Committee, University of Bristol
Stephen Hughes	Betsi Cadwaladr University Health Board
Laura Longshaw	Betsi Cadwaladr University Health Board
Jane Stockport	Betsi Cadwaladr University Health Board
Rachel Hughes	Betsi Cadwaladr University Health Board
Lynne Grundy	Betsi Cadwaladr University Health Board
Lona Tudor Jones	Betsi Cadwaladr University Health Board
Arpan Guha	Betsi Cadwaladr University Health Board
Emma Snashall	Betsi Cadwaladr University Health Board
Tom Eadsforth	Betsi Cadwaladr University Health Board
Sally Reeder	Betsi Cadwaladr University Health Board
Kim Storton	Bradford Teaching Hospitals NHS Foundation Trust
Malathi Munusamy	Bradford Teaching Hospitals NHS Foundation Trust
Bridget Tandy	Bradford Teaching Hospitals NHS Foundation Trust
Akamino Egbo	Bradford Teaching Hospitals NHS Foundation Trust
Stephen Cox	Bradford Teaching Hospitals NHS Foundation Trust
Nabeela Nazir Ahmed	Bradford Teaching Hospitals NHS Foundation Trust
Anil Shenoy	Bradford Teaching Hospitals NHS Foundation Trust

Rachel Bousfield	Department of Infectious Diseases, Cambridge University Hospitals NHS Foundation Trust
Donna Wixted	Dorset County Hospital
Helen Gutteridge	Dorset Research Hub
Becky Mansfield	Dorset Research Hub
Christopher Herbert	Leeds Teaching Hospitals NHS Trust
Kyra Holliday	Leeds Teaching Hospitals NHS Trust
James Calderwood	Leeds Teaching Hospitals NHS Trust
Dominique Barker	Leeds Teaching Hospitals NHS Trust
Jacqueline Brandon	Leeds Teaching Hospitals NHS Trust
Hayley Tulloch	Leeds Teaching Hospitals NHS Trust
Suzie Colquhoun	Leeds Teaching Hospitals NHS Trust
Helen Thorp	Leeds Teaching Hospitals NHS Trust
Helen Radford	Leeds Teaching Hospitals NHS Trust
Julie Evans	Leeds Teaching Hospitals NHS Trust
Helena Baker	Leeds Teaching Hospitals NHS Trust
Jeanette Thorpe	Leeds Teaching Hospitals NHS Trust
Sally Batham	Leicester CRF
Jessica Hailstone	Leicester CRF
Rachael Phillips	Leicester CRF
Dileep Kumar	Leicester CRF
Fran Westwell	Liverpool University Hospitals NHS Foundation Trust
Fiona Makia	London Northwest University Healthcare

NinaSimone Hopkins	London Northwest University Healthcare
Lara Barcella	London Northwest University Healthcare
Mushiya Mpelembue	London Northwest University Healthcare
Maja dabagh	London Northwest University Healthcare
Matilda lang	London Northwest University Healthcare
Farida khan	London Northwest University Healthcare
Olumide Adebambo	London Northwest University Healthcare
Sunder Chita	London Northwest University Healthcare
Tumena Corrah	London Northwest University Healthcare
Ashley Whittington	London Northwest University Healthcare
Laurence John	London Northwest University Healthcare
Siobhan Roche	National Institute of Health Research, West Midlands Clinical Research Network
Lynda Wagstaff	National Institute of Health Research, West Midlands Clinical Research Network
Adam Farrier	Newcastle University
Karen Bisnauthsing	NIHR BRC at Guy's and St Thomas' NHS Foundation Trust
Teona Serafimova	NIHR BRC at Guy's and St Thomas' NHS Foundation Trust
Elisa Nanino	NIHR BRC at Guy's and St Thomas' NHS Foundation Trust
Enya Cooney	NIHR BRC at Guy's and St Thomas' NHS Foundation Trust
Jaimie Wilson-Goldsmith	NIHR BRC at Guy's and St Thomas' NHS Foundation Trust
Hanna Nguyen	NIHR BRC at Guy's and St Thomas' NHS Foundation Trust
Andrea Mazzella	NIHR BRC at Guy's and St Thomas' NHS Foundation Trust
Beth Jackson	NIHR BRC at Guy's and St Thomas' NHS Foundation Trust

Suahil Aslam	NIHR BRC at Guy's and St Thomas' NHS Foundation Trust
Tanveer Bawa	NIHR BRC at Guy's and St Thomas' NHS Foundation Trust
Samantha Broadhead	NIHR BRC at Guy's and St Thomas' NHS Foundation Trust
Sadaf Farooqi	NIHR Clinical Research Facility, Cambridge University Hospitals NHS Foundation Trust
Jo Piper	NIHR Clinical Research Facility, Cambridge University Hospitals NHS Foundation Trust
Rowena Weighell	NIHR Clinical Research Facility, Cambridge University Hospitals NHS Foundation Trust
Lorinda Pickup	NIHR Clinical Research Facility, Cambridge University Hospitals NHS Foundation Trust
Djamila Shamtally	NIHR Clinical Research Facility, Cambridge University Hospitals NHS Foundation Trust
Jason Domingo	NIHR Clinical Research Facility, Cambridge University Hospitals NHS Foundation Trust
Evgenia Kourampa	NIHR Clinical Research Facility, Cambridge University Hospitals NHS Foundation Trust
Colin Hale	NIHR Liverpool & Broadgreen CRF
Jennifer Gibney	NIHR Liverpool & Broadgreen CRF
Michael Stackpoole	NIHR Liverpool & Broadgreen CRF
Zalina Rashid-Gardner	NIHR Liverpool & Broadgreen CRF
Rebecca Lyon	NIHR Liverpool & Broadgreen CRF
Chloe McDonnell	NIHR Liverpool & Broadgreen CRF
Christine Cole	NIHR Liverpool & Broadgreen CRF
Anna Stewart	NIHR Liverpool & Broadgreen CRF
Gillian McMillan	NIHR Liverpool & Broadgreen CRF
Mary Savage	NIHR Liverpool & Broadgreen CRF
Helen Beckett	NIHR Liverpool & Broadgreen CRF
Chantelle Moorbey	NIHR Wessex Local Clinical Research Network

Amisha Desai	NIHR/Wellcome Clinical Research Facility, University Hospitals Birmingham NHS Foundation Trust
Claire Brown	NIHR/Wellcome Clinical Research Facility, University Hospitals Birmingham NHS Foundation Trust
Kush Naker	NIHR/Wellcome Clinical Research Facility, University Hospitals Birmingham NHS Foundation Trust
Ehsaan Qureshi	NIHR/Wellcome Clinical Research Facility, University Hospitals Birmingham NHS Foundation Trust
Charlotte Trinham	NIHR/Wellcome Clinical Research Facility, University Hospitals Birmingham NHS Foundation Trust
Charlotte Sabine	NIHR/Wellcome Clinical Research Facility, University Hospitals Birmingham NHS Foundation Trust
Sophie Moore	NIHR/Wellcome Clinical Research Facility, University Hospitals Birmingham NHS Foundation Trust
Steve Hurdover	NIHR/Wellcome Clinical Research Facility, University Hospitals Birmingham NHS Foundation Trust
Edwin Justice	NIHR/Wellcome Clinical Research Facility, University Hospitals Birmingham NHS Foundation Trust
David Smith	Oxford Vaccine Group, Department of Paediatrics, University of Oxford, Oxford, UK
Emma Plested	Oxford Vaccine Group, Department of Paediatrics, University of Oxford, Oxford, UK
Carla Ferreira Da Silva	Oxford Vaccine Group, Department of Paediatrics, University of Oxford, Oxford, UK
Rachel White	Oxford Vaccine Group, Department of Paediatrics, University of Oxford, Oxford, UK
Hannah Robinson	Oxford Vaccine Group, Department of Paediatrics, University of Oxford, Oxford, UK
Liliana Cifuentes	Oxford Vaccine Group, Department of Paediatrics, University of Oxford, Oxford, UK
Gertraud Morshead	Oxford Vaccine Group, Department of Paediatrics, University of Oxford, Oxford, UK
Rachael Drake-Brockman	Oxford Vaccine Group, Department of Paediatrics, University of Oxford, Oxford, UK
Patrick Kinch	Oxford Vaccine Group, Department of Paediatrics, University of Oxford, Oxford, UK
Mwila Kasanyinga	Oxford Vaccine Group, Department of Paediatrics, University of Oxford, Oxford, UK
Elizabeth A. Clutterbuck	Oxford Vaccine Group, Department of Paediatrics, University of Oxford, Oxford, UK

Sagida Bibi	Oxford Vaccine Group, Department of Paediatrics, University of Oxford, Oxford, UK
Arabella SV Stuart	Oxford Vaccine Group, Department of Paediatrics, University of Oxford, Oxford, UK
Robert H Shaw	Oxford Vaccine Group, Department of Paediatrics, University of Oxford, Oxford, UK
Michael Singh	Pharmacy Clinical Trials (Adult), Guy's and St Thomas NHS Foundation Trust
Trishna Champaneri	PHARMExcel
Margaret Irwin	PHARMExcel
Mohammed Khan	PHARMExcel
Alicja Kownacka	PHARMExcel
Martha Nabunjo	PHARMExcel
Carool Osuji	PHARMExcel
John Hladkiwskyj	PHARMExcel
Dominic Galvin	PHARMExcel
Gita Patel	PHARMExcel
Johanna Mouland	Portsmouth Hospitals University NHS Trust
Beverley Longhurst	Portsmouth Hospitals University NHS Trust
Maria Moon	Portsmouth Hospitals University NHS Trust
Beth Giddins	Portsmouth Hospitals University NHS Trust
Carlota Pereira Dias Alves	Portsmouth Hospitals University NHS Trust
Leah Richmond	Portsmouth Hospitals University NHS Trust
Christine Minnis	Portsmouth Hospitals University NHS Trust
Sonia Baryschpolec	Portsmouth Hospitals University NHS Trust
Scott Elliott	Portsmouth Hospitals University NHS Trust

Lauren Fox	Portsmouth Hospitals University NHS Trust
Victoria Graham	Public Health England
Natalie Baker	Public Health England
Kerry Godwin	Public Health England
Karren Buttigieg	Public Health England
Chanice Knight	Public Health England
Phillip Brown	Public Health England
Paminder Lall	Public Health England
Imam Shaik	Public Health England
Emily Chiplin	Public Health England
Emily Brunt	Public Health England
Stephanie Leung	Public Health England
Lauren Allen	Public Health England
Steve Thomas	Public Health England
Sara Fraser	Public Health England
Bea Choi	Public Health England
Jade Gouriet	Public Health England
Andrew Freedman	Public Health Wales
Jonathan Perkins	Queen Elizabeth University Hospital, NHS Greater Glasgow & Clyde
Andrew Gowland	Queen Elizabeth University Hospital, NHS Greater Glasgow & Clyde
Jonathan Macdonald	Queen Elizabeth University Hospital, NHS Greater Glasgow & Clyde
John Paul Seenan	Queen Elizabeth University Hospital, NHS Greater Glasgow & Clyde

Igor Starinskij	Queen Elizabeth University Hospital, NHS Greater Glasgow & Clyde
Andrew Seaton	Queen Elizabeth University Hospital, NHS Greater Glasgow & Clyde
Erica Peters	Queen Elizabeth University Hospital, NHS Greater Glasgow & Clyde
Stephen Singh	Royal Devon and Exeter Hospital
Ben Gardside	Royal Devon and Exeter Hospital
Avril Bonnaud	Royal Devon and Exeter Hospital
Ceri Davies	Royal Devon and Exeter Hospital
Elizabeth Gordon	Royal Devon and Exeter Hospital
Samantha Keenan	Royal Devon and Exeter Hospital
Jane Hall	Royal Devon and Exeter Hospital
Suzanne Wilkins	Royal Devon and Exeter Hospital
Suzanne Tasker	Royal Devon and Exeter Hospital
Rob James	Royal Devon and Exeter Hospital
Ingrid Seath	Royal Devon and Exeter Hospital
Kelly Littlewood	Royal Devon and Exeter Hospital
Joseph Newman	Royal Papworth Hospital NHS Foundation Trust
Iryna Boubriak	Royal Papworth Hospital NHS Foundation Trust
Debbie Suggitt	Stockport NHS Foundation Trust
Helen Haydock	Stockport NHS Foundation Trust
Sara Bennett	Stockport NHS Foundation Trust
Wiesia Woodyatt	Stockport NHS Foundation Trust
Kerry Hughes	Stockport NHS Foundation Trust

Judith Bell	Stockport NHS Foundation Trust
Tricia Coughlan	Stockport NHS Foundation Trust
Donald van Welsenes	Stockport NHS Foundation Trust
Mohammed Kamal	Stockport NHS Foundation Trust
Chris Cooper	Stockport NHS Foundation Trust
Simon Tunstall	Stockport NHS Foundation Trust
Nicholas Ronan	Stockport NHS Foundation Trust
Rebecca Cutts	The Adam Practice
Tracey Dare	The Adam Practice
Yee Ting Nicole Yim	University College London Hospitals NHS Foundation Trust
Sarah Whittley	University College London Hospitals NHS Foundation Trust
Marivic Ricamara	University College London Hospitals NHS Foundation Trust
Shama Hamal	University College London Hospitals NHS Foundation Trust
Kirsty Adams	University College London Hospitals NHS Foundation Trust
Holly Baker	University College London Hospitals NHS Foundation Trust
Kimberley Driver	University College London Hospitals NHS Foundation Trust
Nicola Turner	University College London Hospitals NHS Foundation Trust
Todd Rawlins	University College London Hospitals NHS Foundation Trust
Subarna Roy	University College London Hospitals NHS Foundation Trust
Marta Merida-Morillas	University College London Hospitals NHS Foundation Trust
Yukari Sakagami	University College London Hospitals NHS Foundation Trust
Antonette Andrews	University College London Hospitals NHS Foundation Trust

Lillian Goncalves cordeiro	University Hospital Southampton NHS Foundation Trust
Matthew Stokes	University Hospital Southampton NHS Foundation Trust
Wythehi Ambihapathy	University Hospital Southampton NHS Foundation Trust
Joanne Spencer	University Hospital Southampton NHS Foundation Trust
Nina Parungao	University Hospital Southampton NHS Foundation Trust
Lisa Berry	University Hospital Southampton NHS Foundation Trust
James Cullinane	University Hospital Southampton NHS Foundation Trust
Laura Presland	University Hospital Southampton NHS Foundation Trust
Amy Ross-Russell	University Hospital Southampton NHS Foundation Trust
Sarah Warren	University Hospital Southampton NHS Foundation Trust
Jonathan Baker	University Hospital Southampton NHS Foundation Trust
Abigail Oliver	University Hospital Southampton NHS Foundation Trust
Amanda Buadi	University Hospital Southampton NHS Foundation Trust
Kim Lee	University Hospital Southampton NHS Foundation Trust
Louise Haskell	University Hospital Southampton NHS Foundation Trust
Rossana Romani	University Hospital Southampton NHS Foundation Trust
Ian Bentley	University Hospital Southampton NHS Foundation Trust
Tim Whitbred	University Hospital Southampton NHS Foundation Trust
Simon Fowler	University Hospital Southampton NHS Foundation Trust
John Gavin	University Hospital Southampton NHS Foundation Trust
Alan Magee	University Hospital Southampton NHS Foundation Trust
Tara Watson	University Hospital Southampton NHS Foundation Trust

Kari Nightingale	University Hospital Southampton NHS Foundation Trust
Phedra Marius	University Hospital Southampton NHS Foundation Trust
Eloise Summerton	University Hospital Southampton NHS Foundation Trust
Emily Locke	University Hospital Southampton NHS Foundation Trust
Thomas Honey	University Hospital Southampton NHS Foundation Trust
Aidan Lingwood	University Hospital Southampton NHS Foundation Trust
Anastasia de la Haye	University Hospital Southampton NHS Foundation Trust
Ryan Stephen Elliott	University Hospital Southampton NHS Foundation Trust
Karen Underwood	University Hospital Southampton NHS Foundation Trust
Mikayala King	University Hospital Southampton NHS Foundation Trust
Sharon Davies-Dear	University Hospital Southampton NHS Foundation Trust
Emily Horsfall	University Hospital Southampton NHS Foundation Trust
Olivia Chalwin	University Hospital Southampton NHS Foundation Trust
Holly Burton	University Hospital Southampton NHS Foundation Trust
Christopher J Edwards	University Hospital Southampton NHS Foundation Trust
Benjamin Welham	University Hospital Southampton NHS Foundation Trust
Sarah Garrahy	University Hospital Sussex NHS Trust
Fran Hall	University Hospital Sussex NHS Trust
Eleni Ladikou	University Hospital Sussex NHS Trust
Dee Mullan	University Hospital Sussex NHS Trust
Daniel Hansen	University Hospital Sussex NHS Trust
Marion Campbell	University Hospital Sussex NHS Trust

Filipa Dos Santos	University Hospital Sussex NHS Trust
Haniah Habash-Bailey	University Hospital Sussex NHS Trust
Nicki Lakeman	University Hospitals Dorset
Debbie Branney	University Hospitals Dorset
Luke Vamplew	University Hospitals Dorset
Alison Hogan	University Hospitals Dorset
Jorden Frankham	University Hospitals Dorset
Martin Wiselka	University Hospitals of Leicester NHS Trust
Denny Vail	University Hospitals of Leicester NHS Trust
Victoria Wenn	University Hospitals of Leicester NHS Trust
Valerie Renals	University Hospitals of Leicester NHS Trust
Kate Ellis	University Hospitals of Leicester NHS Trust
Jessica Lewis-Taylor	University Hospitals of Leicester NHS Trust
Javier Magan	NIHR Wessex Clinical Research network
Anna Hardy	NIHR Wessex Clinical Research network
Kim Appleby	NIHR Wessex Clinical Research Network
