# Supplementary material for: Safety and immunogenicity of seven COVID-19 vaccines as a third dose (booster) following two doses of ChAdOx1 nCov-19 or BNT162b2 in the UK (COV-BOOST): a blinded, multicentre, randomised, controlled, phase 2 trial
Source: Lancet. 2021 Dec 18;398(10318):2258–76. doi: 10.1016/S0140-6736(21)02717-3 (PMC8639161; doi:10.1016/S0140-6736(21)02717-3)
Supplement: Supplementary appendix [file mmc2.pdf]

THE LANCET

Supplementary appendix

This appendix formed part of the original submission and has been peer reviewed. We post it as supplied by the authors.

Supplement to: Munro APS, Janani L, Cornelius V, et al. Safety and immunogenicity of seven COVID-19 vaccines as a third dose (booster) following two doses of ChAdOx1 nCov-19 or BNT162b2 in the UK (COV-BOOST): a blinded, multicentre, randomised, controlled, phase 2 trial. *Lancet* 2021; published online Dec 2. https://doi.org/10.1016/S0140-6736(21)02717-3.

Safety and immunogenicity of seven COVID-19 vaccines as a third dose (booster) following two doses of ChAdOx1-nCov19 (AZD1222) or BNT162b2: A blinded, randomised controlled trial (COV-BOOST)

Supplementary Appendix 2

Contents

COV-BOOST Protocol 2

COV-BOOST Statistical Analysis Plan 104

COV-BOOST
Evaluating COVID-19 vaccine boosters

University of Southampton | NHS University Hospital Southampton NHS Foundation Trust

Study Title: A randomised, phase II UK multi-centre study to determine reactogenicity and immunogenicity of booster vaccination against ancestral and novel variants of SARS-CoV-2

Short Title: Evaluating COVID-19 Vaccine Boosters

IRAS Project ID: 299180

EudraCT Number: 2021-002175-19

UHS Study Number: RHM MED1781

Date and Version No: V6.0 21st October 2021

Chief Investigator: Professor Saul Faust
NIHR Southampton Clinical Research Facility
University Hospital Southampton NHS Foundation Trust,
Southampton SO16 6YD

Investigators:

Sponsor: University Hospital Southampton NHS Foundation Trust

Funder: National Institute Health Research (NIHR), supported by the Vaccine Task Force and DHSC.

Chief Investigator Signature:

Sponsor Signature:

Statistician Signature:

1 Contents

2 Key Trial Contacts ... 8

3 Lay Summary ... 11

4 Background ... 12

5 Study Rationale ... 12

6 Synopsis ... 13

7 Abbreviations ... 20

8 Risks and Benefits ... 22

8.1 Potential benefits ... 22

8.2 Potential risks ... 23

8.2.1 Associated with phlebotomy ... 23

8.2.2 Associated with saliva sampling ... 23

8.2.3 Associated with nasal fluid sampling ... 23

8.2.4 Allergic reactions ... 23

8.2.5 Behaviour change ... 23

8.2.6 Specific risks from vaccines ... 23

8.2.7 Antibody-dependent enhancement and immunopathology ... 23

8.2.8 Unwanted media attention ... 24

8.2.9 Reactogenicity ... 24

9 Study Design ... 24

9.1 Trial Design ... 24

9.1.1 Setting ... 24

9.1.2 Trial duration ... 24

9.1.3 Study groups ... 25

9.1.4 Stage 1 ... 25

9.1.5 Stage 2 (optional group, not funded at time of stage 1 submissions and set up) ... 26

9.1.6 Stage 3 (optional group, not funded at time of stage 1 submissions and set up) ... 27

9.1.7 Stage 1 Group allocations ... 27

9.1.8 Stage 2 Group allocations 29

9.1.9 Stage 3 Group allocations 29

9.1.10 Routine NHS deployment of booster vaccination 30

9.1.11 External Vaccine trial participants Sub-Study 31

10 Study participants 32

10.1 Trial Participants 32

10.2 Inclusion Criteria 32

10.3 Exclusion Criteria 33

10.3.1 Temporary exclusion criteria 34

10.4 Eligibility for External Vaccine Trial Participants Sub-Study 34

11 Trial procedures 36

11.1 Recruitment 36

11.1.1 Volunteer Identification 36

11.2 Screening and Eligibility Assessment 37

11.2.1 Initial screening – Main Trial Only 37

11.2.2 Telephone screening visit(s) – Main Trial Only 38

11.2.3 Screening during Visit 1 38

11.2.4 Screening: External Vaccine Trial Sub-Study 38

11.3 Informed Consent 38

11.4 Randomisation 40

11.5 Blinding and code-breaking 41

11.6 Visits 41

11.6.1 Visit 1 (D0): Final eligibility check, Enrolment and Vaccination visit 42

11.6.2 Safety review 44

11.6.3 Subsequent visits 44

11.6.4 Participants under quarantine 45

11.6.5 Participants with confirmed SARS-CoV-2 infection (COVID-19 Pathway) 45

11.6.6 Admission of participants to hospital with COVID-19 infection 47

11.7 Sample Handling ... 47
11.7.1 Sample handling for trial purposes ... 47
11.7.2 Sample handling for standard of care ... 48
11.8 Early Discontinuation/Withdrawal of Participants ... 49
11.9 Definition of End of Trial ... 50
12 Trial Interventions ... 50
12.1 Investigational Medicinal Product(s) (IMP) Description ... 50
12.1.1 AstraZeneca COVID-19 vaccine (ChAdOx1 nCOV-19) ... 50
12.1.2 Pfizer BioNTech (BNT162b2) ... 51
12.1.3 Moderna (mRNA-1273) ... 51
12.1.4 Novavax (NVXCoV2373) ... 52
12.1.5 Valneva (VLA2001) ... 52
12.1.6 Curevac (CVnCoV) ... 53
12.1.7 Janssen (Ad26.COV2.S) ... 53
12.1.8 Control vaccine ... 53
12.1.9 Blinding of IMPs ... 54
12.1.10 Storage of IMP ... 54
12.1.11 Compliance with Trial Treatment ... 56
12.1.12 Accountability of the Trial Treatment ... 56
12.1.13 Concomitant Medication ... 56
12.1.14 Post-trial Treatment ... 56
12.2 Other Treatments (non-IMPS) ... 57
12.3 Other Interventions ... 57
13 Safety Reporting ... 57
13.1 Safety reporting window ... 57
13.2 Adverse Event Definitions ... 57
13.3 Assessment results outside of normal parameters as AEs and SAEs ... 59
13.3.1 Clinical ... 59

13.3.2 Laboratory ... 59

13.4 Assessment of severity ... 59

13.5 Assessment of Causality ... 61

13.6 Procedures for Reporting Adverse Events ... 62

13.6.1 Solicited AEs ... 62

13.6.2 Unsolicited AEs ... 62

13.6.3 Medically attended AEs ... 62

13.7 Reporting Procedures for Serious Adverse Events ... 62

13.7.1 Events exempt from immediate reporting as SAEs ... 63

13.8 Expectedness ... 63

13.8.1 SAEs ... 63

13.8.2 Thrombocytopaenic thrombosis following adenovirus vector vaccination ... 69

13.8.2.1 ChAdOx1-nCov19 (Oxford) ... 69

13.8.2.2 Ad26.COV2.S (Janssen) ... 70

13.8.3 Disease enhancement following vaccination ... 70

13.9 SUSAR Reporting ... 70

13.10 Development Safety Update Reports ... 70

13.11 Interim reviews ... 71

13.12 Safety Holding Rules ... 71

13.13 Contraception and pregnancy ... 71

13.13.1 Contraception ... 71

13.13.2 Pregnancy ... 72

14 Statistics ... 72

14.1 Sample size ... 72

14.2 Description of statistical methods ... 73

14.3 Interim analyses ... 74

14.4 Missing data ... 75

15 Data Management ... 75

15.1 Access to Data & Data Protection 75
15.2 Data Recording 75
15.3 Record keeping 76
15.4 Source Data and Case Report Forms (CRFs) 77
15.5 Data Quality 77
15.6 Data Sharing 77
16 Quality Assurance Procedures 78
16.1 Risk assessment 78
16.2 Monitoring 78
16.3 Trial committees 78
16.3.1 Trial Steering Committee 78
16.3.2 Data Safety Monitoring Board 78
16.3.3 Study Management Group 79
17 Protocol Deviations 79
18 Serious Breaches 79
19 Ethical And Regulatory Considerations 79
19.1 Declaration of Helsinki 79
19.2 Guidelines for Good Clinical Practice 80
19.3 Approvals 80
19.4 Other Ethical Considerations 80
19.5 Reporting 80
19.6 Transparency in Research 80
19.7 Participant Confidentiality 81
19.8 Expenses and Benefits 81
20 Finance And Insurance 81
20.1 Funding 81
20.2 Contractual arrangements 82
21 Publication Policy 82

22 Development Of A New Product/ Process Or The Generation Of Intellectual Property 82

23 Archiving 82

24 References 83

25 Appendix A 84

25.1 Schedule of visits for general cohort 84

25.2 Schedule of visits for immunology cohorts 85

25.3 Schedule of additional visits for unblinded control arms in event of NHS national booster campaign 86

25.4 Schedule of visits for External vaccine trials participant 87

26 Appendix B: Amendment History 87

27 Appendix C: Toxicity grading scale for lab AEs 93

28 Appendix D: Blood Sampling 94

2 Key Trial Contacts

**Chief Investigator**	Professor Saul Faust<br>Professor of Paediatric Immunology and Infectious Diseases<br>NIHR Southampton Clinical Research Facility<br>University Hospital Southampton NHS Foundation Trust<br>Tremona Road, Southampton<br>United Kingdom<br>s.faust@soton.ac.uk
**Sponsor**	Sharon Davies-Dear<br>University Hospital Southampton NHS Foundation Trust<br>Tremona Road<br>Southampton<br>Hampshire, SO16 6YD<br>023 8120 5146<br>Email: Sharon.davies-dear@uhs.nhs.uk
**Funder(s)**	National Institute for Health Research & UK Vaccine Task Force
**Clinical Trials Unit**	Oxford Vaccine Group,<br>University of Oxford,<br>Centre for Clinical Vaccinology and Tropical Medicine (CCVTM),<br>Churchill Hospital,<br>Oxford, OX3 7LE,<br>United Kingdom<br>info@ovg.ox.ac.uk
**External Contract Research Organisation (CRO)**	PHARMExcel Ltd<br>Albany Chambers<br>26 Bridge Road East<br>Welwyn Garden City<br>Hertfordshire<br>AL7 1HL<br>United Kingdom

**Statisticians**	**Xinxue Liu**<br>Oxford Vaccine Group, Centre for Clinical Vaccinology and Tropical Medicine (CCVTM),<br>University of Oxford, Churchill Hospital, Oxford, OX3 7LE,<br>xinxue.liu@paediatrics.ox.ac.uk<br>**Nick Andrews**<br>Public Health England<br>61 Colindale Ave, London NW9 5EQ<br>nick.andrews@phe.gov.uk<br>**Leila Janani**<br>Imperial College London<br>Exhibition Rd, South Kensington, London SW7 2BX<br>l.janani@imperial.ac.uk<br>**Victoria Cornelius**<br>Imperial College London<br>Exhibition Rd, South Kensington, London SW7 2BX<br>v.cornelius@imperial.ac.uk

**Committees**	**Data Safety Monitoring Board**<br>Chair: Prof Andrew Ustianowski, North Manchester General Hospital<br>Statistician: Prof Chris Rogers, University of Bristol<br>Clinical vaccine expert: Dr Andrew Riordan, Alder Hey Hospital Liverpool.<br>Open meetings: CI, Sponsor Representatives, PHARMExcel representative.<br><br>**Trial Steering Group**<br>Chair: Prof Rob Read, University of Southampton<br>Prof Mary Ramsay, Public Health England<br>Dr Paul Turner, Imperial College London<br>Prof Claire Cameron, University College London<br>Dr Karen Underwood (Sponsor Rep), UHSFT<br>Prof Chris Kipps (Sponsor Rep), UHSFT<br>Yvanne Enever (CRO Rep), PHARMExcel<br><br>**Study Management Group**<br>S Faust (CI)<br>Site PIs:<br>A Minassian (or delegate)<br>K Chatterjee (or delegate)<br>D Saralaya (or delegate)<br>A Goodman (or delegate)<br>C Twelves (or delegate)<br>M Bula (or delegate)<br>E Thomson (or delegate)<br>A Mcgregor (or delegate)<br>O Osanlou (or delegate)<br>R Sheridan (or delegate)<br>D Baxter (or delegate)<br>V Libri (or delegate)<br>K Cathie (or delegate)<br>C Green (or delegate)<br>A Palfreeman (or delegate)<br>S Sharma (or delegate)

	Project Management:<br><br>S Saich (Senior Vaccine Project Manager)<br>S Davies-Dear (Sponsor Representative)<br>PHARMexcel (CRO) project managers

3 Lay Summary

As of 1st June 2021 the MHRA in the UK has granted Regulation 174 approval for emergency use of 4 vaccines for protection against COVID-19 in the UK, including the mRNA vaccines BNT162b2 (Pfizer) the mRNA-1273 (Moderna) vaccine, the chimpanzee adenovirus vector vaccine ChAdOx1-nCov19 (AstraZeneca/Oxford), and the human adenovirus vector vaccine Ad26.COV2.S (Janssen). So far over 33 million people in the UK have received at least one dose of either BNT162b2, ChAdOx1-nCov19, or mRNA-1273. Annual or seasonal booster vaccination for high risk groups is thought likely to be required, especially in light of the emergence of new variants of the SARS-CoV-2 virus. There is concern from studies in South Africa and elsewhere that existing vaccines may be less effective against these variant strains. It is currently unclear which booster vaccine schedule will provide the best safety profile and immune responses, according to which vaccine was originally given. The Joint Committee for Vaccination and Immunisation and UK Chief Medical Officers need timely information regarding the effects of different booster vaccinations on the safety profile and immunity to previous and new variants of SARS-CoV-2 in order to inform national policy for autumn and winter 2021. This study will determine the immune responses provided from different booster vaccinations given a minimum of 3 months from the 2nd dose of an initial course of AstraZeneca/Oxford or Pfizer vaccines.

4 Background

The COVID-19 pandemic was met with a rapid global effort to produce safe and efficacious vaccines to protect vulnerable individuals against severe disease, and to reduce transmission of the virus. Within a year there had been multiple vaccine candidates which were successfully granted emergency use authorisation by several high stringency agencies on the bases of successful clinical safety and efficacy endpoints in phase 3 clinical trials.

Whilst immunity conferred by natural infection has been shown to by durable for many months [1], long term immunogenicity data is not yet available for vaccines beyond 6 months [2]. There is an expectation that the durability of immune response may be affected by 2 factors; the waning over time of immune memory elicited by vaccines, and the emergence of novel variants of the SARS-Cov-2 virus with substantive mutations to the spike protein which enable varying levels of antigen escape [3, 4]. This is likely to necessitate booster doses of COVID-19 vaccines, and potentially periodic boosters. Boosters will either increase personal protection via generating higher antibody levels against the original pandemic variant regardless of circulating variant; or in due course by generating immunity specifically against novel variants of the virus.

5 Study Rationale

The 2 vaccines which have been delivered at scale in the UK so far have been the mRNA vaccine BNT162b2 (Pfizer) and the chimpanzee adenovirus vector vaccine ChAdOx1-nCov19 (Oxford/AstraZeneca). Studies are underway currently to examine the effect of heterologous 2 dose primary regimens using these differing vaccine technologies (the COM-COV trial, IRAS Project ID: 291055 EudraCT Number: 2020-005085-33).

The COV-BOOST trial aims to establish the safety and immunogenicity of seasonal boosting vaccination with different authorised vaccine candidates for participants who have received homologous prim-boosting with either BNT162b2 or ChAdOx1-nCov19 early in the UK NHS deployment campaign. In addition, this study will examine the safety and efficacy of boosting using vaccines designed specifically to target novel variants of the SARS-CoV-2 virus when these vaccines become available.

6 Synopsis

Trial Title	A randomised, phase II UK multi-centre study to determine reactogenicity and immunogenicity of booster vaccination against SARS-CoV-2
Internal ref. no. (or short title)	Evaluating COVID-19 Vaccine Boosters (COV-BOOST)
Trial registration	EudraCT: 2021-002175-19<br>ISRCTN: ISRCTN73765130
Sponsor	University Hospital Southampton NHS Foundation Trust<br>Tremona Road<br>Southampton<br>SO16 6YD
Funder	National Institute for Health Research
Clinical Phase	Phase II
Trial Design	Three stage, randomized, adaptive phase II multicentre study of annual booster vaccination against ancestral and novel variants of SARS-CoV-2. Participants and laboratory staff will remain blind to treatment allocation. Analysing statisticians will be blinded for any stages or sub-stages after sign off of Statistical Analysis Plan (SAP) V1.0
Trial Participants	Adults aged 30 years or older who received a complete homologous 2 dose primary course of vaccination against COVID-19 with their first dose in December 2020, January or February 2021, and their booster at least 84 days prior to day 0 (70 days minimum allowable with Sponsor approval at site). Recruitment will be from participants aged 30 years and older (including specifically those aged 75 years and over).<br>Participants that have taken part in the Phase III NVXCoV2373 clinical trial are eligible for enrolment to the external vaccine trial sub-study of this protocol.
Sample Size	**Existing SARS-CoV-2 strain booster (stage 1)**<br>Stage 1 will be run across 3 groups (A-C) which will each be enrolled at different clusters of sites, with a total of 2886 participants. Site groups A and C will have 888 participants consisting of a cohort of

444 participants who received 2 doses of BNT162b2 with the second dose at least 3 months (=>84 days) prior to enrolment and 444 participants who received 2 doses of ChAdOx1 nCov19 with the second dose at least 3 months (=>84 days) prior to enrolment. Site group B will have 1110 participants consisting of a cohort of 555 participants who received 2 doses of BNT162b2 with the second dose at least 3 months (=>84 days) prior to enrolment and 555 participants who received 2 doses of ChAdOx1-nCov19 with the second dose at least 3 months (=>84 days) prior to enrolment. Total target enrolment figures and the target number of participants per arm are approximates, some minor variation is expected as a result of randomisation stratification.

Site Group A

Each of 2 cohorts of 444 participants will be randomised 1:1:1:1 to receive a single dose of the following vaccines:

- ChAdOx1 nCOV-19 (111 participants)
- Novavax (111 participants)
- Novavax half dose[+] (111 participants)
- Men ACWY (control) (111 participants)

Site Group B

Each of 2 cohorts of 555 participants will be randomised 1:1:1:1:1 to receive a single dose of the following vaccines:

- BNT162b2 (111 participants)
- VLA2001 (111 participants)
- VLA2001 half dose[+] (111 participants)
- Ad26.COV2.S (111 participants)
- MenACWY (111 participants)

Site Group C

Each of 2 cohorts of 444 participants will be randomised 1:1:1:1 to receive a single dose of the following vaccines:

- mRNA-1273 (111 participants)
- CVnCoV (111 participants)
- BNT162b2 half dose[+] (111 participants)
- Men ACWY (111 participants)

Existing SARS-CoV-2 strain booster (stage 2, not yet agreed/funded, left in protocol now for information re how the trial might evolve during the pandemic)

This adaptive and optional stage 2 is in preparation for vaccines which are not authorized or available for use in the UK at the time Stage 1 begins (this may include vaccines currently planned for Stage 1).

	As new vaccines are provided an emergency use authorization by MHRA they will be assigned a new group of 111 participants in each cohort as in stage 1 (For each vaccine, 111 people who have 2 doses of BNT162b2 with the second dose at least 3 months (=>84 days) prior to enrolment and 111 participants who received 2 doses of ChAdOx1 nCov19 with the second dose at least 3 months (=>84 days) prior to enrolment. A further 111 control participants will receive Men ACWY. Total target enrolment figures and the target number of participants per arm are approximates, some minor variation is expected as a result of randomisation stratification.<br><br>Each of 2 cohorts of 222 participants will be randomised 1:1(:1) to receive a single dose of the following vaccines:<br><br>• Vaccine G (111 participants)<br>• Men ACWY (control) (111 participants)<br><br>If other vaccines become available in the interim, a planned amendment will be added to add 2 additional groups with 111 participants each (who have each received 2 doses of BNT162b2 or ChAdOx1 nCov19).<br><br>**Novel variant booster (stage 3, not yet agreed/funded, left in protocol now for information re how the trial might evolve during the pandemic)**<br><br>This adaptive stage 3 is in preparation for future viral variant vaccines. An additional 1110 participants will be enrolled, consisting of a cohort of 555 participants who received 2 doses of BNT162b2 with the second dose at least 3 months (=>84 days) prior to enrolment and 555 participants who received 2 doses of ChAdOx1 nCov19 with the second dose at least 3 months (=>84 days) prior to enrolment. Total target enrolment figures and the target number of participants per arm are approximates, some minor variation is expected as a result of randomisation stratification.<br><br>Each of 2 cohorts of 555 participants will be randomised 1:1:1:1:1 to receive a single dose of the following vaccines:<br><br>• Vaccine H (111 participants)<br>• Vaccine I (111 participants)<br>• Vaccine J (111 participants)<br>• Vaccine K (111 participants)<br>• Men ACWY (control) (111 participants)
Planned Trial Period	12 months per participant in the main study (following on from the first vaccination)<br><br>6 – 10 months per participant in the external vaccine trial sub-study<br><br>Total trial period 1 year, 6 months

	Objectives	Outcome Measures	Timepoint(s)
Co-Primary	To determine the safety and reactogenicity of annual booster doses of vaccines against SARS-CoV-2 following a homologous 2 dose primary regime	Solicited and unsolicited adverse events<br>Serious adverse events<br>Adverse events of special interest	Solicited adverse events: Day 0-7 after immunisation<br>Unsolicited adverse events: Day 0-28 after immunisation<br>Serious adverse events: throughout the study<br>Adverse events of special interest: throughout the study
Co-Primary	To determine whether the immune response to booster immunisation with different COVID-19 vaccines is superior to control vaccination for participants who have received priming vaccination with either ChAdOx1-nCov19 or BNT162b2	Immunogenicity: Anti spike protein IgG	Day 28
Secondary	Further characterisation of immunogenicity of booster vaccination against ancestral and novel variants of SARS-CoV-2	Anti-spike immunoglobulins	Day 0, 7*, 28, 84, 365
		Neutralising antibodies against SARS-CoV-2	Day 0, 28, 84, 365
		Anti-nucleocapsid immunoglobulins	Day 0, 84, 365
		Pseudo neutralising antibodies	Day 0, 28, 84, 365
		Cellular immune responses by ELISpot**	Day 0, 14*, 28, 84, 365

		Cellular immune responses by ICS (Th1/Th2)**	D0*, 14*
	To characterise immune responses of 3rd dose booster vaccinations when administered approximately 6 months after 2nd dose, and in comparison to those administered after 3 months	Anti-spike immunoglobulins, neutralising and pseudo-neutralising antibodies, cellular immune response by ICS** and ELISpot**	Post unblinding of the control vaccine arms: UCD0, UCD14, UCD28
	To assess changes in markers of cardiac muscle damage in participants receiving a 3rd dose COVID-19 booster vaccine approximately 6 months after 2nd dose, post initial vaccination course	High sensitivity Troponin	Post unblinding of the control vaccine arms: UCD0, UCD14
	To characterise immune responses of 3rd dose booster vaccination with BNT162b2 when administered after a primary immunization course of purified protein technology (NVXCoV2373)	Anti-spike immunoglobulins, neutralising and pseudo-neutralising antibodies, cellular immune response by ICS** and ELISpot**	Day 0, 14, 28, 84, 242
	Safety of booster vaccination against ancestral and novel variants of SARS-CoV-2	Medically attended adverse events	Up to 3 months post immunisation
		Changes from baseline in laboratory safety measures	Day 0, 7*, 28,
	To determine the reactogenicity of 3rd dose booster vaccinations when administered approximately 6 months after 2nd dose, and in	Solicited and unsolicited adverse events<br>Medically attended adverse events	Solicited adverse events: Day 0-7 after immunisation

	comparison to those administered after 3 months		Unsolicited adverse events: Day 0-28 after immunization<br>Up to 3 months post immunisation
	To determine the reactogenicity of 3rd dose booster vaccination with BNT162b2 when administered after a primary immunization course of purified protein technology (NVXCoV2373)	Solicited and unsolicited adverse events<br>Medically attended adverse events	Solicited adverse events: Day 0-7 after immunisation<br>Unsolicited adverse events: Day 0-28 after immunization<br>Up to 3 months post immunisation
Exploratory	To characterise COVID-19 infections experienced following booster vaccination against ancestral and novel variants of SARS-CoV-2	Anti-spike & anti-nucleocapsid immunoglobulins, neutralising and pseudo-neutralising antibodies, cellular immune response by ICS and ELISpot<br>Genome sequencing of SARS-CoV-2 viruses isolated from infected participants	From vaccination, and within 1 week of a participant being found to be SARS-CoV-2 positive by external testing
	To further characterise the blood antibody response to booster vaccination against ancestral and novel variants of SARS-CoV-2	Functional antibody assays in line with other NISEC studies.	Day 0, 28, 365
	To characterise and compare the mucosal immune response to immunisation with homologous and heterologous COVID-19 vaccines in the immunology cohort using nasal	IgA & IgG ELISA and exploratory immunological assays	Day 0, 28, 365

	fluid (collected using SAM-strips) and saliva samples. *		

* Only applies to the immunology cohort comprising approximately 25 participants from each group

** Only applies to centres collecting Lithium Heparin blood samples (approximately 50% of participants

+ Half doses of vaccine have been included to investigate as potential dose sparing regimen for use in the UK or globally due to potential shortage of vaccine supply

Intervention(s)

- IMP(s)

Vaccine	Dose	Route of administration
AstraZeneca COVID-19 vaccine AZD1222 (ChAdOx1 nCOV-19)	$5x10^{10}$vp (0.5ml)	Intramuscular
Pfizer BioNTech (BNT162b2)	30 µg (0.3ml)	Intramuscular
Pfizer BioNTech Half dose (BNT162b2)	15 µg (0.15ml)	Intramuscular
NVX–CoV2373, Novavax	5 µg SARS-CoV-2 rS + 50 µg Matrix-M1 adjuvant (0.5ml)	Intramuscular
NVX–CoV2373, Novavax Half Dose+	2.5 µg SARS-CoV-2 rS + 25 µg Matrix-M1 adjuvant (0.25ml)	Intramuscular
VLA2001, Valneva	33 AU + 0.5mg Aluminium hydroxide +1mg CPG (0.5ml)	Intramuscular
VLA2001, Valneva Half Dose+	16.5 AU + 0.25mg Aluminium hydroxide + 0.5mg CPG (0.25ml)	Intramuscular

		mRNA 1273, Moderna	0.10 mg (0.5ml)	Intramuscular
		mRNA 1273, Moderna	0.05 mg (0.25ml)	Intramuscular
		CVnCoV, Curevac	12 µg (0.6ml)	Intramuscular
		Janssen, Ad26COV2.S	$5x10^{10}$vp (0.5ml)	Intramuscular
		Men ACWY	0.5ml	Intramuscular

[+] Half doses of vaccine have been included to investigate as potential dose sparing regimen for use in the UK or globally due to potential shortage of vaccine supply

7 Abbreviations

ADE	Antibody Dependant Enhancement
AE	Adverse event
AESI	Adverse Event of Special Interest
AR	Adverse reaction
AU	Antigen Units
C-19P	COVID-19 Pathway
ChAdOx1	Chimpanzee adenovirus 1
CI	Chief Investigator
CRF	Case Report Form
CT	Clinical Trials
CTA	Clinical Trials Authorisation
CTRG	Clinical Trials and Research Governance
DSMB	Data Safety Monitoring Board
DSUR	Development Safety Update Report

EDC	Electronic Data Capture
ELISPOT	Enzyme-linked Immunospot
FBC	Full blood count
GCP	Good Clinical Practice
GMT	Geometric Mean Titre
GP	General Practitioner
HIV	Human Immunodeficiency virus
HRA	Health Research Authority
IB	Investigators Brochure
ICS	Intracellular Cytokine Staining
ICF	Informed Consent Form
IM	Intramuscular
IMP	Investigational Medicinal Product
IV	Intravenous
JCVI	Joint Committee on Vaccination and Immunisation
MHRA	Medicines and Healthcare products Regulatory Agency
NHS	National Health Service
NIHR	National Institute for Health Research
NISEC	National Immunisation Schedule Evaluation Consortium
PBMC	Peripheral blood mononuclear cell
PCR	Polymerase chain reaction
qPCR	Quantitative polymerase chain reaction
RES	Research Ethics Service
PB	Post-booster
PI	Principal Investigator
PIS	Participant/ Patient Information Sheet

REC	Research Ethics Committee
RSI	Reference Safety Information
SAE	Serious Adverse Event
SAM-strips	Synthetic absorbable matrix strips
SAR	Serious Adverse Reaction
SMPC	Summary of Medicinal Product Characteristics
SOP	Standard Operating Procedure
SUSAR	Suspected Unexpected Serious Adverse Reactions
TMF	Trial Master File
TSG	University Hospital Southampton NHS Foundation Trust Trials Steering Group
UHS NHSFT	University Hospital Southampton NHS Foundation Trust
µg	Microgram
Vp	Viral particle
VTF	Vaccine Task Force
WHO	World Health Organisation

8 Risks and Benefits

8.1 Potential benefits

Participants in this study will have already received a 2 dose primary regime of an approved COVID-19 vaccine through the NHS. Administration of the third dose ‘boost’ of COVID-19 vaccine in this study (from 84 days post dose 2) may be administered earlier than it would be through routine immunisation which is of potential benefit.

It is hoped that the information gained from this study will contribute to the development of a safe, effective and versatile vaccine programme against COVID-19.

8.2 Potential risks

8.2.1 Associated with phlebotomy

Localised bruising and discomfort can occur at the site of venepuncture. Infrequently fainting may occur. These will not be documented as AEs if they occur. The total volume of blood drawn over a 12 month period will be up to 311ml (+ up to 67ml per COVID-19 visit if required, and/or up to 7ml per additional set of safety bloods, + up to 164ml if in the control arm Immunology cohort attending for unblinded booster vaccination) (blood volumes may vary slightly for participants at different investigator sites due to use of different volume vacutainers, following local Trust SOPs). This should not compromise these otherwise healthy volunteers, as these volumes are within the limits of 470mL every 3 – 4 months for blood donations to the National Blood Transfusion Service. Participants will be asked to refrain from blood donation for the duration of their involvement in the trial.

8.2.2 Associated with saliva sampling

Participants may find the saliva collection process unsavoury as it is involves drooling and spitting.

8.2.3 Associated with nasal fluid sampling

Localised discomfort can occur in the nostril. Infrequently, this can result in a small amount of epistaxis, which can be controlled with pressure to the affected area.

8.2.4 Allergic reactions

Allergic reactions from mild to severe may occur in response to any constituent of a medicinal product's preparation. Anaphylaxis is known to occur in approximately 2.5 to 4.7 per million recipients of mRNA COVID-19 vaccines[5], and more generally in around 1 in 1,000,000 doses of all vaccines, but can occur in response to any vaccine or medication[6].

8.2.5 Behaviour change

Participants might feel they can modify their COVID-19 risk behaviours on the assumption that they are protected once vaccinated. Participants will be extensively counselled that they should continue to follow all up to date government advice in relation to COVID-19 precautions during the trial.

8.2.6 Specific risks from vaccines

Please refer to Section 13.8.1 for full details.

8.2.7 Antibody-dependent enhancement and immunopathology

Safety concerns around the use of some viral antigens as a vaccine antigen have been raised following historical and limited reports of immunopathology and antibody dependant enhancement (ADE)

reported in vitro and post SARS-CoV challenge in mice, ferrets and non-human primates immunised with whole SARS-CoV inactivated or full-length S protein based vaccines, including a study using Modified Vaccinia Ankara as a vector [7-9]. To date, there has been one report of lung immunopathology following MERS-CoV challenge in mice immunised with an inactivated MERS-CoV candidate vaccine [8]. However, in preclinical studies of ChAdOx1 immunisation and MERS-CoV challenge, no ADE was observed in hDPP4 transgenic mice, dromedary camels or non-human primates[10, 11].

The possibility of ADE has also been evaluated in clinical and pre-clinical studies of the vaccines used in this trial. Nevertheless, this risk will not have been assessed for heterologous boost schedules. Participants will be made aware of this theoretical risk.

8.2.8 Unwanted media attention

Trial participants can be subjected to unwanted attention from the media. They will therefore be provided with access to a document outlining some suggested media guidance.

8.2.9 Reactogenicity

Preliminary data from the “Comparing COVID-19 vaccine schedule combinations” study (COMCOV) has indicated that there may be increased reactogenicity at the second dose of vaccine from heterologous 2 dose primary regimens than from homologous. It is possible that participants might experience more reactogenicity if their booster vaccine is different to the vaccine used in their 2 dose primary regime.

9 Study Design

9.1 Trial Design

A randomised, phase II UK multi-centre study to determine reactogenicity and immunogenicity of booster vaccination against ancestral and novel variants of SARS-CoV-2. Participants, laboratory and analysing statisticians will remain blind to treatment allocation.

9.1.1 Setting

Multicentre study conducted through academic and NHS clinical trials sites.

9.1.2 Trial duration

Total duration of each participant in the primary phase of Stage 1 will be 12 months from the administration of the first vaccine dose. The total duration of each participant in the External Vaccine

Trial Sub-Study will be 8 months (+/-60 days) from vaccination in the trial. The total trial period will be approximately 1 year, 6 months.

9.1.3 Study groups

The study will initially consist of several cohorts enrolled in 2 or 3 stages

9.1.4 Stage 1

The initial aim is to test several different COVID-19 vaccines which are available and have already, or are expected to be imminently granted reg 147 use by the MHRA in the UK, as annual booster doses for participants who have received a homologous 2 dose primary vaccine schedule with either BNT162b2 (Pfizer) or ChAdOx1-nCov19 (AstraZeneca/Oxford).

Stage 1 will be run across 3 groups (A-C) which will each be enrolled at different clusters of sites, with a total of 2886 participants.

Site groups A and C will have 888 participants consisting of a cohort of 444 participants who received 2 doses of BNT162b2 with the second dose at least 3 months (=>84 days) prior to enrolment and 444 participants who received 2 doses of ChAdOx1 nCov19 with the second dose at least 3 months (=>84 days) prior to enrolment.

Site group B will have 1110 participants consisting of a cohort of 555 participants who received 2 doses of BNT162b2 with the second dose at least 3 months (=>84 days) prior to enrolment and 555 participants who received 2 doses of ChAdOx1-nCov19 with the second dose at least 3 months (=>84 days) prior to enrolment.

Each of the 3 groups in Stage 1 will be enrolled at separate clusters of 6 trial sites to reduce the risk of medication/dosing errors.

Group A: 888 participants (including 444 vaccinated previously with 2 doses of BNT162b2 and 444 vaccinated previously with 2 doses of ChAdOx1-nCov19) will be randomised 1:1:1:1 to receive a booster dose of either ChAdOx1-nCov19, NVX–CoV2373 (Novavax), NVX-CoV2373 half dose, or MenACWY (control group).

Group B: 1110 participants (including 555 vaccinated previously with 2 doses of BNT162b2 and 555 vaccinated previously with 2 doses of ChAdOx1-nCov19) will be randomised 1:1:1:1:1 to receive a booster dose of either BNT162b2, VLA2001 (Valneva), VLA2001 half dose, Ad26.COV2.S (Janssen) or MenACWY (control group).

Group C: 888 participants (including 444 vaccinated previously with 2 doses of BNT162b2 and 444 vaccinated previously with 2 doses of ChAdOx1-nCov19) will be randomised 1:1:1:1 to receive a booster dose of either mRNA-1273 (Moderna), CVnCoV (Curevac), BNT162b2 half dose, or MenACWY (control group).

All participants will be monitored for adverse events and have bloods for immunogenicity taken at days 28, 84, 308 and 365. The primary outcome assessed from stage 1 will be a geometric mean ratio in day 28 anti-spike protein IgG of 1.75 compared to the control group. In addition, an immunology subgroup comprising approximately 25 participants of each vaccine group will attend on day 7 and day 14 for immunology bloods. Half doses of 3 vaccines have been included at the request of the UK Chief Medical Office and Vaccine Task Force to investigate as potential dose sparing regimen for use in the UK or globally due to potential shortage of vaccine supply. Total target enrolment figures and the target number of participants per arm are approximates, some minor variation is expected as a result of randomisation stratification.

For procedures in the event of NHS deployment of COVID-19 booster vaccines, see section 9.1.10.

9.1.5 Stage 2 (optional group, not funded at time of stage 1 submissions and set up)

This stage is adaptive/optional and is available to utilise any additional vaccines which become available to the trial after commencement of stage 1. The design of stage 2 will mimic stage 1. If an additional vaccine is available (or if one of the vaccines planned for inclusion for stage 1 is unavailable at the time it commences and is deferred to stage 2), a further 444 participants will be enrolled (including 222 vaccinated previously with BNT162b2 and 222 vaccinated previously with ChAdOx1-nCov19). They will be randomised 1:1 to receive Vaccine C or Men ACWY as a booster. Participants will be monitored for adverse events and have bloods for immunogenicity taken at days 28, 84, 308 and 365. In addition, an immunology subgroup comprising approximately 25 participants of each vaccine group will attend on day 7 and day 14 for immunology bloods. The primary outcome assessed from stage 2 will be a geometric mean ratio in day 28 anti-spike protein IgG of 1.75 compared to the control group. If/when further vaccines become available, a planned amendment will be added to include additional groups of 222 participants (111 who have previously received 2 doses of BNT162b2 and 111 who have had 2 doses of ChAodOx1-nCov19). Total target enrolment figures and the target number of participants per arm are approximates, some minor variation is expected as a result of randomisation stratification.

9.1.6 Stage 3 (optional group, not funded at time of stage 1 submissions and set up)

New vaccine candidates which are targeted against novel strains of SARS-CoV-2 will be assessed for their safety and immunogenicity profile when used as a booster vaccine for participants who have received a homologous 2 dose primary vaccine schedule with either BNT162b2 or ChAdOx1-nCov19. 1110 participants (including 555 vaccinated previously with BNT162b2and 555 vaccinated previously with ChAdOx1-nCov19) will be randomised 1:1:1:1:1 to receive booster vaccination with Vaccine E, Vaccine F, Vaccine G, Vaccine H or Men ACWY. Participants will be monitored for adverse events and have bloods for immunogenicity taken at days 28, 84, 308 and 365. In addition, an immunology subgroup comprising approximately 25 participants of each vaccine group will attend on day 7 and day 14 for immunology bloods. The primary outcome assessed from stage 3 will be a geometric mean ratio in day 28 anti-spike protein IgG of 1.75 compared to the control group. Total target enrolment figures and the target number of participants per arm are approximates, some minor variation is expected as a result of randomisation stratification.

9.1.7 Stage 1 Group allocations

Group	2 dose Primary	Subgroup	Booster	Visits
**Group A (n=888)**	ChAdOx1-nCov19 (n=444)	ChAd-ChAd (n=111)	ChAdOx1-nCov19	Day 0, 7* 14*, 28, 84, 365
		ChAd- NVX (n=111)	NVX–CoV2373	
		ChAd- NVX50 (n=111)	NVX–CoV2373 Half Dose[+]	
		ChAd-Men (n=111)	MenACWY	
	BNT162b2 (n=444)	BNT-ChAd (n=111)	ChAdOx1-nCov19	Day 0, 7* 14*, 28, 84, 365
		BNT-NVX (n=111)	NVX–CoV2373	
		BNT-NVX50 (n=111)	NVX–CoV2373 Half Dose[+]	
		BNT-Men (n=111)	Men ACWY	
**Group B (n=1110)**	ChAdOx1-nCov19 (n=555)	ChAd-BNT (n=111)	BNT162b2	Day 0, 7* 14*, 28, 84, 365
		ChAd-VLA (n=111)	VLA2001	
		ChAd-VLA50 (n=111)	VLA2001 Half Dose[+]	
		ChAd-Jan (n==111)	Ad26.COV2.S	

		ChAd-Men (n=111)	Men ACWY	
	BNT162b2 (n=555)	BNT-BNT (n=111)	BNT162b2	Day 0, 7* 14*, 28, 84, 365
		BNT-VLA (n=111)	VLA2001	
		BNT-VLA50 (n=111)	VLA2001 Half Dose[+]	
		BNT-Jan (n=111)	Ad26.COV2.S	
		BNT-Men (n=111)	Men ACWY	
**Group C (n=888)**	ChAdOx1-nCov19 (n=444)	ChAd-Mod (n=111)	mRNA-1273	Day 0, 7* 14*, 28, 84, 365
		ChAd-CVn (n=111)	CVnCoV	
		ChAd-BNT50 (n=111)	BNT162b2 Half Dose[+]	
		ChAd-Men (n=111)	Men ACWY	
	BNT162b2 (n=444)	BNT-Mod (n=111)	mRNA-1273	Day 0, 7* 14*, 28, 84, 365
		BNT-CVn (n=111)	CVnCoV	
		BNT-BNT50 (n=111)	BNT162b2 Half Dose[+]	
		BNT-Men (n=111)	Men ACWY	

9.1.7.1 Stage 1 Unblinded control group allocations

Groups	2 dose primary	Subgroup	Booster	Extra visits
Unblinded Control Groups (2 per site group, n=666)	ChAdOx1-nCov19 (n=333)	ChAd-L-BNT (n=111)	BNT162b2	UCD0, 14*, 28
		ChAd-L-BNT50 (n=111)	BNT162b2 Half dose[+]	
		ChAd-L-Mod50 (n=111)	mRNA-1273 Half dose[+]	
	BNT162b2 (n=333)	ChAd-L-BNT	BNT162b2	UCD0, 14*, 28

		(n=111)		
		ChAd-L-BNT50 (n=111)	BNT162b2 Half dose[+]	
		ChAd-L-Mod50 (n=111)	mRNA-1273 Half dose[+]	

* Only applies to the immunology cohort comprising approximately 25 participants from each group

[+] Half doses of 4 vaccines have been included at the request of the UK Chief Medical Office and Vaccine Task Force to investigate as potential dose sparing regimen for use in the UK or globally due to potential shortage of vaccine supply.

9.1.7.2 Stage 1 external vaccine trial sub-study

Groups	2 dose primary	Booster	Visits
External Vaccine Trial Sub-Study (NVX-CoV2373)	NVX–CoV2373 (n=111)*	BNT162b2	D0, D14, D28, D84, D242

*Includes immunology group (n=25)

9.1.8 Stage 2 Group allocations

**2 dose primary**	**Subgroup**	**Booster**	**Visits**
ChAdOx1-nCov19 (n=222)	Ox2A (n=111)	Men ACWY	Day 0, 7*, 14*, 28, 84, 365
	Ox2B (n=111)	Vaccine C	
BNT162b2 (n=222)	Bion2A (n=111)	Men ACWY	Day 0, 7*, 14*, 28, 84, 365
	Bion2B (n=111)	Vaccine C	

* Only applies to the immunology cohort comprising approximately 25 participants from each group

9.1.9 Stage 3 Group allocations

**2 dose primary**	**Subgroup**	**Booster**	**Visits**
ChAdOx1-nCov19 (n=555)	Ox3A (n=111)	Men ACWY	Day 0, 7*, 14*, 28, 84, 365

	Ox3B (n=111)	Vaccine E	
	Ox3C (n=111)	Vaccine F	
	Ox3D (n=111)	Vaccine G	
	Ox3E (n=111)	Vaccine H	
BNT162b2 (n=555)	Bion3A (n=111)	Men ACWY	Day 0, 7*, 14*, 28, 84, 365
	Bion3B (n=111)	Vaccine E	
	Bion3C (n=111)	Vaccine F	
	Bion3D (n=111)	Vaccine G	
	Bion3E (n=111)	Vaccine H	

* Only applies to the immunology cohort comprising approximately 25 participants from each group

9.1.10 Routine NHS deployment of booster vaccination

If a 3rd dose boost vaccine becomes recommended in the UK, we will unblind participants who are in the control arms so that they can receive a 3rd dose of COVID-19 vaccine (no sooner than their day 84 visit) via the study.

As the national booster campaign has been announced by the JCVI and Deputy Chief Medical Officer, all participants will be sent a letter which will either unblind them to allocation to the control arm, or to inform them they have received an active COVID-19 booster vaccine. If they were in the control arm, they will be invited to attend for an additional study visit (UCD0) where they will have baseline bloods taken for safety (FBC and biochem) and immunology (as per Day 0 visit). They will subsequently be randomised to receive a booster vaccination with either BNT162b2, a half dose of BNT162b2, or a half dose of mRNA-1273. Participants will attend follow up visits at day 14 (UCD14) for immunology bloods and for troponin, to monitor for signs of myo/pericarditis on the request of the JCVI and the Public Health England Immunisation Lead. Participants will then attend on day 28 (UCD28) post vaccine for additional immunology bloods. Participants in the Immunology cohort will have additional immunology bloods (see Appendix D) and will provide mucosal immunity samples at UCD0 and UCD28.

Participants will be asked to complete electronic symptom diaries for the 7 days following their booster vaccine for solicited and unsolicited reactions, similar to after their initial D0 visit. They will be asked to enter unsolicited AEs for up to 28 days following vaccination and medically attended AEs for up to 84 days following vaccination.

Participants who have received an active COVID vaccine as 3rd dose in this trial will only be unblinded to receive an additional vaccine if advised by the DMSB, and their schedule of visits will otherwise be unchanged.

9.1.10.1 Eligibility for Unblinded Control Vaccination

Participants in the unblinded control group will be eligible for booster vaccination at the UCD0 visit unless they have had a previous severe adverse reaction to mRNA vaccines or have acquired an additional COVID-19 vaccine outside of the study since enrolling. Other medical criteria will be checked prior to immunisation (including diagnosis of cancer, autoimmune conditions, neurological conditions, blood clotting conditions and pregnancy), but will not be considered a contraindication to vaccination unless the investigator feels there is a specific clinical reason to withhold vaccination for the safety of the participant.

Temporary delay criteria will also be checked including administration of other vaccines within 30 days (or 7 days for influenza vaccines), diagnosis of COVID-19 within 28 days, or current moderate/severe respiratory illness or fever. These are not absolute contraindications to vaccination on the day, and in the presence of these conditions an investigator can decide it is safe to proceed with vaccination if delaying is not possible.

9.1.11 External Vaccine trial participants Sub-Study

Participants in clinical trials for the currently unauthorised vaccine NVXCoV2373 are eligible to receive further vaccinations with authorised COVID-19 vaccines via the NHS in order to qualify for international travel. This sub-study has been set up to generate additional data on the safety, reactogenicity and immunogenicity of receiving a single dose of mRNA vaccine following 2 previous doses of NVXCoV2373.

Participants will be invited to attend a day 0 visit in the same manner as participants in stage 1 of the main Cov-Boost study for screening and vaccination with a single full dose of BNT162b2, with follow up visits at day 14, 28, 84 and then at 6 – 10 months post vaccination (day 242 +/- 60 days). Electronic diaries will be completed in the same manner as the main study.

Participants in this sub-stage of the study will receive one dose from the study team but may choose to receive a second dose via the NHS. Information on receipt of a second dose outside of the trial will be recorded by the study team.

10 Study participants

10.1 Trial Participants

Adults aged 30 years or older who have received a complete homologous 2 dose primary course of COVID-19 vaccination with their first dose of administered in December 2020, January or February 2021.

Recruitment will be from participants aged 30 years and over (including people 75 years and older). Many health care workers will have received BNT162b2 and many adults aged 75 years and older will have received ChAdOx1-nCov19, so recruitment of health care workers who have received ChAdOx1-nCov19 and adults aged 75 years and older who have received BNT162b2 will be particularly encouraged.

Comorbidities of clinical definition mild/moderate/well-controlled will be permitted. Individuals of all ethnicities will be recruited, with recruitment of those identifying as Black and Minority Ethnic particularly encouraged.

The below criteria is inclusion / exclusion criteria for the main study. For inclusion/exclusion criteria of participants taking part in the external vaccine trial sub-study, please see section 10.4.

10.2 Inclusion Criteria

- Participant is willing and able to give written informed consent for participation in the trial.
- Male or Female, aged 30 years or above and in good health as determined by a trial clinician. Participants may have well controlled or mild-moderate comorbidity.
- Female participants of childbearing potential must be willing to ensure that they or their partner use effective contraception from 1 month prior to first immunisation continuously until 3 months after boost immunisation. See Section “Contraception and Pregnancy” for definition of child-bearing potential and definition of effective contraception.
- In the Investigator’s opinion, is able and willing to comply with all trial requirements.
- Willing to allow their General Practitioner and consultant, if appropriate, to be notified of participation in the trial
- Willing to allow investigators to discuss the volunteer’s medical history with their General Practitioner and access all medical records when relevant to study procedures.
- Agreement to refrain from blood donation during the study.
- Received priming dose of COVID-19 vaccination in December 2020, January or February 2021 and is at least 84 days post second vaccination. Due to the NHS deployment timelines, some sites may need to invite people who have been prime-boosted with their second dose of

AstraZeneca with a minimum of 70 days from their second dose. Sites need Sponsor approval for this prior to enrolment of people with a 70-83 day gap since their second dose in any study arm

10.3 Exclusion Criteria

The participant may not enter the trial if ANY of the following apply:

- Receipt of any vaccine (licensed or investigational) other than the study intervention within 30 days before and after each study vaccination (one week for licensed seasonal influenza vaccine or pneumococcal vaccine)
- Prior or planned receipt of any other investigational or licensed vaccine or product likely to impact on interpretation of the trial data (e.g. adenovirus vectored vaccines, any coronavirus vaccines)
- Participants who are pregnant at enrolment or planning to become pregnant during the first 3 months following vaccination.
- Administration of immunoglobulins and/or any blood products within the three months preceding the planned administration of the vaccines
- Any confirmed or suspected immunosuppressive or immunodeficient state; asplenia; recurrent severe infections and use of immunosuppressant medication within the past 6 months, except topical steroids or short-term oral steroids (course lasting ≤14 days)
- History of allergic disease or reactions likely to be exacerbated by any component of study vaccines (e.g. hypersensitivity to the active substance or any of the SmPC-listed ingredients of the Pfizer vaccine)
- Any history of anaphylaxis
- Current diagnosis of or treatment for cancer (except basal cell carcinoma of the skin and cervical carcinoma in situ)
- Bleeding disorder (e.g. factor deficiency, coagulopathy or platelet disorder), or prior history of significant bleeding or bruising following IM injections or venepuncture
- Continuous use of anticoagulants, such as coumarins and related anticoagulants (i.e. warfarin) or novel oral anticoagulants (i.e. apixaban, rivaroxaban, dabigatran and edoxaban)
- History of cerebral venous sinus thrombosis, antiphospholipid syndrome or heparin induced thrombocytopenia and thrombosis (HITT or HIT type 2)
- Suspected or known current alcohol or drug dependency

- Any other significant disease, disorder or finding which may significantly increase the risk to the volunteer because of participation in the study, affect the ability of the volunteer to participate in the study or impair interpretation of the study data
- Severe and/or uncontrolled cardiovascular disease, respiratory disease, gastrointestinal disease, liver disease, renal disease, endocrine disorder and neurological illness (mild/moderate well controlled comorbidities are allowed)
- History of active or previous auto-immune neurological disorders (e.g. multiple sclerosis, Guillain-Barre syndrome, transverse myelitis). Bell's palsy will not be an exclusion criterion
- Significant renal or hepatic impairment
- Scheduled elective surgery during the trial
- Participant with life expectancy of less than 6 months
- Participants who have participated in another research trial involving an investigational product in the past 12 weeks. This does not exclude participants in trials of AZD1222 (ChAdOx1 nCOV-19) who were originally recipients of placebo and who received AZD1222 (ChAdOx1 nCOV-19) or BNT162b2 as part of the "national schedule" with AZD1222 (ChAdOx1 nCOV-19) or BNT162b2 dose 1 from mid-Dec 2020 through end February 2021 and then AZD1222 (ChAdOx1 nCOV-19) or BNT162b2 second dose 12 twelve weeks later (this is allowed by the COV001 and COV002 protocols)
- Insufficient level of English language to undertake all study requirements in opinion of the Investigators except where translation has been able to be provided and is available.

10.3.1 Temporary exclusion criteria

If at Visit 1 Screening & Vaccination the volunteer has any of the following, they will not be enrolled that day.

- Acute respiratory illness (moderate or severe illness with or without fever)

- Fever (oral temperature greater than 37.8°C)

They may be considered for enrolment later in the trial; if they recover in sufficient time.

10.4 Eligibility for External Vaccine Trial Participants Sub-Study

Any participant from the phase 3 study of this vaccine who have not yet received any additional COVID-19 vaccines external to the trial will be eligible providing that they meet the following eligibility criteria:

Inclusion:

- Participant is willing and able to give written informed consent for participation in the trial.
- Male or female aged 30 years or above
- In the Investigator's opinion, is able and willing to comply with all trial requirements.
- Willing to allow their General Practitioner and consultant, if appropriate, to be notified of participation in the trial
- Willing to allow investigators to discuss the volunteer's medical history with their General Practitioner and access all medical records when relevant to study procedures.
- Primary immunization course (2 doses) of NVXCoV2373 COVID-19 vaccine
- Participant plans to receive 1 or 2 doses of licensed vaccine (1 dose will be received as part of this trial, the second dose will not be provided by the trial but the participant may receive a second dose via the NHS if they wish to).

Exclusion:

- Prior receipt of a third dose of COVID-19 vaccine
- History of allergic disease or reactions likely to be exacerbated by any component of the study vaccine (e.g., hypersensitivity to the active substance or any of the SmPC-listed ingredients of the Pfizer vaccine)
- Current diagnosis of or treatment for cancer (except basal cell carcinoma of the skin and cervical carcinoma in situ)
- Any confirmed or suspected immunosuppressive or immunodeficient state; asplenia; recurrent severe infections and use of immunosuppressant medication within the past 6 months, except topical steroids or short-term oral steroids (course lasting ≤14 days)
- Participant with life expectancy of less than 6 months
- Any other significant disease, disorder or finding which in the opinion of the investigator may significantly increase the risk to the volunteer because of participation in the study, affect the ability of the volunteer to participate in the study or impair interpretation of the study data
- Insufficient level of English language to undertake all study requirements in opinion of the Investigators except where translation has been able to be provided and is available.

Other medical criteria will be checked prior to immunisation (including neurological conditions, blood clotting conditions and pregnancy), but will not be considered a contraindication to vaccination unless the investigator feels there is a specific clinical reason to withhold vaccination for the safety of the participant.

Temporary delay criteria will also be checked including administration of other vaccines within 30 days (or 7 days for influenza vaccines), diagnosis of COVID-19 within 28 days, or current moderate/severe respiratory illness or fever. These are not absolute contraindications to vaccination on the day, and in the presence of these conditions an investigator can decide it is safe to proceed with vaccination if delaying is not possible.

11 Trial procedures

See table in appendix A

11.1 Recruitment

11.1.1 Volunteer Identification

Healthy volunteers aged 30 years or older will be recruited through various media. Care will be taken not to recruit from vulnerable groups (mental health or other capacity issues). This will be checked during the screening process.

Volunteers may be recruited by use of an advertisement +/- registration form formally approved by the ethics committee and distributed or posted in the following places:

- On institutional websites where information will be given and the volunteer information sheet will be downloadable or sent to the volunteer upon request
- In public places, including buses and trains, university campus, student bars, halls of residence, health centres etc. with the agreement of the owner / proprietor
- In newspapers or other literature for circulation
- On radio via announcements
- On a website operated by our group or with the agreement of the owner or operator
- As a post on a Twitter, Facebook or other social media account owned and operated by the study research groups
- By email distribution to a group or list only with the express agreement of the network administrator or with equivalent authorisation

- By email distribution to individuals who have already expressed an interest in taking part in any clinical trial at the databases held at study sites.
- On stalls or stands at exhibitions or fairs
- Via presentations (e.g. presentations at lectures or invited seminars)
- Direct mail-out: This will involve obtaining names and addresses of adults via the most recent Electoral Roll. The contact details of individuals who have indicated that they do not wish to receive postal mail-shots would be removed prior to the investigators being given this information. The company providing this service is registered under the Data Protection Act 2018. Investigators would not be given dates of birth or ages of individuals but the list supplied would only contain names of those aged 30 years or older (as per the inclusion criteria).
- NIHR National Vaccine Registry mail-out
- Direct SMS/text message using the NHS vaccine registers
- Via PIC sites and other NHS databases local to sites of those that have received Pfizer and/or AstraZeneca COVID-19 vaccine at the required times for enrolment into the study.
- Volunteers taking part in the external vaccine trial sub-study will be invited to take part by their local external vaccine trial study team. In some cases the COV-Boost study team and external vaccine trial study team will be the same team. However, on occasions where this is not the case, the invite to take part in the study (and participant information sheet if sent at the same time) must be sent by the external vaccine trial team with contact details provided for the COV-Boost study team should the volunteer wish to receive more information or take part.

Volunteers will be sent a copy of the participant information sheet in response to requests for further information. Volunteers will be given a minimum of 24 hours to review the documentation where possible, prior to attending a screening visit. Sufficient time should be given prior to taking informed consent.

11.2 Screening and Eligibility Assessment

11.2.1 Initial screening – Main Trial Only

Once participants express an interest in joining the trial, they will be directed to a 2 stage online screening process, hosted by the University of Oxford. The first stage will assess for obvious exclusion criteria. If they pass this stage they will be asked to indicate their electronic consent to cover:

1) Reporting their medical history (stage 2)

2) Telephone screening visits to review their medical history (if required). Requirement to be determined by review of responses to Part 2 of online questionnaire)
3) Permission to contact the participant's GP for further clarification of past medical history, should this be clinically indicated

Participants without a past medical history or drug history that requires further review may be invited directly to enrolment/vaccination visits.

11.2.2 Telephone screening visit(s) – Main Trial Only

Participants for whom further clarification of eligibility is required, may be invited for telephone screening visit(s), which would then be completed by member(s) of the clinical team, based on the assessment of the part 2 responses. This will be recorded in a screening CRF. This will reduce the amount of time participants have with the clinical team during their screening procedures, should they progress to Visit 1.

We may also contact the subject's general practitioner with the permission of the volunteer. GPs will be notified at the time of enrolment (vaccination) that the subject is taking part in the study.

The interval between the last screening process (whether on-line or by telephone screening) and V1 may be up to a maximum of 120 days. Volunteers will be asked to contact the study team in the interim if there are significant changes to their health status during this time

11.2.3 Screening during Visit 1

The final eligibility assessment and D0 vaccination visit will be combined into Visit 1 (V1). See Section 11.6

11.2.4 Screening: External Vaccine Trial Sub-Study

Informed consent and all screening procedures for participants taking part in the external vaccine trial sub-study will be completed on Day 0 (visit 1).

11.3 Informed Consent

The participant will personally sign and date the latest approved version of the Informed Consent form. A written version and verbal explanation of the Study Information leaflet and Informed Consent will be presented to the participant of the participant detailing:

- The exact nature of the study
- What it will involve for the participant
- The implications and constraints of the protocol

- The known side effects and any risks involved in taking part
- The sample handling protocol – participants will be informed that anonymised samples taken during the study may be shared with study collaborators
- That individual results will not be shared with participants, with the exception of their enrolment COVID-19 antibody test. This would be done at the end of the study, if requested by the participant

The Study Information leaflet will be made available to the participant for an appropriate amount of time (where possible this will be a minimum of 24 hours) prior to consent being obtained. Participants will have the opportunity to individually question an appropriately trained and delegated researcher before signing consent.

The following general principles will be emphasised:

- Participation in the study is entirely voluntary
- Refusal to participate involves no penalty or loss of medical benefits
- The participant may withdraw from the study at any time
- The participant is free to ask questions at any time to allow him or her to understand the purpose of the study and the procedures involved
- That participants will not be sure whether they have received an effective COVID-19 booster vaccination, and whether they may require a further booster vaccination in the future (main study only, this is not applicable for the External Vaccine Trial Sub-Study).
- Participants, like the general population, will not be exempt from following the contemporaneous government COVID-19 guidance to minimise viral transmission
- Samples taken as part of the study may be sent outside of the UK and Europe to laboratories in collaboration with University Hospital Southampton, Public Health England and NISEC collaborating centres. These will be de-identified. Volunteers will be asked if they consent to indefinite storage of any leftover samples for use in other ethically approved research, this will be optional

The participant will be allowed as much time as they wish to consider the information, and the opportunity to question the Investigator, their GP or other independent parties to decide whether they will participate in the study. Written informed consent will then be obtained by means of the participant dated signature, and dated signature of the person who presented and obtained the informed consent. The person who obtained the consent must be suitably qualified and experienced,

and have been authorised to do so by the Chief/Principal Investigator and listed on the delegation log. A copy of the signed informed consent will be given to the participant. The original signed form will be retained at the research study site, in the CRF.

Updated information that requires participants to be re-consented will be sent to participants and written re-consent requested at the earliest scheduled visit. If the earliest visit to occur is in the COVID-19 Pathway (C-19P), the participant may re-consent using an electronic signature for infection control purposes. Where appropriate, and when re-consenting in person is not possible (e.g. participants in self-isolation), participants may be contacted over the phone and an appropriately trained and delegated researcher will obtain re-consent. In this instance the participant will sign the form (electronic or paper) and a copy will be signed by the researcher. The dates of signature may be different, and a copy containing both signatures will be provided to the participant at the next scheduled visit. As the protocol for stage 2 is adaptive, if additional groups are enrolled and stage 2 goes forward participants from stage 1 will not need to be reconsented.

11.4 Randomisation

Computer generated randomisation list will be prepared by the study statistician. In Stage 1 Participants in groups A and C will each be randomised 1:1:1:1 within the 2 cohorts (ChaAdOx1 nCOV-19 and BNT162b2 homologous boost), to the 4 available vaccines in each group using block randomisation. Participants in group B will each be randomised 1:1:1:1:1 within the 2 cohorts (ChaAdOx1 nCOV-19 and BNT162b2 homologous boost), to the 5 available vaccines in each group using block randomisation. Random block sizes of 4 or 8 will be used in group A and C, and random block sizes of 5 or 10 will be used in group B. The randomisation will be stratified by the study sites and by age (<70 and >=70). Although we are not aiming for a formal balance in recruitment of participants over or under the age of 70, this will ensure the recruited population are distributed evenly across all treatment arms within each age group.

Following the announcement of an NHS vaccination campaign, the control group will be unblinded to their allocation and invited to attend for a COVID-19 booster vaccination via the study. Participants in all 3 control groups will be randomised 1:1:1 within the 2 cohorts (ChaAdOx1 nCOV-19 and BNT162b2 homologous boost) to one of the 3 available vaccines: BNT162b2, half dose BNT162b2, or half dose mRNA-1273. Random block sizes of 3 or 6 will be used. The randomisation will be stratified by study sites and by age (<70 and >=70).

Participants in the External Vaccine Trial sub-study will not be randomised. The only vaccine to be used in this part of the study is BNT162b2.

Participants in Stage 2 will be randomised 1:1 within the 2 groups (ChaAdOx1 nCOV-19 and BNT162b2 homologous boost) to either Vaccine C or Men ACWY. Random block sizes of 2 or 4 will be used. The randomisation will be stratified by the study sites. Additional groups may be available for randomisation.

In Stage 3 Participants will be randomised 1:1:1:1:1 within the 2 groups (ChaAdOx1 nCOV-19 and BNT162b2 homologous boost), to Vaccine E, Vaccine F, Vaccine G, Vaccine H or Men ACWY using block randomisation. Random block sizes of 5 or 10 will be used. The randomisation will be stratified by the study sites.

11.5 Blinding and code-breaking

Participants and laboratory staff will remain blind to treatment allocation. Statisticians were blinded during the recruitment phase and for interim analysis up until the Statistical analysis plan (SAP) was signed off. Statisticians will be blinded for future stages or sub-stages after sign off of SAP V1.0. Clinical staff involved in study delivery will be aware of which vaccine the participant is receiving (arm allocation); the participants themselves will remain blinded to their vaccine allocation. Vaccines will be prepared out of sight of the participant and the blind will be maintained by applying a masking tape over the vaccine syringe.

If the clinical condition of a participant necessitates unblinding of the participant, this will be undertaken according to a trial specific working instruction and group allocation sent to the attending physician. This will be done if unblinding is thought to be relevant and likely to change clinical management.

Following the announcement of a national NHS booster vaccination campaign, participants will be unblinded to their allocation to the control arm, or one of the active COVID-19 vaccines (however, the specific COVID-19 vaccine will not be revealed until the end of the study). From this point onwards participants and investigators will be unblinded to the allocation of the control group.

Participants in the External Vaccine Trial sub-study will not be blinded to vaccine allocation. The only vaccine to be used in this part of the study is BNT162b2.

11.6 Visits

The study visits and procedures will be undertaken by one of the clinical trials team. The procedures to be included in each visit are documented in the schedule of attendances (see Appendix A for schedule of attendances). Each visit is assigned a time-point and a window period, within which the visit will be conducted. If a participant cannot attend a visit, where possible, this will be re-arranged to an in-person visit within the time window. A telephone visit may be conducted instead of the in-

person visit to ascertain as much relevant information as possible if the participant is unable to attend a visit in person because of quarantine or self-isolation restrictions and the participant will be out of window if the visit is postponed.

11.6.1 Visit 1 (D0): Final eligibility check, Enrolment and Vaccination visit

11.6.1.1 Informed consent

The participant will have informed consent taken as described in Section 11.3, before proceeding to the final eligibility check Component of V1. Individually, each volunteer will have the opportunity to question an appropriately trained and delegated researcher before signing the consent.

11.6.1.2 Final Eligibility Check V1

During the final eligibility check component of Visit 1 (V1):

If written consent is obtained, the procedures indicated in the schedule of attendances will be undertaken including:

- Confirmation of medical history
- Physical examination (if required)
- Height and weight
- Blood tests including:
    - COVID-19 immunogenicity bloods
    - Baseline bloods for safety monitoring (routine haematology & biochemistry tests)
- Nasal fluid sample
- Saliva sample (where available)
- Observations (temperature, heart rate, respiratory rate, blood pressure and oxygen saturation)
- Urine pregnancy test in females of childbearing potential

The eligibility of the volunteer will be reviewed by a suitable member of the clinical team. Decisions to exclude the volunteer from enrolling in the trial or to withdraw a volunteer from the trial will be at the discretion of the Investigator. Note that the blood tests results from this visit will not ordinarily be available at the time the decision to proceed to immunisation with these vaccines is made. Instead, these blood tests will act as a baseline assessment for any subsequent derangements of laboratory measures. Abnormal clinical findings from blood tests at screening will be assessed by a medically qualified study member. Where available, these may be compared to blood test results taken prior to the trial as part of the participant's normal medical care, to ascertain if the derangement is an acute abnormality or is a chronic change. Abnormal blood tests following screening will be assessed

according to site-specific laboratory adverse event grading tables. Any abnormal test result deemed clinically significant may be repeated to ensure it is not a single occurrence. If an abnormal finding is deemed to be clinically significant, the volunteer will be informed and appropriate medical care arranged with the permission of the volunteer.

As per Section “Temporary exclusion criteria”: If a volunteer has an acute respiratory illness (moderate or severe illness with/without fever) or a fever (oral temperature > 37.8°C) at Visit 1 Screening, the volunteer will not be enrolled that day, but may be considered for enrolment at a later date if they recover in sufficient time. For control participants receiving a booster and for external vaccine trial sub-study participants this temporary exclusion criteria is not a direct contraindication to vaccination.

11.6.1.3 Vaccination at V1

Volunteers will be considered enrolled to the trial at the point of vaccination. All vaccines will be administered intramuscularly according to the IMP Handling Manual, applicable COV-Boost Preparation Guide and individual IMP pharmacy manual (if applicable). The participant will stay in the trial site for observation for at least 15 minutes, in case of immediate adverse events. Photographs of vaccination sites may be taken, if required (with the participants’ written, informed consent) and will not include the participants’ face. Photographs will be identified by date, trial code and subject’s unique identifier.

11.6.1.4 Diary cards

Participants will be given an oral thermometer, tape measure and diary card (electronic, but for those who are unable to use electronic diary cards, a paper version will be made available), with instructions on use. All participants will be given the emergency 24 hour telephone number to contact the on-call study physician if needed. Participants will be instructed on how to self-assess the severity of these AEs. There will also be space on the diary card to self-document unsolicited AEs, and whether medication was taken to relieve the symptoms. There will also be a separate e-diary to log any medically attended AEs up until 3 months post vaccination (any medical conditions for which a doctor/dentist is seen outside of routine, planned follow-up), and any serious medical illnesses or hospital visits may have occurred over the entire course of the study. Participants will be asked to report on solicited AEs for 7 days (and longer if symptoms persist at day 7, until resolution or stabilisation of symptoms) and unsolicited AEs for 28 days. Diary cards will collect information on the timing and severity of the following solicited AEs:

Table 1. Solicited AEs collected on post vaccination diary cards

**Local solicited AEs**	Pain, Redness, Warmth, Itch, Swelling, Induration

**Systemic solicited AEs**	Fever, Feverishness, Chills, Joint pains, Muscle pains, Fatigue, Headache, Malaise, Nausea, Vomiting, Diarrhoea

Post-vaccination diary cards will be reviewed at the next scheduled study visit. The local study team will receive electronic alerts for any Grade 3 or above AEs entered into diaries, which will be monitored daily. Participants may be telephoned to discuss further, should there be any clinical concerns.

Participants will also be instructed on the use of the Medically Attended Diary Card. They will be asked to record the following healthcare encounters up until 3 months post booster dose:

- GP visits that were not planned or routine
- Attendances at A&E
- Unplanned outpatient visits to hospital e.g. attending an "Ambulatory Care" unit
- Non-routine dental visits (i.e. dental emergency)

This information will be reviewed routinely only at follow up visits. The diary card will contain an instruction to contact the trial team by telephone should any encounter be a hospitalisation, or if they have concerns about their health.

Participants entering the COVID-19 pathway will also be asked to complete a diary, see section 11.6.5 below.

11.6.2 Safety review

While there will be no planned safety pause, a review of reactogenicity data will be conducted after the initial 20 (approximately) participants per arm have received their trial dose in stage 1, stage 2, and stage 3. In case that the 3 groups in stage 1 will not start recruiting at the same time, the review will be conducted separately for the 3 groups. This will assess reactogenicity in the first 48 hours after immunisation, with data presented by 'anonymised' group to the DSMB chair. Should significant safety concerns arise at this point a full DSMB meeting will be organised.

11.6.3 Subsequent visits

Follow-up visits will take place as per the schedule of attendances described in Appendix A. Participants will be assessed for local and systemic adverse events, interim history, review of diary cards (paper or electronic) and blood fluid/nasal fluid/saliva tests at these time points as detailed in the schedule of attendances. Additional information may be collected such as days off work following the study vaccine, any days requiring extra help or support and extra medical consultations due to side effects. Blood will also be taken for immunology purposes. Observations and physical exam will be performed as and when clinically indicated.

If participants experience adverse events (laboratory or clinical), which the investigator (physician), CI and/or DSMB chair determine necessary for further close observation, the participant may be admitted to an NHS hospital for observation and further medical management under the care of the Consultant on call.

11.6.4 Participants under quarantine

Given the evolving epidemiological situation both globally and in the UK, should a participant be unable to attend any of their scheduled or unscheduled visits, a telephone consultation will be arranged to obtain core study data where possible. Participants should not attend for in-person visits if they are in their period of self-isolation/quarantine – the exception to this is the COVID-19 Pathway.

11.6.5 Participants with confirmed SARS-CoV-2 infection (COVID-19 Pathway)

Participants will be counselled at enrolment that should they receive a positive SARS-CoV-2 test (e.g. an antigen detection or nucleic acid amplification test, for example, via test and trace or occupational health services) they should contact the trial team on receipt of the positive result. Participants will be reminded of this with a weekly text/email message (participant choice), which will commence after the first vaccine dose.

This COVID-19 (C-19) pathway will apply to participants tested via symptomatic and asymptomatic pathways.

Participants enrolled in the external vaccine trial sub-study will not need to attend COVID-19 pathway visits but positive SARS-CoV-2 tests will be documented by the study team.

Once the participant has conveyed their result to the study team, and the study team confirm an appropriate test has been used (verbal discussion with participant as to how testing was obtained, confirmatory documentation will not be sought), an appointment will be arranged to review the participant at the relevant study site. At this visit blood samples for safety (FBC, Biochemistry, CRP and others if deemed clinically relevant) and immunology (PBMCs and serum for cellular and humoral immune responses) will be taken, along with a nasopharyngeal swab for storage and subsequent viral isolation. Nasal fluid and saliva (where available) for mucosal immune response will be taken for those who receive this at their regular study visits. Vital signs and other clinical data will be recorded. Participants will also be provided with a symptom diary, which they will fill in both solicited and unsolicited symptoms for at least 7 days and until symptom resolution (excepting persistent cough and anosmia/dysgeusia, as these are recognised to be able to continue for extended periods). Additional visits on this pathway may be arranged at the clinical discretion of the investigator.

Participants will only be invited to a C-19P visit if they have access to private transport and would not require assistance to attend the visit. Participants may not attend the visit using public transport or taxis.

The windows for performing this visit are as per Table 2.

Table 2. C-19P visit windows

Visit booking P0	Margin
**Within 7 days of positive test**	0 – 7 days

Participants should be screened for severity of disease on contacting the trial team with their positive result and referred to NHS care as appropriate.

Table 3. Remote risk stratification of COVID-19 infection

Severity of illness	Features	Advice and action
**Mild**	Completing full sentences	Paracetamol for fever
	No SOB (Grade 0)	Can use NSAIDs according to NHS recommendations (advise lowest dose and shortest duration possible)
	No chest tightness (Grade 0)	Regular fluids
	Able to do ADLS (Grade 0-1)	Self-isolate as per current government guidelines
	RR 12-20	Safety net re worsening symptoms:<br>- Trial doctor for advice in hours (999 in an emergency)<br>- 111 out of hours (non-emergent)
	No other red flags/concerning features from history	Paracetamol for fever
**Moderate A**	Completing full sentences	Can use NSAIDs according to NHS recommendations (advise lowest dose and shortest duration possible)
	Able to do ADLs but lethargic (Grade 1-2)	Regular fluids
	Mild chest tightness (Grade 1)	Self-isolate as per current government guidelines
	Mild SOB on exertion only (Grade 1)	Safety net re worsening symptoms:<br>- Trial doctor for advice in hours (999 in an emergency)<br>111 out of hours (non-emergent)
	RR 12-20 (if can be observed)	
	Any symptoms from other systems considered to be moderate and not requiring medical review	
	No other red flag features from history	
**Moderate B**	Completing full sentences	For medical review<br>- Trial doctor to arrange medical review with a non-trial medical practitioner e.g. GP or hospital doctor (in-hours)<br>- Trial doctor to signpost to NHS services (out-of hours)

	Able to do ADLs but lethargic (Grade 1-2)	Safety net – 999 if worsening beyond current symptoms
	Mild chest tightness (Grade 1-2)	Inform senior on-call clinician
	Mild SOB on exertion only (Grade 1)	
	RR 20-24 (if can be observed)	
	Any symptoms from other systems considered to be moderate and requiring medical review	
**Severe**	Any one of:	Urgent medical review
	Inability to complete full sentences	Advise participant to call 999
	Unable to do any ADLs/get out of bed (Grade 3)	Inform senior on-call clinician
	RR >25 if can be observed	
	Any other clinical concerns for severe disease	
**Of note, this is not an all-encompassing guide and individual clinical judgement by reviewing clinician should always be taken into account. Should the reviewing clinician have any concerns regardless of risk stratification then they can contact the appropriate senior clinician for further advice.**		

11.6.6 Admission of participants to hospital with COVID-19 infection

With the participant's consent, the study team will request access to medical notes or submit a data collection form for completion by attending clinical staff on any COVID-19 episodes resulting in hospitalisation. Any data which are relevant to ascertainment of efficacy endpoints and disease enhancement will be collected. These are likely to include, but not limited to, information on ICU admissions, clinical parameters such as oxygen saturation, respiratory rates and vital signs, need for oxygen therapy, need for ventilatory support, imaging and blood tests results, amongst others.

11.7 Sample Handling

Please refer to Appendix D: Blood Sampling for schedule of frequency and volume of blood sampling.

11.7.1 Sample handling for trial purposes

11.7.1.1 Immunology blood tests

Immunogenicity will be assessed by a variety of immunological assays. This will include antibodies to SARS-CoV-Spike and non-Spike antigens by ELISA, ex vivo ELISpot assays for interferon gamma and flow cytometry assays, neutralising and other functional antibody assays. Other exploratory immunological assays including cytokine analysis and other antibody assays, DNA analysis of genetic polymorphisms potentially relevant to vaccine immunogenicity and gene expression studies amongst others may be performed at the discretion of the Investigators.

Collaboration with other specialist laboratories in the UK, Europe and outside of Europe for further exploratory tests may occur. This would involve the transfer of serum, plasma, PBMC and/or other

study samples to these laboratories, but these would remain anonymised. The analyses and which laboratories carry these out will be specified in the laboratory analysis plan.

Subjects will be informed that there may be leftover samples of their blood (after all testing for this study is completed), and that such samples may be stored indefinitely for possible future research (exploratory immunology), including genotypic testing of genetic polymorphisms potentially relevant to vaccine immunogenicity. Subjects will be able to decide if they will permit such future use of any leftover samples. With the participants' informed consent, any leftover cells and serum/plasma will be frozen indefinitely for future analysis of COVID-19 and other coronaviruses related diseases or vaccine-related responses. If a subject elects not to permit this, all of that participants' leftover samples will be discarded at the end of the trial.

Samples that are to be stored for future research will be transferred to the National Biosample Centre.

11.7.1.2 Nasal fluid & saliva samples

An exploratory analysis of mucosal immunity will be conducted using nasal fluid collected at Day 0, 28 and 365 in the immunology cohort (n=approximately 650), using SAM-strips (synthetic absorptive matrix) and, where available, saliva samples. All participants who have been enrolled to groups who will have SAM-strip and saliva sampling (where available) will also have SAM-strips and saliva taken at the C19P visit if they attend this visit. Analysis will be conducted initially with IgA and IgG ELISAs, with further exploratory immunology assays conducted based on results – more detail will be included in the laboratory analysis plan. The same statements regarding collaboration, storage and use of samples as for blood in Section 11.7.1.1 apply here. Participants in the immunology cohort of the external vaccine trial sub-study will not have nasal fluid and saliva samples taken.

11.7.1.3 Nasopharyngeal swabs

Participants seen in the C-19 pathway will have nasopharyngeal swabs taken (instructions on performing sampling in CSP). These swabs will be tested for presence of the SARS-Cov-2 virus centrally. This analysis is for research purposes, and will not be conducted in 'real-time', so will not be used to inform the requirements for participant self-isolation etc. Swabs, and/or samples obtained from them, will be stored for potential further analysis (e.g. whole genome sequencing of identified SARS-CoV-2).

11.7.2 Sample handling for standard of care

Urinary pregnancy testing

For female participants of child bearing potential only, urine will be tested for beta-human chorionic gonadotrophin (β-HCG) at screening and again immediately prior to booster vaccination. This will be a point of care test and no sample will be stored.

11.7.2.1 Safety monitoring blood tests

These will be processed at agreed NHS Trust laboratories, and destroyed in accordance with standard NHS processes. They will include:

- **Haematology** – Full Blood Count
- **Biochemistry** – Sodium, Potassium, Urea, Creatinine, Albumin, Liver Function Tests (ALT, ALP, Bilirubin) and if relevant C-reactive protein (CRP)

11.8 Early Discontinuation/Withdrawal of Participants

In accordance with the principles of the current revision of the Declaration of Helsinki and any other applicable regulations, a participant has the right to withdraw from the study at any time and for any reason, and is not obliged to give his or her reasons for doing so. The Investigator may withdraw the participant at any time in the interests of the participants' health and well-being. In addition, the participant may withdraw/be withdrawn for any of the following reasons:

- Administrative decision by the Investigator
- Ineligibility (either arising during the study or retrospectively, having been overlooked at screening).
- Significant protocol deviation
- Participant non-compliance with study requirements
- An AE, which requires discontinuation of the study involvement or results in inability to continue to comply with study procedures

The reason for withdrawal will be recorded in the CRF. If withdrawal is due to an AE, appropriate follow-up visits or medical care will be arranged, with the agreement of the volunteer, until the AE has resolved, stabilised or a non-trial related causality has been assigned. The DSMB or DSMB chair may recommend withdrawal of participants.

If a participant withdraws from the study, storage of samples will continue unless the participant specifically requests otherwise. Any data collected before their withdrawal will still be used in the analysis for safety and trial integrity; if the participant requests this could be de-identified following the end of the study.

In cases of subject withdrawal, long-term safety data collection, including some procedures such as safety bloods, may continue as appropriate if subjects have received one or more vaccine doses, unless they decline any further follow-up.

11.9 Definition of End of Trial

The end of the trial is the date of the last assay conducted on the last sample collected.

12 Trial Interventions

12.1 Investigational Medicinal Product(s) (IMP) Description

The marketing authorisation status of the vaccines included here is that the ChAdOx1-nCOV-19, BNT162b2, mRNA-1273 and Ad26.COV2.S vaccines are approved for use under a temporary authorisation of the supply of an unlicensed vaccine; regulation 174 of the Human Medicines Regulations 2012. For these vaccines there will not be IMP labelling for this trial where there is Regulation 174 approval and products will be used as supplied by manufacturer (as for national supply) and blinding performed as per section 11.5. This section will be revised with submission of the next substantial amendment if additional vaccines receive Regulation 174 approval during the trial period.

12.1.1 AstraZeneca COVID-19 vaccine (ChAdOx1 nCOV-19)

ChAdOx1 nCoV-19 is a recombinant replication-defective chimpanzee adenovirus expressing the SARS-CoV-2 spike (S) surface glycoprotein with a leading tissue plasminogen activator (TPA) signal sequence. S is a type I, trimeric, transmembrane protein located at the surface of the viral envelope, giving rise to spike shaped protrusions from the virion. The S proteins subunits are responsible for cellular receptor ACE-2 binding via the receptor-binding domain and fusion of virus and cell membranes, thereby mediating the entry of SARS-CoV-2 into the target cells. The S protein has an essential role in virus entry and determines tissue and cell tropism, as well as host range.

ChAdOx1 nCoV-19 expresses a codon-optimised coding sequence for Spike protein from the SARS-CoV-2 genome sequence accession MN908947. ChAd is a non-enveloped virus, and the glycoprotein antigen is not present in the vector, but is only expressed once the genetic code within the vector enters the target cells. The vector genes are also modified to render the virus replication incompetent, and to enhance immunogenicity. Once the vector is in the nucleus, mRNA encoding the spike protein is produced that then enters the cytoplasm. This then leads to translation of the target protein which act as an intracellular antigen

12.1.1.1 Dosage, scheduling and packaging

The dose of AstraZeneca COVID-19 vaccine is $5x10^{10}$vp in 0.5ml. The vaccine should be administered intramuscularly. The AstraZeneca vaccine is supplied in packs of 10 vials. Each vial contains 8 or 10 doses of vaccine, and is a colourless to slightly yellow, clear to slightly opaque liquid. Each dose is prepared by withdrawing 0.5 mL from a vial in a sterile 1 mL or equivalent syringe.

12.1.2 Pfizer BioNTech (BNT162b2)

BNT162b2 is a lipid nanoparticle-formulated, nucleoside-modified mRNA vaccine that encodes trimerised SARS-CoV-2 spike glycoprotein. BNT162b2 encodes the SARS-CoV-2 full-length spike, modified by two proline mutations to lock it in the prefusion conformation and more closely mimic the intact virus with which the elicited virus-neutralizing antibodies must interact. mRNA vaccines use the pathogen's genetic code as the vaccine; this then exploits the host cells to translate the code and then make the target spike protein. The protein then acts as an intracellular antigen to stimulate the immune response. The mRNA is then degraded within days. The vaccine RNA is formulated in lipid nanoparticles (LNPs) for more efficient delivery into cells after intramuscular injection.

12.1.2.1 Dosage, scheduling and packaging

The dose of Pfizer BioNTech COVID-19 vaccine is 30µg contained in 0.3ml of the diluted vaccine. A half dose is 15 µg contained in 0.15ml of the diluted vaccine. Each pack of the Pfizer BioNTech vaccine contains 195 vials with 5 doses per vial (975 doses per pack). It is supplied with 0.9% sodium chloride diluent for injection plastic ampoules.

A half dose has been included at the request of the UK Chief Medical Office and Vaccine Task Force to investigate as potential dose sparing regimen for use in the UK or globally due to potential shortage of vaccine supply.

12.1.3 Moderna (mRNA-1273)

COVID-19 Vaccine Moderna (mRNA-1273) encodes the S-2P antigen, consisting of the SARS-CoV-2 glycoprotein with a transmembrane anchor and an intact S1–S2 cleavage site. S-2P is stabilized in its prefusion conformation by two consecutive proline substitutions at amino acid positions 986 and 987, at the top of the central helix in the S2 subunit. The lipid nanoparticle capsule is composed of four lipids, and formulated in a fixed ratio of mRNA and lipid.

12.1.3.1 Dosage, scheduling and packaging

The dose of Moderna COVID-19 vaccine is 0.10mg of mRNA in 0.5ml of the diluted vaccine. Each pack of the Moderna vaccine contains 10 vials with 10 doses per vial (100 doses per pack) and is a white to off white dispersion. Each dose is prepared by withdrawing 0.5ml from a vial in a sterile 1ml or equivalent syringe.

Moderna have applied to the US FDA and European Medicines Agency for a homologous 3rd dose to be of 0.05mg mRNA. Because of this and reactogenicity profiles seen in COV-BOOST stage 1 (publication in preparation, data seen by MHRA and JCVI) the JCVI, Deputy Chief Medical Officer for England and Head of Immunisation at Public Health England (co-Investigator MR) have recommended

that half dose (0.05mg mRNA) be tested in COV-BOOST as a late boost in people previously given control vaccine.

12.1.4 Novavax (NVXCoV2373)

Novavax, NVXCoV2373 is a nano-particle vaccine. It is constructed from the full-length wild-type (prototype Wuhan sequence) pre-fusion trimers of SARS-CoV2 spike glycoprotein. The native protein has been modified with several substitutions to limit protease cleavage and enhance thermal stability (the putative native furin cleavage site has been modified from RRAR to QQAQ and 2 proline substitutions (positions K986P and V987P) in the HR1 domain). It has also been optimised for expression in insect (Spodoptera frugiperda) Sf9 cells. The recombinant S-protein genes are cloned into a baculovirus vector before being transferred into Sf9 cells. These cells then produce the protein which is extracted and purified and arranged into nanoparticles. It is co-formulated with a saponin-based adjuvant, Matrix-M1™.

12.1.4.1 Dosage, scheduling and packaging

The dose of NVXCoV2373 is 5 µg recombinant spike protein with 50 µg Matrix-M1 adjuvant (0.5ml). The vaccine is supplied in 10 dose vials. A half dose will be drawn up at 0.25ml per dose.

A half dose has been included at the request of the UK Chief Medical Office and Vaccine Task Force to investigate as potential dose sparing regimen for use in the UK or globally due to potential shortage of vaccine supply.

12.1.5 Valneva (VLA2001)

VLA2001 is a highly purified, whole virus, SARS-CoV-2 vaccine produced on serum-free Vero cells and inactivated with β propiolactone. The viral strain is derived from a Chinese tourist diagnosed in a hospital in Rome. VLA2001 is adjuvanted with the licensed adjuvant cytosine phosphor-guanine (CpG) 1018 in combination with Aluminium Hydroxide.

12.1.5.1 Dosage, scheduling and packaging

The dose of VLA2001 is 33 antigen units/dose of SARS-CoV-2 inactivated with β propiolactone with 0.5mg aluminium hydroxide and 1mg CpG 1018 in 0.5ml. Each dose is prepared by withdrawing 0.5ml from a vial in a sterile 1ml or equivalent syringe. The vaccine is supplied in 10 dose vials. A half dose will be drawn up at 0.25ml per dose.

A half dose has been included at the request of the UK Chief Medical Office and Vaccine Task Force to investigate as potential dose sparing regimen for use in the UK or globally due to potential shortage of vaccine supply.

12.1.6 Curevac (CVnCoV)

CVnCoV is an mRNA based SARS-CoV-2 vaccine encapsulated in lipid nanoparticles. The mRNA encodes a pre-fusion conformation stabilised version of the full-length Spike protein of the SARS-CoV-2 virus. The active substance is R9515, incorporating amino acid substitutions aimed to stabilise the encoded protein in the prefusion conformation, i.e. Lysine to Proline at position 986 and Valine to Proline at position 987.

12.1.6.1 Dosage, scheduling and packaging

The dose of CVnCoV is 12µg in 0.6ml of the diluted vaccine. The vaccine is supplied in 12 dose vials. It is supplied with 0.9% sodium chloride diluent for injection plastic ampoules.

12.1.7 Janssen (Ad26.COV2.S)

Ad26.COV2.S is a monovalent vaccine composed of a recombinant, replication-incompetent Ad26 vector, constructed to encode a membrane-bound full-length SARS-COV-2-S protein incorporating 2 amino acid changes in the S1/S2 junction that knock out the furin cleavage site, and 2 proline substitutions in the hinge region.

12.1.7.1 Dosage, scheduling and packaging.

Ad26.COV2.S will be supplied at a concentration of $1x10^{11}$ vp/mL as a suspension in single-use vials, with an extractable volume of 0.5 mL. $5x10^{10}$ vp dose level: 0.5 mL is withdrawn from one vial containing 0.69 mL $1x10^{11}$ vp/mL.

12.1.8 Control vaccine

Participants who are allocated to the control groups will receive an injection of MenACWY. One of two licensed quadrivalent protein-polysaccharide conjugate vaccine MenACWY vaccines will be used, with Nimenrix being considered first choice. Menveo should only be used in the event that a study site is unable to obtain supplies of Nimenrix.

Nimenrix (Pfizer). The licensed posology of this vaccine for those over 6 months of age is a single (0.5ml) intramuscular dose, containing 5mcg each of *Neisseria meningitidis* group A, C, W and Y polysaccharide, each conjugated to 44 mcg tetanus toxoid carrier protein.

Menveo (GlaxoSmithkline). The licensed posology of this vaccine for those 2 years of age and over is a single (0.5ml) intramuscular dose, containing

- 10 mcg meningococcal group A polysaccharide, conjugated to 16.7 to 33.3 mcg *Corynebacterium diphtheriae* CRM_{197} protein

- 5mcg meningococcal group C polysaccharide, conjugated to 7.1 to 12.5 mcg *C. diphtheriae* CRM_{197} protein
- 5mcg meningococcal group W polysaccharide, conjugated to 3.3 to 8.3 mcg *C. diphtheriae* CRM_{197} protein
- 5mcg meningococcal group Y polysaccharide, conjugated to 5.6 to 10.0 mcg *C. diphtheriae* CRM_{197} protein

Previous receipt of either vaccine (or a plain polysaccharide quadrivalent meningococccal A, C, W and Y vaccine) will not be a contraindication to receiving a further vaccine in this study.

12.1.8.1 Dosage, scheduling and packaging

Doses of the two vaccines are as described above, with a single dose administered in 0.5ml by intramuscular injection. There will be no additional labelling of these vaccines beyond their licensed packaging.

12.1.9 Blinding of IMPs

See Section "Blinding and codebreaking" for detail.

12.1.10 Storage of IMP

Vaccines will be stored in accordance with manufacturers' recommendations.

All movements of the study vaccines will be documented in accordance with existing standard operating procedure (SOP). Vaccine accountability, storage, shipment and handling will be in accordance with relevant SOPs and forms. To allow for participants to receive the vaccine in a short time period, additional clinic locations may be used. In this instance vaccines will be transported in accordance with local SOP's and approvals as required.

12.1.10.1 AstraZeneca COVID-19 vaccine (ChAdOx1 nCOV-19)

The AstraZeneca vaccine should be stored at +2ºC to +8ºC and has a shelf life of 6 months. The vaccine does not contain any preservative. After first opening the vial, it should be used within 6 hours when stored at room temperature (up to 30º C) or within 48 hours when stored in a refrigerator (2 to 8º C [36 to 46°F]). After this time, the vial must be discarded. The total cumulative storage time once opened must not exceed 48 hours.

12.1.10.2 Pfizer BioNTech (BNT162b2)

The Pfizer BioNTech vaccine should be stored at -70°C +/- 10°C and has shelf life of 6 months. Once thawed, the vaccine may be stored for 31 days at 2-8°C.

12.1.10.3 Moderna (mRNA 1273)

The COVID-19 Vaccine Moderna can be stored for 7 months at -25ºC to -15ºC. It should not be stored or transported on dry ice or below -40ºC. Once thawed it should not be re-frozen and may be stored refrigerated at 2 °C to 8 °C protected from light for up to 30 days if not used (needle-punctured). Chemical and physical stability of an unopened vial after removal from refrigerated conditions has been demonstrated for 24 hours at 8° to 25°C. Chemical and physical in-use stability has been demonstrated for 6 hours at 2 to 25 ºC after first puncture. It should not be re-frozen once thawed.

12.1.10.4 Novavax (NVXCoV2373)

SARS-CoV-2 rS and Matrix-M1 adjuvant should be stored at 2°C to 8°C and not frozen.

12.1.10.5 Valneva (VLA2001)

VLA2001 should be refrigerated at +2°C to +8°C and not frozen. The punctured vial should not exceed 6 hours independent of whether at room temperature or refrigerated.

12.1.10.6 Curevac (CVnCoV)

CVnCoV should be stored at -60°C or below. The diluted IMP can be stored for a maximum of 16 hours at 5 ± 3°C in the mixing vial, and subsequently stored in the syringe for a maximum of 2 hours at room temperature.

12.1.10.7 Janssen (Ad26.COV2.S)

Ad26.Cov2.S should be stored between -25°C and – 15°C

Once removed from the freezer, the unopened vaccine may be stored refrigerated at 2°C to 8°C, protected from light, for a single period of up to 28 days, not exceeding the printed expiry date (EXP).

Once thawed, the vaccine should not be re-frozen. The product can be stored between 2°C-8°C for a maximum of 6 hours or remain at room temperature (maximally 25°C) up to 3 hours after first puncture of the vial.

12.1.10.8 Control Vaccine

MenACWY should be refrigerated at +2°C to +8°C and not frozen.

12.1.11 Compliance with Trial Treatment

All vaccinations will be administered by the research team and recorded in the CRF. The study medication will be at no time in the possession of the participant and compliance will not, therefore, be an issue.

12.1.12 Accountability of the Trial Treatment

Accountability of the IMPs will be conducted in accordance with the IMP Handling Manual and IMP Management Plan.

12.1.13 Concomitant Medication

As set out by the exclusion criteria, volunteers may not enter the study if they have received: any vaccine other than the licensed seasonal influenza vaccine or pneumococcal vaccine in the 30 days prior to enrolment or there is planned receipt of any other vaccine within 30 days of each vaccination, any investigational product within 30 days prior to enrolment or if receipt is planned during the study period, or if there is any use of immunosuppressant medication within 6 months prior to enrolment or if receipt is planned at any time during the study period (except topical steroids and short course of low dose steroids < 14 day). Concomitant medications taken at enrolment will be recorded, as will new medications taken within the 28 days after each immunisation. Subsequently only new medications taken in response to a medically attended adverse event up until 3 months post boost will be recorded.

12.1.14 Post-trial Treatment

If any of the booster vaccinations are deselected as 3rd dose boost candidates as a result of the 28 day immunogenicity data and the participant is eligible for a booster vaccination via the NHS, they may be offered an additional vaccine which has been demonstrated to elicit an acceptable rise in the primary immunogenicity analysis. Decisions regarding the need for a booster dose, the nature of the booster dose and mode of delivery (e.g. NHS vs study site) will be made in consultation with the DSMB and study management group and will take into account national JCVI advice.

12.2 Other Treatments (non-IMPS)

Participants will be advised that they may take paracetamol prophylactically after vaccine administration. This will be from the participants own supplies rather than supplied by the study team.

12.3 Other Interventions

There are no additional investigations other than those specified in this protocol.

13 Safety Reporting

13.1 Safety reporting window

Safety reporting for the trial will commence once the first participant is consented; and will end 12 months after the last participant has received the first dose of an IMP for SAEs and Adverse Events of Special Interest (AESI)s.

For individual participants the reporting period begins when they are consented, in person, at the V1 visit , and ends 12 months after the first dose of vaccine for SAEs and AESIs.

All adverse events (AEs) that result in a participants' withdrawal from the study will be followed up until a satisfactory resolution occurs, or until a non-study related causality is assigned (if the participant consents to this).

13.2 Adverse Event Definitions

**Adverse Event (AE)**	Any untoward medical occurrence in a participant to whom a medicinal product has been administered, including occurrences which are not necessarily caused by or related to that product.
**Adverse Reaction (AR)**	An untoward and unintended response in a participant to an investigational medicinal product which is related to any dose administered to that participant.<br><br>The phrase "response to an investigational medicinal product" means that a causal relationship between a trial medication and an AE is at least a reasonable possibility, i.e. the relationship cannot be ruled out.<br><br>All cases judged by either the reporting medically qualified professional or the Sponsor as having a reasonable suspected causal relationship to the trial medication qualify as adverse reactions.
**Adverse Events of Special Interest (AESI)**	Adverse events identified as being of particular relevance to the IMP's. These will also reported as an SAE, if meeting SAE criteria (e.g. hospitalisation)
**Serious Adverse Event (SAE)**	A serious adverse event is any untoward medical occurrence that:<br>• Results in death<br>• Is life-threatening

	• Requires inpatient hospitalisation or prolongation of existing hospitalisation<br>• Results in persistent or significant disability/incapacity<br>• Consists of a congenital anomaly or birth defect*<br><br>Other 'important medical events' may also be considered a serious adverse event when, based upon appropriate medical judgement, the event may jeopardise the participant and may require medical or surgical intervention to prevent one of the outcomes listed above.<br><br>NOTE: The term "life-threatening" in the definition of "serious" refers to an event in which the participant was at risk of death at the time of the event; it does not refer to an event which hypothetically might have caused death if it were more severe.
**Serious Adverse Reaction (SAR)**	An adverse event that is both serious and, in the opinion of the reporting Investigator, believed with reasonable probability to be due to one of the trial treatments, based on the information provided.
**Suspected Unexpected Serious Adverse Reaction (SUSAR)**	A serious adverse reaction, the nature and severity of which is not consistent with the Reference Safety Information for the medicinal product in question set out:<br>• In the case of a product with a marketing authorisation, in the approved summary of product characteristics (SmPC) for that product<br>• In the case of any other investigational medicinal product, in the approved investigator's brochure (IB) relating to the trial in question

NB: to avoid confusion or misunderstanding of the difference between the terms "serious" and "severe", the following note of clarification is provided: "Severe" is often used to describe intensity of a specific event, which <u>may</u> be of relatively minor medical significance. "Seriousness" is the regulatory definition supplied above.

13.3 Assessment results outside of normal parameters as AEs and SAEs

13.3.1 Clinical

Abnormal clinical findings from medical history or examination will be assessed as to their clinical significance throughout the trial. If an abnormal finding is deemed to be clinically significant, the participant will be informed and appropriate medical care arranged with the permission of the participant as per Section 14.6

13.3.2 Laboratory

Abnormal clinical findings from safety blood tests will be assessed by a (blind to treatment allocation) medically qualified study member. Laboratory AEs will be assessed using specific toxicity grading scales adapted from the FDA Toxicity Grading Scale for Healthy Adult and Adolescent Volunteers Enrolled in Preventive Vaccine Clinical Trials (Appendix C: Toxicity grading scale for lab AEs)

Any abnormal test result deemed clinically significant may be repeated to ensure it is not a single occurrence, if deemed appropriate to do so in the medical opinion of the investigator.

If a repeated test remains clinically significant, the participant will be informed and appropriate medical care arranged as appropriate and with the permission of the volunteer.

13.4 Assessment of severity

The severity of clinical and laboratory adverse events will be assessed according to scales based on FDA toxicity grading scales for healthy adult volunteers enrolled in preventive vaccine clinical trials, listed in the Clinical Study Plan and in Table 44-Table 6 below.

Table 4. Severity grading for local adverse events

Adverse Event	Grade	Intensity
**Pain at injection site**	1	Pain that is easily tolerated
	2	Pain that interferes with daily activity
	3	Pain that prevents daily activity
	4	A&E visit or hospitalization
**Erythema at injection site***	1	2.5 - 5 cm
	2	5.1 - 10 cm
	3	>10 cm

	4	Necrosis or exfoliative dermatitis
**Induration/Swelling at injection site**	1	2.5 – 5 cm and does not interfere with activity
	2	5.1 - 10 cm or interferes with activity
	3	>10 cm or prevents daily activity
	4	Necrosis
***erythema ≤2.5cm is an expected consequence of skin puncture and will therefore not be considered an adverse event**		

Table 5. Severity grading criteria for physical observations.

Vital Signs	Grade 1 (mild)	Grade 2 (moderate)	Grade 3 (severe)	Grade 4 Potentially Life threatening
**Fever (Oral - °C)**	38.0 - 38.4	38.5 – 38.9	39.0 - 40	> 40
**Tachycardia (bpm)***	101 - 115	116 – 130	>130	A&E visit or hospitalisation for arrhythmia
**Bradycardia (bpm)****	50 – 54	45 – 49	<45	A&E visit or hospitalisation for arrhythmia
**Systolic hypertension (mmHg)**	141 - 150	151 – 155	≥155	A&E visit or hospitalization for malignant hypertension
**Diastolic hypertension (mmHg)**	91 - 95	96 – 100	>100	A&E visit or hospitalization for malignant hypertension
**Systolic hypotension (mmHg)*****	85 - 89	80 – 84	<80	A&E visit or hospitalization for hypotensive shock
**Respiratory Rate (breaths per minute)**	17 - 20	21-25	>25	Intubation
***Taken after ≥10 minutes at rest **When resting heart rate is between 60 – 100 beats per minute. Use clinical judgement when characterising bradycardia among some healthy subject populations, for example, conditioned athletes. ***Only if symptomatic (e.g. dizzy/ light-headed)**				

Table 6. Severity grading for local and systemic AEs

GRADE 0	None

**GRADE 1**	Mild: Transient or mild discomfort (< 48 hours); No interference with activity; No medical intervention/therapy required
**GRADE 2**	Moderate: Mild to moderate limitation in activity – some assistance may be needed; no or minimal medical intervention/therapy required
**GRADE 3**	Severe: Marked limitation in activity, some assistance usually required; medical intervention/therapy required.
**GRADE 4**	Potentially Life-threatening: Requires assessment in A&E or hospitalisation

13.5 Assessment of Causality

For every AE, an assessment of the relationship of the event to the administration of the vaccine will be undertaken by the local PI with CI oversight. An interpretation of the causal relationship of the intervention to the AE in question will be made, based on the type of event; the relationship of the event to the time of vaccine administration; and the known biology of the vaccine therapy. Alternative causes of the AE, such as the natural history of pre-existing medical conditions, concomitant therapy, other risk factors and the temporal relationship of the event to vaccination will be considered and investigated. Causality assessment will take place during planned safety reviews, interim analyses (including if the study is paused by the DSMB due to safety concerns) and at the final safety analysis, except for SAEs, which should be assigned by the reporting investigator, immediately. Causality assessment will be recorded on the eCRF.

Table 7. Guidelines for assessing the relationship of vaccine administration to an AE.

**0**	No relationship	No temporal relationship to study product and<br>Alternate aetiology (clinical state, environmental or other interventions); and<br>Does not follow known pattern of response to study product
**1**	Unlikely	Unlikely temporal relationship to study product ***and***<br>Alternate aetiology likely (clinical state, environmental or other interventions) ***and***<br>Does not follow known typical or plausible pattern of response to study product.
**2**	Possible	Reasonable temporal relationship to study product; ***or***<br>Event not readily produced by clinical state, environmental or other interventions; ***or***<br>Similar pattern of response to that seen with other vaccines
**3**	Probable	Reasonable temporal relationship to study product; ***and***

		Event not readily produced by clinical state, environment, or other interventions ***or***<br>Known pattern of response seen with other vaccines
**4**	Definite	Reasonable temporal relationship to study product; ***and***<br>Event not readily produced by clinical state, environment, or other interventions; ***and***<br>Known pattern of response seen with other vaccines

13.6 Procedures for Reporting Adverse Events

13.6.1 Solicited AEs

Participants will be asked to record local and systemic AEs for 7 days (and longer if symptoms persist at day seven, until resolution or stabilisation) following vaccination in the electronic diary (solicited AEs).

13.6.2 Unsolicited AEs

All local and systemic AEs occurring in the 28 days following vaccination observed by the Investigator or reported by the participant, whether or not attributed to study medication, will be recorded in electronic diaries or study database. All AEs that result in a participants' withdrawal from the study will be followed up until a satisfactory resolution occurs, or until a non-study related causality is assigned (if the participant consents to this) as per Section 11.8

SAEs and AESIs will be actively solicited at each study visit throughout the entire trial period.

13.6.3 Medically attended AEs

A medically attended AE, is defined as any adverse event for which the participant seeks medical attention either at hospital or from primary care. This explicitly excludes seeking medical attention solely for a SARS-CoV2 test. Participants will be asked to record any medically attended AEs on their diary cards. Medically attended AEs occurring up to 3 months post boost, will be directly solicited and reviewed at each study visit.

13.7 Reporting Procedures for Serious Adverse Events

In order to comply with current regulations on SAE reporting to regulatory authorities, the event will be documented accurately and notification deadlines respected. SAEs will be reported immediately the local Investigator/study team becomes aware of their occurrence. Copies of all reports will be forwarded for review to the Chief Investigator (as the Sponsor's representative) or delegate within 24 hours of the Investigator being aware of the suspected SAE. The DSMB will be notified of SAEs that

are deemed possibly, probably or definitely related to study interventions; the chair of DSMB will be notified immediately (within 24 hours) of the CI/sponsor being aware of their occurrence. SAE/AESIs will not normally be reported immediately to the ethical committee(s) unless there is a clinically important increase in occurrence rate, an unexpected outcome, or a new event that is likely to affect safety of trial participants, at the discretion of the Chief Investigator and/or DSMB. In addition to the expedited reporting above, the Investigator shall include all SAE/AESIs in the annual Development Safety Update Report (DSUR) report.

Grade 4 laboratory AEs should be reported as SAEs and under the category of outcome of an important medical event.

Cases falling under the Hy's Law should be reported as SAEs. A Hy's Law Case is defined by FDA Guidance for Industry "Drug-Induced Liver Injury: Premarketing Clinical Evaluation" (2009). Any study participant with an increase in Aspartate Aminotransferase (AST) or **Alanine Aminotransferase (ALT) ≥ 3x Upper Limit of Normal (ULN) together with Total Bilirubin ≥2xULN**, **where no other reason can be found to explain the combination of these abnormal results**, e.g., elevated serum alkaline phosphatase (ALP) indicating cholestasis, viral hepatitis A, B or C, another drug capable of causing the observed injury, amongst others.

13.7.1 Events exempt from immediate reporting as SAEs

Hospitalisation for a pre-existing condition, including elective procedures planned prior to study entry, which has not worsened, does not constitute a serious adverse event. A&E attendances should not routinely be reported as SAEs unless they meet the SAE definition described above.

13.8 Expectedness

13.8.1 SAEs

With the exception described below there are no expected serious adverse reactions. All SARs identified as probably, possibly or definitely related will therefore be treated as unexpected and reported as SUSARs.

13.8.1.1 AstraZeneca COVID-19 vaccine (ChAdOx1 nCOV-19)

No SAEs expected. Thromboembolic events associated with thrombocytopaenia are sufficiently rare that they will be reported as AESIs and SUSARs (see section 13.8.3).

13.8.1.2 Pfizer BioNTech (BNT162b2)

Anaphylaxis following immunisation is reported in the BNT162b2 Summary of Product Characteristics as an expected adverse event of unknown frequency. Accordingly, anaphylaxis within 24 hours of

receipt of BNT162b2 will be considered an expected SAR to this vaccine. Acute peripheral facial nerve palsy and lymphadenopathy are described as rare and uncommon (respectively) adverse events following BNT162b2; should these be observed in participants receiving BNT162b2 and no other vaccine, and if they met the criteria for an SAE, these would be considered an expected SAR. If experienced in participants receiving BNT162b2 and another COVID-19 vaccine then they should be classified as 'unexpected'.

13.8.1.3 COVID-19 Vaccine Moderna

Anaphylaxis following receipt of COVID-19 Vaccine Moderna is reported in the Regulation 174 Information for UK healthcare professionals as an expected adverse event of unknown frequency. Accordingly, anaphylaxis within 24 hours of receipt of COVID-19 Vaccine Moderna will be considered an expected SAR to this vaccine. Acute facial swelling (in those with a history of injections of dermatological filler) and peripheral facial nerve palsy are described as rare adverse events following COVID-19 Vaccine Moderna, while lymphadenopathy and injection site urticaria are reported as very common and common (respectively). Should these be observed in participants receiving COVID-19 Vaccine Moderna, and if they met the criteria for an SAE, these would be considered expected SARs.

13.8.1.4 Novavax, NVXCoV2373

13.8.1.5 Cholecystitis and cholecystitis acute are considered expected by the Sponsor for the purpose of expedited reporting of SUSARs and identification of SUSARs Valneva, VLA2001

No SAEs expected

13.8.1.6 Curevac, CVnCoV

No SAEs expected

13.8.1.7 Janssen, Ad26.COV2.S

No SAEs expected. Thromboembolic events associated with thrombocytopaenia are sufficiently rare that they will be reported as AESIs and SUSARs (see section 13.8.3). Foreseeable Adverse Reactions

The foreseeable ARs following vaccination are as follows:

13.8.1.8 AstraZeneca COVID-19 vaccine (ChAdOx1 nCOV-19)

**Local reactions**	**Systemic reactions**		**Laboratory events**
At injection site<br>Common & expected	Common &<br>expected<br>Mild to moderate	Uncommon &<br>expected<br>Mild to moderate	

Tenderness	Fatigue	Abdominal pain	Transient neutropaenia from baseline is common and expected
Pain	Headache	Feeling dizzy	
Warmth	Myalgia	Decreased appetite	
Redness	Arthralgia	Enlarged lymph nodes	
Itching	Nausea or vomiting	Excessive sweating, itchy skin or rash	
Swelling/ bruising at the injection site	Malaise		
Lump at the injection site	Chills		
	Feverishness		
	Fever >38°		
	Coryza (sore throat, runny nose)		
These are expected to be less common after the second dose			

13.8.1.9 Pfizer BioNTech (BNT162b2)

Very common	***Common***	***Uncommon***	***Rare***	***Unknown***
Headache	Injection site redness	Lymphadenopathy	Acute peripheral facial paralysis	Anaphylaxis
Arthralgia	Nausea	Insomnia		Hypersensitivity
Myalgia		Pain in extremity		
Injection site pain/swelling		Malaise		

Fatigue		Injection site pruritis		
Chills				
Pyrexia				

13.8.1.10 COVID-19 Vaccine Moderna

Very common		***Common***	***Uncommon***	***Rare***	***Unknown***
Fever	Injection site swelling	Rash	Itching at injection site	Facial swelling – seen in recipients with history of injection of dermal fillers with 48 hours of vaccination	Anaphylaxis
Headache	Lymphadenopathy	Injection site redness		Facial paralysis	Hypersensitivity
Nausea	Fatigue	Injection site rash			
Vomiting	Chills				
Myalgia	Injection site pain				
Arthralgia					

13.8.1.11 Novavax, NVXCoV2373

(From the IB report of data from clinical trials)

- Reactogenicity is generally mild, and vaccinations were well tolerated.
- Following first vaccination, local reactogenicity is more frequent for the SARS-CoV-2 rS/Matrix-M1 adjuvant regimens than the unadjuvanted or placebo regimens.
- Tenderness and pain were the most frequent local AEs.
- Systemic reactogenicity were individually less frequent but were observed with greater frequency in the SARS-CoV-2 rS/Matrix-M1 adjuvant groups.
- Headache, fatigue, and myalgia were the most frequent systemic AEs.
- Following second vaccination, SARS-CoV-2 rS/Matrix-M1 adjuvant induced greater local and systemic reactogenicity, but the majority of reported symptoms remained at grade ≤ 1.

- Mean duration of reactogenicity events was ≤ 2 days without appreciable change in duration with second vaccination.
- Severe reactogenicity was infrequent (2 events after Dose 1 and 8 events after Dose 2), occurring more often with second vaccination and for systemic events, with placebo subjects citing similar frequencies as those receiving SARS-CoV-2rS.
- No subjects sought medical intervention or refused second vaccination because of reactogenicity.

13.8.1.12 Valneva, VLA2001

(From the IB report of data from clinical trials)

- The vast majority of AEs were assessed as mild or moderate
- Solicited injection site reactions were common, occurring in two thirds of vaccinees. The most common were tenderness and pain.
- The most frequent solicited systemic solicited AEs were headache, fatigue and muscle pain. Fever was uncommon, affecting only 2 participants.
- One AESI was observed, being chilblains. This was felt to be unrelated to the vaccine and a second dose was received without further problems.

13.8.1.13 Curevac, CVnCoV

(From the IB report of data from clinical trials)

- Majority of solicited AEs were mild and transient, generally resolving to normal within 48hrs
- Grade 3 AEs were more common in younger age participants and in females than males.
- Injection site pain is the most common solicited local AE.
- The most common solicited systemic AEs were fatigue, headache, myalgia and chills.
- The rate of grade 3 AEs increased after the second dose administration.
- An allergic reaction and a potential allergic reaction following vaccination was reported from 2 subjects.
- Very few subjects did not receive a second dose due to related AEs.

13.8.1.14 Janssen, Ad26.COV2.S

Very common (>1/10)	***Common (>1/100)***	***Uncommon (<1/100)***	***Rare (<1/1000)***

Injection site pain	Joint pain	Sneezing	Muscle weakness	Allergic reaction
Headache	Coughing	Tremor	Arm/leg pain	Thrombosis with thrombocytopaenia (<1/10,000)
Tiredness	Fever	Throat pain	Backache	
Muscle pain	Chills	Rash	Weakness	
Nausea	Redness and swelling of injection site	Sweating	Feeling generally unwell	

The following adverse events are considered adverse events of special interest.

Table 8. AESIs

**Immunologic**	Anaphylaxis	
**Neurological**	Isolated anosmia/ageusia*<br>Guillain-Barre Syndrome<br>Acute disseminated encephalomyelitis (ADEM)<br>Aseptic meningitis	Meningoencephalitis<br>Peripheral facial nerve palsy<br>Generalised convulsion<br>Myelitis
**Haematological**	Thrombosis**<br>Stroke<br>Thrombocytopaenia***<br>Eosinophilia****	Coagulation disorder (includes coagulopathy, thrombosis, thromboembolism, internal/external bleed and stroke)
**Cardiac**	Acute cardiovascular injury (includes myocarditis, pericarditis, arrhythmias, heart failure, infarction)	
**Dermatological**	Chilblain-like lesions<br>Single organ cutaneous vasculitis	Erythema multiforme<br>Alopecia
**Gastrointestinal**	Acute liver injury †††	Appendicitis
**Respiratory**	ARDS††	

**Renal**	Acute kidney injury	
**Other**	COVID-19 disease†	SARS-CoV2 positivity on a validated test
*In the absence of COVID-19<br>** Excluding superficial thrombophlebitis (including line-associated)<br>*** G3 or above<br>**** This will be used as a marker of skewed Th2 responses and will be routinely monitored in participants attending the COVID-19 Pathway and follow-up visits. Only G2 and above.<br>† In particular, any occurrence of suspected vaccine associated enhanced disease (VAED) as defined by most recent Brighton Collaboration Case Definition (REF)<br>†† In the absence of an infective aetiology (including COVID-19)<br>††† As defined in Hy's Law (see Cases falling under the Hy's Law should be reported as SAEs. A Hy's Law Case is defined by FDA Guidance for Industry "Drug-Induced Liver Injury: Premarketing Clinical Evaluation" (2009). Any study participant with an increase in Aspartate Aminotransferase (AST) or **Alanine Aminotransferase (ALT) ≥ 3x Upper Limit of Normal (ULN) together with Total Bilirubin ≥2xULN, where no other reason can be found to explain the combination of these abnormal results**, e.g., elevated serum alkaline phosphatase (ALP) indicating cholestasis, viral hepatitis A, B or C, another drug capable of causing the observed injury, amongst others.)		

AESIs should be collected and recorded in the AE reporting form in RedCap throughout the duration of this study. These should also be reported as SAEs if they fulfil the definition criteria for SAEs. All AESIs not already reported as SAEs should be included in the reports to the DSMB.

13.8.2 Thrombocytopaenic thrombosis following adenovirus vector vaccination

13.8.2.1 ChAdOx1-nCov19 (Oxford)

Serious thromboembolic events with concurrent thrombocytopenia, sometimes accompanied by bleeding, have occurred very rarely following vaccination with COVID-19 Vaccine AstraZeneca during post-authorisation use. This includes life-threatening and fatal cases presenting as venous thrombosis, including unusual sites such as cerebral venous sinus thrombosis, splanchnic vein thrombosis, as well as arterial thrombosis, combined with thrombocytopenia that can rapidly progress. Multifocal venous and arterial thromboses have been reported in serious cases. The majority of the events occurred within the first 14 days following vaccination but have also been reported after this period. Due to the rarity of these events, thromboembolic events with concurrent thrombocytopaenia will be considered an AESI and reported as a SUSAR.

13.8.2.2 Ad26.COV2.S (Janssen)

A warning about unusual blood clots with low platelets has been added to the product information for COVID-19 Vaccine Janssen and listed as a very rare side effect. This is following evidence in April 2021 from 8 reports from approximately 7 million people vaccinated in the US. All cases occurred in people under 60 years of age within 3 weeks of vaccination, the majority women. Blood clots occurred mostly at unusual sites such as cerebral venous sinus thrombosis (CVST), and in arteries together with low levels of blood platelets and sometimes bleeding. Due to the rarity of these events, thromboembolic events with concurrent thrombocytopaenia will be considered an AESI and reported as a SUSAR. (https://www.ema.europa.eu/en/news/covid-19-vaccine-janssen-ema-finds-possible-link-very-rare-cases-unusual-blood-clots-low-blood)

13.8.3 Disease enhancement following vaccination

Severe COVID-19 disease will be defined as hospitalisation, with further grading of severity according to the WHO ordinal scale (June 2020) [12]. Cases of COVID-19 disease will be examined for the possibility of vaccine associated enhanced disease (VAED). This will be evaluated on the basis of the most recent recommendations of the Brighton Collaboration [13]. Detailed clinical parameters will be collected from medical records and aligned with agreed definitions, as they emerge. Samples will be collected for evaluation of immunological evidence of VAED. Investigations will be defined by the laboratory analysis plan.

13.9 SUSAR Reporting

Under direction of the Chief Investigator, all SUSARs will be reported by the CRO to the relevant Competent Authority and to the REC and other parties as applicable. For fatal and life-threatening SUSARs, this will be done no later than 7 calendar days after the CI, Sponsor or delegate is first aware of the reaction. Any additional relevant information will be reported within 8 calendar days of the initial report. All other SUSARs will be reported within 15 calendar days.

Principal Investigators will be informed of all SUSARs.

13.10 Development Safety Update Reports

A Development Safety Update Report (DSUR) will be prepared by the Sponsor to cover a one year reporting period in line with current UK legislation requirements and the approved safety management plan. This will be submitted to the Competent Authority, Ethics Committee and HRA within 60 calendar days of the defined Data Lock Point (DLP).

13.11 Interim reviews

The safety profile will be assessed on an on-going basis by the Investigators. The CI and relevant Investigators (as per the trial delegation log) will also review safety issues and SAEs as they arise. A review of reactogenicity data will occur after the initial 20 (approximately) participants per arm have received their trial dose, as per section “Safety review”

The DSMB will evaluate safety data every 4-8 weeks and/or as required and will review safety data accumulated when the study is fully recruited. The DSMB may also be consulted should safety concerns arise at any point.

13.12 Safety Holding Rules

There will be no formal pausing rules given the extensive safety database for all vaccines used in this study, however reactogenicity data will be reviewed as per section “Safety review”.

The study can be paused upon advice of the DSMB, Chief Investigator, Study Sponsor, regulatory authority, Ethical Committee(s), for any single event or combination of multiple events which, in their professional opinion, jeopardise the safety of the participants or the reliability of the data.

13.13 Contraception and pregnancy

13.13.1 Contraception

Female participants of childbearing potential are required to use an effective form of contraception until three months after booster immunisation. A woman of childbearing potential is defined as a pre-menopausal female who is capable of becoming pregnant. Menopause can be diagnosed in a woman aged over 50 after one year of amenorrhoea (this applies only if the woman is not using hormonal contraception).

Acceptable forms of contraception for volunteers of female sex include:

- Established use of oral, injected or implanted hormonal methods of contraception
- Placement of an intrauterine device (IUD) or intrauterine system (IUS)
- Total hysterectomy
- Bilateral Tubal Occlusion
- Barrier methods of contraception (condom or occlusive cap with spermicide)
- Male sterilisation, if the vasectomised partner is the sole partner for the subject
- True abstinence, when this is in line with the preferred and usual lifestyle of the subject (Periodic abstinence and withdrawal are not acceptable methods of contraception)

13.13.2 Pregnancy

Should a participant become pregnant during the trial, no further study IMP will be administered. They will be followed up for clinical safety assessment with their ongoing consent and in addition will be followed until pregnancy outcome is determined. We would not routinely perform venepuncture in a pregnant participant unless there is clinical need.

14 Statistics

14.1 Sample size

The primary analysis of this study will be a superiority test between the COVID-19 vaccine arm and the MenACWY arm within each of the three site groups and each of the two cohorts who received 2 doses of BNT162b2 or 2 doses of ChAdOx1 nCov19.

The below sample size calculation is based on the primary analysis of anti-spike protein IgG at D28 post vaccination. The current available data from the ongoing ChAdOx1 nCoV-19 trial suggests the standard deviation of anti-spike IgG at log scale (base 10) measured by standardised ELISA is 0.4 at D28 post 2nd dose.

The sample calculation for this trial is based on the following assumptions:

1. The minimum clinical difference to detect is 1.75 folds difference in GMC between the COVID-19 vaccine and control arms, i.e. 0.243 on log scale (base 10).
2. The standard deviation of the GMC on log scale (base 10) is 0.4 based on the current available data.

Based on the above assumptions, the study will need to recruit 83 participants in each arm of each cohort to achieve 90% of power at two-sided 1% significance level. Analysis will be performed separately for the three groups (A,B and C) each of which have their own control arm. We assume ~25% of study participants will be excluded from the primary analysis due to positive on anti-nuclesocapsid IgG at baseline or loss of follow-up. Therefore, the sample size in each arm will be expanded to approximately 111. The total sample size will be 111*4*2=888 for groups A and C and 111*5*2 for group B in stage 1. For 3 groups, the total sample size will be 888*2 + 1110*1 = 2886. The sample sizes will be 111*2*2=444 in stage 2, and up to 111*5*2=1110 in stage 3. The immunology cohort will used for exploratory analyses to generate hypothesis, and thus no formal sample size calculation was carried out for this cohort. The sample size of approximately 25 per arm was therefore

chosen based on practical constraints. This means we will have around 20 seronegative participants in each arm for analysis.

Based on sample size of 111 per arm, the precisions and 95% confidence intervals of safety events are:

True safety event rate	Precision(normal approximation)	95% exact binomial CI
5%	±4.1%	1.8%-10.9%
10%	±5.6%	5.1%-17.1%
25%	±8.1%	17.3%-34.1%
50%	±9.3%	40.4%-59.6%

14.2 Description of statistical methods

Safety and Reactogenicity

The analysis population will be all randomised participants who received study vaccine. Counts and percentages of each local and systemic solicited adverse reaction from diary cards, and all unsolicited AEs and SAEs will be presented by groups of vaccine revived.

Immunogenicity

The immunogenicity data is expected to be highly skewed with censored data at the lower detection threshold. A value half the lower detection threshold will be used to impute the data, and the data will be log-transformed prior to analysis. The geometric mean concentration and associated 95% confidence interval will be summarised for each group at each time point, by computing the anti-log of the mean of the log-transformed data.

The primary endpoint is anti-spike IgG at Day 28 post vaccination. The geometric mean concentrations (GMC) of anti-spike IgG will be compared between each of the vaccine arms and the Men ACWY arm within each cohort under the hypothesis:

H_0: $GMC_{vaccine} / GMC_{control} = 1$ or $log_{10} GMC_{vaccine} - log_{10} GMC_{control} = 0$;

H_1: $GMC_{vaccine} / GMC_{control} \neq 1$ or $log_{10} GMC_{vaccine} - log_{10} GMC_{control} \neq 0$.

As all the trial participants received two doses of COVID-19 vaccine before enrolling, we would not expect a large proportion of censored data. Therefore, for each group A,B and C we will we will test the above hypothesis using a linear regression model adjusting for randomisation design variables study site and age (<70 and >=70), as well as baseline immunogenicity, duration between 1st- 2nd vaccine, and duration between 2nd to booster vaccine. Six regression model will be fitted for each of the three groups and previous vaccine received (ChAdOx1-nCov19 or BNT162b2). Sensitivity analysis will be carried out to further adjust for other covariates, which will be pre-specified in the statistical analysis plan. If a very high proportion (20%) of censored data are observed in primary endpoint, then consideration will be made to use an alternative outcome as primary outcome, such as live neutralising antibodies against SARS-CoV-2, or pseudo neutralising antibodies.

As an exploratory analysis, we will also undertake target comparisons where the data across the three groups (A, B and C) are combined to allow comparison of different vaccine schedules, especially the homologous prime-boost schedule and heterologous prime-boost schedules. If useful, we will calculate the adjusted difference in Geometric Mean Ratio (GMR) compared to the combined control group for each vaccine schedule enabling an informal indirect comparison. We will then calculate direct comparisons for each homologous prime-boost schedule (ie ChAdOx1/ ChAdOx1/ ChAdOx1 and BNT162b2/ BNT162b2/BNT162b2) versus the relevant heterologous prime-boost schedules i.e. those involving the same primary immunisation. For these comparisons, as they are exploratory we will present the GMR with 95% confidence interval. Other targeted comparisons we be performed as required.The primary analysis will be conducted on the modified intent-to-treat basis, i.e. we will only include people who were negative on anti-nucleocapsid IgG at baseline and whose primary endpoint at D28 post vaccination is available. The statistical tests for the primary analysis will be two-sided and a p-value less than 0.01 will be considered significant. Multiple imputation will be performed as a sensitivity analysis including all participants randomised. Residual analysis will be used to check model fit. If model fit is not satisfactory specification of model covariates will be examined or alternative model will be used.

A fully detailed statistical analysis plan will be prepared and will be signed off by the Chief Investigator and Statistician prior to conducting any data analyses.

14.3 Interim analyses

The interim analysis on immunogenicity will be carried out when the primary endpoint of D28 anti-spike IgG data become available at each stage, while the interim analysis on reactogenicity will be conducted when the 7-day dairy data become available. Due to the urgency in providing evidence to MHRA/JCVI/VTF for policy making on the third dose vaccine recommendation, we presented the preliminary analysis results before the data lock for interim analysis when >95% of primary endpoint

data were available. The aggregated results were only shared with the relevant parties before publishing the full interim analysis.

There will be stopping rules for the interim analyses and the analyses will not affect the continuation of trial.

14.4 Missing data

The level and pattern of the missing data in the baseline variables and outcomes will be reported. The potential causes of any missing data will be investigated and documented as far as possible. Any missing data will be dealt with using methods appropriate to the conjectured missing mechanism and level of missing.

15 Data Management

The Chief Investigator will be responsible for all data that accrues from the study. The sponsor will act as data controller, University of Oxford will act as a data processor under appropriate agreements.

15.1 Access to Data & Data Protection

Direct access will be granted to authorised representatives from the Sponsor, host institution and the regulatory authorities to permit trial-related monitoring, audits and inspections. The study protocol, documentation, data and all other information generated will be held in strict confidence. No information concerning the study or the data will be released to any unauthorised third party, without prior written approval of the sponsor.

15.2 Data Recording

All clinical study data including participant diary will be recorded directly into an EDC system (REDCap) or onto a paper source document for later entry into EDC if direct entry is not available. This includes safety and outcome data. Any additional information that needs recording, but is not relevant for the eCRF (e.g signed consent forms) will be recorded on separate paper source documents. All documents will be stored safely and securely in confidential conditions. The EDC online data is stored on University of Oxford servers.

All participant reported adverse event data (both solicited & unsolicited) will be entered onto electronic diary cards (e-diaries) for a maximum of 28 days following administration of the IMP. The eDiary provides a full audit trial of edits and will be reviewed at time-points as indicated in the schedule of events. Any adverse event continuing beyond the period of the diary will be copied into the eCRF as required for safety review.

The participants will be identified by a unique trial specific number and code in any database. The name and any other identifying detail will NOT be included in any trial data electronic file, with the exception of the electronic diaries, for which consent will be obtained to store the participant email address for quality control purposes. Only site research staff, sponsor staff, PHARMExcel monitors (as delegated by sponsor) and University of Oxford data managers have access to view the email address.

The EDC system (CRF data) uses a relational database (MySQL/ PostgreSQL) via a secure web interface with data checks applied during data entry to ensure data quality. The database includes a complete suite of features which are compliant with GCP, EU and UK regulations and Sponsor security policies, including a full audit trail, user-based privileges, and integration with the institutional LDAP server. The MySQL and PostgreSQL database and the webserver will both be housed on secure servers maintained by the University of Oxford IT personnel. The servers are in a physically secure location in Europe. Backups will be stored in accordance with the IT department schedule of daily, weekly, and monthly retained for one month, three months, and six months, respectively. The IT servers provide a stable, secure, well-maintained, and high capacity data storage environment. REDCap is a widely-used, powerful, reliable, well-supported system. Access to the study's database will be restricted to members of the study team by username and password.

15.3 Record keeping

The Investigators will maintain appropriate medical and research records for this trial, in compliance with GCP and regulatory and institutional requirements for the protection of confidentiality of volunteers. The Chief Investigator, co-Investigators and clinical research nurses will have access to records. The Investigators will permit authorised representatives of the Sponsor(s) and Host institution, as well as ethical and regulatory agencies to examine (and when required by applicable law, to copy) clinical records for the purposes of quality assurance reviews, audits and evaluation of the study safety and progress.

Identifiable information such as contact details will be stored for a minimum of 5 years from the end of the study. This includes storage of consent forms. Storage of these data will be reviewed every 5 years and files will be confidentially destroyed if storage is no longer required. Considerations at the time of this review will include the value of retaining this information for participant safety (e.g. to inform participants of unexpected safety signals emerging from post-licensing surveillance), as a resource for the participants (e.g. if they wish to check which vaccines they have received in the study) and any regulatory requirements. Financial information will be stored for 7 years. De-identified research data maybe be stored indefinitely. If volunteers consent to be contacted for future research, a record of this consent will be recorded, retained and stored securely and separately from the

research data. If volunteers consent to have their samples stored and used in future research, information about their consent form will be retained and stored securely as per Biobanking procedures and SOP.

15.4 Source Data and Case Report Forms (CRFs)

All protocol-required information will be collected in CRFs designed by the Investigator. All source documents will be filed in the participant file. Source documents are original documents, data, and records from which the participant CRF data are obtained. For this study, these will include, but are not limited to, volunteer consent form, blood results, GP response letters, laboratory records, diaries, medical records and correspondence. In the majority of cases, CRF entries will be considered source data as the CRF is the site of the original recording (i.e. there is no other written or electronic record of data). In this study this will include, but is not limited to medical history, medication records, vital signs, physical examination records, urine assessments, adverse event data and details of vaccinations. All source data and participant files will be stored securely. Safety blood results source data will be available in the form of an original print out from each local site's safety lab systems. Safety blood results will be entered into the eCRF using double data entry.

To prevent withdrawal of a participant due to relocation, if there is a nearby participating site and with the consent of the participant, copies of relevant participant research records (such as ICF, paper source documents) will be transferred to the local site using secure email addresses such as nhs.net or by password protected sheets. The electronic research data stored on REDCap will also be transferred to the new site. The original records will be retained by the recruiting site.

15.5 Data Quality

Data collection tools will undergo appropriate validation to ensure that data are collected accurately and completely. Datasets provided for analysis will be subject to quality control processes to ensure analysed data is a true reflection of the source data.

Trial data will be managed in compliance with local University of Oxford data management SOPs. If additional, study specific processes are required, an approved Data Management Plan will be implemented

15.6 Data Sharing

For participants who are also registered on NHS Digital's '*Sign up to be contacted for coronavirus vaccine studies'* service, we will share the minimum amount of information necessary with NHS Digital in order to allow them to update their database so that participants are not contacted about further trials, as participants are permitted only to be in one vaccine study at a time.

Personally identifiable information will be shared with Public Health England regarding SARS-CoV2 PCR test results depending on the most up to date legal requirement to report on Notifiable Diseases at the time.

16 Quality Assurance Procedures

16.1 Risk assessment

The trial will be conducted in accordance with the current approved protocol, GCP, relevant regulations and standard operating procedures. A risk assessment and monitoring plan will be prepared before the study opens and will be reviewed as necessary over the course of the trial to reflect significant changes to the protocol or outcomes of monitoring activities.

16.2 Monitoring

Monitoring will be performed according to Good Clinical Practice (GCP) guidelines by external monitors. Following written SOPs, the monitors will verify that the clinical trial is conducted and data are generated, documented and reported in compliance with the protocol, GCP and the applicable regulatory requirements. The investigator sites will provide direct access to all trial related source data/documents and reports for the purpose of monitoring and auditing by the Sponsor or the Host institution and inspection by local and regulatory authorities

16.3 Trial committees

16.3.1 Trial Steering Committee

A Trial Steering Committee will be formed to oversee the study, and advise the Study Management Committee on key issues of study conduct, including, but not limited to, study pauses due to safety concerns on the advice of the DSMB.

16.3.2 Data Safety Monitoring Board

A Data Safety Monitoring Board (DSMB) will be convened. The DSMB will evaluate frequency of events, safety and efficacy data as specified in the DSMB charter. The DSMB will make recommendations concerning the conduct, continuation or modification of the study for safety reasons to the Trial Steering Committee.

The DSMB will review SAEs or AESIs deemed possibly, probably or definitively related to study interventions. The DSMB will be notified within 24 hours of the CI/Sponsor being aware of their occurrence. The DSMB can recommend placing the study or specific arms of the study on hold if deemed necessary following a study intervention-related SAE.

16.3.3 Study Management Group

Consists of the site Investigators, Sponsor Representatives, PHARMExcel project managers the Laboratory lead for Public Health England.

17 Protocol Deviations

A trial related deviation is a departure from the ethically approved trial protocol or other trial document or process (e.g. consent process or IMP administration) or from Good Clinical Practice (GCP) or any applicable regulatory requirements. Deviations from the protocol will be documented in a protocol deviation form according to SOP R&D/Gen/Admin/009 and filed in the trial master file.

These will be managed as per Sponsor (UHS) SOP R&D/Gen/Admin/009.

18 Serious Breaches

The Medicines for Human Use (Clinical Trials) Regulations contain a requirement for the notification of "serious breaches" to the MHRA within 7 days of the Sponsor becoming aware of the breach.

A serious breach is defined as “A breach of GCP or the trial protocol which is likely to affect to a significant degree –

(a) the safety or physical or mental integrity of the subjects of the trial; or

(b) the scientific value of the trial”.

In the event that a serious breach is suspected the Sponsor must be contacted within 1 working day. In collaboration with the CI the serious breach will be reviewed by the Sponsor and, if appropriate, the Sponsor will report it to the REC committee, Regulatory authority and the relevant NHS host organisation within seven calendar days.

19 Ethical And Regulatory Considerations

19.1 Declaration of Helsinki

The Investigator will ensure that this trial is conducted in accordance with the principles of the Declaration of Helsinki.

19.2 Guidelines for Good Clinical Practice

The Investigator will ensure that this trial is conducted in accordance with relevant regulations and with Good Clinical Practice.

19.3 Approvals

Following Sponsor approval the protocol, informed consent form, participant information sheet and any proposed advertising material will be submitted to an appropriate Research Ethics Committee (REC), HRA (where required), regulatory authorities (MHRA in the UK), and host institution(s) for written approval. No amendments to this protocol will be made without consultation with, and agreement of, the Sponsor.

The Investigator is responsible for ensuring that changes to an approved trial, during the period for which regulatory and ethical committee(s) approval has already been given, are not initiated without regulatory and ethical committee(s)' review and approval except to eliminate apparent immediate hazards to the subject (i.e. as an Urgent Safety Measure).

19.4 Other Ethical Considerations

Study team members are not eligible for participation in the study. Family members of the study team are not barred from inclusion in the trial.

Participants who become eligible for routine booster SARS-CoV-2 immunisation as per national guidelines will not be excluded from participation in the trial; but will be counselled specifically on the risks of receiving an unapproved schedule. In particular, the risks of reduced efficacy and unforeseen safety concerns will be discussed.

19.5 Reporting

The CI shall submit once a year throughout the clinical trial, or on request, an Annual Progress Report to the REC, HRA (where required), host organisation, funder (where required) and Sponsor. In addition, an End of Trial notification and final report will be submitted to the MHRA, the REC, host organisation and Sponsor.

19.6 Transparency in Research

Prior to the recruitment of the first participant, the trial will have been registered on a publicly accessible database. Results will be uploaded to ISRCTN database within 12 months of the end of trial declaration by the CI or their delegate. Where the trial has been registered on multiple public platforms, the trial information will be kept up to date during the trial, and the CI or their delegate will upload results to all those public registries within 12 months of the end of the trial declaration.

19.7 Participant Confidentiality

The study will comply with the General Data Protection Regulation (GDPR) and Data Protection Act 2018, which require data to be de-identified as soon as it is practical to do so. The processing of personal data of participants will be minimised by making use of a unique participant study number only on all study documents and any electronic database(s), with the exception of informed consent forms, participant ID log and electronic diaries. All documents will be stored securely and only accessible by study staff and authorised personnel. The study staff will safeguard the privacy of participants' personal data. A separate confidential file containing identifiable information will be stored in a secured location in accordance with the current data protection legislation. Photographs of vaccination sites if required (with the participants' written, informed consent), will not include the participants' face and will be identified by the date, trial code and subject's unique identifier. Once developed, photographs will be stored as confidential records, as above. This material may be shown to other professional staff, used for educational purposes, or included in a scientific publication.

19.8 Expenses and Benefits

Volunteers will be compensated for their time, the inconvenience of having blood tests and procedures, and their travel expenses. The total amount compensated will depend on the exact number of visits, and whether any repeat or additional visits are necessary. For all trial visits compensation will be calculated according to the following:

- Travel expenses: £15 per visit
- Inconvenience of blood tests: £10 per blood donation
- Time required for visit: £20 per visit

20 Finance And Insurance

20.1 Funding

The study is funded by National Institute Health Research (NIHR), supported by the Vaccine Task Force and DHSC.

Insurance

The Sponsor has a specialist insurance policy in place which would operate in the event of any participant suffering harm as a result of their involvement in the research. NHS indemnity operates in respect of the clinical treatment that is provided.

20.2 Contractual arrangements

Appropriate contractual arrangements will be put in place with all third parties.

21 Publication Policy

The Investigators will be involved in reviewing drafts of the manuscripts, abstracts, press releases and any other publications arising from the study. Data from the study may also be used as part of a thesis for a PhD or MD.

22 Development Of A New Product/ Process Or The Generation Of Intellectual Property

Ownership of IP generated by employees of the Trust vests in the Trust. The Trust will ensure appropriate arrangements are in place as regards any new IP arising from the trial.

23 Archiving

Study data may be stored electronically on a secure server, and paper notes will be kept in a key-locked filing cabinet at the site. All essential documents will be retained for a minimum of 5 years after the study has finished with 5 yearly reviews. The need to store study data for longer in relation to licensing of the vaccine will be subject to ongoing review. For effective vaccines that may be licensed, we may store research data securely at the site at least 15 years after the end of the study, subject to adjustments in clinical trials regulations. Where relevant participants' bank details will be stored for 7 years in line with the site financial policy. De-identified research data maybe be stored indefinitely, but with 5 yearly review.

General archiving procedures will be conducted in compliance to SOP R&D/Gen/Admin/022 SOP for the Archiving of Clinical Trial Data.

24 References

1. Dan, J.M., et al., *Immunological memory to SARS-CoV-2 assessed for up to 8 months after infection.* Science, 2021. **371**(6529).
2. Doria-Rose, N., et al., *Antibody Persistence through 6 Months after the Second Dose of mRNA-1273 Vaccine for Covid-19.* N Engl J Med, 2021.

3. Wu, K., et al., *Serum Neutralizing Activity Elicited by mRNA-1273 Vaccine.* N Engl J Med, 2021.
4. Madhi, S.A., et al., *Efficacy of the ChAdOx1 nCoV-19 Covid-19 Vaccine against the B.1.351 Variant.* N Engl J Med, 2021.
5. Shimabukuro, T., et al., *Reports of Anaphylaxis After Receipt of mRNA COVID-19 Vaccines in the US—December 14, 2020-January 18, 2021.* JAMA, 2021.
6. Public Health England, *Vaccine safety and adverse events following immunisation: The Green Book, Chapter 8.* 2020.
7. Weingartl, H., et al., *Immunization with modified vaccinia virus Ankara-based recombinant vaccine against severe acute respiratory syndrome is associated with enhanced hepatitis in ferrets.* J Virol, 2004. **78**(22): p. 12672-6.
8. Agrawal, A.S., et al., *Immunization with inactivated Middle East Respiratory Syndrome coronavirus vaccine leads to lung immunopathology on challenge with live virus.* Hum Vaccin Immunother, 2016. **12**(9): p. 2351-6.
9. Liu, L., et al., *Anti-spike IgG causes severe acute lung injury by skewing macrophage responses during acute SARS-CoV infection.* JCI Insight, 2019. **4**(4).
10. Munster, V.J., et al., *Protective efficacy of a novel simian adenovirus vaccine against lethal MERS-CoV challenge in a transgenic human DPP4 mouse model.* NPJ Vaccines, 2017. **2**: p. 28.
11. Alharbi, N.K., et al., *Humoral Immunogenicity and Efficacy of a Single Dose of ChAdOx1 MERS Vaccine Candidate in Dromedary Camels.* Sci Rep, 2019. **9**(1): p. 16292.
12. W. H. O. Working Group on the Clinical Characterisation Management of Covid Infection, *A minimal common outcome measure set for COVID-19 clinical research - The Lancet Infectious Diseases.* 2020.
13. Brighton Collaboration. *COVID-19 publications*. [cited 2020; Available from: https://brightoncollaboration.us/covid-19/.

25 Appendix A

25.1 Schedule of visits for general cohort

	Screening	V1	V2	V3	V4	(VPP) Only if enter C19P
**Study timeline**		D0	D28	D84	D365	(D0-D364)
**Study window**		Within 120d of screening	-7/+3	±14	±28	Within 7 days of positive test
**Informed consent**	X*	X				
**Safety bloods**		X	X			X
**Medical history**	X					
**Interim medical history**		X	X	X	X	X
**Physical examination (as required)**		(X)	(X)	(X)	(X)	X
**Urine test (Pregnancy) (if required)**		X				
**COVID-19 vaccination**		X				
**COVID-19 immunogenicity bloods**		X	X	X	X	X
**SARS-Cov-2 viral swab**						X
**Diary card review**			X			X
**SAE/AESI/Medically attended AE check**			X	X	X	X

*Online Consent. Restricted to taking a limited medical history online, contacting the GP for further medical record if required and telephone screening visit(s)

25.2 Schedule of visits for immunology cohorts

	Screening	V1	V2	V3	V4
**Study timeline**		D0	D7	D14	D28
**Study window**		Within 120d of screening	±1	±2	-7/+3
**Informed consent**	X*	X			
**Safety bloods**		X	X		X
**Medical history**	X				
**Interim medical history**		X	X	X	X
**Physical examination (as required)**		(X)	(X)	(X)	(X)
**Urine test (Pregnancy) (if required)**		X			
**COVID-19 vaccination**		X			
**COVID-19 immunogenicity bloods**		X	X	X	X
**SAM strip**		X			X
**Saliva****		X			X
**SARS-Cov-2 viral swab**					
**Diary card review**					X
**SAE/AESI/Medically attended AE check**			X	X	X

*Online Consent. Restricted to taking a limited medical history online, contacting the GP for further medical record if required and telephone screening visit(s)** Where available

25.3 Schedule of additional visits for unblinded control arms in event of NHS national booster campaign

	Additional V1	Additional V2	Additional V3
**Study timeline**	UCD0	UCD14	UCD28
**Study window**	NA	±3	±7
**Informed consent**	X		
**Interim medical history**	X		
**Physical examination (as required)**	(X)	(X)	(X)
**Urine test (Pregnancy) (if required)**	X		
**COVID-19 vaccination**	X		
**COVID-19 immunogenicity bloods**	X	X	X
**Safety bloods**	X		X
**Troponin**	X	X	
**SAM strip***	X*		X*
**Saliva***	X*		X*
**SAE/AESI/Medically attended AE check**	X	X	X

* Immunology cohort only

25.4 Schedule of visits for External vaccine trials participant

	V1	V2	V3	V4	V5
**Study timeline**	D0	D14	D28	D84	D242
**Study window**	N/A	±3	-7/+3	±14	±60
**Informed consent**	X				
**Safety bloods**	X	X	X		
**Medical history**	X				
**Interim medical history**		X	X	X	X
**Physical examination (as required)**	(X)	(X)	(X)	(X)	(X)
**Urine test (Pregnancy) (if required)**	X				
**COVID-19 vaccination**	X				
**COVID-19 immunogenicity bloods**	X	X	X	X	X
**Diary card review**		X	X		
**SAE/AESI/Medically attended AE check**		X	X	X	X
**Troponin**	X	X			

26 Appendix B: Amendment History

Amendment No.	Protocol Version No.	Date issued	Author(s) of changes	Details of Changes made
1	2.0	May 14th 2020	Alasdair Munro	• Amended eligibility criteria to be minimum of 84 days post second COVID-19 vaccine, however minimum of 70 days post second vaccine of ChAdOx-nCoV19 is allowable with Sponsor approval at site.<br>• Removed confirmed SARS-CoV-2 infection as exclusion criteria<br>• Amended pregnancy/contraception as inclusion/exclusion to only be relevant for

				the first 3 months of the trial following vaccination<br>• Clarified diary checks to be performed at follow up visits with daily monitoring for email alerts of Grade 3 AEs<br>• Thrombocytopaenic thrombosis amended to no longer be expected SAE. Now will be considered an AESI and SUSAR<br>• Clarification that CI will be responsible for SUSAR reporting<br>• Mucosal immunity samples only to be collected at D0, 28 and 365<br>• Additional serum sample from immunology cohort at D0, 28 and 365 for functional antibody assays<br>• Removed safety bloods from D14 in the schedule of visits for the immunology cohort. This was an error and conflicted with appendix D<br>• Clarified that participants should have a minimum of 24 hours between receiving documents and attending a screening visit where possible. Sufficient time should be given before consent.<br>• Amended the D28 visit window to -7/+3 days
2	2.1	May 19th 2021	Stephen Saich<br><br>Alasdair Munro	• Removed reference to an alternative dose for Ad26.COV2.S vaccine in section 12.1.7. This was an administrative error has been removed. The table of interventions remains correct.<br>• Removed reference to a group of participants receiving two doses of Men ACWY vaccines. This was an administrative

				error and has been removed. The table of interventions remains correct.<br>• Adjusted blood collection volumes due to align serum sample collection volumes across groups and reduce LiHep sample volumes due to meet lab capacity constraints.<br>• Added mRNA1273 to IMP section of synopsis, previously omitted as administrative error.<br>• Clarification that saliva samples will only be taken where available<br>• Removal of tenderness from the list of solicited AEs collected due to anticipated participant difficulty in differentiating this from pain.<br>• Correction of typographical errors
3	3.0	June 1st 2021	Alasdair Munro	• The treatment arm for half dose CVnCoV (Curevac) in Group C has been changed to a half dose of BNT162b2 (Pfizer) on advice from the UK Vaccine Task Force.<br>• Lay summary and IMP section updated to reflect the reg 174 approval of the Janssen vaccine Ad26.COV2.S.<br>• Inclusion criteria amended to include volunteers who received their first dose of COVID-19 vaccine in February 2021, who are also a minimum of 84 days post boost (or 70 days post second dose of Ox/AZ if site has sponsor approval)<br>• Addition of text to support recruitment "Direct SMS/text message using the NHS vaccine registers"

				• Updated BNT162b2 (Pfizer) storage conditions to 31 days from 5 days in line with national guidance.
	3.1	June 10th 2021	Saul Faust	• Addition of items to be included in follow up CRFs<br>• Correction of typos (2 additional instances of reference to JCVI priority groups 1-3 identified and removed).
	3.2	June 14th 2021	Alasdair Munro	• Clarification that number of participants in each immunology subgroup is approximately 25, and that the allocation of participants to each treatment arm is approximately 111
	4.0	Sept 8th 2021	Alasdair Munro	• Additional secondary endpoint added for immunological outcomes for a later booster immunization (after 7m), and in comparison to early booster (after 3m).<br>• Additional secondary endpoint added for changes in biomarkers of myocardial injury in participants having later booster immunisation via Troponin<br>• Additional section 9.1.7.1 outlining group allocations of unblinded controls randomized to a booster vaccine.<br>• Amended section 9.1.10 to clarify procedures for unblinding the control arm in event of deployment of a national NHS booster vaccine campaign and administer vaccine as part of the study.

				• Amended section 11.4 to update randomisation plan for unblinded control group vaccination visit<br>• Amended section 11.5 to clarify that after unblinding of the control arm, statisticians and investigators will also have been unblinded to their initial allocation.<br>• Amended schedule of visitation (25.3) for additional booster vaccine visits for the unblinded control group (UCD0 – UCD28).<br>• Amended blood sampling tables (28) for additional booster vaccine visits for the unblinded control group (UCD0 – UCD28).<br>• Addition of Troponin blood test to the UCD0 and UCD14 visit.
	5.0	Oct 1st 2021	Alasdair Munro	• Clarification of eligibility criteria for vaccination of unblinded control group, including that pregnancy will not be an exclusion criteria or contraindication to vaccination<br>• Clarification of secondary endpoints for unblinded control group vaccination being approximately 6 months after primary COVID-19 immunisation schedule
	6.0	Oct 21st 2021	Alasdair Munro	• Addition of section on new sub-study of previous external vaccine clinical trial participants who have received NVXCoV2373 as their primary immunization course, receiving a 3rd booster dose<br>• Additional schedule of events for external vaccine clinical trial sub study participants<br>• Additional blood sampling table for external vaccine clinical trial sub study participants

				• Addition of new secondary endpoints for safety, reactogenicity and immunogenicity of external vaccine clinical trial sub study participants<br>• Amended section 11.8 to remove legacy section which is no longer relevant since unblinding of control group<br>• Additional recruitment methods added for the external vaccine trial sub-study<br>• Addition of expected SAE for the Novavax vaccine

27 Appendix C: Toxicity grading scale for lab AEs

**Haematology**			**Lab Range**	**Grade 1**	**Grade 2**	**Grade 3**	**Grade 4**
Haemoglobin Absolute	**Male**	g/l	130 - 170	115-125	100-114	85-99	<85
Haemoglobin Absolute	**Female**		120 - 150	105-113	90-104	80-89	<80
Haemoglobin Change from Baseline (Decrease)			n/a	10-15	16-20	21-50	>50
White Blood Cells	**Elevated**	x109/l	11	11.5-15.00	15.01-20	20.01-25	>25
White Blood Cells	**Low**		4.0	2.5-3.5	1.5-2.49	1.0-1.49	<1.0
Platelets	**Low**		150-400	125-140	100-124	25-99	<25
Neutrophils	**Low**		2.0-7.0	1.5-1.99	1.0-1.49	0.5-0.99	<0.50
Lymphocytes	**Low**		1.0-4.0	0.75-0.99	0.5-0.74	0.25-0.49	<0.25
Eosinophils	**Elevated**	x109/l	0.02 - 0.5	0.65-1.5	1.51-5.00	>5.00	Hypereosinophilia
**Biochemistry**							
Sodium	**Elevated**	mmol/l	145	146-147	148-149	150-155	>155
Sodium	**Low**		135	132-134	130-131	125-129	<125
Potassium	**Elevated**	mmol/l	5	5.1-5.2	5.3-5.4	5.5-6.5	>6.5
Potassium	**Low**		3.5	3.2-3.3	3.1	2.5-3.0	<2.5
Urea	**Elevated**	mmol/l	2.5 - 7.4	8.2-9.3	9.4-11.0	>11.0	Requires dialysis
Creatinine	**Elevated**	µmol/l	49 - 104	1.1-1.5xULN<br>114-156	>1.5-3.0xULN<br>157-312	>3.0xULN<br>>312	Requires dialysis
Bilirubin	**Normal LFTs**	µmol/l	0-21	1.1-1.5xULN<br>23-32	>1.5-2xULN<br>33-42	>2-3xULN<br>43-63	>3xULN<br>≥64
Bilirubin	**Abnormal LFTs**	µmol/l	0 - 21	1.1-1.25xULN<br>23-26	>1.25-1.5xULN<br>27-32	>1.5-1.75xULN<br>33-37	>1.75xULN<br>>37
ALT		IU/l	10 - 45	1.1-2.5xULN<br>49-112	>2.-5xULN<br>113-225	>5-10xULN<br>226-450	>10xUPN<br>>450
Alk Phosphatase	**Elevated**	IU/l	30 -130	1.1-2xULN<br>143-260	>2.-3xULN<br>261-390	>3-10xULN<br>391-1300	>10xULN<br>>1300
Albumin		g/l	32-50	28-31	25-27	<25	-

Normal lab ranges may vary between sites and should be adapted accordingly

28 Appendix D: Blood Sampling

General cohort (sites collecting Lithium Heparin samples)

	**V1**	**V2**	**V3**	**V4**	**(VPP)<br>Only if enter C19P**
**Study timeline**	**D0**	**D28**	**D84**	**D365**	**(D0-D365)**
**Safety bloods**	1 x FBC (up to 2ml)<br>1 x Biochem (up to 5ml)	1 x FBC (up to 2ml)<br>1 x Biochem (up to 5ml)			1 x FBC (up to 2ml)<br>1 x biochem (up to 5ml)
**COVID-19 vaccination**	**X**				
**Primary endpoint**		**Nexelis**<br>Anti-spike IgG			
**Secondary endpoints**	**PHE**<br>Neutralising Ab<br>Anti-N IgG<br><br>**Nexelis**<br>Pseudo-neut Ab<br>Anti-spike IgG<br><br>**IMMUNOTEC**<br>ELIspot	**PHE**<br>Neutralising Ab<br><br>**Nexelis**<br>Pseudo-neut Ab<br><br>**IMMUNOTEC**<br>ELIspot	**PHE**<br>Neutralising Ab<br>Anti-N IgG<br><br>**Nexelis**<br>Pseudo-neut Ab<br>Anti-spike IgG<br><br>**IMMUNOTEC**<br>ELIspot	**PHE**<br>Neutralising Ab<br>Anti-N IgG<br><br>**Nexelis**<br>Pseudo-neut Ab<br>Anti-spike IgG<br><br>**IMMUNOTEC**<br>ELIspot	**PHE**<br>Neutralising Ab<br>Anti-N IgG<br><br>**Nexelis**<br>Pseudo-neut Ab<br>Anti-spike IgG<br><br>**IMMUNOTEC**<br>ELIspot
**No of tubes & colours**	Up to 2x10ml Red<br><br>Up to 2x10ml Green (LiHep)*<br><br>1 x FBC (up to 2ml)<br>1 x Biochem (up to 5ml)	Up to 2x10ml Red<br><br>Up to 2x10ml Green (LiHep)*<br><br>1 x FBC (up to 2ml)<br>1 x Biochem (up to 5ml)	Up to 2x10ml Red<br><br>Up to 2x10ml Green (LiHep)*	Up to 2x10ml Red<br><br>Up to 2x10ml Green (LiHep)*	Up to 2x10ml Red<br><br>Up to 2x10ml Green (LiHep)<br><br>1 x FBC (up to 2ml)<br>1 x Biochem (up to 5ml)
**Total volume per visit**	Up to 47ml	Up to 47ml	Up to 40ml	Up to 40ml	Up to 47ml
**Total volume by end of study**	At any point in the study, a repeat of safety bloods may be recommended at the clinical discretion of the investigator. This will result in up to an extra 7ml per repeat blood sample.			Up to 174ml	+ Up to 47ml per C-19 pathway attended

***Lithium Heparin tube or equivalent**

General Cohort at sites NOT collecting Lithium Heparin samples

	V1	V2	V3	V5	(VPP)<br>Only if enter C19P
**Study timeline**	D0	D28	D84	D365	(D0-D294)
**Safety bloods**	1 x FBC (up to 2ml)<br>1 x Biochem (up to 5ml)	1 x FBC (up to 2ml)<br>1 x Biochem (up to 5ml)			1 x FBC (up to 2ml)<br>1 x Biochem (up to 5ml)
**COVID-19 vaccination**	**X**				
**Primary endpoint**		**Nexelis**<br>Anti-spike IgG			
**Secondary endpoints**	**PHE**<br>Neutralising Ab<br>Anti-N IgG<br><br>**Nexelis**<br>Pseudo-neut Ab<br>Anti-spike IgG	**PHE**<br>Neutralising Ab<br><br>**Nexelis**<br>Pseudo-neut Ab	**PHE**<br>Neutralising Ab<br>Anti-N IgG<br><br>**Nexelis**<br>Pseudo-neut Ab<br>Anti-spike IgG	**PHE**<br>Neutralising Ab<br>Anti-N IgG<br><br>**Nexelis**<br>Pseudo-neut Ab<br>Anti-spike IgG	**PHE**<br>Neutralising Ab<br>Anti-N IgG<br><br>**Nexelis**<br>Pseudo-neut Ab<br>Anti-spike IgG
**No of tubes & colours**	Up to 20ml Red<br><br>1 x FBC (up to 2ml)<br>1 x Biochem (up to 5ml)	Up to 20ml Red<br><br>1 x FBC (up to 2ml)<br>1 x Biochem (up to 5ml)	Up to 20ml Red	Up to 20ml Red	Up to 20ml Red<br><br>1 x FBC (up to 2ml)<br>1 x Biochem (up to 5ml)
**Total volume per visit**	Up to 27ml	Up to 27ml	Up to 20ml	Up to 20ml	Up to 27ml
**Total volume by end of study**	At any point in the study, a repeat of safety bloods may be recommended at the clinical discretion of the investigator. This will result in up to an extra 7ml per repeat blood sample.			Up to 94ml	+ Up to 27ml per C-19 pathway attended

Immunology Cohort

	V1	V1A	V1B	V2	V3	V5	(VPP)<br>Only if C19P
**Study timeline**	**D0**	**D7**	**D14**	**D28**	**D84**	**D365**	**(D0-D294)**
**Safety bloods**	X	X		X			X
**COVID-19 vaccination**	**X**						
**Primary endpoint**				**Nexelis**<br>Anti-spike IgG			
**Secondary endpoints**	**PHE**<br>Neutralising Ab<br>Anti-N IgG<br>**Nexelis**<br>Pseudo-neut Ab<br>Anti-spike IgG<br>**OVG**<br>Functional Abs<br>**IMMUNOTEC**<br>ELISpot<br>ICS	**Nexelis**<br>Anti-spike IgG	**IMMUNOTEC**<br>ELISpot<br>ICS	**PHE**<br>Neutralising Ab<br>**Nexelis**<br>Pseudo-neut Ab<br>**OVG**<br>Functional Abs<br>**IMMUNOTEC**<br>ELISpot	**PHE**<br>Neutralising Ab<br>Anti-N IgG<br>**Nexelis**<br>Pseudo-neut Ab<br>Anti-spike IgG<br>**IMMUNOTEC**<br>ELISpot	**PHE**<br>Neutralising Ab<br>Anti-N IgG<br>**Nexelis**<br>Pseudo-neut Ab<br>Anti-spike IgG<br>**OVG**<br>Functional Abs<br>**IMMUNOTEC**<br>ELISpot	**PHE**<br>Neutralising Ab<br>Anti-N IgG<br>**Nexelis**<br>Pseudo-neut Ab<br>Anti-spike IgG<br>**IMMUNOTEC**<br>ELISpot<br>ICS
**No of tubes & colours**	Up to 20ml Red<br>Up to 40ml Green (LiHep)*<br>1xFBC (up to 2ml)<br>1xBiochem (up to 5ml)	Up to 20ml Red<br>1xFBC (up to 2ml)<br>1xBiochem (up to 5ml)	Up to 20ml Red<br>Up to 40ml Green (LiHep)*	Up to 20ml Red<br>Up to 30ml Green (LiHep)*<br>1xFBC (up to 2ml)<br>1xBiochem (up to 5ml)	Up to 20ml Red<br>Up to 30ml Green (LiHep)*	Up to 20ml Red<br>Up to 30ml Green (LiHep)*	Up to 20ml Red<br>Up to 40ml Green (LiHep)*<br>1xFBC (up to 2ml)<br>1xBiochem (up to 5ml)
**Total vol. per visit**	Up to 67ml	Up to 27ml	Up to 60ml	Up to 57ml	Up to 50ml	Up to 50ml	Up to 67ml
**Total volume (study end)**	At any point in the study, a repeat of safety bloods may be recommended at the clinical discretion of the investigator. This will result in up to an extra 7ml per repeat blood sample.					Up to 311ml	+ Up to 67ml per C-19P attended

*LiHep tube or equivalent

Additional Booster visit bloods for unblinded participants from the control arms

General cohort (with LiHep)

**Study timeline**	**UCD0**	**UCD14**	**UCD28**
**COVID-19 vaccination**	**X**		
**Secondary endpoints**	**PHE**<br>Neutralising Ab<br>Anti-N IgG<br><br>**Nexelis**<br>Pseudo-neut Ab<br>Anti-spike IgG<br><br>**IMMUNOTEC**<br>ELIspot<br><br>Troponin	**Nexelis**<br><br>Anti-spike IgG<br><br>Troponin	**PHE**<br>Neutralising Ab<br><br>**Nexelis**<br>Pseudo-neut Ab<br>Anti-spike IgG<br><br>**IMMUNOTEC**<br>ELIspot
**No of tubes & colours**	Up to 2x10ml Red<br><br>Up to 2x10ml Green (LiHep)*<br><br>1xFBC (up to 2ml)<br>1xBiochem (up to 5ml)	10ml Red	Up to 2x10ml Red<br><br>Up to 2x10ml Green (LiHep)*<br><br>1xFBC (up to 2ml)<br>1xBiochem (up to 5ml)
**Total volume per visit**	Up to 47ml	Up to 10ml	Up to 47ml

*LiHep tube or equivalent

UC = Unblinded Control

General cohort (without LiHep)

**Study timeline**	**UCD0**	**UCD14**	**UCD28**
**COVID-19 vaccination**	**X**		
**Secondary endpoints**	**PHE**<br>Neutralising Ab<br>Anti-N IgG<br><br>**Nexelis**<br>Pseudo-neut Ab<br>Anti-spike IgG<br><br>Troponin	**Nexelis**<br><br>Anti-spike IgG<br><br>Troponin	**PHE**<br>Neutralising Ab<br><br>**Nexelis**<br>Pseudo-neut Ab<br>Anti-spike IgG
**No of tubes & colours**	Up to 20ml Red<br><br>1xFBC (up to 2ml)<br>1xBiochem (up to 5ml)	10ml Red	Up to 20ml Red<br><br>1xFBC (up to 2ml)<br>1xBiochem (up to 5ml)
**Total volume per visit**	Up to 27ml	Up to 10ml	Up to 27ml

Additional Booster visit bloods for unblinded participants from the control arms

Immunology cohort

Study timeline	UCD0	UCD14	UCD28
**COVID-19 vaccination**	**X**		
**Exploratory endpoints**	**PHE**<br>Neutralising Ab<br>Anti-N IgG<br><br>**Nexelis**<br>Pseudo-neut Ab<br>Anti-spike IgG<br><br>**OVG**<br>Functional Abs<br><br>**IMMUNOTEC**<br>ELISpot<br>ICS<br><br>Troponin	**Nexelis**<br><br>Anti-spike IgG<br><br>**IMMUNOTEC**<br>ELISpot<br>ICS<br><br>Troponin	**PHE**<br>Neutralising Ab<br><br>**Nexelis**<br>Pseudo-neut Ab<br>Anti-spike IgG<br><br>**OVG**<br>Functional Abs<br><br>**IMMUNOTEC**<br>ELISpot
**No of tubes & colours**	Up to 20ml Red<br><br>Up to 30ml Green (LiHep)*<br><br>1xFBC (up to 2ml)<br>1xBiochem (up to 5ml)	Up to 20ml Red<br><br>Up to 30ml Green (LiHep)*	Up to 20ml Red<br><br>Up to 30ml Green (LiHep)*<br><br>1xFBC (up to 2ml)<br>1xBiochem (up to 5ml)
**Total vol. per visit**	Up to 57ml	Up to 50ml	Up to 57ml

*LiHep tube or equivalent

UC = Unblinded Control

External Vaccine Trial Sub-Study Participants

Immunology Cohort

	**V1**	**V2**	**V3**	**V4**	**V5**
**Study timeline**	**D0**	**D14**	**D28**	**D84**	**D242**
**Safety bloods**	X	X	X		
**COVID-19 vaccination**	**X**				
**Exploratory Endpoints**	**PHE**<br>Neutralising Ab<br>Anti-N IgG<br><br>**Nexelis**<br>Pseudo-neut Ab<br>Anti-spike IgG<br><br>**OVG**<br>Functional Abs<br><br>**IMMUNOTEC**<br>ELISpot<br>ICS<br><br>Troponin	**Nexelis**<br>Anti-spike IgG<br><br>**IMMUNOTEC**<br>ELISpot<br>ICS<br><br>Troponin	**PHE**<br>Neutralising Ab<br><br>**Nexelis**<br>Pseudo-neut Ab<br>Anti-spike IgG<br><br>**OVG**<br>Functional Abs<br><br>**IMMUNOTEC**<br>ELISpot	**PHE**<br>Neutralising Ab<br>Anti-N IgG<br><br>**Nexelis**<br>Pseudo-neut Ab<br>Anti-spike IgG<br><br>**IMMUNOTEC**<br>ELISpot	**PHE**<br>Neutralising Ab<br>Anti-N IgG<br><br>**Nexelis**<br>Pseudo-neut Ab<br>Anti-spike IgG<br><br>**OVG**<br>Functional Abs<br><br>**IMMUNOTEC**<br>ELISpot
**No of tubes & colours**	Up to 20ml Red<br><br>Up to 40ml Green (LiHep)*<br><br>1xFBC (up to 2ml)<br>1xBiochem (up to 5ml)	Up to 20ml Red<br><br>Up to 40ml Green (LiHep)*<br><br>1xFBC (up to 2ml)<br>1xBiochem (up to 5ml)	Up to 20ml Red<br><br>Up to 30ml Green (LiHep)*<br><br>1xFBC (up to 2ml)<br>1xBiochem (up to 5ml)	Up to 20ml Red<br><br>Up to 30ml Green (LiHep)*	Up to 20ml Red<br><br>Up to 30ml Green (LiHep)*

	V1	V2	V3	V4	V5
**Total vol. per visit**	Up to 67ml	Up to 67ml	Up to 57ml	Up to 50ml	Up to 50ml
**Total volume (study end)**	At any point in the study, a repeat of safety bloods may be recommended at the clinical discretion of the investigator. This will result in up to an extra 7ml per repeat blood sample.				Up to 291ml

* LiHep sample or equivalent, only applicable for sites collecting LiHep samples

External Vaccine Trial Sub-Study Participants

General Cohort (WITH LiHep)

	**V1**	**V2**	**V3**	**V4**	**V5**
**Study timeline**	**D0**	**D14**	**D28**	**D84**	**D242**
**Safety bloods**	X	X	X		
**COVID-19 vaccination**	**X**				
**Exploratory Endpoints**	**PHE**<br>Neutralising Ab<br>Anti-N IgG<br><br>**Nexelis**<br>Pseudo-neut Ab<br>Anti-spike IgG<br><br>**IMMUNOTEC**<br>ELISpot<br><br>Troponin	**Nexelis**<br>Anti-spike IgG<br><br>**IMMUNOTEC**<br>ELISpot<br><br>Troponin	**PHE**<br>Neutralising Ab<br><br>**Nexelis**<br>Pseudo-neut Ab<br>Anti-spike IgG<br><br>**IMMUNOTEC**<br>ELISpot	**PHE**<br>Neutralising Ab<br>Anti-N IgG<br><br>**Nexelis**<br>Pseudo-neut Ab<br>Anti-spike IgG<br><br>**IMMUNOTEC**<br>ELISpot	**PHE**<br>Neutralising Ab<br>Anti-N IgG<br><br>**Nexelis**<br>Pseudo-neut Ab<br>Anti-spike IgG<br><br>**IMMUNOTEC**<br>ELISpot
**No of tubes & colours**	Up to 20ml Red<br><br>Up to 20ml Green (LiHep)*<br><br>1xFBC (up to 2ml)<br>1xBiochem (up to 5ml)	Up to 20ml Red<br><br>Up to 20ml Green (LiHep)*<br><br>1xFBC (up to 2ml)<br>1xBiochem (up to 5ml)	Up to 20ml Red<br><br>Up to 20ml Green (LiHep)*<br><br>1xFBC (up to 2ml)<br>1xBiochem (up to 5ml)	Up to 20ml Red<br><br>Up to 20ml Green (LiHep)*	Up to 20ml Red<br><br>Up to 20ml Green (LiHep)*
**Total vol. per visit**	Up to 47ml	Up to 47ml	Up to 47ml	Up to 40ml	Up to 40ml
**Total volume (study end)**	At any point in the study, a repeat of safety bloods may be recommended at the clinical discretion of the investigator. This will result in up to an extra 7ml per repeat blood sample.				Up to 221ml

External Vaccine Trial Sub-Study Participants

General Cohort (WITHOUT LiHep)

	V1	V2	V3	V4	V5
**Study timeline**	**D0**	**D14**	**D28**	**D84**	**D242**
**Safety bloods**	X	X	X		
**COVID-19 vaccination**	**X**				
**Exploratory Endpoints**	**PHE**<br>Neutralising Ab<br>Anti-N IgG<br><br>**Nexelis**<br>Pseudo-neut Ab<br>Anti-spike IgG<br><br>Troponin	**Nexelis**<br>Anti-spike IgG<br><br>Troponin	**PHE**<br>Neutralising Ab<br><br>**Nexelis**<br>Pseudo-neut Ab<br>Anti-spike IgG	**PHE**<br>Neutralising Ab<br>Anti-N IgG<br><br>**Nexelis**<br>Pseudo-neut Ab<br>Anti-spike IgG	**PHE**<br>Neutralising Ab<br>Anti-N IgG<br><br>**Nexelis**<br>Pseudo-neut Ab<br>Anti-spike IgG
**No of tubes & colours**	Up to 20ml Red<br><br>1xFBC (up to 2ml)<br>1xBiochem (up to 5ml)	Up to 20ml Red<br><br>1xFBC (up to 2ml)<br>1xBiochem (up to 5ml)	Up to 20ml Red<br><br>1xFBC (up to 2ml)<br>1xBiochem (up to 5ml)	Up to 20ml Red	Up to 20ml Red
**Total vol. per visit**	Up to 27ml	Up to 27ml	Up to 27ml	Up to 20ml	Up to 20ml
**Total volume (study end)**	At any point in the study, a repeat of safety bloods may be recommended at the clinical discretion of the investigator. This will result in up to an extra 7ml per repeat blood sample.				Up to 121ml

Imperial Clinical Trials Unit	STATISTICAL ANALYSIS PLAN – MAIN ANALYSIS	Version:1.0

Study Title: A randomised, phase II UK multi-centre study to determine reactogenicity and immunogenicity of booster vaccination against ancestral and novel variants of SARS-CoV-2

Short Title: Evaluating COVID-19 Vaccine Boosters

IRAS Project ID: 299180

EudraCT Number: 2021-002175-19

UHS Study Number: RHM MED1781

Based on the Protocol version V3.2 14th June 2021

Statistical Analysis Plan for COV-BOOST

Written by Lelia Janani[1], Xinxue Liu[2], Victoria Cornelius[1]

[1] Imperial Clinical Trials Unit, Imperial College London

[2] Oxford Vaccine Group, Oxford University

Chief Investigator: Professor Saul Faust
NIHR Southampton Clinical Research Facility
University Hospital Southampton NHS Foundation Trust,
Southampton SO16 6YD

Sponsor: University Hospital Southampton NHS Foundation Trust

Funder: National Institute Health Research (NIHR), supported by the Vaccine Task Force and DHSC.

COV-BOOST
Evaluating COVID-19 vaccine boosters

Imperial Clinical Trials Unit	STATISTICAL ANALYSIS PLAN – MAIN ANALYSIS	Version:1.0

Document version history log:

Version	Date	Authors	Summary of changes made
Version 1.0	07/07/2021	Dr Victoria Cornelius,<br>Dr Xinxue Liu<br>Dr Leila Janani	Version 1.0 is created

Approval Signatures:

NAME	ROLE	SIGNATURE	DATE
	Chief Investigator	Saul N. Faust Digitally signed by Saul N. Faust Date: 2021.08.31 09:09:18 +01'00'	
	Senior Statistician	Victoria Cornelius Digitally signed by Victoria Cornelius Date: 2021.08.27 18:43:47 +01'00'	
	TSC Chair		

Table of Contents

1. Abbreviations 5
2. Introduction 7
2.1. Study summary 7
2.2. Study Flowchart 7
2.3. Purpose and scope of the plan 8
3. Study Objectives / Hypotheses Testing 8
3.1. Co-Primary Objectives 8
3.2. Secondary objectives 8
3.3. Exploratory objectives 8
4. Design 9
4.1. Study Design 9
4.2. Treatment Groups 9
4.3. Study Population 11
4.4. Eligibility Criteria 11
4.5. Blinding and Codebreaking 12
4.6. Sample Size 12
4.7. Randomisation 13
4.8. Schedule of Time and Events 14
4.8.1. Schedule of visits for General cohorts 14
4.8.2. Schedule of visits for Immunology cohorts 15
5. Populations for Analysis 16
6. Variables of Analysis 18
7. Statistical Methodology 20
7.1. General Methodology 20
7.2. Baseline Demographics 21
7.3. The co-primary Safety and Reactogenicity analysis 22
7.3.1. Definition of outcomes 22
7.3.2. Population for analysis 23
The primary analysis population is defined in Section 5 and consists of all randomised participants who received a vaccine dose. The analysis will be repeated for baseline seropositive and seronegative populations. 23
7.3.3. Statistical analysis 23
Missing data 24
7.4. The co-primary immunogenicity analysis 24
7.4.1 Definition of outcome 24
7.4.2 Populations for analysis 25
7.4.3 Statistical analysis 25
7.4.4 Subgroup analyses 26
7.4.5 Missing data 27
7.5. Secondary immunogenicity outcomes Analysis 27
7.5.1 Definition of outcomes 27

7.5.2 Populations for analysis 27
7.6. Interim Analysis 28
7.7. Sensitivity Analysis 28
8. Statistical Considerations 28
8.1. Missing baseline data 28
8.2. Missing safety outcome data 28
8.3. Missing Immunogenicity outcome data 29
8.4. Multiple comparisons 29
9. Software 29
10. Amendments to Version 29
11. References 29

1. Abbreviations

AE	Adverse event
AESI	Adverse Event of Special Interest
AR	Adverse reaction
C-19P	COVID-19 Pathway
ChAdOx1	Chimpanzee adenovirus 1
CI	Chief Investigator
CRF	Case Report Form
DSMB	Data Safety Monitoring Board
DHSC	Department of Health and Social Care
EDC	Electronic Data Capture
ELISPOT	Enzyme-linked Immunospot
FBC	Full blood count
GCP	Good Clinical Practice
GMT	Geometric Mean Titre
GP	General Practitioner
ICS	Intracellular Cytokine Staining
ICF	Informed Consent Form
IV	Intravenous
JCVI	Joint Committee on Vaccination and Immunisation
NHS	National Health Service
NIHR	National Institute for Health Research
PBMC	Peripheral blood mononuclear cell
RES	Research Ethics Service

PB	Post-booster
PI	Principal Investigator
REC	Research Ethics Committee
RSI	Reference Safety Information
SAE	Serious Adverse Event
SAM-strips	Synthetic absorbable matrix strips
SAR	Serious Adverse Reaction
SMPC	Summary of Medicinal Product Characteristics
SOP	Standard Operating Procedure
SUSAR	Suspected Unexpected Serious Adverse Reactions
µg	Microgram
Vp	Viral particle
VTF	Vaccine Task Force
WHO	World Health Organisation

2. Introduction

2.1. Study summary

COV-BOOST is a randomised, phase II UK multi-centre study to determine reactogenicity and immunogenicity of booster vaccination against ancestral and novel variants of SARS-CoV-2. It is a multicentre study conducted through academic and NHS clinical trials sites. The study will consist of 2 or 3 stages. Participants, laboratory and analysing statisticians will remain blind to treatment allocation.

2.2. Study Flowchart

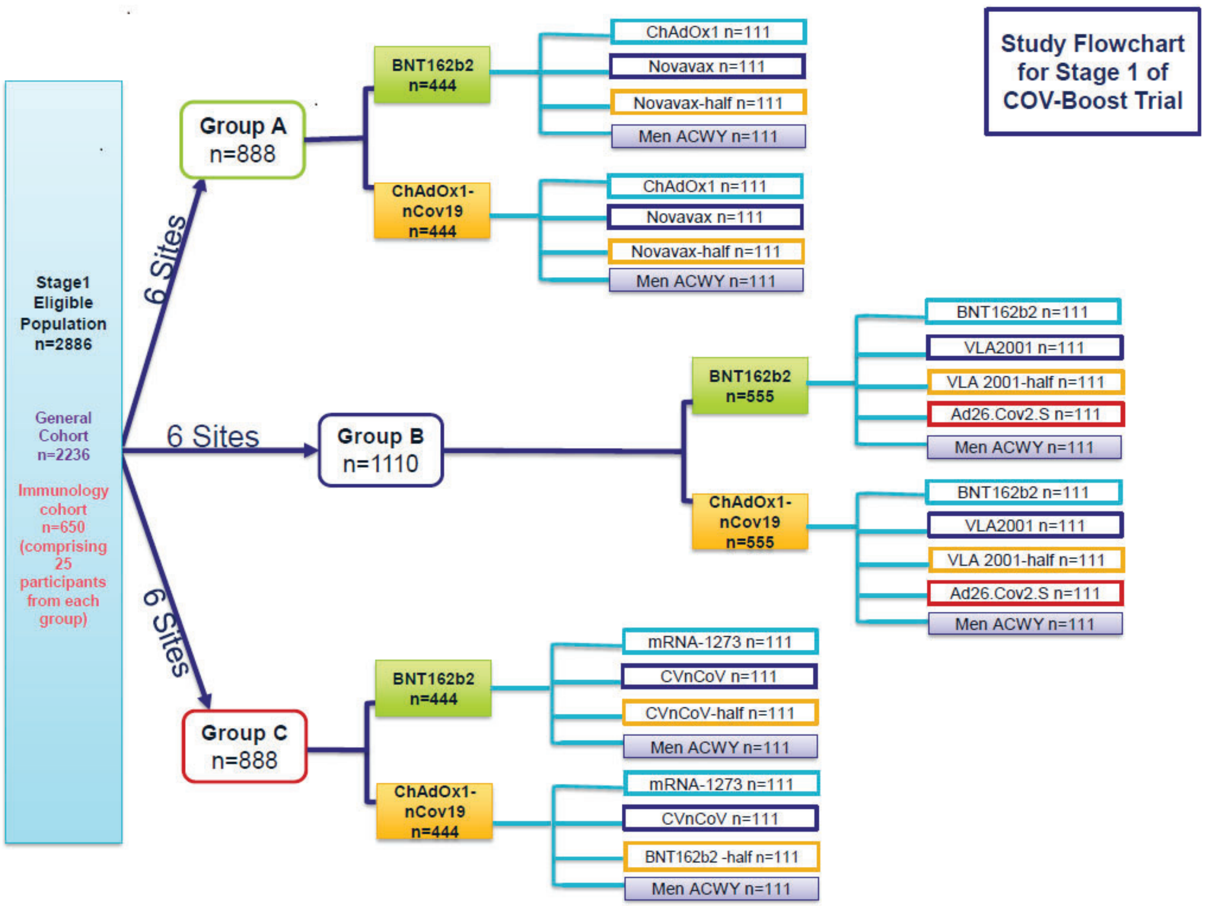

2.3. Purpose and scope of the plan

This document details the proposed analysis of the main paper(s) reporting results from stage 1 of COV-BOOST trial. The results reported in these papers should follow the strategy set out here. The scope of this analysis plan does not extend to include exploratory outcomes. Subsequent analyses of a more exploratory nature will not be bound by this strategy, though they are expected to follow the broad principles set out here. This plan will be used to produce the statistical analysis reports and main trial publications. The statisticians will undertake analysis and provide results for publications based on this plan.

3. Study Objectives / Hypotheses Testing

3.1. Co-Primary Objectives

- To determine the safety and reactogenicity of annual booster doses of vaccines against SARS-CoV-2 following a homologous 2 dose primary regime with either ChAdOx1-nCov19 or BNT162b2
- To determine whether the immune response (based on Anti spike protein IgG) to a booster immunisation of different COVID-19 vaccines is superior to control vaccination for participants who have received two doses of priming vaccination with either ChAdOx1-nCov19 or BNT162b2

3.2. Secondary objectives

- To determine further characterisation of immunogenicity of booster vaccination against ancestral and novel variants of SARS-CoV-2
- To determine safety of booster vaccination against ancestral and novel variants of SARS-CoV-2 using Medically attended adverse events

3.3. Exploratory objectives

- To characterise COVID-19 infections experienced following booster vaccination against ancestral and novel variants of SARS-CoV-2
- To further characterise the blood antibody response to booster vaccination against ancestral and novel variants of SARS-CoV-2

- To characterise and compare the mucosal immune response to immunisation with homologous and heterologous COVID-19 vaccines in the immunology cohort using nasal fluid (collected using SAM-strips) and saliva samples. *

* Only applies to the immunology cohort comprising approximately 25 participants from each group

4. Design

4.1. Study Design

A randomised, phase II UK multi-centre study to determine reactogenicity and immunogenicity of booster vaccination against ancestral and novel variants of SARS-CoV-2.

4.2. Treatment Groups

Stage 1 will be run across three groups (A-C) which will each be enrolled at a different cluster of sites, with a total of 2886 participants.

Site groups A and C will each have 888 participants in total across 4 arms. Within A and C there will be 444 participants who received 2 doses of BNT162b2 prior to enrolment, and 444 participants who received 2 doses of ChAdOx1 nCov19. The second dose will be at least 3 prior to enrolment.

Site group B will have 1110 participants in total across 5 arms. Within B there will be 555 participants who received 2 doses of BNT162b2 prior to enrolment, and 555 participants who received 2 doses of ChAdOx1-nCov19 prior to enrolment.

Each of the three groups in Stage 1 will be enrolled at separate clusters of 6 trial sites to reduce the risk of medication/dosing errors.

Group A: 888 participants (stratified by previous vaccination) will be randomised 1:1:1:1 to receive a booster dose of either **ChAdOx1-nCov19**, **NVX–CoV2373 (Novavax)**, **NVX-CoV2373 half dose**, or **MenACWY (control group)**.

Group B: 1110 participants (stratified by previous vaccination) will be randomised 1:1:1:1:1 to receive a booster dose of either **BNT162b2**, **VLA2001 (Valneva)**, **VLA2001 half dose**, **Ad26.COV2.S (Janssen)** or **MenACWY (control group)**.

Group C: 888 participants (stratified by previous vaccination) will be randomised 1:1:1:1 to receive a booster dose of either **mRNA-1273 (Moderna)**, **CVnCoV (Curevac)**, **BNT162b2 half dose**, or **MenACWY (control group)**.

Table1: Treatment arm allocation by group

Group	2 dose Primary	Subgroup	Booster	Visits
**Group A (n=888)**	ChAdOx1-nCov19 (n=444)	ChAd-ChAd (n=111)	ChAdOx1-nCov19	Day 0, 7* 14*, 28, 84, 365
		ChAd- NVX (n=111)	NVX–CoV2373	
		ChAd- NVX50 (n=111)	NVX–CoV2373 Half Dose[+]	
		ChAd-Men (n=111)	MenACWY	
	BNT162b2 (n=444)	BNT-ChAd (n=111)	ChAdOx1-nCov19	Day 0, 7* 14*, 28, 84, 365
		BNT-NVX (n=111)	NVX–CoV2373	
		BNT-NVX50 (n=111)	NVX–CoV2373 Half Dose[+]	
		BNT-Men (n=111)	Men ACWY	
**Group B (n=1110)**	ChAdOx1-nCov19 (n=555)	ChAd-BNT (n=111)	BNT162b2	Day 0, 7* 14*, 28, 84, 365
		ChAd-VLA (n=111)	VLA2001	
		ChAd-VLA50 (n=111)	VLA2001 Half Dose[+]	
		ChAd-Jan (n==111)	Ad26.COV2.S	
		ChAd-Men (n=111)	Men ACWY	
	BNT162b2 (n=555)	BNT-BNT (n=111)	BNT162b2	Day 0, 7* 14*, 28, 84, 365
		BNT-VLA (n=111)	VLA2001	
		BNT-VLA50 (n=111)	VLA2001 Half Dose[+]	
		BNT-Jan (n=111)	Ad26.COV2.S	
		BNT-Men (n=111)	Men ACWY	

**Group C (n=888)**	ChAdOx1-nCov19 (n=444)	ChAd-Mod (n=111)	mRNA-1273	Day 0, 7* 14*, 28, 84, 365
		ChAd-CVn (n=111)	CVnCoV	
		ChAd-BNT50 (n=111)	BNT162b2 Half Dose+	
		ChAd-Men (n=111)	Men ACWY	
	BNT162b2 (n=444)	BNT-Mod (n=111)	mRNA-1273	Day 0, 7* 14*, 28, 84, 365
		BNT-CVn (n=111)	CVnCoV	
		BNT-BNT50 (n=111)	BNT162b2 Half Dose+	
		BNT-Men (n=111)	Men ACWY	

* Only applies to the immunology cohort comprising approximately 25 participants from each group

+ Half doses of 3 vaccines have been included at the request of the UK Chief Medical Office and Vaccine Task Force to investigate as potential dose sparing regimen for use in the UK or globally due to potential shortage of vaccine supply.

4.3. Study Population

Adults aged 30 years or older who have completed homologous two dose primary course of COVID-19 vaccination with their first dose of administered in December 2020, January or February 2021. Recruitment will be from participants aged 30 years and over (including people 75 years and older). Comorbidities of clinical definition mild/moderate/well-controlled will be permitted. Individuals of all ethnicities will be recruited.

4.4. Eligibility Criteria

The inclusion and exclusion criteria can be found in the most recent version of the protocol. In summary, participants will be aged 30 or above, be in good health but may have a well-controlled mild or moderate co- morbidity. They must have received a homologous 2 dose primary course of COVID-19 vaccination over December 2020, January or February 2021 and be at least 84 days post second vaccination (some participants who had 2 doses of ChAdOx1-nCov19 will be at least 70 days post second vaccination due to timings of NHS vaccine deployment).

4.5. Blinding and Codebreaking

Participants, laboratory will remain blind to treatment allocation. Analysing statisticians will remain blind until the SAP is signed off, as analysis of *targeted comparisons* will unblind them (see below). Clinical staff involved in study delivery will be aware of which vaccine the participant received (arm allocation); the participants themselves will remain blinded to their vaccine allocation. Vaccines will be prepared out of sight of the participant and the blind will be maintained by applying a masking tape over the vaccine syringe.

If the clinical condition of a participant necessitates unblinding of the participant, this will be undertaken according to a trial specific working instruction and group allocation sent to the attending physician. This will be done if unblinding is thought to be relevant and likely to change clinical management. The study team will remain blinded.

4.6. Sample Size

The primary analysis of this study will be a superiority test between each COVID-19 vaccine arm and the MenACWY arm within each of the three groups (A, B and C). The tests within each group will be performed separately for those who received 2 doses of BNT162b2 and 2 doses of ChAdOx1 nCov19. The below sample size calculation is based on the primary analysis of anti-spike protein IgG at D28 post vaccination. The current available data from the ongoing ChAdOx1 nCoV-19 trial suggests the standard deviation of anti-spike IgG at log scale (base 10) measured by standardised ELISA is 0.4 at D28 post 2nd dose.

The sample calculation for this trial is based on the following assumptions:

1. The minimum clinical important difference (MCID) to detect is 1.75 folds difference in GMC between the COVID-19 vaccine and control arms, i.e. 0.243 on log scale (base 10).

2. The standard deviation of the GMC on log scale (base 10) is 0.4 based on the current available data.

Based on the above assumptions, the study will need to recruit 83 participants to each arm and priming vaccine status to achieve 90% power at two-sided 0.01 significance level. Analysis will be performed using the control arm within the associated group (A, B and C). We have taken a conservative approach to calculate the sample size adjusting for three comparisons within groups A

and C by priming vaccine status, and four comparisons in group B by priming vaccine status (as a Bonferroni correction would only require a significance level 0.0167 for three, and 0.0125 for four comparisons). The analysis will use an appropriate p-value multiple correction to adjust for correlation between arms (Dunnett). We assume ~25% of study participants will not be included in the primary analysis due to either a positive anti-nuclesocapsid IgG at baseline or due to loss of follow-up. Therefore, the sample size in each arm will be inflated to approximately 111. The total sample size will be 111*4*2=888 for groups A and C and 111*5*2 for group B in stage 1. For 3 groups, the total sample size will be 888*2 + 1110*1 = 2886. The sample sizes will be 111*2*2=444 in stage 2, and up to 111*5*2=1110 in stage 3.

The immunology cohort will used for exploratory analyses only to generate hypotheses as the number included has been restricted by cost. No formal sample size calculation was carried out for this cohort. The sample size of approximately 25 per arm was chosen based on knowledge of immunological distribution shifts after vaccination and practical constraints. This means we expect around 20 seronegative participants in each arm for analysis.

Based on sample size of 111 per arm, the precision and 95% confidence interval for safety events are:

True safety event rate	Precision in terms of 95% CI normal approximation	95% exact binomial CI
5%	±4.1%	1.8%-10.9%
10%	±5.6%	5.1%-17.1%
25%	±8.1%	17.3%-34.1%
50%	±9.3%	40.4%-59.6%

4.7. Randomisation

A computer-generated randomisation list will be blinded for analysis by the unblinded study statistician for analysis prior to the sign off of the SAP. In Stage 1, participants in groups A and C will be randomised in allocation ratio 1:1:1:1 to the 4 available vaccines in each group. Participants in group B will be randomised in allocation ratio 1:1:1:1:1 to the 5 available vaccines in each group. Permuted random blocks will be used. Randomisation will be stratified by the study sites, age (<70 and ≥70) and priming vaccine (ChaAdOx1 nCOV-19 and BNT162b2 homologous boost). Although we

are not aiming for a formal balance in recruitment of participants over or under the age of 70, this will ensure the recruited population are distributed evenly across all treatment arms. Stata version 15 will be used to produce a randomisations list.

4.8. Schedule of Time and Events

The study visits and procedures will be undertaken by one of the clinical trials team. Each visit is assigned a time-point and a window period, within which the visit will be conducted. If a participant cannot attend a visit, where possible, this will be re-arranged to an in-person visit within the time window. A telephone visit may be conducted instead of the in-person visit to ascertain as much relevant information as possible if the participant is unable to attend a visit in person because of quarantine or self-isolation restrictions and the participant will be out of window if the visit is postponed. For the details of visits, please see the section 11.6 of the protocol.

4.8.1. Schedule of visits for General cohorts

The following table presents the scheduled visits for the general cohort.

	Screening	V1	V2	V3	V4	(VPP) Only if enter C19P
**Study timeline**		D0	D28	D84	D365	(D0-D364)
**Study window**		Within 120d of screening	-7/+3	±14	±28	Within 7 days of positive test
**Informed consent**	X*	X				
**Safety bloods**		X	X			X
**Medical history**	X					
**Interim medical history**		X	X	X	X	X
**Physical examination (as required)**		(X)	(X)	(X)	(X)	X
**Urine test (Pregnancy) (if required)**		X				
**COVID-19 vaccination**		X				
**COVID-19 immunogenicity bloods**		X	X	X	X	X
**SARS-Cov-2 viral swab**						X
**Diary card review**			X			X
**SAE/AESI/Medically attended AE check**			X	X	X	X

*Online Consent. Restricted to taking a limited medical history online, contacting the GP for further medical record if required and telephone screening visit(s).

4.8.2. Schedule of visits for Immunology cohorts

The following table presents the scheduled visits for the immunology cohort. Immunology cohort comprising approximately 25 participants from each group.

	Screening	V1	V2	V3	V4	V5	V6	(VPP) Only if enter C19P
**Study timeline**		D0	D7	D14	D28	D84	D365	(D0-D364)
**Study window**		Within 120d of screening	±1	±2	-7/+3	±14	±28	Within 7 days of positive test
**Informed consent**	X*	X						
**Safety bloods**		X	X		X			X
**Medical history**	X							
**Interim medical history**		X	X	X	X	X	X	X
**Physical examination (as required)**		(X)	(X)	(X)	(X)	(X)	(X)	X
**Urine test (Pregnancy) (if required)**		X						
**COVID-19 vaccination**		X						
**COVID-19 immunogenicity bloods**		X	X	X	X	X	X	X
**SAM strip**		X			X		X	X
**Saliva****		X			X		X	X
**SARS-Cov-2 viral swab**								X
**Diary card review**					X			X
**SAE/AESI/Medically attended AE check**			X	X	X	X	X	X

*Online Consent. Restricted to taking a limited medical history online, contacting the GP for further medical record if required and telephone screening visit(s)** Where available

5. Populations for Analysis

Population	Description
All participants	All participants screened for the trial, to be used for reporting CONSORT diagram
**Primary safety** analysis population	**Safety modified Per-protocol population (with correction for vaccination error)**<br>• All randomised participants who received a study vaccine, including both seronegative and seropositive populations at baseline<br>• Any vaccination error will be accounted for in this analysis set by assigning participants to the vaccination arm of the vaccine they received<br>• Participants who withdraw from the trial will be included up to the date of their study termination<br>• Logged on to eDiary record for at least one day in 7 days.
**Primary immunogenicity** analysis population-	**Seronegative (COVID-19 disease naïve) modified intent-to-treat population**<br>• COVID-19 disease naïve participants who are seronegative at baseline (defined as cut off index <1.0 by the Roche Elecsys anti-Sars-CoV-2 assay at D0)<br>• With no confirmed SARS-CoV-2 infection within 14 days (either pre or post vaccination<br>• Randomised and analysed in the assigned vaccine arm regardless of what they received<br>• Participants with endpoint data available<br>Note- The expected loss of seropositive participants has been accounted for in the power calculation
Secondary analysis population	**Modified intent-to-treat population**<br>• All randomised participants regardless of vaccine given, including both seronegative (disease naïve) and seropositive (previous COVID-19 infection) populations at baseline<br>• Participants with endpoint data available
Secondary analysis population	**Seropositive (evidence of previous COVID-19 infection) modified intent-to-treat population (modified ITT)**<br>• All randomised participants meeting the below criteria:<br>• Seropositive (laboratory evidence of COVID-19 infection) at baseline (defined as cutoff index ≥1.0 by the Roche Elecsys anti-Sars-CoV-2 assay at D0)<br>• With confirmed SARS-CoV-2 infection within 14 days (inclusive post vaccination<br>• Randomised and analysed in the assigned arm for vaccine regardless for what they received<br>• With endpoint data available
C19P analysis population	**COVID-19 positive population**<br>• All randomised participants meeting the below criteria:

	• Participants who received a positive rt-PCR test for SARS-CoV-2 during the study period

6. Variables of Analysis

	Objectives	Outcome Measures	Timepoint(s)
**Co-Primary**	To determine the safety and reactogenicity of annual booster doses of vaccines against SARS-CoV-2 following a homologous 2 dose primary regime	Solicited and unsolicited adverse events<br>Serious adverse events<br>Adverse events of special interest	Solicited adverse events: Day 0-7 after immunisation<br>Unsolicited adverse events: Day 0-28 after immunisation<br>Serious adverse events: throughout the study throughout study up to 12 months<br>Adverse events of special interest: throughout study up to 12 months
**Co-Primary**	To determine whether the immune response to booster immunisation with different COVID-19 vaccines is superior to control vaccination for participants who have received priming vaccination with either ChAdOx1-nCov19 or BNT162b2	Immunogenicity: Anti spike protein IgG	Day 28
**Secondary**	Further characterisation of immunogenicity of booster vaccination against ancestral and novel variants of SARS-CoV-2	Anti-spike immunoglobulins	Day 0, 7*, 28, 84, 365
		Neutralising antibodies against SARS-CoV-2	Day 0, 28, 84, 365
		Anti-nucleocapsid immunoglobulins	Day 0, 84, 365
		Pseudo neutralising antibodies	Day 0, 28, 84, 365

		Cellular immune responses by ELISpot**	Day 0, 14*, 28, 84, 365
		Cellular immune responses by ICS (Th1/Th2)**	D0*, 14*
	Safety of booster vaccination	Medically attended adverse events	Up to 3 months post immunisation
		Changes from baseline in laboratory safety measures	Day 0, 7*, 28,
**Exploratory**	To characterise COVID-19 infections experienced following booster vaccination against ancestral and novel variants of SARS-CoV-2	Anti-spike & anti-nucleocapsid immunoglobulins, neutralising and pseudo-neutralising antibodies, cellular immune response by ICS and ELISpot<br>Genome sequencing of SARS-CoV-2 viruses isolated from infected participants	From vaccination, and within 1 week of a participant being found to be SARS-CoV-2 positive by external testing
	To further characterise the blood antibody response to booster vaccination against ancestral and novel variants of SARS-CoV-2	Functional antibody assays in line with other NISEC studies.	Day 0, 28, 365
	To characterise and compare the mucosal immune response to immunisation with homologous and heterologous COVID-19 vaccines in the immunology cohort using nasal fluid (collected using SAM-strips) and saliva samples. *	IgA & IgG ELISA and exploratory immunological assays	Day 0, 28, 365

* Only applies to the immunology cohort comprising approximately 25 participants from each group

** Only applies to centres collecting Lithium Heparin blood samples (approximately 50% of participants

7. Statistical Methodology

7.1. General Methodology

The primary outcome analysis will be carried out once the primary outcome data for D28 immunogenicity data (anti-spike IgG) becomes available. The co-primary analysis of safety will be analysed at periodic intervals throughout the study using the most recently available data.

All arms will be compared to the control arm within their group among the seronegative ITT populations. All analyses will be conducted separately for participants who receive two doses of ChAdOx1-nCov19 and two doses of BNT162b2 before recruitment and for age groups <70 years and >=70 years.

Censored data are expected for immunogenicity endpoints as these assays normally have a lower limit of detection. Data below the lower limit of detection/quantification will be imputed by a value half the lower limit of detection, prior to log transformation. For data above the high limit of detection/quantification, we will discuss with immunologist before imputing the data. Continuous variables that follow an approximately normal distribution will be summarised using means, standard deviations and range values, and number of missing values. Skewed continuous variables will be summarised using medians/geometric mean (where appropriate), inter-quartile ranges and range values, and number of missing values. Categorical/binary variables will be summarised using frequencies and percentages.

Baseline characteristics will be summarised for each arm to describe the study population. No formal statistical comparisons of baseline characteristics between randomised groups will be conducted. Participant flow from screening, enrolment, through to randomisation, vaccination, follow up and analysis will be presented in a CONSORT flow diagram.(Moher et al 2012) This will contain the numbers of participants randomly assigned to each group, receiving vaccination, completing the study and analysed for the primary outcome. It will also include a breakdown of reasons for withdrawal and their relative time points.

For the primary analyses on immunogenicity (comparing each study vaccine arm with MenACWY as control), the statistical tests will be two-sided and a p-value less than 0.01 will be considered significant. The significance level for all the other secondary analyses will be 2-sided 0.05, unless specified otherwise in the analysis section below.

All analysis will be performed by group and the two-dose primary vaccine schedule. All analysis will be repeated by age groups (< 70, ≥70).

As an exploratory analysis, we will also undertake target comparisons where
the data across the three groups (A, B and C) are combined to allow comparison of different vaccine schedules, especially the homologous boost schedule and heterologous boost schedules. If useful, we will calculate the adjusted difference in Geometric Mean Ratio (GMR) compared to the combined control group for each vaccine schedule enabling an informal indirect comparison. We will then calculate direct comparisons for each
homologous prime-boost schedule (ie ChAdOx1/ ChAdOx1/ ChAdOx1 and BNT162b2/ BNT162b2/ BNT162b2) versus the relevant heterologous boost schedules i.e. those involving the same primary immunisation. For these comparisons, as they are exploratory we will present the GMR with 95% confidence interval. Other targeted comparisons we be performed as required.

Histograms and boxplots will be used to check the distribution and for possible outliers for continuous variables. Outliers will be checked to confirm the validity of the data. Mathematical transformations (log10) will be applied, where appropriate, in order to render a normal distribution.

7.2. Baseline Demographics

For groups A, B and C baseline characteristics will be summarised by treatment arms stratified by the two-dose primary vaccine schedule received (BNT162b2/ BNT162b2 and ChAdOx1-nCov19/ ChAdOx1-nCov19).

7.3. The co-primary Safety and Reactogenicity analysis

7.3.1. Definition of outcomes

The safety and reactogenicity analysis includes multiple outcomes. These comprise of both solicited adverse events over the first 7 days of local and systemic reactions (reactogenicity outcomes), and spontaneously reported (unsolicited) adverse events, serious adverse events (SAEs), suspected unexpected serious adverse reaction (SUSARs). Adverse events will also be detected through routine clinical and blood screening tests over the full 12M study follow up period.

Solicited adverse events

Solicited adverse events are named in the section 13.4 of the protocol.

Adverse Events

Adverse events are all events that have been spontaneously reported (unsolicited) or adverse events detected through routine clinical and blood screening tests.

Adverse Events of Special Interest (AESI)

AESIs are defined as any adverse event identified as being of particular relevance to the IMP's and are listed in Table 8 of the protocol. This assessment will be made at the time of MedDRA coding by the medical coders.

SAEs and SUSARs

An **SAE** is any untoward medical occurrence that:

- Results in death
- Is life-threatening
- Requires inpatient hospitalisation or prolongation of existing hospitalisation
- Results in persistent or significant disability/incapacity
- Consists of a congenital anomaly or birth defect

A **SUSAR** is an SAR, where the nature and severity of which is not consistent with the Reference Safety Information for the medicinal product in question.

More details for AE's and safety outcome definitions can be found in the Protocol in section 13.

7.3.2. Population for analysis

The primary safety analysis population is defined in Section 5. The analysis will be repeated for baseline seropositive (evidence of previous COVID-19 infection) and seronegative (COVID-19 naïve) populations.

7.3.3. Statistical analysis

The SAEs, SUSARs, AESI, and will be coded by MedDRA by medical coders and the frequency for each trial arm will be analysed at the Preferred Term level and again at System Organ Class.

Solicited AEs within first 7 days

The proportion of *any systemic and local AEs* over the first seven days will be presented in Radial plot showing vaccine arm, with one plot per group (A, B and C) by priming vaccine and age group.

The proportion with *at least one moderate or severe* event systemic and local AEs over the first seven days will be presented in Radial plot showing vaccine arm, with one plot per group (A,B and C) and priming vaccine.

The *average duration* of systemic and local AE over the first seven days will be presented in Radial plot showing vaccine arm, with one plot per group (A, B and C) by priming vaccine and age group.

An additional view of reactogenicity outcomes will be provided by group, priming vaccine, and arm in line with the COV-BOOST interim analysis and in line with prior published vaccine trials i.e. the information will be visualised using a stacked bar chart by severity and day (x-axis) for each solicited event in one row (one row per vaccine arm).

SUSARs, SAEs and AESI up to 28 days and then 12 months

All individual SUSARs, SAEs and AESI will be tabulated by vaccine schedule and priming vaccine, and the total number and proportion of participants experiencing at least one event will be calculated for each group (A,B and C).

AE's up to 28 days and then 12 months

All AEs will be summarised at the system organ class (SOC) level and will be tabulated by vaccine schedule and priming vaccine.

We will tabulate the number and proportion with at least one AE as well as the number of AEs. Depending on the repeated AE rate the proportions of participants with at least one event (risk ratio, RR) or rate of events (incidence rate ratio, IRR) between arms will be calculated with 95% CIs. This information will be visualised using a dot plot showing the relative and absolute measures. (Cornelius et.al. 2020)

Further analysis will be considered where there are a sufficiently high rate of AEs. This will be for the calculation of an adjusted between arm comparison (RR or IRR) using negative binomial regression model. The model will be adjusted for randomisation stratification variables.

A tabulation of AEs (proportion with one event and number of total events) at the preferred term will be tabulated by vaccine schedule and priming vaccine will also be provided.

The analysis of blood results will be performed using a distributional method to retain power of the continuous measure using an automated approach to select the most suitable distribution. (Chis Ster 2021) The method will be implemented using user written commands for R (Sauzet 2018).

As the safety analysis in not powered, interpretation will be made on the magnitude of the point estimate and associated precision. Analysis will be repeated for **targeted comparisons** as outlined in section 7.1.

Missing data

It is expected that there will be missing data in the self-reported diary. The completeness of diary data will be described by vaccine arms. There will be no imputation for diary data. The maximum severity will be derived based on all the available data across 7 days. Participants who fail to report any solicited AEs data at all in the 7 days post vaccination will be excluded from the solicited AE analysis.

7.4. The co-primary immunogenicity analysis

This section covers the primary immunogenicity outcome

7.4.1 Definition of outcome

Anti-spike protein IgG at day 28

7.4.2 Populations for analysis

The population for analysis will follow the primary immunogenicity analysis population in Section 5. This is the seronegative (COVID-19 naïve) population and who have outcome data at day 28.

7.4.3 Statistical analysis

The primary endpoint is anti-spike IgG at Day 28 post vaccination. The geometric mean concentrations (GMC) of anti-spike IgG will be compared between each of the vaccine arms and the Men ACWY arm within each cohort under the hypothesis:

H0: GMC vaccine / GMC control =1 or log10 GMC vaccine - log10 GMC control = 0;

H1: GMC vaccine / GMC control ≠1 or log10 GMC vaccine - log10 GMC control ≠ 0.

The primary analyses will be a superiority comparison between the experimental arms individually and the control arm of MenACWY. As each of the groups (A, B and C) have their own control arm, the comparisons will be carried out separately for each group and separately for participants who have received 2-dose of ChaAdOx1 nCOV-19 and those who have received 2-dose of BNT162b2.

The GMC of each arm will be calculated as the antilogarithm of Σ (log10 transformed titre)/n, i.e., as the antilogarithm transformation of the mean of the log10 transformed titre, where n is the number of participants in that arm. The 95% CI will be calculated as the anti-logarithm transformation of the upper and lower limits for a two-sided CI for the mean of the log10 transformed titres (95% CI is reported for descriptive summaries, 99% CI will be used for formal hypothesis testing). Data reported as lower than the detection threshold will be imputed with a value equal to half of the threshold before the transformation.

The Geometric Mean Ratio (GMR) will be calculated as antilogarithm of the difference between the mean of the log10 transformed titre in the vaccine arm and that in the control arm (as the reference), after adjusting for baseline immunogenicity and randomisation design variables (study site, age group) as well as duration between 1st- 2nd vaccine, and duration between 2nd to booster vaccine, in a linear mixed-effect regression model with study site as a random effect. Separate linear regression models will be fitted for each group and priming vaccine. This will allow the GMRs of the study arms to the control arm with each group of A, B, and C to be reported separately for the participants who have received 2-dose of ChaAdOx1 nCOV-19 and those who have received 2-dose of BNT162b2. The two-sided 99% confidence interval of the adjusted GMR

will be calculated as the antilogarithm transformation of the upper and lower 99% CI limits of the adjusted difference of the log10 transformed means.

The proportion of missing outcome data is expected to be very low and no exploration for the impact of missing will be performed. Residual analysis will be performed on the primary population analysis to assess the fit of the model and validity of the model assumptions. If these are not met, suitable adjustments to the model will be made and the impact on the resulting estimates will be examined. If there is an issue with convergence, then the duration between vaccination will be removed from the model.

In the final analysis when full follow-up data is available, the analysis will be repeated for data at all time points e.g. day 7, 84 and 365. A line plot of the mean with a measure of precision (SE or 95%CI) will be plotted across available time points for each arm within a cohort.

Exploratory analyses for targeted comparisons will be performed as outlined in section 7.1. We will used mixed-effects model when combining the data from the three groups to account for potential clustering effects between sites across groups. The site variable will be included as random effect in the model, while fixed effects include age group (<70, >=70),ethnicity, healthcare worker (y/n), gender (M/F), diabetes, respiratory disease, cardiovascular disease, baseline immunogenicity. Controls across different groups will be pooled into one arm in the model in the primary immunogenicity population. To estimate effects for within each age group (<70 and >=70 years) a separate model will be fitted for each group with age group removed as a covariate from the model).

7.4.4 Subgroup analyses

Subgroup analyses for the primary outcome will be conducted by adding interaction terms to the primary model in Error! Reference source not found. after removing the subgroup variables, where needed. The adjusted GMR and two-sided 95% CI will be presented for each subgroup. The subgroups analyses will be performed for all participants in each group of A-C and participants with different prime vaccine status separately and include:

- Age group (<70 years and ≥70 years)
- Sex (Male and Female)

- Comorbidity (With and without comorbidities at baseline, including cardiovascular diseases, respiratory diseases, and diabetes)

7.4.5 Missing data

There will no missing data (by definition) on the outcome in the "seronegative modified intent-to-treat population". For covariates in the subgroup analyses, missing data will not be imputed, and a complete-case analysis will be informed.

7.5. Secondary immunogenicity outcomes Analysis

7.5.1 Definition of outcomes

Secondary outcomes include:

- Anti-spike immunoglobulins at day 0, 7*, 28, 84, 365
- Neutralising antibodies against SARS-CoV-2 at day 0, 28, 84, 365
- Anti-nucleocapsid immunoglobulins at day 0, 84, 365
- Pseudo neutralising antibodies at day 0, 28, 84, 365
- Cellular immune responses by ELISpot** at Day 0, 14*, 28, 84, 365
- Cellular immune responses by ICS (Th1/Th2) at D0*, 14*

* Only applies to the immunology cohort comprising approximately 25 participants from each group

** Only applies to centres collecting Lithium Heparin blood samples (approximately 50% of participants)

7.5.2 Populations for analysis

Secondary outcomes will be performed among the primary immunogenicity analysis population defined in section 5. For the outcomes collected only in the Lithium Heparin blood cohort (Cellular immune responses) or immunology cohort, the analysis population will be those in the primary immunogenicity analysis population enrolling into the Heparin blood cohort (Cellular immune responses) or immunology cohort.

7.5.2 Statistical Analysis

The analysis for all secondary outcomes will follow the same principle and test the same hypothesis as set out in the primary immunogenicity analysis described in section 7.4.3. We will use transformations and comparing the GMRs where suitable.

Secondary outcome analysis will focus on the description of immunogenicity kinetics between different arms. A line plot of the GMC with a measure of precision (SE or 95%CI) or median with IQR will be plotted across available time points for each arm within a group. The between group comparisons at one certain time point may be carried out. In that case, we will follow section 7.1 to calculate GMR and 95%CI. From the model we will report two-sided 95% confidence intervals.

In the final analysis when full follow-up data is available, mixed-effects model to account for site and repeated measurements will be used for data at all time points, e.g. day 0, 7,28 ,84 and 365.

7.6. Interim Analysis

Interim analysis will be performed as requested by the DMC and the Joint Committee on Vaccination and Immunisation (JVCI). No formal interim analysis with statistical boundaries will be undertaken. The DMC will receive regular reports and will informally review safety and reactogenicity data. The JVCI will be provided results for safety, reactogenicity and immunogenicity to inform policy decisions.

7.7. Sensitivity Analysis

As the analysis is repeated for differing populations and there are 4 stratified groups, no sensitivity analysis will be performed.

8. Statistical Considerations

8.1. Missing baseline data

It is likely that missing baseline data will be low as this is collected at the first visit. However, if baseline values are missing mean imputation will be used. This approach will improve statistical efficiency in and is justifiable as randomisation ensures that baselines are independent of arm. (White et al 2005)

8.2. Missing safety outcome data

It is expected that there will be missing data in the self-reported diary. The completeness of diary data will be described by day for each vaccine arm. No imputation will be undertaken for diary data. The maximum severity will be derived based on all the available data across 7 days. We will exclude participants from the denominator in analysis of the 7-day solicited AEs analysis if they failed to report <u>any</u> diary data over the 7 days post vaccination.

8.3. Missing Immunogenicity outcome data

The level and pattern of outcome missing data at baseline and follow up will be reported. The potential causes of any missing data will be investigated and documented as far as possible. Any missing data will be dealt with using methods appropriate to the conjectured missing mechanism and level of missing.

8.4. Multiple comparisons

For primary immunogenicity outcome hypothesis tests we will correct the p-value for multiple comparisons to adjust for correlation between arms (Dunnett's test).

9. Software

We will use R software for the analysis.

10. Amendments to Version

11. References

Chis Ster A, Phillips R, Sauzet O, Cornelius V, 2021, Improving analysis practice of continuous adverse event outcomes in randomised controlled trials – a distributional approach, *Trials*, Vol: 22, ISSN: 1745-62

Cornelius, V., Cro, S. & Phillips, R. Advantages of visualisations to evaluate and communicate adverse event information in randomised controlled trials. *Trials* 21, 1028 (2020).

Moher D, Hopewell S, Schulz KF, Montori V, Gøtzsche PC, Devereaux PJ, Elbourne D, Egger M, Altman DG; CONSORT. CONSORT 2010 explanation and elaboration: updated guidelines for reporting parallel group randomised trials. Int J Surg. 2012;10(1):28-55.

Sauzet O , Rehse J, Breiding JH. DistdichoR a R Package for the distributional dichotomisation of continuous outcomes. arXiv: Applications. 2018.

White IR, Thompson SG. Adjusting for partially missing baseline measurements in randomized trials. Statistics in Medicine. 2005;24(7):993-1007.
